# Phylogenetic analysis of the metazoan carotenoid oxygenase superfamily: a new ancestral gene assemblage of BCO-like (BCOL) proteins

**DOI:** 10.1038/s41598-017-13521-x

**Published:** 2017-10-16

**Authors:** Eugenia Poliakov, Joseph Soucy, Susan Gentleman, Igor B. Rogozin, T. Michael Redmond

**Affiliations:** 1 Laboratory of Retinal Cell & Molecular Biology, National Eye Institute, National Institutes of Health, Bethesda, MD 20892 USA; 2 National Center for Biotechnology Information, National Library of Medicine, National Institutes of Health, Bethesda, MD 20894 USA

## Abstract

Here we describe a new family of carotenoid cleavage oxygenases (CCOs) in metazoans, the BCO2-like (BCOL) clade, which contains lancelet, nematode, and molluscan carotenoid oxygenase sequences. Phylogenetic analysis of CCOs in all kingdoms of life confirmed that the BCOL enzymes are an independent clade of ancient origin. One of the predicted lancelet BCOL proteins, cloned and analyzed for carotenoid cleavage activity in a bacterial carotenoid expression system, had activity similar to lancelet BCO2 proteins, although with a preference for *cis* isomers. Our docking predictions correlated well with the *cis*-favored activity. The extensive expansions of the new animal BCOL family in some species (e.g., lancelet) suggests that the carotenoid cleavage oxygenase superfamily has evolved in the “extremely high turnover” fashion: numerous losses and duplications of this family are likely to reflect complex regulation processes during development, and interactions with the environment. These findings also serve to provide a rationale for the evolution of the BCO-related outlier RPE65 retinol isomerase, an enzyme that does not utilize carotenoids as substrate or perform double-bond cleavage.

## Introduction

Carotenoids comprise a major class of molecules in organisms such as algae and plants, where they are employed in photosynthetic light harvesting and light protection. Furthermore, metabolism of carotenoids yields a large variety of compounds that include color and scent compounds, epoxidation and hydroxylation products, and the abscisic acid (in plants) and retinoic acid (in animals) hormones. Members of the carotenoid cleavage oxygenase (CCO) superfamily are key to many of these processes and include lignostilbene dioxygenase in bacteria (LSDs), apocarotenoid oxygenases in bacteria (ACOs), torulene oxygenases in fungi (TOs), epoxycarotenoid-cleaving enzymes required for abscisic acid biosynthesis in plants (NCEDs), and carotenoid cleaving and double bond isomerizing enzymes in insects (NinaB (=neither inactivation nor afterpotential B, from the electrophysiological phenotype of mutant Drosophila))^1^, carotenoid cleaving enzymes in plants (CCDs) and animals (BCOs). Among vertebrates there are three members of the superfamily: 15,15′-β-carotene oxygenase (BCO1), 9′,10′-β-carotene oxygenase (BCO2), and the visual chromophore isomerase RPE65. All CCO enzymes are non-heme iron (II) oxygenases, with ferrous iron coordinated by four histidines at the axis of a seven-bladed beta-propeller chain fold. Crystal structures of the cyanobacteria *Synechocystis* apocarotenoid oxygenase (ACO)^2^, vertebrate RPE65 retinol isomerase from *Bos taurus* ^3^, plant viviparous14 (VP14) from *Zea mays* ^4^, and bacterial resveratrol-cleaving dioxygenase NOV1 from *Novosphingobium* ^5^ confirm the conservation of overall fold and active site arrangement in all superfamily members. All members of the carotenoid oxygenase superfamily, except for RPE65, oxidatively cleave conjugated polyene substrates. In contrast, the substrate of RPE65 is all*-trans* retinyl ester, implying acquisition of a novel retinol isomerase function by an ancestral carotenoid oxygenase^6–8^. We and others have determined that the first functional RPE65 orthologues appear only in vertebrates and not in either urochordates (tunicates) or cephalochordates (lancelets)^9,10^, although it was suggested that a CCO gene in *Ciona*, a urochordate, resembled RPE65^11^. With the publication of a second whole genome assembly for lancelet, we took a further look at this superfamily in cephalochordates but still did not find any RPE65 orthologues; serendipitously, however, we uncovered a new clade of BCO2-like oxygenases (BCOLs).

Animal BCO1s produce vitamin A aldehyde (all-*trans* retinal) from from carotenoids by symmetric cleavage of β-carotene but can also catalyze oxidative cleavage of other provitamin A carotenoids, such as β-cryptoxanthin, α-carotene and β-apo-8′-carotenal^12^. The non-provitamin A carotenoid lycopene can also be cleaved with a catalytic efficiency similar to that of β-carotene^12,13^. In contrast, animal BCO2s asymmetrically cleave carotenoids. Mouse β-carotene-9′,10′-monooxygenase (BCO2) activity was shown to cleave β-carotene to produce β-apo-10′-apocarotenal and β-ionone^14^ and to catalyze oxidative cleavage of xanthophylls such as zeaxanthin and lutein to generate the 9′,10′- and 9,10- cleavage product rosafluene^15^. Recently β-apo-8′-carotenal and β-apo-12′-carotenal have also been found to be products of BCO2 mediated cleavage^16^. Purified chicken BCO2 has a broad substrate specificity and catalyzes oxidative cleavage of provitamin A carotenoids and non-provitamin A carotenoids^12^. Mammalian BCO2 has been proposed to have the function of preventing excessive accumulation of carotenoids in mitochondria, and its broad substrate specificity is consistent with such a function^15,17^. Genetic studies of “yellow fat” phenotype in cattle, sheep, and chicken BCO2 mutations show that BCO2 is implicated in carotenoid homeostasis of various tissues^18–21^. Given such promiscuous substrate specificity of BCO2-like proteins, we believe they could adapt to the specific carotenoid environment of different animals. Here we describe a new family of CCO of ancient origin (the BCOL clade), containing lancelet, nematode, and molluscan carotenoid oxygenase sequences with a demonstrated BCO2-like activity. The functional significance of this clade in those organisms in which it is expressed is not yet clear; however, given the phylogenetic distribution and the characteristics of the BCOLs, a role in carotenoid management is indicated.

## Results

### Expansion of carotenoid oxygenase genes in Lancelet and a new family of BCO-like oxygenases (BCOL) in animals

**Figure 1:**
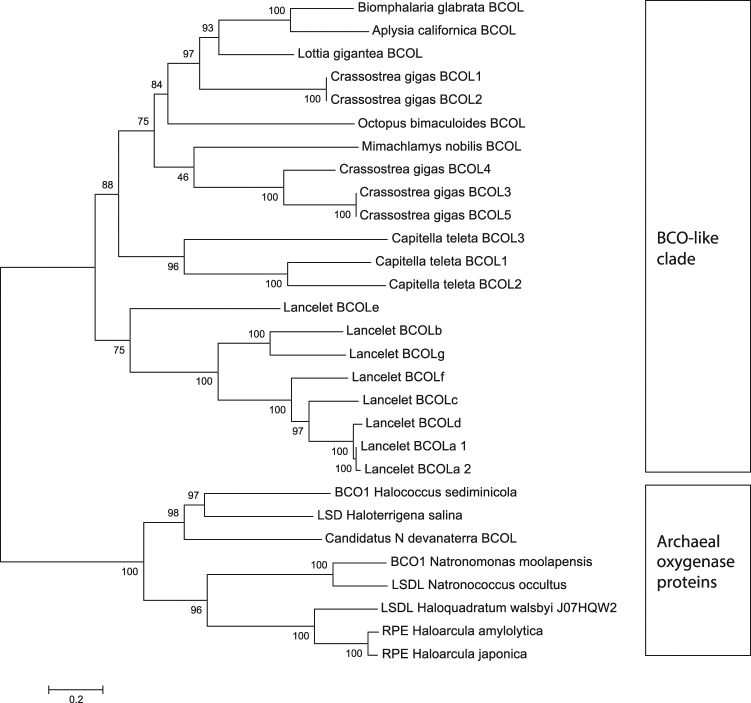
The phylogenetic tree of BCOL proteins. The phylogenetic tree of BCOL proteins was inferred by using the Maximum Likelihood method based on the WAG matrix-based model. Archaeal oxygenase proteins were used as an outgroup. The percentage of trees in which the associated taxa clustered together (bootstrap support values) is shown next to the branches. Initial tree(s) for the heuristic search were obtained automatically by applying Neighbor-Joining and BioNJ algorithms to a matrix of pairwise distances estimated using the WAG model, and then selecting the topology with superior log likelihood value. A discrete Gamma distribution was used to model evolutionary rate differences among sites (2 categories). The rate variation model allowed for some sites to be evolutionarily invariable.

**Figure 2:**
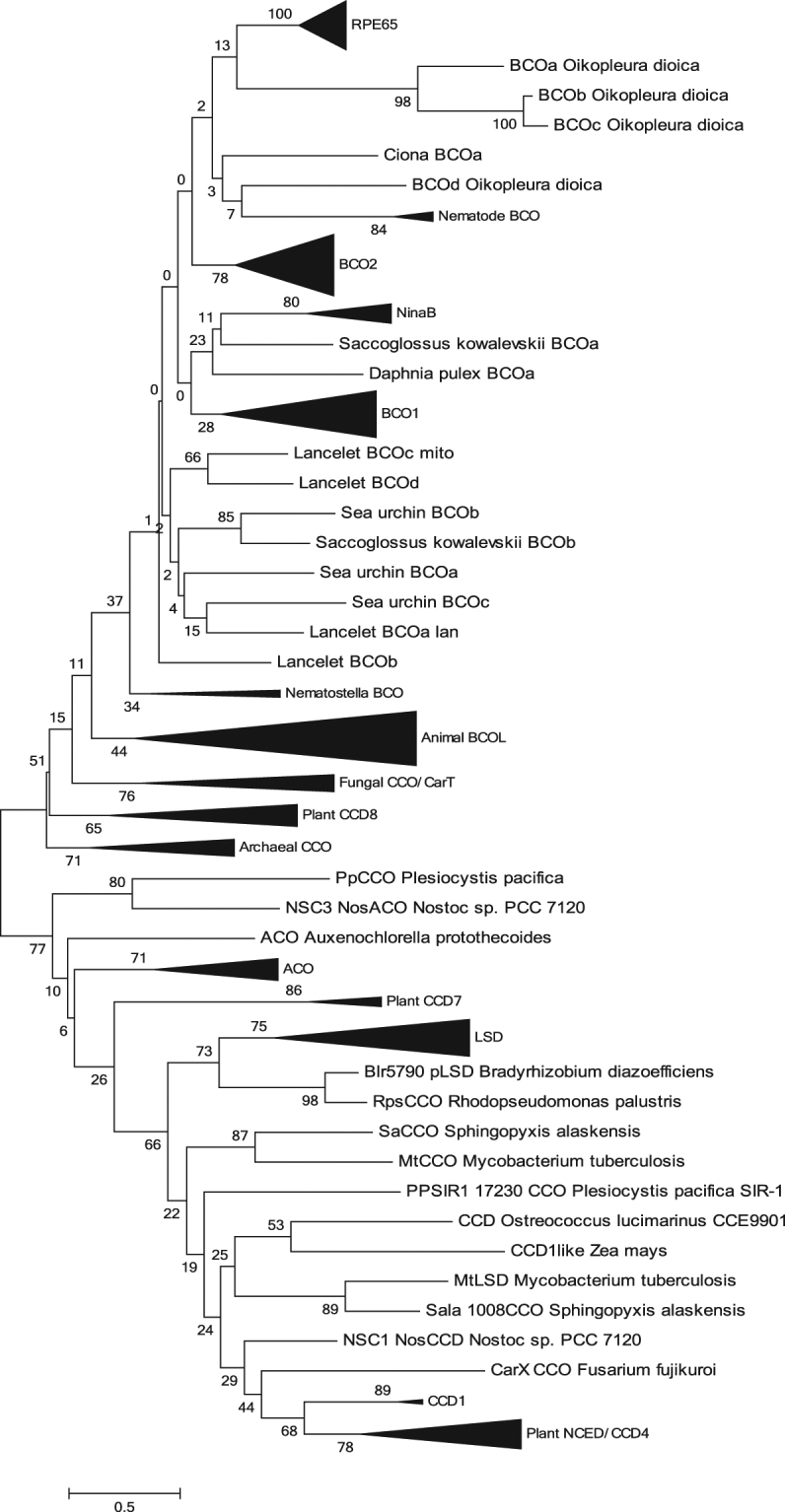
The phylogenetic tree of the carotenoid cleavage oxygenase (CCO) superfamily. The phylogenetic tree was inferred by using the Maximum Likelihood method based on the WAG matrix-based model. The percentage of trees in which the associated taxa clustered together (bootstrep support values) is shown next to the branches. Initial tree(s) for the heuristic search were obtained automatically by applying Neighbor-Join and BioNJ algorithms to a matrix of pairwise distances estimated using the WAG model, and then selecting the topology with superior log likelihood value. A discrete Gamma distribution was used to model evolutionary rate differences among sites (2 categories). The rate variation model allowed for some sites to be evolutionarily invariable.

**Figure 3:**
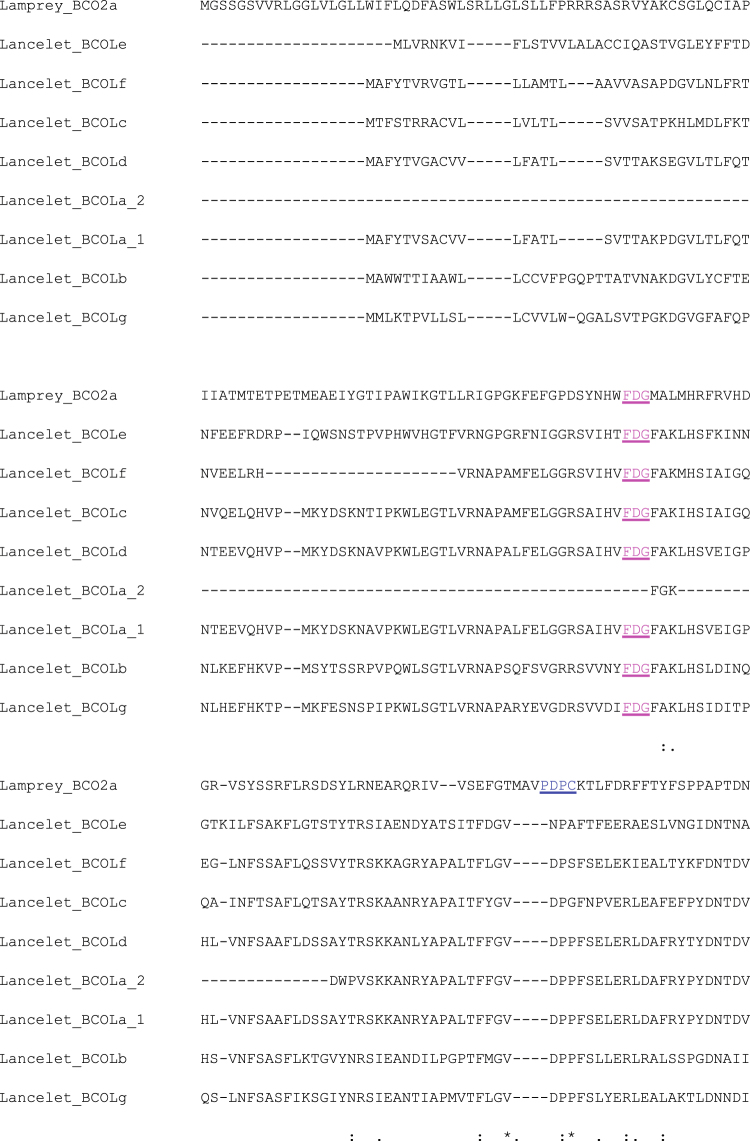
Partial alignment of N-terminal part of all eight lancelet BCOL proteins and Lamprey BCO2a. The FDG motif is shown in purple and the PDPC motif is shown in deep blue. BCOLa_1 (XP_002598440.1, the first BCOL domain, positions 1–512), BCOLa_2 (XP_002598440.1, the second BCOL domain, positions 513–925), BCOLb (XP_002610022.1),_BCOLc (XP_002610868.1), BCOLd (XP_002598424.1), BCOLe (XP_002610869.1), BCOLf (XP_002610867.1), BCOLg (XP_002601288.1), a-g proteins XP numbers.

The carotenoid oxygenase superfamily (CCOs) consists of oxygenases that cleave conjugated polyene substrates with one major exception: RPE65, a retinol isomerase. Earlier we concluded that RPE65 emerged in a common ancestor of jawed and jawless vertebrates, as demonstrated by the presence of active RPE65 in sea lamprey, but not in lower chordates. While the RPE65 clade is separated by a long branch from BCOs in other deuterostome species^10^, questions about putative RPE65 orthologs in lancelet (Cephalochordata) still lingered with addition of new genomes and their better assemblies. To answer this question we set out to better characterize the lancelet carotenoid oxygenase family members by taking a second look at this superfamily in the new draft of the lancelet genome. But, using various query sequences for database searches, we still did not find any putative RPE65 ortholog(s). However, we serendipitously found a novel family of animal BCO-like (BCOL) proteins with members found in lancelet (8 proteins with 23–28% amino acid identity with lamprey BCO2 in protein database analysis) and representatives in several other non-chordate species. Phylogenetic analysis of these BCOL proteins suggests that they form a well-supported clade (Figs 1 and 2, Supplemental Figure S1). According to this analysis, in all four phylogenetic trees obtained, based on the maximum likelihood (ML), neighbor-joining (NJ), maximum parsimony (MP) and minimum evolution (ME) methods, BCOL sequences form a separate clade with the reliable bootstrap support: 44% (ML), 66% (MP), 98% (ME) and 99% (NJ) and thus are likely to be monophyletic (Fig. 2, Supplemental Figure S1). Some of the BCOL proteins have C-terminal extensions, and at least some of these extensions do not seem to be artifacts of gene prediction procedures. All 8 lancelet BCOL proteins contain the four canonical conserved histidines required for iron coordination in the active center. Additionally, all but one contain the highly conserved “FDG” motif of the carotenoid oxygenase superfamily. We suspect that the *BCOLa2* gene which is missing the first methionine and FDG motif is either pseudogene or it is not properly assembled in the genome (Fig. 3). With the publication of the *B. belcheri* genome^22^ in which six *BCOL* genes were found, an extensive number of *BCOL* genes in this genus is supported as being a general characteristic of lancelets, at least in the genus Branchiostoma which contains the majority of extant lancelets.

### BCOL variations in a characteristic motif of the metazoan BCO family and analysis of N-terminal sequences

All previously described metazoan BCO/RPE65 proteins contain a short conserved motif PDPC(K), including the LanBCOa and LanBCOc proteins (Supplementary Figure S2); however, lancelet BCOLg and other newly described BCOL proteins do not contain the PDPC(K) motif (Fig. 3). This motif is located in the center of a large loop spanning beta sheet 1 and 2 over the substrate cleft. In all crystal structures of the CCO superfamily, this loop tends to be mobile (higher b values), and all the comparable sequences to the PDPC(K) motif are on the outer surface of the proteins (Supplementary Figure S3). In the RPE65 crystal, in fact, this motif has never been fully resolved.

Several of the lancelet BCOLs have an extended N-terminal sequence, a characteristic that has been noted in some canonical BCO2s (e.g., Human BCO2a). While the BCO2 N-terminal sequences tend to show the characteristics of being a mitochondrial target peptide^16,23^ (Supplementary Table S1), the N-terminal sequences of the lancelet BCOLs are evaluated by the TargetP^24,25^ program as being signal peptides rather than mitochondrial targeting peptides (Table 1). Such peptides are considered to be possibly indicative of intracellular trafficking to endoplasmic reticulum (ER) membranes.

**Table 1:** Signal peptide prediction with TargetP for BCOL proteins.

name	Species	Length	mTP*	SP*	Other*	Localization
XP_011445777.1	Crassostrea	536	0.033	0.918	0.054	S
XP_011418795.1	Crassostrea	509	0.041	0.941	0.083	S
EKC29689.1	Crassostrea	570	0.431	0.196	0.323	M
XP_005108550.2	Aplysia	553	0.127	0.896	0.011	S
XP_011455847.1	Crassostrea	512	0.023	0.95	0.068	S
XP_014781013.1	Octopus	530	0.024	0.965	0.055	S
XP_009063032.1	Lottia	522	0.017	0.971	0.047	S
XP_019637735.1	B. belcheri	523	0.016	0.92	0.139	S
XP_002610869.1	B. floridae	533	0.025	0.958	0.035	S
XP_019626983.1	B. belcheri	541	0.008	0.983	0.111	S
XP_019637736.1	B. belcheri	520	0.025	0.88	0.073	S
XP_019636352.1	B. belcheri	519	0.019	0.964	0.047	S
XP_019637828.1	B. belcheri	519	0.024	0.956	0.042	S
XP_002610868.1	B. floridae	518	0.232	0.831	0.008	S
XP_002601288.1	B. floridae	531	0.019	0.951	0.058	S
XP_002610022.1	B. floridae	538	0.029	0.969	0.063	S
XP_002610867.1	B. floridae	502	0.045	0.931	0.029	S
XP_019618646.1	B. belcheri	537	0.028	0.974	0.042	S
ELT90061.1	Capitella	518	0.172	0.81	0.034	S
ELT89518.1	Capitella	539	0.021	0.751	0.574	S

*“mTP”, “SP”, “other” are TargetP scores. The actual prediction is in column “Localization” and the characters in this column have the following interpretations: (M) Mitochondrion, the sequence contains a mitochondrial targeting peptide, (S) Secretory pathway, the sequence contains a signal peptide, (other) any other location.

### Catalytic activity and substrate specificity of three lancelet members of BCO family in *E. coli*

**Figure 4:**
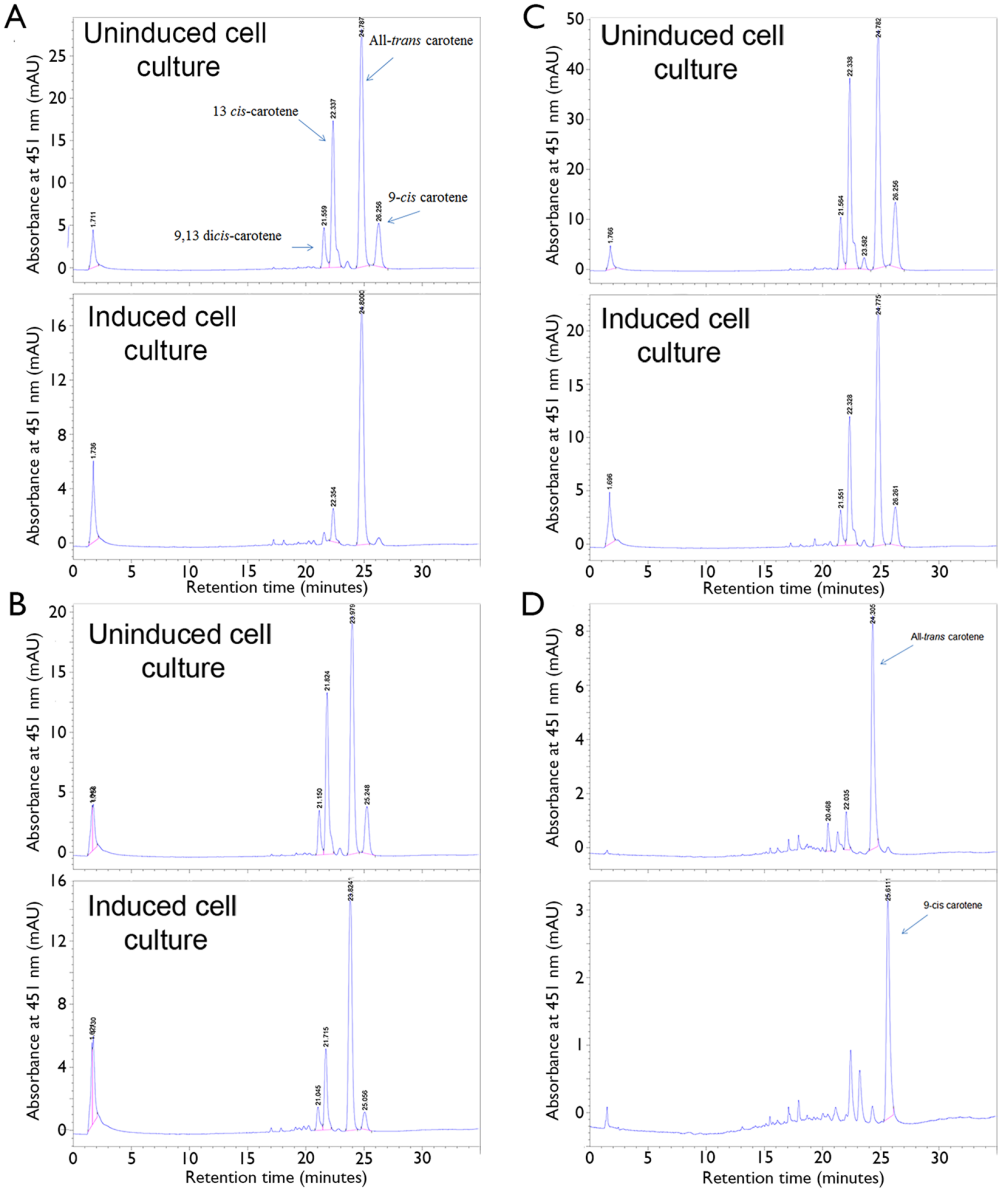
Reverse phase HPLC analysis of β-carotene cleavage by Lancelet BCOLg (**A**), lanBCOa (**B**), lanBCOc (**C**). HPLC profile of all-*trans* β-carotene and 9-*cis* β-carotene are presented in D panel.

To investigate if BCOL proteins have carotenoid oxygenase activity, three lancelet proteins were cloned into into pBADtopo vectors for assay in β-carotene and lycopene-producing *E. coli*. We selected lancelet BCOa (LanBCOa), which seemed to be the closest to vertebrate RPE65 clades, lancelet BCOc (LanBCOc), as close to vertebrate BCO2 clades, according to our database searches and phylogenetic analyses (see Fig. 1 in the Poliakov and co-workers paper^10^), and we also chose BCOLg, one of the BCOL proteins. All three proteins including BCOLg exhibited comparable β-carotene and lycopene cleavage activity in carotenoid-producing *E. coli*. (Table 2). We did not observe preference for lycopene or β-carotene for any of these three proteins. On the other hand, several examined canonical BCO2 proteins demonstrated clear preference for lycopene in our system (Table 2). We found that BCOLg enzyme and, to some degree, LanBCOa do not cleave all-*trans* β-carotene efficiently and appear to be more preferentially *cis*-carotenoid cleavage enzymes, as compared to LanBCOc (Fig. 4a–c respectively).

**Table 2:** Catalytic activity of BCO2 proteins (% substrate left compared to uninduced control).

Sequence name	lycopene	β-carotene
	Top10 cells	Top10 cells
Human BCO2a	44 ± 4	40 ± 3
Human BCO2b	22 ± 3	32 ± 2
Ciona RPE65 like (ciBCOa)	66 ± 10	43 ± 5
Nematostella BCOa	2.0 ± 0.7	60 ± 15
Petromyzon BCO2a	30 ± 2	108 ± 18
LanBCOc	33 ± 1	35 ± 2
LanBCOa	59 ± 11	57 ± 6
BCOLg	31 ± 4	39 ± 1

**Figure 5:**
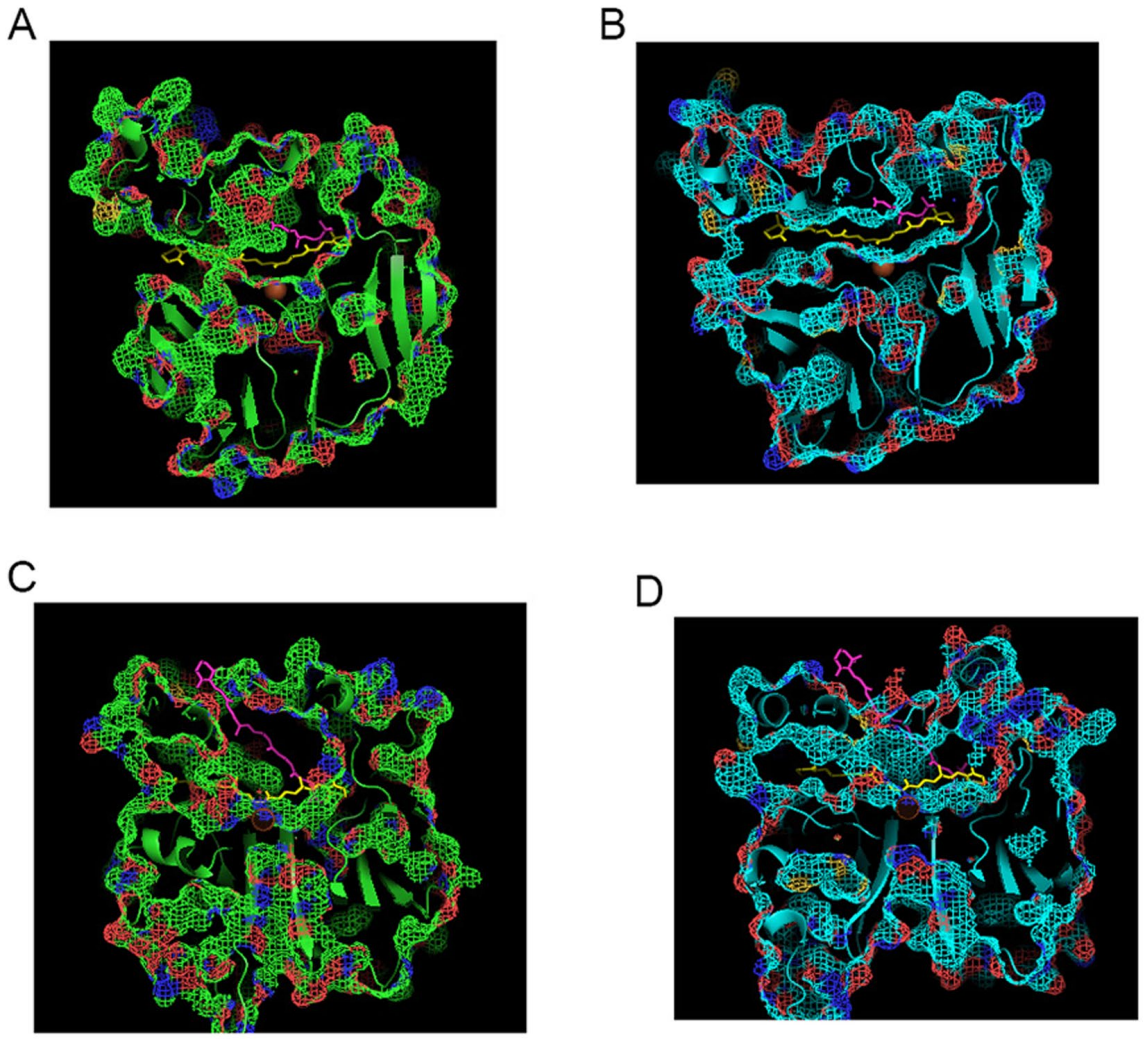
Comparison of β-carotene isomers dockings in model of Lancelet BCOLg and LanBCOc. (**A**,**C**) All*-trans *β-carotene (red) docked on the BCOLg model (green protein structure). All*-trans *β-carotene (yellow) docked on the lanBCOc model is superimposed on BCOLg model. (**B**,**D**) All-*trans *β-carotene (yellow) docked on the lanBCOc model (blue protein structure). All-*trans *β-carotene (red) docked on the BCOLg model is superimposed on the lanBCOc model.

To explain this trend we performed modeling of substrate isomer docking (13-*cis*, 9-*cis* and all-*trans* isomers of β-carotene) in LanBCOa, LanBCOc and BCOLg. Modeling of BCOs was done using the Swiss model random coil of 4F30 crystal of bovine RPE65. We observed a lower binding energy of the all-*trans *isomer to LanBCOc protein and higher binding energy of all-*trans* β-carotene to LanBCOa or BCOLg proteins (Table 3). All-*trans* β-carotene did not dock properly in an active site of BCOLg and the area where substrate docked in LanBCOc is blocked in BCOLg, and vice versa (Fig. 5a–d). Because in our system β-carotene is synthesized in cells from lycopene, a lower proportion of *cis* isomers of β-carotene in the thermodynamic equilibrium mixture of isomers in induced BCOLg-present culture could be explained by direct cleavage of *cis* isomers of β-carotene and/or by lower accumulation of these from the corresponding isomers of lycopene. In both cases, the BCOLg enzyme prefers to cleave *cis* isomers of substrates. Similarly, ferret BCO2 was shown to have low activity towards all-*trans* lycopene and higher activity towards 5-*cis* and 13-*cis* isomers of lycopene^26^.

**Table 3:** Autodock Vina modeling of affinity of beta-carotene isomers to Lancelet BCOs. The lowest (best) docking energy is used.

Lancelet BCO sequence name	All-*trans* carotene (kcal/mol)	9-*cis* carotene (kcal/mol)	13-*cis* carotene (kcal/mol)
BCOLg	−9.9	−10.1	−12.2
LanBCOa	−9.0	−10.9	−12.0
LanBCOc	−12.1	−12.2	−11.7

### Only two mutations of non-active site residues P108S and N190D in mouse BCO2 change specificity of enzyme towards lycopene in TOP10 cells

The specificity of carotenoid cleavage oxygenases is very broad and evolutionarily flexible. Mammalian and chicken BCO1 and BCO2 can cleave some β-apocarotenals while Fusarium carotenoid cleavage enzyme (CCO) CarX and NCS3 Nostoc ACO can cleave β-carotene, torulene and β-Apo-8′-carotenal^12,27–29^.

To examine the substrate specificity of canonical BCO2 clade enzymes we analysed catalytic activity of one BCO2 protein from the animal phylum Cnidaria (Nematostella BCOc) and several from Chordata. Together with Lancelet BCOa and BCOc we examined tunicate Ciona BCOa (previously labeled as ciRPE65), jawless veterbrate Petromyzon BCO2a, and jawed vertebrate mouse BCO2 and human BCO2 long and short isoform. We determined that Nematostella BCOa is much more specific to lycopene cleavage compared to β-carotene, and that Petromyzon BCO2a does not cleave β-carotene but can cleave lycopene, while mammalian mouse and human long (579 aa) and short (545 aa) BCO2s as well as LanBCOc and LanBCOa do not show preference to lycopene vs β-carotene in bacterial system. Ciona BCOa (previously labeled as ciRPE65) prefers β-carotene as substrate in TOP10 *E. coli* (Table 2). We observed that specificity of BCO2 proteins is very species-specific and do not follow any evolutionary trends.

**Figure 6:**
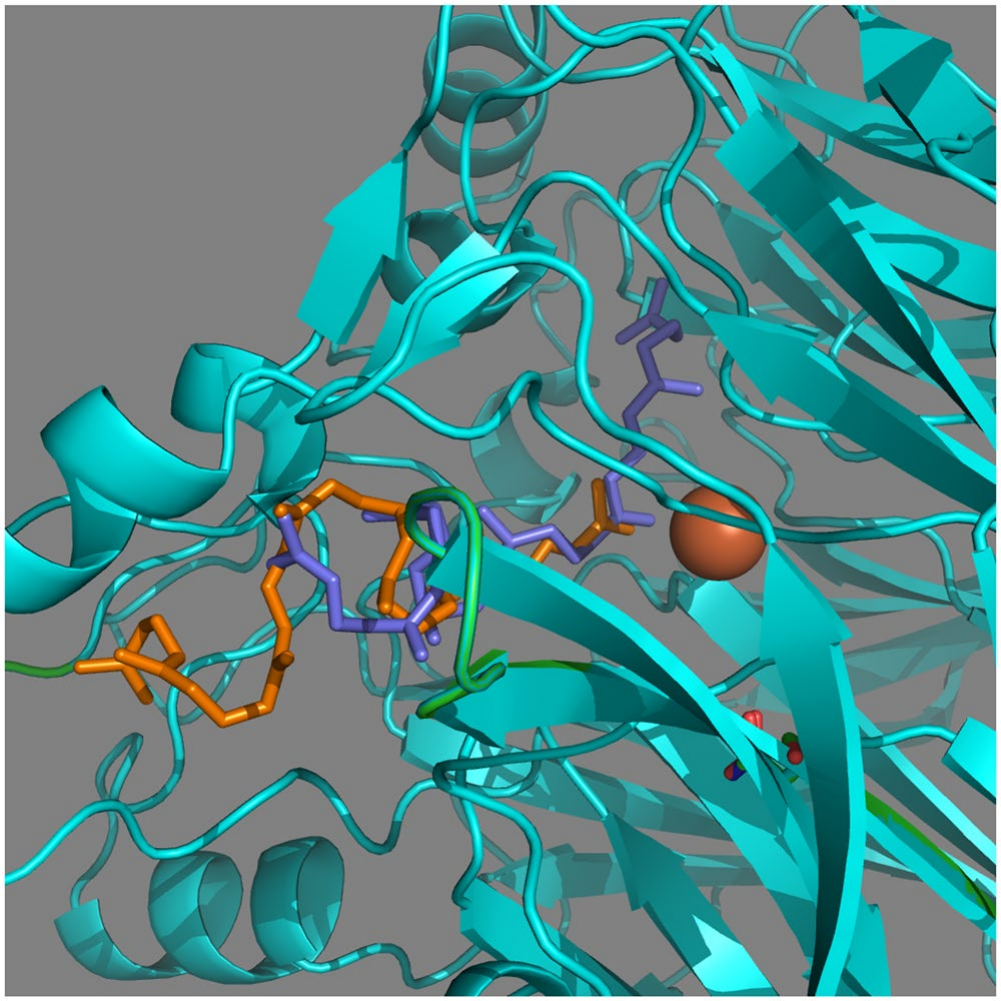
Visualization of lycopene docking on the mBCO2 protein model (lycopene in orange and protein in green) and on the mBCO2SD protein model (lycopene in purple and protein in cyan).

Second, we cloned mouse BCO2 from the Sugano mouse library (C57/BL6 Japanese strain, clone 3F7) and found that it contained 2 amino acid changes, P108S and N190D, compared to the curated wildtype mouse BCO2. This double mutant mBCO2SD has a lower activity towards β-carotene but higher activity towards lycopene compared to wildtype mouse BCO2 (Table 4). We performed modeling of substrate docking in wildtype and mBCO2SD. We used the 4F30 crystal of bovine RPE65 as template for Swiss Pro modeling. The lowest binding energy for β-carotene was not changed for mBCO2SD (−12.5 vs −12.4 kcal/mol) but the binding energy of lycopene to protein was improved for mBCO2SD (−8.4 vs −9.0 kcal/mol) and significant difference was observed in lycopene docking position in mBCO2SD relative to position of iron in the active site (Fig. 6). Thus, we found that two non-active site mutations are sufficient to significantly modulate substrate specificity of mouse BCO2 enzyme. Analysis of these positions in the multiple sequence alignment (Supplementary Data) suggested that these positions are highly variable, reflecting broad specificity and evolutionary flexibility of carotenoid cleavage oxygenases.

**Table 4:** Catalytic activity of mouse BCO2 (mBCO2) and mouse BCO2P108SN190D (mBCO2SD) proteins (% substrate left compared to uninduced control).

Sequence name	lycopene	β-carotene
	Top10 cells	Top10 cells
mBCO2	39 ± 5	31 ± 6
mBCO2SD	13 ± 3	59 ± 7

### Phylogenetic analysis of the CCO superfamily

A maximum likelihood (ML) phylogenetic tree of the oxygenase superfamily is shown in Fig. 2. The ML tree is not rooted. However, the closest relative of the animal BCO family is the archaeal CCO family with high bootstrap support (=77; Fig. 2); the subtree includes plant CCD8, fungal CCO/CarT and animal BCO/BCOL families. The phylogenetic tree suggests that the animal BCOs and BCOL families diverged after the separation of the fungal and animal clades, but before the separation of major animal clades (Fig. 2). A detailed tree of the BCOL clade is shown in Fig. 1. Lancelet BCOL proteins form a separate clade with a high bootstrap support (=75) whereas those of other deuterostomes (Mollusca, Nematoda) form another clade (Fig. 1).

We also performed phylogenetic analyses using NJ (neighbor-joining), MP (maximum parsimony) and ME (minimum evolution) methods. The topologies of ML, NJ, MP and ME trees are slightly different (Fig. 2, and Supplementary Figure S1). However these differences do not affect the results and conclusions of this study: that the BCOL family forms a well-supported clade with the archaeal CCO family as the closest prokaryotic clade (Fig. 2 and Supplementary Figure S1).

Analysis of functionally important residues using DIVERGE2 (see Materials and Methods) did not reveal any residues that were significantly functionally diverged from BCO1 or BCO2 clades (Supplementary Data). We also found the same trend for comparisons of the BCOL family vs. insect NinaB, fungal CCO, and plant CCD8 families (Supplementary Data). These results suggest that the BCOL family is not functionally different from closely related CCO clades, at least in the core catalytic region.

## Discussion

The carotenoid oxygenase superfamily includes enzymes with diverse substrate specificities in all the kingdoms of life. In vertebrates, there are three carotenoid oxygenase members, including BCO1 that cleaves β-carotene symmetrically to produce all-*trans* retinal, an entry point for vertebrate retinoid metabolism, BCO2 that asymmetrically cleaves β-carotene and several other carotenoids as a postulated scavenger, and RPE65 retinol isomerase required for production of chromophore in the visual cycle of the vertebrate retina. The specific mechanism of BCO2 activity is still widely debated, although carotenoid cleavage activity is demonstrated in carotenoid-accumulating *E. coli* and *in vitro*. However, the products of this reaction are still not very clear. The current consensus is that the BCO2 enzyme cleaves β-carotene and xanthophylls asymmetrically either only at the 9′,10′ double bond or at both the 9,10 and 9′,10′ double bond^15,30^ as well as at adjacent double bonds on the conjugated polyene chain^12,16^. Ferret BCO2 catalyzes cleavage of lycopene being mostly active towards *cis* isomers^26^.

Here we describe BCOL, a new metazoan protein family of the CCO superfamily. We originally cloned Lancelet BCOLg and found that its activity resembled that of BCO2. This protein oxidatively cleaves lycopene and β-carotene, preferring *cis* isomers of β-carotene to all-*trans* β-carotene as substrate, though our separation method could not resolve separate lycopene isomers. Following on this, we analyzed the activity of two other canonical lancelet BCO2s. We found that *cis* β-carotene isomers were the preferred substrates for one of the proteins annotated as lanBCOa. The second canonical protein lanBCOc is also active towards lycopene and β-carotene but, in contrast, does not prefer *cis* isomers of β-carotene. The activity of the BCOLg is thus not catalytically significantly different from the BCO2 proteins, and thus the absence/alteration of the otherwise conserved PDPC(K) motif may imply a change in the intracellular interactions and/or substrate capture by BCOL proteins as compared with the BCO1, BCO2 and RPE65 proteins. The placement of the motif on the outer surface of a loop covering the substrate cleft and the higher mobility of that loop is congruent with such a hypothesis.

We discovered that the substrate specificity of mouse BCO2 enzyme could be fine-tuned by several non-active-site amino acid changes in the enzyme. We cloned a polymorphic variant of mouse BCO2 BCO2/P108S+N190D from the Sugano cDNA library derived from the C57BL/6 mouse. Variations of substrains of C57BL/6 mice are well documented^31^; *Rpe65* itself has a hypomorphic variant specific to C57BL/6 mice^32^. We demonstrated that these two amino acid changes are enough to significantly change preferred substrate from β-carotene to lycopene. Our docking simulations are in agreement with our experimental results and the binding energy for lycopene is improved in the mouse BCO2/P108S+N190D (BCO2SD) double mutant. Lycopene is docked much deeper in the binding tunnel of BCO2SD and positioned properly relative to the position of the iron center. There are numerous examples of broad and easily changing specificity of carotenoid oxygenases. Mammalian and chicken BCO1 and BCO2 can cleave some apocarotenals, while Fusarium carotenoid cleavage enzyme (CCO) CarX can cleave β-carotene, torulene and β-Apo-8′-carotenal^12,28,29^. Interestingly, PpCCO from the marine myxobacterium *Plesiocystis pacifica* ^33^ and the cyanobacterial NCS3 Nostoc ACO, which also show torulene cleavage activity^27^ are closely related in sequence (Fig. 2). In the fungus *Neurospora*, the resveratrol oxygenase (RCO) CAO-1 is allied to the lignostilbene dioxygenase superfamily^34^, while its torulene oxygenase is allied to the CAO-2 carotenoid oxygenase family^35^. However, the protein sequences of these two functionally different enzymes are closely related. From an evolutionary point of view, Fusarium CarX (Carotenoid cleavage enzymes CCO)^29^ and plant CCD1,CCD4 and NCEDs are located on adjacent branches forming a clade (Fig. 2), and most probably have a common CCO-like ancestor. All these findings suggest that the range of enzymatic specificity of members of the carotenoid oxygenase superfamily is extremely broad and flexible (easily changing).

This newly described animal BCOL family, represented in both protostome and deuterostome lineages, with extensive expansions in some taxa (e.g., the Cephalochordate lancelets), strongly suggests that the overall BCO family evolves in the “high propensity for gene loss” fashion^36^: numerous losses and duplications of this family are likely to reflect complex regulation processes during evolution, development, and interactions of the organisms with the environment. We cannot exclude the scenario that the BCOL and BCO families diverged before the animal-fungi split with subsequent losses of BCOL proteins in fungal genomes (at least in available fungal genomes). This scenario is supported by the NJ tree (Supplementary Figure S1). However the ML, ME and MP phylogenetic trees (Fig. 2 and Supplementary Figure S1) suggest that the BCOL and BCO families diverged after the animal-fungi split.

This evident flexibility of CCO superfamily substrate specificity, discussed above, suggests that this may create a evolutionary basis for the remarkable de novo switch in enzymatic function of RPE65 retinol isomerase, a distinct outlier of the CCO superfamily in terms of function. RPE65, probably the most recently diverged clade, was present in the last common ancestor of jawed and jawless vertebrates^9,10^. This entity is not present in earlier chordate or non-chordate taxa, despite the existence of closely related sequences in taxa such as urochordates that were suggested to be orthologous to RPE65^11^. We speculate that the seemingly inherent plasticity of substrate specificity of the CCOs, along with concurrent evolution of another apparently vertebrate-specific entity, lecithin:retinol acyltransferase (LRAT^9,10^), allowed for the ancestor of RPE65 to forgo carotenoids as substrate in favor of the carotenoid derivative retinyl palmitate (or other retinyl ester), and to replace oxidative cleavage of carotenoid carbon:carbon double bonds by concerted O-alkyl cleavage of a retinyl ester and *trans* to *cis* double bond isomerization of the retinyl moiety^3,37^.

## Methods

### Cloning

A panel of BCO proteins from various species was made using pBADTOPO (ThermoFisher) vector, as previously described for pBAD/BCMO1 construct^38^ as a template. These constructs contain the respective open reading frames with C-terminal V5 epitopes and polyhistidine tags. Constructs were transformed either in TOP10 (ThermoFisher) or DE3 cells (Millipore Sigma) transformed with pAC-LYC or pAC-BETA plasmids (these plasmids are a generous gift from Dr. Francis Cunningham). Protein expression was induced with 0.02% of arabinose in both types of cells.

### *In vivo* assay of enzymatic activity of BCOs

pBAD/BCOs, expression constructs were transformed into Z-competent cells (Zymo Research) from a strain of *E. coli* transformed with the vector pAC-BETA that produces and accumulates β-carotene or pAC-LYC that produces and accumulates lycopene^39^. Overnight cultures of TOP10 *E. coli*/pAC-BETA or TOP10 *E. coli*/pAC-LYC with the different pBAD/BCO constructs were grown in LB broth supplemented with 100 µg/mL ampicillin and 30 µg/mL chloramphenicol at 30 °C. One ml of the overnight culture was used to inoculate 50 mL of LB broth supplemented with the same antibiotics. The culture was allowed to grow at 37 °C to mid-log phase (OD_600_ = ~0.6) and was then split; one half was induced with 0.02% (w/v) of arabinose while the other was not induced. Both cultures were grown for another 19 hours at 30 °C under yellow light, the optical density measured, and cells were collected by centrifugation at 5,000 g for 20 min. Pellets were frozen in dry ice and stored at −80 °C. To quantify the activity of the various constructs, carotenoids were extracted from frozen bacterial cultures. Small aliquots (4 mL) were frozen for protein determinations and western blotting. To quantify the activity of the various constructs, β-carotene was extracted from frozen bacterial cultures. The pellet was resuspended in lysis buffer (0.9 ml/20 ml of initial culture with *A*_600_ = 1.8) containing 0.1 M Tricine, 0.1 M NaCl, 4% formaldehyde, 0.5% Tween, 1% pyrogallol, and 1.18 μm α-tocopherol acetate as an internal standard. Resuspended cells were divided into three equal aliquots, and each was centrifuged at 16,060 × *g* for 1 min. Supernatants were collected, and carotenoids were extracted from each pellet with three sequential aliquots of 300 μl of acetonitrile with rigorous vortexing. Supernatants and acetonitrile extracts were combined and filtered and then 100 μl was injected onto a reverse phase HPLC column (see below). Samples were assayed in triplicate from induced and uninduced cultures and the activity expressed as the ratio (%) of β-carotene extracted from induced cells to β-carotene extracted from uninduced cells, normalized to cell density.

For lycopene analysis, a 1 mL volume of acetone + 1.8% formaldehyde/0.1% MeOH was added to the pellet from 20 mL of cell culture (OD_600_ = 1.8). Cells were homogenized in the acetone mixture and divided into three equal aliquots. Each aliquot was centrifuged for 5 min at 13,000 g and then each pellet was again extracted with 0.3 mL of acetone solution. Extracts were combined and centrifuged for 5 min at 13,000 g and then 50 µL was analyzed by reverse phase HPLC (below).

### HPLC Analysis of *β*-Carotene and lycopene

β-Carotene and all-*trans* retinal were separated on a 4.6 × 150-mm YMC carotenoid 3-μm column at a flow rate 1 ml/min with simultaneous UV detection at 451 and 383 (Agilent 1260 series HPLC). The initial conditions consisted of acetonitrile and 0.015 M ammonium acetate (60:40) and were held for 5 min, followed by a linear gradient to 50:50 acetonitrile/isopropanol over 10 min which was held for an additional 10 min, then followed by a linear gradient to 20:80 acetonitrile/isopropanol which was held for 2 more minutes, and finally the system was returned to initial conditions in 3 min^38^.

Lycopene and lycopene cleavage products were separated on the same YMC carotenoid column with at a flow rate of 1 ml/min with simultaneous UV detection at 455 and 471 nm. We followed a previously described method with small modifications^26^. In brief, the initial conditions consisted of methanol: methyl tert-butyl ether (MTBE): 1% Ammonium acetate 83:15:2 (mobile phase B) and were held for 3 min, followed by a linear gradient to 70:30 B:C (mobile phase C = methanol: MTBE: 1% Ammonium acetate 8:90:2) over 7 min which was held for an additional 5 min, followed by a linear gradient to 45:55 B:C in 9 min which was held for 2 more minutes, then followed by linear gradient to 5:95 B:C in 7 min which was held for 6 more minutes, and finally the system was returned to initial conditions in 1 min.

### Datasets, phylogenetic analysis and modeling

Protein sequences were downloaded from the NCBI and ENSEMBL web sites. Similarity searches were performed using the non-redundant protein sequence database at the NCBI and the gapped BLASTP program. Multiple protein sequence alignments were constructed using the Muscle program and then adjusted by hand (Supplementary Data, details available upon request from Eugenia Poliakov, Poliakove@nei.nih.gov). Phylogenetic trees based on multiple alignments of protein sequences were constructed using the maximum-likelihood method as implemented in MEGA^40^, FASTTREE^41^ and PAUP* programs^42,43^. The “Find Best Model (ML)” function of the MEGA package was used to determine the appropriate substitution models for each dataset. The model with the lowest Bayesian Information Criterion (BIC) score was considered to best describe the substitution pattern for that dataset and was subsequently chosen for phylogenetic analysis. A statistical method for estimating type-II (cluster-specific) functional divergence of protein sequences implemented in the DIVERGE2 program^44^ was used for analysis of functionally important residues (we used vertebrate BCO1 and BCO2 clades for analysis, in addition to insect NinaB, fungal CCO, and plant CCD8 families) (Supplementary Data). DIVERGE2 is designed to detect functional divergence between member genes of a protein family based on (site-specific) shifted evolutionary rates after gene speciation or duplication. Posterior analysis results in a site-specific profile for predicting amino acid residues that are responsible for functional divergence^44^.

### Data availability

All data generated or analysed during this study are included in this published article (and its Supplementary Information files).

## Electronic supplementary material

Supplemental Materials
