# Supplementary material for: Phylogenetic analysis of the metazoan carotenoid oxygenase superfamily: a new ancestral gene assemblage of BCO-like (BCOL) proteins
Source: Sci Rep. 2017 Oct 16;7:13192. doi: 10.1038/s41598-017-13521-x (PMC5643517; doi:10.1038/s41598-017-13521-x)
Supplement: Supplementary file 1 — Supplemental Materials [file 41598_2017_13521_MOESM1_ESM.pdf]

Supplementary materials and data for:

Phylogenetic analysis of the metazoan carotenoid oxygenase superfamily: a new ancestral gene assemblage of BCO-like (BCOL) proteins.

Eugenia Poliakov[1*], Joseph Soucy[1], Susan Gentleman[1], Igor B. Rogozin[2] and T. Michael Redmond[1*].

[1]Laboratory of Retinal Cell & Molecular Biology, National Eye Institute,

[2] National Center for Biotechnology Information, National Library of Medicine,

NIH, Bethesda, MD 20894, USA

[*] Corresponding author

E-mail: EP:poliakove@nei.nih.gov; TMR:redmondd@helix.nih.gov

Supplementary Table S1. Mitochondrial localization prediction with MitoProt II.

Sequence name	Protein length (aa)	Probability of export to mitochondria
Ciona RPE65like BCOa	553	0.9729
Ciona BCOb	524	0.01
Petromyzon BCO2a	583	0.8078
Mouse BCO2	532	0.0061
Human BCO2b	545	0.2545
Human BCO2a	579	0.9932
lanBCOc (XP_002605888.1)	524	0.9908
lanBCOa (XP_002598485.1)	501	0.0767
BCOLg (XP_002601288.1)	506	0.0691

Supplementary Figure S1. The phylogenetic tree of carotenoid oxygenase superfamily.

(A) The phylogenetic tree of carotenoid oxygenase superfamily (see below) was inferred by using the Maximum Likelihood method based on the WAG matrix-based model. The tree with the highest log likelihood is shown. The percentage of trees in which the associated taxa clustered together is shown next to the branches. Initial tree(s) for the heuristic search were obtained automatically by applying Neighbor-Join and BioNJ algorithms to a matrix of pairwise distances estimated using the WAG model, and then selecting the topology with superior log likelihood value. A discrete Gamma distribution was used to model evolutionary rate differences among sites (2 categories). The rate variation model allowed for some sites to be evolutionarily invariable.

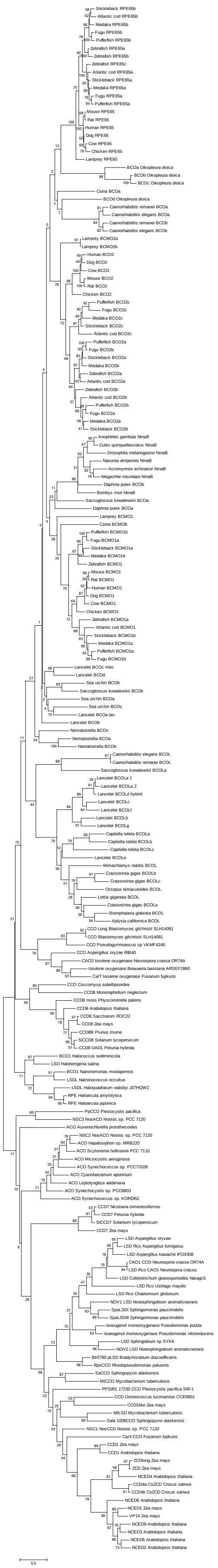
Stickleback RPE65b
Atlantic cod RPE65b
Medaka RPE65b
Fugu RPE65b
Pufferfish RPE65b
Zebrafish RPE65a
Zebrafish RPE65b
Zebrafish RPE65c
Atlantic cod RPE65a
Stickleback RPE65a
Medaka RPE65a
Fugu RPE65a
Pufferfish RPE65a
Mouse RPE65
Rat RPE65
Human RPE65
Dog RPE65
Cow RPE65
Chicken RPE65
Lamprey RPE65
BCOa Oikopleura dioica
BCOb Oikopleura dioica
BCOc Oikopleura dioica
Ciona BCOa
BCOd Oikopleura dioica
Caenorhabditis remanei BCOa
Caenorhabditis elegans BCOa
Caenorhabditis remanei BCOb
Caenorhabditis elegans BCOb
Lamprey BCMO2a
Lamprey BCMO2b
Human BCO2
Dog BCO2
Cow BCO2
Mouse BCO2
Rat BCO2
Chicken BCO2
Pufferfish BCO2c
Fugu BCO2c
Medaka BCO2c
Stickleback BCO2c
Atlantic cod BCO2c
Pufferfish BCO2a
Fugu BCO2b
Stickleback BCO2a
Medaka BCO2b
Zebrafish BCO2a
Atlantic cod BCO2a
Zebrafish BCO2b
Atlantic cod BCO2b
Pufferfish BCO2b
Fugu BCO2a
Medaka BCO2a
Stickleback BCO2b
Anopheles gambiae NinaB
Culex quinquefasciatus NinaB
Drosophila melanogaster NinaB
Nasonia vitripennis NinaB
Acromyrmex echinatior NinaB
Megachile rotundata NinaB
Daphnia pulex BCOb
Bombyx mori NinaB
Saccoglossus kowalevskii BCOa
Daphnia pulex BCOa
Lamprey BCMO1
Ciona BCMOb
Pufferfish BCMO1b
Fugu BCMO1a
Stickleback BCMO1a
Medaka BCMO1b
Zebrafish BCMO1
Mouse BCMO1
Rat BCMO1
Human BCMO1
Dog BCMO1
Cow BCMO1
Chicken BCMO1
Zebrafish BCMO1a
Atlantic cod BCMO1
Stickleback BCMO1b
Medaka BCMO1a
Pufferfish BCMO1a
Fugu BCMO1b
Lancelet BCOc mito
Lancelet BCOd
Sea urchin BCOb
Saccoglossus kowalevskii BCOb
Sea urchin BCOa
Sea urchin BCOc
Lancelet BCOa lan
Lancelet BCOb
Nematostella BCOc
Nematostella BCOa
Nematostella BCOb
Caenorhabditis elegans BCOL
Caenorhabditis remanei BCOL
Saccoglossus kowalevskii BCOLa
Lancelet BCOLa 1
Lancelet BCOLa 2
Lancelet BCOLd hybrid
Lancelet BCOLc
Lancelet BCOLf
Lancelet BCOLb
Lancelet BCOLg
Capitella teleta BCOLa
Capitella teleta BCOLb
Capitella teleta BCOLc
Lancelet BCOLe
Mimachlamys nobilis BCOL
Crassostrea gigas BCOLb
Crassostrea gigas BCOLc
Octopus bimaculoides BCOL
Lottia gigantea BCOL
Crassostrea gigas BCOLa
Biomphalaria glabrata BCOL
Aplysia californica BCOL
CCO Long Blastomyces gilchristii SLH14081
CCO Blastomyces gilchristii SLH14081
CCO Pseudogymnoascus sp VKMF4246
CCO Aspergillus oryzae RIB40
CAO2 torulene oxygenase Neurospora crassa OR74A
torulene oxygenase Beauveria bassiana ARSEF2860
CarT torulene oxygenase Fusarium fujikuroi
CCO Coccomyxa subellipsoidea
CCD8 Monoraphidium neglectum
CCD8 moss Physcomitrella patens
CCD8 Arabidopsis thaliana
CCD8 Saccharum ROC22
CCD8 Zea mays
CCD8B Prunus mume
SiCCD8 Solanum lycopersicum
CCD8 DAD1 Petunia hybrida
BCO1 Halococcus sediminicola
LSD Haloterrigena salina
BCO1 Natronomonas moolapensis
LSDL Natronococcus occultus
LSDL Haloquadratum walsbyi J07HQW2
RPE Haloarcula amylolytica
RPE Haloarcula japonica
PpCCO Plesiocystis pacifica
NSC3 NosACO Nostoc sp. PCC 7120
ACO Auxenochlorella protothecoides
NSC2 NosACO Nostoc sp. PCC 7120
ACO Hapalosiphon sp. MRB220
ACO Scytonema hofmannii PCC 7110
ACO Microcystis aeruginosa
ACO Synechococcus sp. PCC73109
ACO Cyanobacterium aponinum
ACO Leptolyngbya valderiana
ACO Synechocystis sp. PCC6803
ACO Synechococcus sp. KORDI52
CCD7 Nicotiana tomentosiformis
CCD7 Petunia hybrida
SiCCD7 Solanium lycopersicum
CCD7 Zea mays
LSD Aspergillus oryzae
LSD Rco Aspergillus fumigatus
LSD Aspergillus kawachii IFO4308
CAO1 CCO Neurospora crassa OR74A
LSD Rco CAO1 Neurospora crassa
LSD Colletotrichum gloeosporioides Naragc5
LSD Rco Ustilago maydis
LSD Rco Chaetomium globosum
NOV1 LSD Novosphingobium aromaticivorans
SpaLSDI Sphingomonas paucimobilis
SpaLSDIII Sphingomonas paucimobilis
isoeugenol monooxygenase Pseudomonas putida
isoeugenol monooxygenase Pseudomonas nitroreducens
LSD Sphingobium sp SYK6
NOV2 LSD Novosphingobium aromaticivorans
Blr5790 pLSD Bradyrhizobium diazoefficiens
RpsCCO Rhodopseudomonas palustris
SaCCO Sphingopyxis alaskensis
MtCCO Mycobacterium tuberculosis
PPSIR1 17230 CCO Plesiocystis pacifica SIR-1
CCD Ostreococcus lucimarinus CCE9901
CCD1like Zea mays
MtLSD Mycobacterium tuberculosis
Sala 1008CCO Sphingopyxis alaskensis
NSC1 NosCCD Nostoc sp. PCC 7120
CarX CCO Fusarium fujikuroi
CCD1 Zea mays
CCD1 Arabidopsis thaliana
ZCDlong Zea mays
ZCD Zea mays
NCED4 Arabidopsis thaliana
CCD4a CsZCD Crocus sativus
CCD4b CsZCD Crocus sativus
NCED6 Arabidopsis thaliana
NCED1 Zea mays
VP14 Zea mays
NCED9 Arabidopsis thaliana
NCED3 Arabidopsis thaliana
NCED5 Arabidopsis thaliana
NCED2 Arabidopsis thaliana
0.5

(B) The phylogenetic tree of carotenoid oxygenase superfamily (see below) was inferred using the Neighbor-Joining method. The optimal tree with the sum of branch length = 85.21510162 is shown. The percentage of replicate trees in which the associated taxa clustered together in the bootstrap test (1000 replicates) are shown next to the branches. The tree is drawn to scale, with branch lengths in the same units as those of the evolutionary distances used to infer the phylogenetic tree. The evolutionary distances were computed using the JTT matrix-based method and are in the units of the number of amino acid substitutions per site. The rate variation among sites was modeled with a gamma distribution (shape parameter = 2.4). The analysis involved 195 amino acid sequences. All positions with less than 75% site coverage were eliminated. That is, fewer than 25% alignment gaps, missing data, and ambiguous bases were allowed at any position. There were a total of 470 positions in the final dataset.

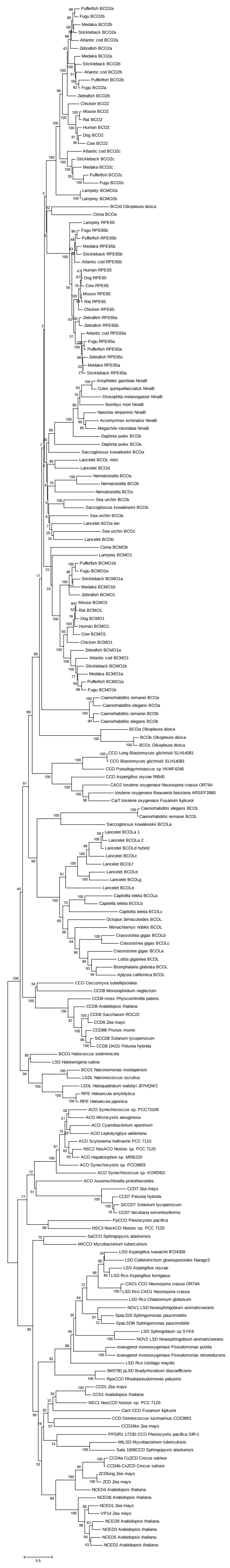
Pufferfish BCO2a
Fugu BCO2b
Medaka BCO2b
Stickleback BCO2a
Atlantic cod BCO2a
Zebrafish BCO2a
Medaka BCO2a
Stickleback BCO2b
Atlantic cod BCO2b
Pufferfish BCO2b
Fugu BCO2a
Zebrafish BCO2b
Chicken BCO2
Mouse BCO2
Rat BCO2
Human BCO2
Dog BCO2
Cow BCO2
Atlantic cod BCO2c
Stickleback BCO2c
Medaka BCO2c
Pufferfish BCO2c
Fugu BCO2c
Lamprey BCMO2a
Lamprey BCMO2b
BCOd Oikopleura dioica
Ciona BCOa
Lamprey RPE65
Fugu RPE65b
Pufferfish RPE65b
Medaka RPE65b
Stickleback RPE65b
Atlantic cod RPE65b
Human RPE65
Dog RPE65
Cow RPE65
Mouse RPE65
Rat RPE65
Chicken RPE65
Zebrafish RPE65a
Zebrafish RPE65b
Atlantic cod RPE65a
Fugu RPE65a
Pufferfish RPE65a
Zebrafish RPE65c
Medaka RPE65a
Stickleback RPE65a
Anopheles gambiae NinaB
Culex quinquefasciatus NinaB
Drosophila melanogaster NinaB
Bombyx mori NinaB
Nasonia vitripennis NinaB
Acromyrmex echinatior NinaB
Megachile rotundata NinaB
Daphnia pulex BCOb
Daphnia pulex BCOa
Saccoglossus kowalevskii BCOa
Lancelet BCOc mito
Lancelet BCOd
Nematostella BCOa
Nematostella BCOb
Nematostella BCOc
Sea urchin BCOb
Saccoglossus kowalevskii BCOb
Sea urchin BCOa
Lancelet BCOa lan
Sea urchin BCOc
Lancelet BCOb
Ciona BCMOb
Lamprey BCMO1
Pufferfish BCMO1b
Fugu BCMO1a
Stickleback BCMO1a
Medaka BCMO1b
Zebrafish BCMO1
Mouse BCMO1
Rat BCMO1
Dog BCMO1
Human BCMO1
Cow BCMO1
Chicken BCMO1
Zebrafish BCMO1a
Atlantic cod BCMO1
Stickleback BCMO1b
Medaka BCMO1a
Pufferfish BCMO1a
Fugu BCMO1b
Caenorhabditis remanei BCOa
Caenorhabditis elegans BCOa
Caenorhabditis remanei BCOb
Caenorhabditis elegans BCOb
BCOa Oikopleura dioica
BCOb Oikopleura dioica
BCOc Oikopleura dioica
CCO Long Blastomyces gilchristii SLH14081
CCO Blastomyces gilchristii SLH14081
CCO Pseudogymnoascus sp VKMF4246
CCO Aspergillus oryzae RIB40
CAO2 torulene oxygenase Neurospora crassa OR74A
torulene oxygenase Beauveria bassiana ARSEF2860
CarT torulene oxygenase Fusarium fujikuroi
Caenorhabditis elegans BCOL
Caenorhabditis remanei BCOL
Saccoglossus kowalevskii BCOLa
Lancelet BCOLa 1
Lancelet BCOLa 2
Lancelet BCOLd hybrid
Lancelet BCOLc
Lancelet BCOLf
Lancelet BCOLb
Lancelet BCOLg
Lancelet BCOLe
Capitella teleta BCOLa
Capitella teleta BCOLb
Capitella teleta BCOLc
Octopus bimaculoides BCOL
Mimachlamys nobilis BCOL
Crassostrea gigas BCOLb
Crassostrea gigas BCOLc
Crassostrea gigas BCOLa
Lottia gigantea BCOL
Biomphalaria glabrata BCOL
Aplysia californica BCOL
CCO Coccomyxa subellipsoidea
CCD8 Monoraphidium neglectum
CCD8 moss Physcomitrella patens
CCD8 Arabidopsis thaliana
CCD8 Saccharum ROC22
CCD8 Zea mays
CCD8B Prunus mume
SiCCD8 Solanum lycopersicum
CCD8 DAD1 Petunia hybrida
BCO1 Halococcus sediminicola
LSD Haloterrigena salina
BCO1 Natronomonas moolapensis
LSDL Natronococcus occultus
LSDL Haloquadratum walsbyi J07HQW2
RPE Haloarcula amylolytica
RPE Haloarcula japonica
ACO Synechococcus sp. PCC73109
ACO Microcystis aeruginosa
ACO Cyanobacterium aponinum
ACO Leptolyngbya valderiana
ACO Scytonema hofmannii PCC 7110
NSC2 NosACO Nostoc sp. PCC 7120
ACO Hapalosiphon sp. MRB220
ACO Synechocystis sp. PCC6803
ACO Synechococcus sp. KORDI52
ACO Auxenochlorella protothecoides
CCD7 Zea mays
CCD7 Petunia hybrida
SiCCD7 Solanium lycopersicum
CCD7 Nicotiana tomentosiformis
PpCCO Plesiocystis pacifica
NSC3 NosACO Nostoc sp. PCC 7120
SaCCO Sphingopyxis alaskensis
MtCCO Mycobacterium tuberculosis
LSD Aspergillus kawachii IFO4308
LSD Colletotrichum gloeosporioides Naragc5
LSD Aspergillus oryzae
LSD Rco Aspergillus fumigatus
CAO1 CCO Neurospora crassa OR74A
LSD Rco CAO1 Neurospora crassa
LSD Rco Chaetomium globosum
NOV1 LSD Novosphingobium aromaticivorans
SpaLSDI Sphingomonas paucimobilis
SpaLSDIII Sphingomonas paucimobilis
LSD Sphingobium sp SYK6
NOV2 LSD Novosphingobium aromaticivorans
isoeugenol monooxygenase Pseudomonas putida
isoeugenol monooxygenase Pseudomonas nitroreducens
LSD Rco Ustilago maydis
Blr5790 pLSD Bradyrhizobium diazoefficiens
RpsCCO Rhodopseudomonas palustris
CCD1 Zea mays
CCD1 Arabidopsis thaliana
NSC1 NosCCD Nostoc sp. PCC 7120
CarX CCO Fusarium fujikuroi
CCD Ostreococcus lucimarinus CCE9901
CCD1like Zea mays
PPSIR1 17230 CCO Plesiocystis pacifica SIR-1
MtLSD Mycobacterium tuberculosis
Sala 1008CCO Sphingopyxis alaskensis
CCD4a CsZCD Crocus sativus
CCD4b CsZCD Crocus sativus
ZCDlong Zea mays
ZCD Zea mays
NCED4 Arabidopsis thaliana
NCED6 Arabidopsis thaliana
NCED1 Zea mays
VP14 Zea mays
NCED9 Arabidopsis thaliana
NCED3 Arabidopsis thaliana
NCED5 Arabidopsis thaliana
NCED2 Arabidopsis thaliana
0.5

(C) The phylogenetic tree of carotenoid oxygenase superfamily (see below) was inferred using the Minimum Evolution method. The optimal tree with the sum of branch length = 85.21510162 is shown. The percentage of replicate trees in which the associated taxa clustered together in the bootstrap test (1000 replicates) are shown next to the branches. The tree is drawn to scale, with branch lengths in the same units as those of the evolutionary distances used to infer the phylogenetic tree. The evolutionary distances were computed using the JTT matrix-based method and are in the units of the number of amino acid substitutions per site. The rate variation among sites was modeled with a gamma distribution (shape parameter = 2.4). The ME tree was searched using the Close-Neighbor-Interchange (CNI) algorithm at a search level of 1. The Neighbor-joining algorithm was used to generate the initial tree. The analysis involved 195 amino acid sequences. All positions with less than 75% site coverage were eliminated. That is, fewer than 25% alignment gaps, missing data, and ambiguous bases were allowed at any position. There were a total of 470 positions in the final dataset.

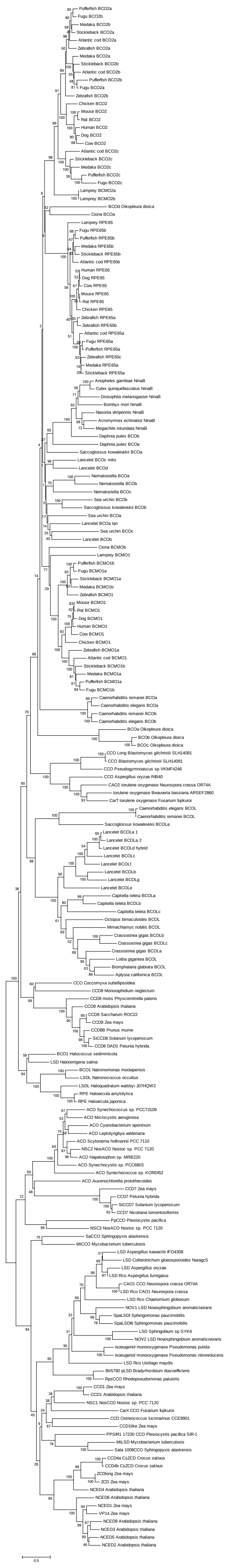
Pufferfish BCO2a
Fugu BCO2b
Medaka BCO2b
Stickleback BCO2a
Atlantic cod BCO2a
Zebrafish BCO2a
Medaka BCO2a
Stickleback BCO2b
Atlantic cod BCO2b
Pufferfish BCO2b
Fugu BCO2a
Zebrafish BCO2b
Chicken BCO2
Mouse BCO2
Rat BCO2
Human BCO2
Dog BCO2
Cow BCO2
Atlantic cod BCO2c
Stickleback BCO2c
Medaka BCO2c
Pufferfish BCO2c
Fugu BCO2c
Lamprey BCMO2a
Lamprey BCMO2b
BCOd Oikopleura dioica
Ciona BCOa
Lamprey RPE65
Fugu RPE65b
Pufferfish RPE65b
Medaka RPE65b
Stickleback RPE65b
Atlantic cod RPE65b
Human RPE65
Dog RPE65
Cow RPE65
Mouse RPE65
Rat RPE65
Chicken RPE65
Zebrafish RPE65a
Zebrafish RPE65b
Atlantic cod RPE65a
Fugu RPE65a
Pufferfish RPE65a
Zebrafish RPE65c
Medaka RPE65a
Stickleback RPE65a
Anopheles gambiae NinaB
Culex quinquefasciatus NinaB
Drosophila melanogaster NinaB
Bombyx mori NinaB
Nasonia vitripennis NinaB
Acromyrmex echinatior NinaB
Megachile rotundata NinaB
Daphnia pulex BCOb
Daphnia pulex BCOa
Saccoglossus kowalevskii BCOa
Lancelet BCOc mito
Lancelet BCOd
Nematostella BCOa
Nematostella BCOb
Nematostella BCOc
Sea urchin BCOb
Saccoglossus kowalevskii BCOb
Sea urchin BCOa
Lancelet BCOa lan
Sea urchin BCOc
Lancelet BCOb
Ciona BCMOb
Lamprey BCMO1
Pufferfish BCMO1b
Fugu BCMO1a
Stickleback BCMO1a
Medaka BCMO1b
Zebrafish BCMO1
Mouse BCMO1
Rat BCMO1
Dog BCMO1
Human BCMO1
Cow BCMO1
Chicken BCMO1
Zebrafish BCMO1a
Atlantic cod BCMO1
Stickleback BCMO1b
Medaka BCMO1a
Pufferfish BCMO1a
Fugu BCMO1b
Caenorhabditis remanei BCOa
Caenorhabditis elegans BCOa
Caenorhabditis remanei BCOb
Caenorhabditis elegans BCOb
BCOa Oikopleura dioica
BCOb Oikopleura dioica
BCOc Oikopleura dioica
CCO Long Blastomyces gilchristii SLH14081
CCO Blastomyces gilchristii SLH14081
CCO Pseudogymnoascus sp VKMF4246
CCO Aspergillus oryzae RIB40
CAO2 torulene oxygenase Neurospora crassa OR74A
torulene oxygenase Beauveria bassiana ARSEF2860
CarT torulene oxygenase Fusarium fujikuroi
Caenorhabditis elegans BCOL
Caenorhabditis remanei BCOL
Saccoglossus kowalevskii BCOLa
Lancelet BCOLa 1
Lancelet BCOLa 2
Lancelet BCOLd hybrid
Lancelet BCOLc
Lancelet BCOLf
Lancelet BCOLb
Lancelet BCOLg
Lancelet BCOLe
Capitella teleta BCOLa
Capitella teleta BCOLb
Capitella teleta BCOLc
Octopus bimaculoides BCOL
Mimachlamys nobilis BCOL
Crassostrea gigas BCOLb
Crassostrea gigas BCOLc
Crassostrea gigas BCOLa
Lottia gigantea BCOL
Biomphalaria glabrata BCOL
Aplysia californica BCOL
CCO Coccomyxa subellipsoidea
CCD8 Monoraphidium neglectum
CCD8 moss Physcomitrella patens
CCD8 Arabidopsis thaliana
CCD8 Saccharum ROC22
CCD8 Zea mays
CCD8B Prunus mume
SiCCD8 Solanum lycopersicum
CCD8 DAD1 Petunia hybrida
BCO1 Halococcus sediminicola
LSD Haloterrigena salina
BCO1 Natronomonas moolapensis
LSDL Natronococcus occultus
LSDL Haloquadratum walsbyi J07HQW2
RPE Haloarcula amylolytica
RPE Haloarcula japonica
ACO Synechococcus sp. PCC73109
ACO Microcystis aeruginosa
ACO Cyanobacterium aponinum
ACO Leptolyngbya valderiana
ACO Scytonema hofmannii PCC 7110
NSC2 NosACO Nostoc sp. PCC 7120
ACO Hapalosiphon sp. MRB220
ACO Synechocystis sp. PCC6803
ACO Synechococcus sp. KORDI52
ACO Auxenochlorella protothecoides
CCD7 Zea mays
CCD7 Petunia hybrida
SiCCD7 Solanium lycopersicum
CCD7 Nicotiana tomentosiformis
PpCCO Plesiocystis pacifica
NSC3 NosACO Nostoc sp. PCC 7120
SaCCO Sphingopyxis alaskensis
MtCCO Mycobacterium tuberculosis
LSD Aspergillus kawachii IFO4308
LSD Colletotrichum gloeosporioides Naragc5
LSD Aspergillus oryzae
LSD Rco Aspergillus fumigatus
CAO1 CCO Neurospora crassa OR74A
LSD Rco CAO1 Neurospora crassa
LSD Rco Chaetomium globosum
NOV1 LSD Novosphingobium aromaticivorans
SpaLSDI Sphingomonas paucimobilis
SpaLSDIII Sphingomonas paucimobilis
LSD Sphingobium sp SYK6
NOV2 LSD Novosphingobium aromaticivorans
isoeugenol monooxygenase Pseudomonas putida
isoeugenol monooxygenase Pseudomonas nitroreducens
LSD Rco Ustilago maydis
Blr5790 pLSD Bradyrhizobium diazoefficiens
RpsCCO Rhodopseudomonas palustris
CCD1 Zea mays
CCD1 Arabidopsis thaliana
NSC1 NosCCD Nostoc sp. PCC 7120
CarX CCO Fusarium fujikuroi
CCD Ostreococcus lucimarinus CCE9901
CCD1like Zea mays
PPSIR1 17230 CCO Plesiocystis pacifica SIR-1
MtLSD Mycobacterium tuberculosis
Sala 1008CCO Sphingopyxis alaskensis
CCD4a CsZCD Crocus sativus
CCD4b CsZCD Crocus sativus
ZCDlong Zea mays
ZCD Zea mays
NCED4 Arabidopsis thaliana
NCED6 Arabidopsis thaliana
NCED1 Zea mays
VP14 Zea mays
NCED9 Arabidopsis thaliana
NCED3 Arabidopsis thaliana
NCED5 Arabidopsis thaliana
NCED2 Arabidopsis thaliana
0.5

(D) The phylogenetic tree of carotenoid oxygenase superfamily (see below) was inferred using the Maximum Parsimony method. The most parsimonious tree with length = 24881 is shown. The consistency index is (0.235554), the retention index is (0.578292), and the composite index is 0.136432 (0.136219) for all sites and parsimony-informative sites (in parentheses). The percentage of replicate trees in which the associated taxa clustered together in the bootstrap test (1000 replicates) are shown next to the branches. The MP tree was obtained using the Subtree-Pruning-Regrafting (SPR) algorithm with search level 1 in which the initial trees were obtained by the random addition of sequences (10 replicates). The analysis involved 195 amino acid sequences. All positions with less than 75% site coverage were eliminated. That is, fewer than 25% alignment gaps, missing data, and ambiguous bases were allowed at any position. There were a total of 470 positions in the final dataset.

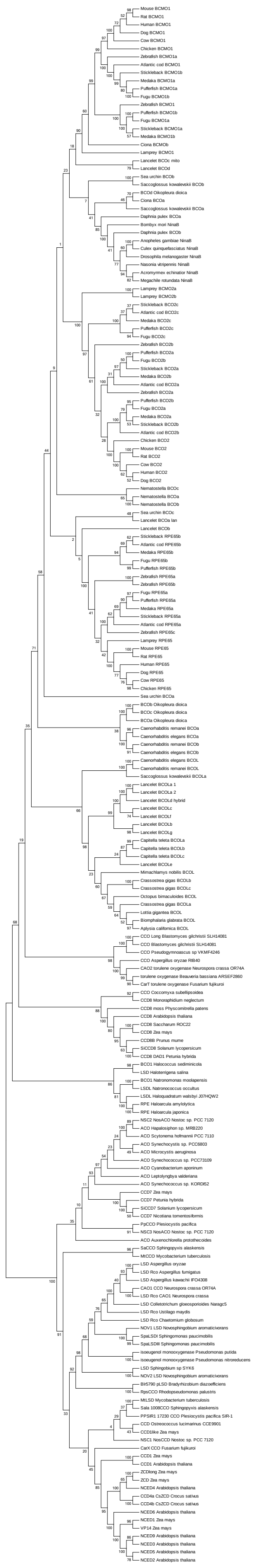
Mouse BCMO1
Rat BCMO1
Human BCMO1
Dog BCMO1
Cow BCMO1
Chicken BCMO1
Zebrafish BCMO1a
Atlantic cod BCMO1
Stickleback BCMO1b
Medaka BCMO1a
Pufferfish BCMO1a
Fugu BCMO1b
Zebrafish BCMO1
Pufferfish BCMO1b
Fugu BCMO1a
Stickleback BCMO1a
Medaka BCMO1b
Ciona BCMOb
Lamprey BCMO1
Lancelet BCOc mito
Lancelet BCOd
Sea urchin BCOb
Saccoglossus kowalevskii BCOb
BCOd Oikopleura dioica
Ciona BCOa
Saccoglossus kowalevskii BCOa
Daphnia pulex BCOa
Bombyx mori NinaB
Daphnia pulex BCOb
Anopheles gambiae NinaB
Culex quinquefasciatus NinaB
Drosophila melanogaster NinaB
Nasonia vitripennis NinaB
Acromyrmex echinatior NinaB
Megachile rotundata NinaB
Lamprey BCMO2a
Lamprey BCMO2b
Stickleback BCO2c
Atlantic cod BCO2c
Medaka BCO2c
Pufferfish BCO2c
Fugu BCO2c
Zebrafish BCO2b
Pufferfish BCO2a
Fugu BCO2b
Stickleback BCO2a
Medaka BCO2b
Atlantic cod BCO2a
Zebrafish BCO2a
Pufferfish BCO2b
Fugu BCO2a
Medaka BCO2a
Stickleback BCO2b
Atlantic cod BCO2b
Chicken BCO2
Mouse BCO2
Rat BCO2
Cow BCO2
Human BCO2
Dog BCO2
Nematostella BCOc
Nematostella BCOa
Nematostella BCOb
Sea urchin BCOc
Lancelet BCOa lan
Lancelet BCOb
Stickleback RPE65b
Atlantic cod RPE65b
Medaka RPE65b
Fugu RPE65b
Pufferfish RPE65b
Zebrafish RPE65a
Zebrafish RPE65b
Fugu RPE65a
Pufferfish RPE65a
Medaka RPE65a
Stickleback RPE65a
Atlantic cod RPE65a
Zebrafish RPE65c
Lamprey RPE65
Mouse RPE65
Rat RPE65
Human RPE65
Dog RPE65
Cow RPE65
Chicken RPE65
Sea urchin BCOa
BCOb Oikopleura dioica
BCOc Oikopleura dioica
BCOa Oikopleura dioica
Caenorhabditis remanei BCOa
Caenorhabditis elegans BCOa
Caenorhabditis remanei BCOb
Caenorhabditis elegans BCOb
Caenorhabditis elegans BCOL
Caenorhabditis remanei BCOL
Saccoglossus kowalevskii BCOLa
Lancelet BCOLa 1
Lancelet BCOLa 2
Lancelet BCOLd hybrid
Lancelet BCOLc
Lancelet BCOLf
Lancelet BCOLb
Lancelet BCOLg
Capitella teleta BCOLa
Capitella teleta BCOLb
Capitella teleta BCOLc
Lancelet BCOLe
Mimachlamys nobilis BCOL
Crassostrea gigas BCOLb
Crassostrea gigas BCOLc
Octopus bimaculoides BCOL
Crassostrea gigas BCOLa
Lottia gigantea BCOL
Biomphalaria glabrata BCOL
Aplysia californica BCOL
CCO Long Blastomyces gilchristii SLH14081
CCO Blastomyces gilchristii SLH14081
CCO Pseudogymnoascus sp VKMF4246
CCO Aspergillus oryzae RIB40
CAO2 torulene oxygenase Neurospora crassa OR74A
torulene oxygenase Beauveria bassiana ARSEF2860
CarT torulene oxygenase Fusarium fujikuroi
CCO Coccomyxa subellipsoidea
CCD8 Monoraphidium neglectum
CCD8 moss Physcomitrella patens
CCD8 Arabidopsis thaliana
CCD8 Saccharum ROC22
CCD8 Zea mays
CCD8b Prunus mume
SlCCD8 Solanum lycopersicum
CCD8 DAD1 Petunia hybrida
BCO1 Halococcus sediminicola
LSD Haloterrigena salina
BCO1 Natronomonas moolapensis
LSDL Natronococcus occultus
LSDL Haloquadratum walsbyi J07HQW2
RPE Haloarcula amylolytica
RPE Haloarcula japonica
NSC2 NosACO Nostoc sp. PCC 7120
ACO Hapalosiphon sp. MRB220
ACO Scytonema hofmannii PCC 7110
ACO Synechocystis sp. PCC6803
ACO Microcystis aeruginosa
ACO Synechococcus sp. PCC73109
ACO Cyanobacterium aponinum
ACO Leptolyngbya valderiana
ACO Synechococcus sp. KORDI52
CCD7 Zea mays
CCD7 Petunia hybrida
SlCCD7 Solanum lycopersicum
CCD7 Nicotiana tomentosiformis
PpCCO Plesiocystis pacifica
NSC3 NosACO Nostoc sp. PCC 7120
ACO Auxenochlorella protothecoides
SaCCO Sphingopyxis alaskensis
MtCCO Mycobacterium tuberculosis
LSD Aspergillus oryzae
LSD Rco Aspergillus fumigatus
LSD Aspergillus kawachii IFO4308
CAO1 CCO Neurospora crassa OR74A
LSD Rco CAO1 Neurospora crassa
LSD Colletotrichum gloeosporioides Nara gc5
LSD Rco Ustilago maydis
LSD Rco Chaetomium globosum
NOV1 LSD Novosphingobium aromaticivorans
SpaLSDI Sphingomonas paucimobilis
SpaLSDII Sphingomonas paucimobilis
isoeugenol monooxygenase Pseudomonas putida
isoeugenol monooxygenase Pseudomonas nitroreducens
LSD Sphingobium sp SYK6
NOV2 LSD Novosphingobium aromaticivorans
Bll5790 pLSD Bradyrhizobium diazoefficiens
RpsCCO Rhodopseudomonas palustris
MtLSD Mycobacterium tuberculosis
Sala 1008CCO Sphingopyxis alaskensis
PPSIR1 17230 CCO Plesiocystis pacifica SIR-1
CCD Ostreococcus lucimarinus CCE9901
CCD1like Zea mays
NSC1 NosCCD Nostoc sp. PCC 7120
CarX CCO Fusarium fujikuroi
CCD1 Zea mays
CCD1 Arabidopsis thaliana
ZCDlong Zea mays
ZCD Zea mays
NCED4 Arabidopsis thaliana
CCD4a CsZCD Crocus sativus
CCD4b CsZCD Crocus sativus
NCED6 Arabidopsis thaliana
NCED1 Zea mays
VP14 Zea mays
NCED9 Arabidopsis thaliana
NCED3 Arabidopsis thaliana
NCED5 Arabidopsis thaliana
NCED2 Arabidopsis thaliana

Supplementary Figure S2. Partial alignment of N-terminal of BCO2 proteins. PDPCK motif is shown in green. Putative mitochondria targeting signal is shown in red.

```
cionaBCOa(ciRPE65)              -----------------------------MFAIQRRPFFAVFRNFNKMSAPSKTKSYVK

NematostellaBCOa                ---------------------------------------------------MASSKYTG

XP_002598485.1lanBCO2a          ------------------------------------------------------MGVEQ

XP_002605888.1mitolanBCO2c      ---------MLKLLVSQRSSGRLLSAPVGRRIISLSRLNPGVSVS--LFRTSSQHFGMDA

lampreyBCO2a                    MGSNGSVVRLGGLVLGLLWIFLQDFASWLSRLLGISLLFPRRRSASRVYAKCSGLQCIAP

humanBCO2a                      ----MFFRVFLHFIRSHSATAVDFLPVMVHRLPVFKRYMGNTPQKKAVFGQCRGLPCVAP

humanBCO2b                      -------------------------------------MGNTPQKKAVFGQCRGLPCVAP

mouseBCO2                       ----------------------------------------------MLGPKQSLPCIAP



cionaBCOa(ciRPE65)              LLQKAEERANA-ECVVTGCIPEWLNGDVLRNGPAEFDIGPDTFKHWFDGHALLHKFSMFE

NematostellaBCOa                IVESCIELDRPIQAKVIGQIPPWLNGTLLRNGPGKFEFGDTSYNHWFDGQSLLHRFTIHN

XP_002598485.1lanBCO2           IYKTYTEHPDPVKAVIKGNFPKWLSGGLVRNGPGMFDIGEDKISHWFDGLGLMHRFNFKD

XP_002605888.1mitolanBCO2       LFKSVEETSQPIQADVKGQIPDWLKGSLLRVGPGKFEIGDQSYNHLFDGLSLIHRFNIEG

lampreyBCO2a                    IIATMTETPETMEAEIYGTIPAWIKGTLLRIGPGKFEFGPDSYNHWFDGMALMHRFRVHD

humanBCO2a                      LLTTVEEAPRGISARVWGHFPKWLNGSLLRIGPGKFEFGKDKYNHWFDGMALLHQFRMAK

humanBCO2b                      LLTTVEEAPRGISARVWGHFPKWLNGSLLRIGPGKFEFGKDKYNHWFDGMALLHQFRMAK

mouseBCO2                       LLTTAEETLSAVSARVRGHIPEWLNGYLLRVGPGKFEFGKDRYNHWFDGMALLHQFRMER

                                :  .  *     .. : * :* *:.* ::* **. *::*    .* *** .*:*.* .


cionaBCOa(ciRPE65)              GKVTYSSKFLRSGTYKTNHENSRIIIGEFGTASRPDPCKNMFSRFFTNFVEI---APRSD

NematostellaBCOa                GEVEYFNRFLRSKAYVENTKANRITRSEFGTNAPDPCKNIFDRYFSYYFGG---DDITD

XP_002598485.1lanBCO2           GEVTYRSKYLRGETYERCMKENRIAFTGFGSVHYPDPCKNIFRRFLSYFRME----EPTD

XP_002605888.1mitolanBCO2       SKVTYQNRFLRSDSYVLAQQQNRIVLTEFGTNSYPDPCKNIFSRIFSYFWDT---SGSSD

lampreyBCO2a                    GRVSYSSRFLRSDSYLRNEARQRIVVSEFGTMAVPDPCKTLFDRFFTYFSPP----APTD

humanBCO2a                      GTVTYRSKFLQSDTYKANSAKNRIVISEFGTLAPDPCKNVFERFMSRFELPGKAAAMTD

humanBCO2b                      GTVTYRSKFLQSDTYKANSAKNRIVISEFGTLALPDPCKNVFERFMSRFELPGKAAAMTD

mouseBCO2                       GTVTYKSKFLQSDTYKANSAGGRIVISEFGTLAPDPCKSIFERFMSRFEPP----TMTD

                                . * * ..:*.. :*       **    **:   *****.:* * :: :         :*
```

Supplementary Figure S3. The short conserved motif PDPC(K) which is located in the center of a large loop spanning beta sheet 1 and 2 over the substrate cleft. In all crystal structures of the CCO superfamily, this loop tends to be mobile (higher b values), and all the comparable sequences to the PDPC(K) motif are on the outer surface of the proteins.

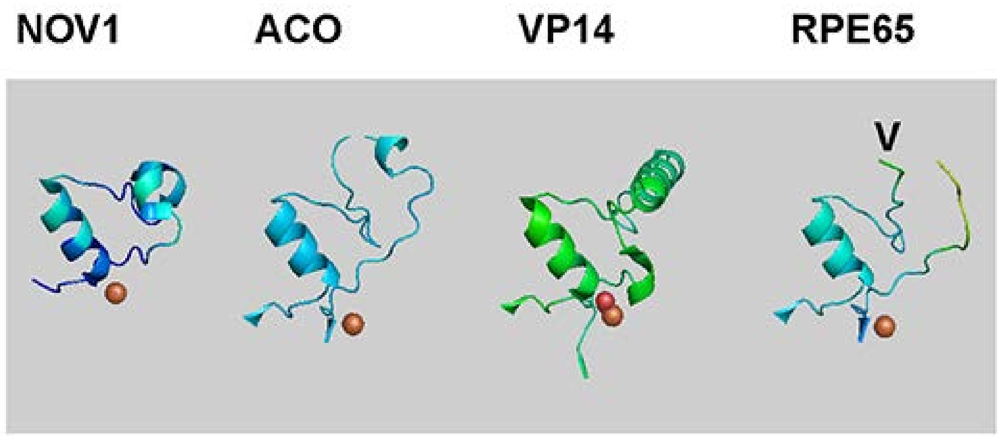

Supplementary Data.

The CCO multiple sequence alignment and results of DIVERGE3

```
Multiple sequence alignment (X1=P108S, X2=N190D, see the Results section)

>LSDL_Haloquadratum_walsbyi_J07HQW2
X1
X2
----------------------------------------------------------------------------
----------------------------------------------------------------------------
---------------------------------------------------------MSTHPGF------
HSLTTET--DTS------------------ISV------------------EGS--LPSWL-TGTLIRNGPG----
-AF------SF-----SE--GSSVDHWFDGLAMLTRFTF---DPTKGADDI------V------HYQNRFLRT---
---DAYEAAREG-----EFTGGFA-------TGETTLR------------SRLAGFLTA-----------------
---TYDNTNIIVERVD--------DT----------------------FLALT-ESP--RSVSV----
NPTTLETS--GQ-------I----------E-------------------YD--GTAPT-GQLR----------
CAHFRRDPA------TGTLLT------------V--DTSFGRR-------------SEYHIYTI------------
-------------------------DST---------DS----------HTHIGSVPTE----KPA-------
YMHSFG-LTPRYVILTEFPL--RLDPLQF----LR--PGR-QDP-FIEQF-------------------------
EWEPDRGTR-IVVVDRM-------TG-------DIV--ADPV--------------V-----D--
PVFGFHHVNAF-------ERDGGRKVVFDLETVP--DDTS-----------------IDS--LYLDTL--------
---------------REGKLGTLAG----------------------RLERFVVDL--------------------
-------------------------------------GSPAGDRYGSTTA--------------------------
-----TVSQ-------------------------------------------------------------------
----------------------------------------------------------------------------
----------------------------------------------------------------------------
----------------------------------------------------------------------------
----------------------------------------------------------------------------
----------------------------------------------------------------------------
-----------------------------QMLYDDGT----------------ALPTTS-
PAQWCQPHRYIYAMSMEQPVTEWA---------------QAILKFDTETT--------N-----------------
---SI--------EYGQGADY--FTEPIFVPN---P-VGES---RDDG--VVLTVGLDT-------D--------
QDRSRL---FVLSGDTLNE----HARVTLP--HAI-PFDFHGRY--------------------------------
------------FPNVHVA---------------------------------------------------------
----------------------------------------------------------------------------
----------------------------------------------------------------------------
----------------------------------------------------------------------------
----------------------------------------------------------------------------
----------------------------------------------------------------------------
----------------------------------------------------------------------------
----------------------------------------------------------------------------
-------------------------------------
>BCO1_Halococcus_sediminicola
----------------------------------------------------------------------------
----------------------------------------------------------------------------
-------------------------------------------------------------MDH------
RLGFEPV-REEC------------TRKR--LPV------------------EGD--LPDWL-AGTLLRNGPG----
-RF------RV-----GN---RPLNHWFDGFALLRRFGI---R-----DGA------V------VYSNRFLRS---
---EAYEHAREHGAL--GFRE-FA----TD-PETGPLGRL----------KRLVSIAFT-----------------
-----DNACVDIEVRG--------EG----------------------FVATT-ESP--RAVSF----
DPETLATR--GH--------------------------------------HE--RERLG--TTT----------
TVHPHYDAR------RGETIG------------Y--TTRLGRK----------P---GYTLYRI------------
-------------------------PDG---------GT---------EPDVIGGVTID----EPA-------
YLHSFA-LTENHIVLTEGPF--VVSPLDL----L---GGA---S-VAESF-------------------------
```

AWKPERGTR-FIVFDRE-------SG-------DVA--ATHR--------------A-----K--
PFFVFHHVNAF----E--RNG---EIVLDLVAFP--DASV-----------------VGA--LSLDNL--------
---------------RSPSPSLPTG----------------------ALRRYHLPK--------------------
--GSTRAVER--------------------------

-----------------------------ETLYAGHV----------------
ELPTIDYARRNTRPYRYVYGVSNRKRPPAEF--------------QNRLVKIDVESH--------T----------
----------TE--------TWEEPNTY--PGEPVFVPA---L-DDAATAGEDEG--VILSVVLDA-------A--
------AERSFL---LVLDAASFTERA--RAVV--P--HPI-PFGFHGQF--------------------------
------------------YRAGERPTRSMA--

>LSD_Haloterrigena_salina

MPPEHRPADRCSNDRTTDDDDGDANRSVEQGDRESTASTALDRRDVLGTGTVAAGALLGGCI--------------
------------------------------GGSESSGTASVDERHPGFRSL-ENEV------------ADRS--
LPV------------------EGE--LPSWL-EGTLLRTGPA-----KF------EV-----GD---
RHLRHWFDGFAMLHRFAI---D-----DGA------V------TYSNRFLET------RAYEHATDEGEL--
GYRE----------FATDPCRRI---W------ERFFTLFSD-------------------TTTDNANVSVATHA-
-------DR----------------------FRAMT-ETP--IPVEF----DPETLETL--GV-------V-----
-------------------------AD--GTDLG--DLT----------TAHPHRD--------DGETIN------
------Y--VTDLSRT----------S---EYRVFRM-------------------------------------
PDG---------EH---------ERDVFATVSRD----RPA-------YMHSFG-LTDRYVILAEFPF--
VVDPLEF----LVR-----DRP-FIENY-------------------------EWVPDRGTR-FLVIERA------
-TG-------EVV--STHE--------------T-----D--AFFAFHHVNAF-------EDGD--
DIVVDVVAYD--DASI-----------------VED--LYLEEI-----------------------
RSEGYSPDGG----------------------RLDRFRL-------------------------------------
---------------------------GETVAR---

--------------EALYEGPI----------------ELPGINYDRVNAEPYRYAYGVGNRENPPRDV-------
-------PNTLVKVDVETG--------A--------------------AS--------EWEEPGTY--
AGEPVFVES---P-DATA---EDDG--VILSVVLDA-------A--------AERSFL---LVLDAGTFEELA--
RAPA--P--HHI-PLGFHGIF---ADL-------


```
----------------------------------------------------------------------------
--------------
>RPE_Haloarcula_amylolytica
----------------------------------------------------------------------------
----------------------------------------------------------------------------
-------------------------------------------------------------MTF------
HPGFHSL-HDET---------------AASISV------------------NGD--LPDWL-RGSLIRNGPG----
-AF------SL-----PN--GSSVDHWFDGFAMLYRFTF---DPDS---DA------V------HYRNRFLRT---
---DAYEAATSG-----EFEGGFA-------TGETTLR------------SRLATFLTD-----------------
---PYDNTNIIAERFG--------RE----------------------YVALT-ESP--RKVRF----
DPNTLEKT--GH-------V------------------------------EHDDGVPTG--QLS----------
CAHVKRDPT------SGVLVN------------V--DTAFGRT-------------SQYHVTAM------------
-------------------------SPD---------GS----------RRHVGSVDTD----QPA-------
YMHSFA-LTPRYVVLTEFPL--RLDPRRF----LK--PGR-QAP-FIEQF-------------------------
EWDPDRGTR-IVVMDRT-------TG-------TVV--AEPV--------------I-----D--
AVFGFHHVNAF-------ERAGGRELVFDLETVP--DATT-----------------IDS--LYLDNL-------
RA----------------GEMGAIVG----------------------RIERFTVDL-------------------
---------------------------------------------GTGSGV-------------------------
----PRYRDV------------------------------------------------------------------
----------------------------------------------------------------------------
----------------------------------------------------------------------------
----------------------------------------------------------------------------
----------------------------------------------------------------------------
----------------------------------------------------------------------------
------------------------------DATVSREM------VYPDGS----ALPTVS-
PARWCRPHRYVYAMG-MDTPVTEW--------------ARRVLKLDTDTG--------A-----------------
---VE--------TFDDGGDY--FGEPVFVPE---P-DGDA---ADDG--VVLVVALDA-------D--------
ADRSRL---LVLDGQTFEE----RARATLP--HAA-PFDFHGRY--------------------------------
------------FPELRVATGD------------------------------------------------------
----------------------------------------------------------------------------
----------------------------------------------------------------------------
----------------------------------------------------------------------------
----------------------------------------------------------------------------
----------------------------------------------------------------------------
----------------------------------------------------------------------------
----------------------------------------------------------------------------
-------------------------------------
>BCO1_Natronomonas_moolapensis
----------------------------------------------------------------------------
----------------------------------------------------------------------------
---------------------------------------------------------MTEQPAP------
GPGFRSL-ESEY------------DDYR--PTV------------------EGS--IPEWL-SGTLVRNGPG----
-RF------EA-----GD---SRVNHWFDGLAMLRRYAF---D-----DGE------V------RYSNRFLRT---
---DAYADAAEGR----LTGQ-FG----TD------TRGW----------RRFLETVAS---------------
FGLPEPTDNANVHVARID--------GE----------------------YVALT-EAP--RRVAF----
DPETLATR--GE--------------------------------------FSFEDGVTE--HLT----------
AAHLVDDPH------REEVVG------------F--ATQFGMP---------P---QYHVYRV------------
-------------------------PKG---------SR---------RRELIASIEAD----GPA-------
YIHDCS-VTADHVLLVESPL--ELSVLRA----LN--PFS-DGA--IDLL-------------------------
KWNPDRPTR-VLVVDRD-------SG-------DLL--ADPT--------------V-----E--
AAFTFHHVNAY-------LDD---GIVLDLVEFP--DADI-----------------VDS--LSLAAL--------
--------------DGVGFPDVPDG----------------------RLVRYRIDP--------------------
--------------------------------------------VTNAVD--------------------------
---R-TRLY-------------------------------------------------------------------
----------------------------------------------------------------------------
----------------------------------------------------------------------------
----------------------------------------------------------------------------
```


-----------------------------DG----GI----------------ELPRSV-RSRVGRRHRYAYGQA-
TDREGANG-----------------LVKVDCASG--------T--------------------AR--------
EWWERSVY--VEEPIPIQR---P-DGDA---EDDG--VVVATALDV-------D--------RERTAL---
LIFDAATLDVEA--RAWL--P--HAE-PFGFHGRY---
---FPDV---

>LSDL_Natronococcus_occultus

--MTTPTLGF------
HSLTTEL-RDHR------------------LPV------------------EGT--VPDWL-SGTLIRNGPG----
-RF------DV-----GN---RRVNHWFDGLAMLRRYAF---E-----DGQ------V------RYSNRFLRT---
---GAYGAAIQGQ----LSGQ-FG----TDTRGWRDLLGA----------AISPGPPEP-----------------
----TDNASVHVARID--------GE----------------------YVALT-EAP--SRVAF----
DPETLETR--GR-------F----------E-------------------FD--DDLPE--HVA----------
AAHLVDDPV------RDELVG------------F--TTQFGLQ----------P---RYHLYRL------------
-------------------------PYG---------SR---------TRERIAAVDAN----GPA-------
YVHDCS-VTATHVVIVESPL--VLSALRA----LD--PFA-DGP--IDML-------------------------
EWQPERGTR-VLVVDRD-------TG-------ELV--ADPT--------------L-----D--
PVFTFHHVNAY-------VDGE--TVVLDLVEYP--DGDI-----------------VGS--MSLSEL-------
DG-------------DDEGFPAVSDA----------------------RLVRYRIDL-------------------
---RANAVE-------------------------
----R-TPRY--

------------------------------DG----SV----------------EMPRVA-
RSVVGRHHRYAYGQA-TGRGGASG-----------------LVKVDCETG--------T-----------------
---AR--------EWWKQGCY--VEEPVPVRR---P-GGDA---EDDG--VVLATTLET-------N--------
AERTLL---LVFDAATLTL----RARVALP--HAE-PFGFHGRF--------------------------------
------------FENV--

>RPE_Haloarcula_japonica

---MSSHLGF------
HSLYDET--AAS------------------LSV------------------TGA--FPDWL-HGSLIRNGPG----
-AF------SL-----PN--GSNVDHWFDGFAMLYRFTF-------DPDSD------A-----VHYRNRFLRT---
---DAYEAATSG-----EFEGGFA-------TGETTLR------------SRLATFLTD-----------------

```
--P-YDNTNIIAERFG--------GE----------------------YVALT-ESP--RKVRF----
DPNTLETT--GA-------L----------E-------------------HD-DDVPTG--QLS----------
CAHLKRDPT------SGVLVN------------V--DTAFGRT-------------SQYHVTAI------------
-------------------------SSD---------GT----------RRHVGSVDTD----QPA-------
YMHSFA-LTPRYVVLTEFPL--RLDPRRF----LK--PGR-QAP-FIEQF-------------------------
EWEPDRGTR-IIVMDRT-------TG-------TVV--AEPV--------------I-----D--
AVFGFHHVNAF-------ERAGGRELVFDLETVP--DATT-----------------IDS--LYLDNL-------
RA----------------GEMGAIVG----------------------RIERFTVTL-------------------
---------------------------------------------GTGSDV-------------------------
----TRYGDA------------------------------------------------------------------
----------------------------------------------------------------------------
----------------------------------------------------------------------------
----------------------------------------------------------------------------
----------------------------------------------------------------------------
----------------------------------------------------------------------------
------------------------------DATVSREM------LYPDGS----ALPTVS-
PSRWCRPHRYVYAMG-MDTPVTEW--------------ARRVLKLDTDTG--------A-----------------
---VE--------TFDDGGDY--FGEPVFVPA---P-DGDT---EDDG--VVLVVALDA-------D--------
ADRSRL---LVLDGQTFKE----CARVLLP--HAA-PFDFHGRY--------------------------------
------------FPELRVAAGD------------------------------------------------------
----------------------------------------------------------------------------
----------------------------------------------------------------------------
----------------------------------------------------------------------------
----------------------------------------------------------------------------
----------------------------------------------------------------------------
----------------------------------------------------------------------------
----------------------------------------------------------------------------
-------------------------------------
>CCO_Coccomyxa_subellipsoidea
----------------------------------------------------------------------------
--------------------------------------------------------
MAQDRGASVRADAATLEAPTTSSAPSTYPT----------------------------------------------
EAARRTAF------REDFTPE-LTEP------------VE----ANI------------------QGS--VPGWL-
HGSFVRNGPG-----TF------------------SGMKHLFDGYAMLAKFEF---E-----NGS------V----
--KVSHKYVQT------EAYKAFRQTGKM--QFPE-FG----TT---VSPLKSM------------WLALLGF---
------------------TDNSSVNVVPTPD-------GK---------------------
QLISLTESVAGTFRLDM----DLNTLGQL----------------------K-------------------FE--
DGVKG--LLT----------TAHPTLLP-------DGSMIN------------L--TSGFGTH-------------
--YTVFRQ------------------------------------NRAT---------NV----------
REEIAQVPLCSPP-TPS-------WIHDFP-VTENYAIVPETPI--FFNLKAA----LFG-----GAD--YVMF--
-----------------------DWKPEQRTI-LHLVNLK-------TG-------AVT---QFD-----------
---A-----P--PYFTFHYINSF----ETADGA---SLCFDFSRFE--DPDF-----------------MNR--
LMLDNL-------RG----------------NKRPIAAS----------------------PVVRITVPL------
--------------------------------------------------------DGSSSSAK------------
-----------------L-EPLM-----------------------------------------------------
----------------------------------------------------------------------------
----------------------------------------------------------------------------
----------------------------------------------------------------------------
----------------------------------------------------------------------------
----------------------------------------------------------------------------
-------------------------------------------KDDSYNFC----------------ELPRVN-
PHYKGKEYRYAYAVS-AKLPASFG---------------NSLAKFDVEEG--------S-----------------
---VK--------QWHEAGCI--PVEPLMVPR---P-GAQD---EDDG--VVLSVVTGA----------------
DGKSFL---LVLDASSFQELA--RAHL--P--HGI-PYGFHGTF--------------------------------
------------VHAD------------------------------------------------------------
----------------------------------------------------------------------------
----------------------------------------------------------------------------
```


>CCD8_Monoraphidium_neglectum

MLLRPGTATPAAPALPATGD--
HVPTANAR------QALFGVA-KEQV--------E---EV-P--ARV------------------VGA--IPKWL-
SGSLVVNGGG-------------DY----------SQMTHLFDGYAQLVKARF---K-----DGQ------A----
--WGAQRYIDT------EAYRAFKREGKV--VYRE-FA----TPPATSSSLEALLSGL------RDVVAVVTG---
--------------ATNTTDNASVSLHSVGPKGGR---GR--------------------RQLLAVS-ETP-
KASYLI----DPDTLATI--GR------------------G-----------------------GAPAV--DLQ--
--PAGRSHPKPPGPP--------RSRNLP------------LLPQASFPDK-------------------------
--------------------------------DPET---------LK----------RTEVAFVADRRPL-TPA--
-----WLHDFP-STDDYAVIIEHPL--YINLGSL----LFG-----NPR-PYVFM---------------------
----DWQPEDGTR-ITVVRLD-------GS-------EPQ--RVFD--------------A-----P--
PFFVFHYGQCY----EATAADGSRQLVVDMAAYD--DPTI-----------------LND--LGLQPL--------
---------------KEPQRQVSQS----------------------YYKRLTIPL--------------------
--SGPATSLP--------------------------
--APTPLAA---

-----------------------------DPSANDFC----------------EFPAIN-PAFRGRPYRYAYTLS-
AVRPTNMG---------------NALSRIDVATG--------E--------------------TL--------
TWHQPGAA--IGEPLFVAA---P-EAAA---EDDG--VVLAPGAAP----------------NGGGFV---
VVLDAKTMAELG--RVEL--P--FEA-PYRFHGIW---
---LDGRE--

>ACO_Auxenochlorella_protothecoides

MVAPPTHTETDRV--DEREWAEF------
LKSFTSL-HQER--------SYWVPD----AQV------------------EGT--IPKEL-EGTLLKNGPG----
-QF------EI-----GG---QKVAHPFDGDGMVAAFAF---K-----EGR------C------FFSNRFVRT---
---KAYTEEQRAGRM--LYRG----------AFSAGNPGG----------KGFFNPFKL-----------------
--DVKGIANTGVVRWG--------GR----------------------TLALY-ERD--MPYEL----
ESPDLQTL--GT-------T----------D-------------------AD--GALKD--TDP--------
YFGAHYRITTD-----ADGKRTL------------I--GFSSSEV--------GSQ--NRLTVWEF----------
---------------------------DED---------------------FNRLTRTQEVFKG-AFG-------
FYHDFA-MTEKHYVFLQNPI--RLNLYKF----VTQYML--GKAGVAECL-------------------------
EFDTSQKSL-IHLIPRP--------G-------KGGMRGTYE--------------T-----T--
PFFAFHHCNAF-------ESADGKTLILDTVAEL--KGVI-----------------FSA----------------
---------KVEGPDTVYGREENRG----------------------SLTRLSINL--------------------
--ASGKVD--------------------------

```
--------Q-------------------------------------------------------------------
----------------------------------------------------------------------------
----------------------------------------------------------------------------
----------------------------------------------------------------------------
----------------------------------------------------------------------------
----------------------------------------------------------------------------
-----------------------------RVLIDRSV----------------EFPSVA-
STVVAKPHRHIYMVGDRFNSPDTW------------GPVQVICKVSISEGIESLSPKVSQ----------------
---DV--------YYPGKSCW--VHEPVFVPR---P-NAKA---EDDG--WVLAMVNDS-------T--------
TGQAFM---AILEAQDLAAGP--IAKIHLK--HHY-PQALHGSW--------------------------------
------------SPDFIGPDPKQPYEPRLHDTRGVDPLVYA-----------------------------------
----------------------------------------------------------------------------
----------------------------------------------------------------------------
----------------------------------------------------------------------------
----------------------------------------------------------------------------
----------------------------------------------------------------------------
----------------------------------------------------------------------------
----------------------------------------------------------------------------
-------------------------------------
>CCD_Ostreococcus_lucimarinus_CCE9901
----------------------------------------------------------------------------
-------
MGSLSAHNAPVRYYKTYRRKSCLLSLQTRRVSSILRVQTETDLSTRTIQEIDSIQPSSDEDTGYISAAASTSYQLL
DPVEY--------------------------------------------GEHGAAFL------SGNYYPV-
GQEVTACCDYDAQAGDDSST--MKI------------------NGK--
IPSDFPLGQYAYVGPNPKFAVDHYKRWGAGPGQVDFGL--GSGWHHWFEGDGMIYAVDF---C-----TGN-----
RV------KFRNRFIRT------SSWKREIESGKR--IFRP-LM----NADGGTFLANAL----------
SNLITGGMF--------------------MKDSANTALLHFA--------GR----------------------
TFALQ-DTC--PPWEL----DPDTLETI--SA-------C----------T-------------------FD--
GKLPP--YVP---------FTAHPKVLPS------TGELIF------------F--GFNPVSP----------P--
-HCSVGTL-------------------------------------KPD---------GT----------
ISSINSLWSIPFV-GSI-------FMHDFC-VTEHFTVLFEGSM--DIKPVRQ----LL------G----KHPL--
-----------------------QYNEDKRAR-FGVLNRD-------AQ-------STS--VTWF-----------
---N---CSS--SQMVYHFINSW----EEKNERNEQLIVITGIRED---------------------------
GFFQEAM-------RA-------NGSSEWIKEAVRVGKIP----------------------KVHEWRINL-----
-----------------------------------------------------------VTGKVT-----------
------------------E-RYLF----------------------------------------------------
----------------------------------------------------------------------------
----------------------------------------------------------------------------
----------------------------------------------------------------------------
----------------------------------------------------------------------------
----------------------------------------------------------------------------
--------------------------------------------SI----PL----------------ETPRIN-
DAFVGRRNRYAYAGRVLLSELEST----------TQLKFDAVVKIDMQEQ--------KT----------------
---VT--------YEHGPNRY--GMEAQFVGR---P-GGVD---EDDG--WLVMYVHDE-------SYTATDV--
NGRTEC---VIIDAQDVEAGP--VATILLP--ERV-PYGAHCMW--------------------------------
-------
RADLKYPGATFEDTAVAFEALSANFQVNEPKMFAFEQSQRGELLEAVWKGLSRVALGLFVHGWSPRIARENVTEYA
FVRGAGLRFLEVSKLGSHRLREAVKERADATPAPPTLTLYDVEDDDSCRLVREVLSICDVSYLCKPCPTASCSNSS
ELAILQGVELGSEVVPFLRDDRNDDIAVKGADGIIQYLYQEYLDGEEPASLVSLFQRFAQASKINDASHRRTSRAG
EQPLIFWGYEASPFCALVRKALNERGISYVFRPCARGSPRRSLLLNRTGIFQVPYLEDPNTGISLFESVDIISYLK
KTYD------------------------------------------------------------------------
----------------------------------------------------------------------------
----------------------------------------------------------------------------
----------------------------------------------------------------------------
------------------------------
>CCD8_Saccharum_ROC22
```


MSPTMASPFCVLQPLAASVVTETPTPTPAIAPVPPARPLV------------------------------------
--------DAPRRRGG-RSTGEHAAWKSI-RQER------W-E---GA----LEL------------------
EGE--LPLWL-DGTYLRNGPG-----LW------NL-----GD---YGFRHLFDGYATLVRVSF---R-----
DGH------A------VGAHRQIES------EAYKAARAHGKV--CYRE-FS----EV-PKADSLLSY---V----
--GQLATLFSG----------S--------SLTDNSNTGVVRLGD-------GR----------------------
VLCLT-ETI--KGSIVV---DPDTLDTL--GK-------F----------E-------------------YT--
DKLGG--LIH----------SAHPIVT--------DTEFWT------------L--VPDLIRP-------------
-GYSVVRM-------------------------------------DAG---------TN---------
ERRFVGRVDCRGGP-APG-------WVHSFP-ITDHYVVVPEMPL--RYCARNL----LRA-----EPT-PLYKF-
------------------------EWHPESGSY-MHVMCKA-------SG-------RVV--ASVE----------
----V-----P--PYVTFHFINAY---EEKDDEGRVTAIVADCCEHN-ANTSI-----------------LDK--
LRLQNL-------RS--------------STGQDVLPDA----------------------RVGRFRIPL------
---DGSPFGELE------------
-------------------SALD---

---PDQHGRGM----------------DMCSIN-
PAHIGKKYRYAYACG-AQRPCNFP---------------NTLTKIDLVEK--------T-----------------
---AK--------NWYEEGAV--PSEPFFVPR---P-GAVE---EDDG--VAISMVSAK----------------
DGSAYA---LVLDAKTFQEIA--RAKF--P--YAM-PYGLHCCW--------------------------------
------------VPRTSDA---

>CCD8B_Prunus_mume

MASITFSGTSGNSFFLPTASVHSNQNIRDGFNPLKKNLSDTRKKHNGRGLVSTNVASPPLPAVAPLLPRRE-----
---SGNSGNRS------HVAWTSV-RQER------W-
E---GE----LLV------------------QGE--IPFWL-KGTYLRNGPG-----LW------HI-----GD--
-YNFRHLFDGYAMLVKLQF---E-----DGR------L------IAGHRQIES------EAYKAAMKTQKL--
CYRE-FS----EV-PKPDNFLSY---I------GELANLFSG---------A-------
SLTDNANTGVVMLAD-------GR----------------------VVCLT-ETQ--KGSIVI---DPATLDTL--
GK-------F----------E-------------------YS--DTLGG--LIH----------SAHPIVT-----
---ETEFLS------------L--LPDLINP--------------GYLAVRM------------------------
-------------EPG---------TN---------ERKVIGRVACSRGP-APG-------WVHSFP-
VTEHYIIVPEMPL--RYCAGNL----LKA-----EPT-PLYKF-------------------------
EWHPESKAF-MHVMCKA-------SG-------NIV--ASVE--------------V-----P--
LYITFHFINAY---EEKDEDGRVTAVIADCCEHN-ADTTI-----------------LDK--LRIHNL--------
-------------RSYTGQDVLPDA----------------------RVGRFRIPF--------------------
---DGSAYGKLE--------------------------
-----AALD---

-----------------------------PNEHGRGL----------------DMCSIN-PAYLGKQYRYAYACG-
AQRPCNFP---------------NTLTKIDLVNK--------K--------------------AK--------
NWHDEGAV--PSEPFFVAR---P-GATE---EDDG--VVISMVSGK----------------NGDGYA---
LLLDGSTFQEIA--RAKF--P--YGL-PYGLHGCW---
---VPSK---

>CCD8_moss_Physcomitrella_patens

MTSAMSRLAPPTCSTYQSITVIDDLHQRSTQPSTRWLQPATRLKNHALASRRRSNQSIRMPTISAKSSPDRDQRTK
AIAQT--VQSESIPAGDYYPGSNAWQTV-KQDE-
-----------WQGE--MEV------------------QGE--IPSWL-DGTYLRNGPG-----YF------EA--
---GG---KEFPHLFDGYSTLIRLNF---N-----NSK------L------IAHHAQLQS------
EAYKAVKSSGKV--SFRE-FA----VT-PKHNNVFEW---M------GEVAGIAMG----------T--------
TLTDNANTGVIKLGD-------GR----------------------VVCLT-ETC-KGSIQI----NPDTLETI--
GQ-------F----------K-------------------YT--DNLGG--LIH----------AAHPYVD-----
---ENEMIT------------L--LPDLLNP--------------GYTAVRM------------------------
------------VAGT---------NE----------RVPIGRVNCNGP--QPG-------WVHSFA-
VTENYIVVPEGPL--RYSVRNL----LKA-----EEA-EYFKF-------------------------
EWLPESGAW-IHIMDRF-------TG-------KIV--TCVE--------------V-----P--
NFVTFHFINGY----EDVDENGKPQIIVDCCEHH-ADPVI-----------------LKR--MKLNEL-------
RS---------------YPGKVLPDA----------------------RVGRFTIPL-------------------
--DGSKTGTLT-------------------------
------AAVP--

------------------------------IEEHGAGL----------------DMNTIN-
PVYTSKKYRYVYACG-ASRPCNFP---------------NTLTKIDLETK--------T-----------------
---AK--------NWHHPGGI--PTEPFFVPR---P-GATE---EDDG--VLISLVSDD----------------
SGGGFI---LILNGSDFTELA--RADL--P--YGL-PYGLHGCW--------------------------------
------------VPGQSSRLRI--

>SiCCD7_Solanium_lycopersicum

MDLQFVSLPPNSKTKAKMQAKACHNINNIPPKLLPPAKLPSTVAMSPSQLTLPSHVARAITITTSPTHEVYT--
PEIDD---TVTAYWDY------QFLFVSQ-
RSEA------T-E---PV-S--LRV-----------------VEGS--IPSDFPSGTYYLTGPG-----LF-----
-A------DD--HGSTVHPLDGHGYLRTFEI---DGS---TGQ------V------KFMARYIET------
EAQTEERDPVSG--KWR--FT----H--RGPFSVLKG----------GKMVGNTKV----------M---------

-KNVANTSVLQWG--------GR----------------------LFCLW-EGG--DPYEI----DSKTLNTL--
GKFELIKN-S----------DQVLEDKKISHSDFLDVAAQLL-KPILYG--VFK----MSPKRLLSHYKIDTR---
---RNRLLI------------M--SCNAEDM---------LLPRSNFTFYEF------------------------
-------------DSN---------------------FQLLQSQEFEIP--DHL-------MIHDWA-
FTDTHYILFGNRI--KLDIPGS----MTAVC---GLSPMISAL-------------------------
SVNPSKPTSPIYLLPRF-------RN-------NNV-ERDWR--------------KPIEAPS--
QMWVLHVGNAF-EEIDEQNGNLNIQIQASGCSYQWFNFQK-----------------MFGYDWQSGKL--------
---------DPSMMNVEEGEEKLLP----------------------HLVQVCINL--------------------
--------------------------------------DKKGNCTKCSVN--------------------------
---DLNPEW---

-----------------------------NK----AA----------------DFPAMN-
PEFSGRKNRYIYAATCTGSRQALP-----------HFPFDAVVKLNAVDK--------SV----------------
---QK--------WSAGRRRF--IGEPVFIPR-----GTNK---EDDG--YLLVVEYAV-------S--------
TQRCYL---VILDAQKIGEKNEVVARLEVPRHLNF-PLGFHGFW--------------------------------
------------APTNSSLANLQKIESKCKNSWSMMKDNMVKLGQ-------------------------------
----

>SiCCD8_Solanum_lycopersicum

MASLASSTTKIYCNKILPDMFDHGKHESHLGSKLKNNEKNKKKLDLKLVTKVANQLPVI-----------------
---------------------------VPPPDQEVISKEKKLAAWTSV-RQER------W-E---GE----LVV--
----------------EGE--LPLWL-NGTYLRNGPG-----QW------HI-----GD---
YNFRHLFDGYATLVRLHF---E-----NGR------L------IMGHRQIES------DAYKAAKISKKI--
CYRE-FS----EV-PKVDNFLSY---I------GDMAKLLSG----------A--------
SLTDNANTGVVKLGD-------GR----------------------VVCLT-ETI--KGSIVI---DPNTLDTI--
GK-------F----------E-------------------YS--DSLGG--LIH----------SAHPVVT-----
---DSEFIT------------L--IPDLMNP--------------GYTVVRM------------------------
------------EAGT---------NE----------RKYIGRVSCRGGP-APG-------WVHSFP-
VTENYVIVPEMSL--RYCAKNL----LKA-----EPT-PLYKF-------------------------
EWHPDSKAF-VHVMCKA-------SG-------NIV--ASVE--------------V-----P--
LYVTFHFINGY---EEKDEDGRVTAVIADCCEHS-ADTTI-----------------LDK--LRLENL-------
RS--------------FNGKDVLPDA----------------------RVGRFRIPL-------------------
--DGSPYGELE-------------------------
------AALD--

------------------------------PNEHGKGM----------------DMCSMN-
PAYLGKKYRYAYACG-AKRPCNFP---------------NTLTKIDLFDK--------K-----------------
---AK--------NWYDEGAV--PSEPFFVAR---P-GATE---EDDG--VVISMISDK----------------
NGEGYA---LILDGSTFEEIA--RAKF--P--YGL-PYGLHGCW--------------------------------
------------VPKI--

>CCD8_DAD1_Petunia_hybrida

MASFAHSATKIYCNKILPERFDHGKDEPHFGKIKINEKNKKNMDLKLVTNVASQLPVI------------------
-------------------------------VSPLDQEFVKKEKKLAAWTSV-CQER------------WEGE--
LVV------------------EGE--LPLWL-SGTYLRNGPG-----LW------HV-----GD---
YNFRHLFDGYATLIRLHF---E-----NGR------L------IMGHRQLES------DAYKAAKKNNKV--
CYRE-FS----EA-PKPENFLSY---I------GDMAKLFSG------------------
ASLTDNANTGVIKLGD-------GR----------------------VVCLTETIK--GSITI----DPMTLDTI-
-GK-------F----------E-------------------YS--DSLGG--LIH----------SAHPIVT----
----DSEFFT------------L--IPDLINP--------------GYVVVRM-----------------------
-------------EAGT---------NE----------RKFIGRVSCRGGP-APG-------WVHSFP-
VTEHYIIVPEMPL--RYCAQNL----LKA-----EPT-PLYKF-------------------------
EWHPHSKGF-MHVMCKA-------SG-------KIV--ASVE--------------V-----P--
LYVTFHFINAY---EEKDEDGRVTAVIADCCEHS-ADTTI-----------------LDK--LRLENL-------
RS--------------FDGVDVLPDA----------------------RVGRFRIPF-------------------
--DGSPDGELE-------------------------
------AALD--

------------------------------PNEHGRGM----------------DMCSIN-
PAYLGLKYRYAYACG-AKRPCNFP---------------NTLTKIDLFEK--------K-----------------
---AK--------NWYDEGAV--PSEPFFVAR---P-GATE---EDDG--VVISMISDK----------------
KGEGYA---LILDGSTFEEIA--RAKF--P--YGL-PYGLHGCW--------------------------------
------------VPKK--

>CCD7_Nicotiana_tomentosiformis

-------------------------MQAKACHNI--IPPKLLPPAKLPSTA----
SHITLPSHVPRAITITTSPTHEVYTPVPEIDD--
-TITAFWDY------QFLFVSQ-RSEA------T-E---PI-T--LRV-----------------VEGA--
IPTDFPSGKYYLTGPG-----LF------A------DD--HGSTVHPLDGHGYLRTFEI---DGG---SGQ-----
-V------KFMARYIET------EAQAEERDPVTG--KWR--FT----H--RGPFSVLKG----------
GKMVGNTKV----------M----------KNVANTSVLQWG--------GR----------------------
LFCLW-EGG--DPYEI----DSKTLKTV--GKFELINN-C----------KSLAEQKKLINGDFLDVAAQIL-
KPILYG--VFK----MPPKRLLSHYKIDAR------RNRLLI------------A--SCNAEDM---------
LLPRSNFTFYEF-------------------------------------DSN---------------------
FQLLQSQEFDIP--DHL-------MIHDWA-FTDTHYILFGNRI--KLDIPGS----MTAVC---GLSPMISAL--
-----------------------SVNPSKATSPIYLLPRF-------SD---HNQTNNIVQRDWR-----------
---KPIEAPT--QMWVLHVGNAF----EEKDENGNVNIQIQASGCS-----------------------YQW--

FNFQKM-------FGYDWQSGKLDPSMMNVEEGEEKLLP----------------------HLVQVSISL------
--DTNGNCTRSSVN------------
-----------------DLNTQW---

---NK----AA----------------DFPAMN-
PDYSGKKNEYVYAATSSGSRQALP-----------HFPFDTVVKLNTADK--------SV----------------
---RK--------WSAGRRRF--IGEPVFIPR------GIT---EDDG--YLLVVEYAV-------S--------
TQRCYL---VILDAQIIGEKNEVVARLEVPRHLNF-PLGFHGFW--------------------------------
------------APSNTGQGNLPNFESKCKDSWSMLEDNMVNLGNSKNSR--------------------------
----

>CCD7_Petunia_hybrida

------------------------MLQAKACHNI--IPSKLLPPAKLPSTA----
SQLTLPSHVPRAITITTIPSHEVYT--PEIDD--
-TITAFWDY------QFLFMSQ-RSET------T-E---PI-A--LRV-----------------VEGA--
IPSDFPSGTYYLTGPG-----LF------T------DD--HGSTVHPLDGHGYLRTFEI---DGG---SGQ-----
-V------KFMARYIET------EAQAEERDPVTG--QWR--FT----H--RGPFSVLKG----------
GKMVGNTKV----------M----------KNVANTSVLRWG--------GR----------------------
LLCLW-EGG--EPYEI----DSKTLNTV--GKFELIKT-C----------ESLAEEEKF-HGDFLDVAAQIL-
KPILYG--VFK----MTPKRLLSHYKIDAR------RNRLLI------------M--TCNAEDM---------
LLPRSNFTFYEF-------------------------------------DSN---------------------
FKLLQSQEFNIP--DHL-------MIHDWA-FTDTHYILFGNRI--KLDIPGS----MTAVC---GLSPMISAL--
-----------------------SVNPSKPTSPIYLLPRFPDDKNHETT-------NINVQRDWR-----------
---IPIEAPT--QMWVLHVGNAFEEIRD--ENG---NVDIQIQASG-CSYQW----------------------
FNFQKM-------FGYDWQSGKLDPSMMNVNEEKEKLLP----------------------HLVQVSISL------
---DTNGNCTKCSVND------------
-----------------L-NPQW---

---NK----AA----------------DFPAMN-
PDFSGRNNAYVYAATSSGSRQALP-----------SFPFDTVVKLNTANK--------SV----------------
---HT--------WSAGRRRF--IGEPVFIPR------GTI---EDEG--YLLVVEYAV-------ST-------
Q-RCYL---VVLDAQKIGDKGEVVARLEVPRNLNF-PVGFHGFW--------------------------------
------------APSNSAL---

>BCOa_Oikopleura_dioica

```
----------------------------------------------------------------------------
-------------------------------------------------------------
MKVFNFFFSLALAND--------------------------------------------------------
EASYTEDV------GGLFRTM-EEHP--------E---RVSL--SNI------------------DGQ--VPQWL-
KGSMFRNGPG-----QY------EY-----GT---DHFNHIFDPSAIIQKIQF---D-----NGK------V----
--HYQSKYIDS------THRLANVEKNAI--VYTE-MG----TW-AEPERYEEM---EDEELSMARCLHLNES---
----------------FPSDNTIVSLYPIH--------GW----------------------MVGFT-ESR--
MVTLN----DPETLETK--HVLDLGKSAS----------E-----------------------GYFII--TV----
-------LAHGSLD-E------NGDFWT------------M--SVGVQKE------SYAFMPKTAYGTLKI-----
-------------------------------KNAMRDSENPFAYQA--------
TPEEFLASVEFGESLFNQDERDRTVRYFHMFV-QTTEYLVLPVTSI--ELDPAKM----ANACKE--GQP-
PMDMM-------------------------FYNEEKPGY-FRIFDKK--------------------NMKWF----
----------PKTFTTE--AFMQIHMIQAY-------VNEEGSKIIFDTCETP--KGDI-----------------
MMA--YYLETV-------NA--------TGQALQEVHESMLPIG----------------------VPVRYEFGL-
------------------------------------------------------------DGLNSKTEE-------
------------------------YVEP------------------------------------------------
----------------------------------------------------------------------------
----------------------------------------------------------------------------
----------------------------------------------------------------------------
----------------------------------------------------------------------------
----------------------------------------------------------------------------
------------------------------------------------TVLFEPDMENWPMYTTAGT-----
DFPMIN-FDYIGVEYDHFWSVG---MGNVMA---------------DRLYHSQISTG--------D----------
----------RH--------VWREVGYS--PSEPFFVSR---P-GASS---ETDG--VLLSLVSAF----------
--EGHSNLRPFM---LILNPIEMTE----IARIWLPEDYDI-SVSFHGTF--------------------------
------------------VKH-------------------------------------------------------
----------------------------------------------------------------------------
----------------------------------------------------------------------------
----------------------------------------------------------------------------
----------------------------------------------------------------------------
----------------------------------------------------------------------------
----------------------------------------------------------------------------
----------------------------------------------------------------------------
-------------------------------------------
>BCOb_Oikopleura_dioica
----------------------------------------------------------------------------
-------------------------------------------------------------
MNFLLLIFAGIFCSDVND-----------------------------------------------------
DISLTENV------GRAYQSM--SEI------P-E---PV-E--LKT----------------EIIGT--VPKWL-
FGSLYRNGPG-----KF------EY-----GN---DTFEHLFDPSAMIQRLHF---E-----DGR------V----
--FYQRKLIKS------THTLLNMKEKRI--VLTE-MG----TW-TEPENTDEI---HAEDISKERCKHLLNS---
-------F---------PTDNTIVSVFPIH--------GW----------------------IVAFT-ESP--
IITLN----DRITLETE--HI-------L----------D-------------------IR--NAPAF--PEG---
-LALTTVLAHGVTD-K------NGDFWT------------F--ATGVWTE------QFAFIPKVVYLAVKI-----
-------------------------------ANASKLD------KS--------
SPEEILKNISFSDFLFNSNVRDFKMRYFHSFVQASEDFLILPFTSV--EFQPDAI----LNSCKN--GQP-
PIEMM-------------------------NFNKNADGF-YRIFDKK-------TM-------KWL-PKEFI----
----------S-----E--TNFQMHPIQGF----------------------------------------------
YDH--LFFENL---------------NKTGEALVDAYWSIIPVG----------------------TPVRYRMST-
---------------------------------------------------------VDLNSVTTESIS-------
----------------------RIAIFN------------------------------------------------
----------------------------------------------------------------------------
----------------------------------------------------------------------------
----------------------------------------------------------------------------
----------------------------------------------------------------------------
----------------------------------------------------------------------------
------------------------------------------------DDFDKFQSYTLAGT----------
```

```
DFPTVD-ISRLDKEQDDFWSLG---VGNFVA---------------DRIYHSKISTG--------E----------
----------RW--------IWRKPGFS--PSEPIFVRE---P-GKTD---SLEG--VIISVASPV----------
--DVNSTEKAFV---LILEPEDLSEIA--RIYFEED--VKF-ALGFHGIF--------------------------
------------------VPFQ------------------------------------------------------
----------------------------------------------------------------------------
----------------------------------------------------------------------------
----------------------------------------------------------------------------
----------------------------------------------------------------------------
----------------------------------------------------------------------------
----------------------------------------------------------------------------
----------------------------------------------------------------------------
-------------------------------------------
>BCOc_Oikopleura_dioica
----------------------------------------------------------------------------
-------------------------------------------------------------
MNFLLLIFAGIFCSDVND-----------------------------------------------------
DISLTENV------GRAYQSM--SEI------P-E---PV-E--LKT----------------EIIGT--VPKWL-
FGSLYRNGPG-----KF------EY-----GN---DTFKHLFDPSAMIQRLHF---E-----DGR------V----
--FYQRKLIKS------THTLLNMKEKRI--VLTE-MG----TW-TEPENTDEI---HAEDISKERCKHLLNS---
-------F---------PTDNTVVSVFPIH--------GW----------------------IVAFT-ESP--
IITLN----DRITLETE--HI-------L----------D-------------------IR--NAPAF--PEG---
-LALTTVLAHGVTDK-------NGDFWT------------F--ATGVWTE------QFPFIPKVVYLAVKI-----
--------------------------------ANA---------SK----
LDKSSPEEILENISFSDFLFNSNVRDFKMRYFHSFVQASEDFLILPFTSV--EIQPDAI----LNSCKN--GQP-
PIEMM-------------------------NFNKNADGF-YRIFDKK-------TM-------KWL--PKEF----
----------I---S-E--TNFQMHPIQGF----------------------------------------------
YDR---------------------------------------------------------------ESEEFVLDT-
----------------------------------------------------------------------------
----------------------------------------------------------------------------
----------------------------------------------------------------------------
----------------------------------------------------------------------------
----------------------------------------------------------------------------
----------------------------------------------------------------------------
----------------------------------------------------------------------------
----------------------------------------------------------------------------
-----------------------------------------------LEIMNH-----------------------
------DM------------------IQEKVFSSM-----------------------------------------
-------------------------ISKF-----SIV---------------------------------------
----------------------------------------------------------------------------
----------------------------------------------------------------------------
----------------------------------------------------------------------------
----------------------------------------------------------------------------
----------------------------------------------------------------------------
----------------------------------------------------------------------------
----------------------------------------------------------------------------
----------------------------------------------------------------------------
---------------------------------------
>BCOd_Oikopleura_dioica
----------------------------------------------------------------------------
--------------------------------------
MTFSGKLQKFNQVQQTSDLKHLPMVTQRSARAFTFTQTVRCCNKGTII----------------------------
------------------AKQKETGMPATVPVSEIVQSV-PELE------------EEQP--TKI-----------
-------TGLGTIPEWL-EGTILRNGVG-----QW------DL-----GG---ETVCHWFDGHAFIHKFEI---K-
----NGS-------------GTRDIIVVS--------RKQIVSDKRL-------FN--------------------
-------------------------------------DNCNVNLMPIG--------DK------------------
----VFAMT-ETT--AIREV----DLKTLETG--ER--------------------------------------
LAYNDYMTI--HTA----------TAHPHILPG-------GEFLN------------M--GSSFGKE---------
```

----TCYNVIQV-------------------------------------DST---------GD-------
FDKMKVVAKFPATNPK-CPS-------YYHSFG-LTRDHILFLEMPY--RINMLKM----LTR-KFT-NAA-
PAEAM-------------------------FYDKQVHSS-IRIADLK-------TG-------DIL-PIKIN----
----------T-----E--GMGSFHFGNSY----EIEVDGGIFCVWDACPNYK--AQGA-----------------
AHL--FNIETL---------------RSRSAADIDEAFKEAGEN----------------------ELARFVFPI-
--KLPRDATVGEDLN-------
---KRLAGKLQTTASAVLKEDGSVWLEK--

--ELLIDNSDEKIGFYGF--------
EFPRYN-YALNGLPYSYLYGNG---FGTILP---------------DRIIKVNVNTK--------D----------
----------MK--------VWQEKDIF--PSEPVFVQNKSVT-DPSAPGEEDNG--VILSICVST-------KK-
------DVPAFL---IILDAKTLTELA--RCTTTV---TGL-PYGFHHTF--------------------------
------------------LPSDF---

>CCO_Long_Blastomyces__gilchristii_SLH14081

MAKGIGRNHDAIRVVNLGQEFGLTITQIKMTKSLTSTESLENGP--------------------------------
------------PPDTNPVT------LGLYASP-EIRE------------PT-N--LKI-----------------
-EGT--IPTWL-TGSLYRGAAA-----TW------DV-----GT---YTAEHWFDGFSRNHRFEI---A-----
NGA------V------SYRSRNGAE------ELMDFVRETGR--------YP----TSSFGSDPCKVI---F----
--GAFEATYRD--------------GSSSRGKASSSNVGVSYVPNFAGIPGNST--------------
TQGAPFDALVSTT-DAN--ELQRI----DPVTLEPG--EL-------F----------T-----------------
--YEASNKLLV--NSG--------QSAAHPVVG-E------DGAVYN------------Y--VLDQKTS-------
--PP---TYYIFGI------------------------------------SPPK---------GE----------
TKILATITD--A--PAS-------YIHALF-GSEKHLVLVVWQA--DFAKEAI---------------TILDSIG-
-----------------------DWDPERKTL-FYVIDRA-------NG-------GLL--RKYE-----------
-------SPD--AFFAFHEINTF----EN-EAG---DIFVDLPRMD--NYSF-----------------LSA--
AKISNL----------------RANLGTPNANSSNDLAG----------------------AFTRYRLPY------
--HDSHAPLADGSTLP------------
-------------------TYKA---

---EIDISLPLAQANI-----------ELPRIH-
PGKMGRPYRFSYGIH-VEKTGNFA---------------DSIIKIDGDEK--------------------------
---TW-------LVWAPATRH-VPSEPIFVPR---P-GATD---EDDG--VLLTVVMDV-------H--------
VKQSSL---VVIDVKTMKELG--RARM--P--IVM-TYGFHGTW--------------------------------
------------GQGPIQPRAGEL--

>CCO_Pseudogymnoascus_sp_VKMF4246

MVPSTFLVLSSLALGISATAVDSQH--
HSPANPFI------VGFYGTP-ETRD------------PI-N--LKV------------------KGK--IPRWV-
SGSLYRGASA-----TW------DN-----GN---YTSEHWFDGFSRNHRFEV---S-----DGK------V----
--SYRSRNSSD------ELEDFVRETGLF-------PG----GS-FGGDPCKAI---F------GAFETTYRD---
-------G-------INPVGNVSTDTVMVSFITNFPGLDR----------NVSGTEHGPFVTLVKTT-DGN--
SLQQI----DPVTLEPM--EL-------F----------N-------------------YK-STNINI--TDG---
-----KTSSHPAHG-K------SGELYN------------YFLNADATPP--------------EYNIFEV-----
--------------------------------NSS---------GE----------GSILATITDA----PPA---
----YIHSMF-STENYVILIVWQS----DFGKV------------TPKPYYNVLDNIK------------------
---DWDPERKTL-FYVVDKV-------KG-------GVI--AKYT--------------S-----E--
TFFAFHEINSF----ED-SDG---SIVIDLPVMK--TNAI-----------------LEA--ARLQNL--------
--------RTYVGRHNATAPHDIAG----------------------AFRRFRLQD--------------------
--------------------------------------YAHGIQANGTLI--------------------------
---TRPAVT---

-----------------------------DFELDFKSGNI-------------ELPRLN-EAYQGKPYQYAYGIH-
IKERGFFT---------------DSLIKIDTHTR--------K--------------------TK--------
LWKPRTPH-LPSEPVFVAN---P-QGTR---EDDG--VLLTMALDA-------N--------RKQSAL---
VVIDARNMKEIA--RAEM--P--VVA-GFGFHGVW---
---GNN--

>CCO_Blastomyces__gilchristii_SLH14081

MTKSLTSTESLENGP---
PPDTNPVT------LGLYASP-EIRE------------PT-N--LKI------------------EGT--IPTWL-
TGSLYRGAAA-----TW------DV-----GT---YTAEHWFDGFSRNHRFEI---A-----NGA------V----
--SYRSRNGAE------ELMDFVRETGR--------YP----TSSFGSDPCKVI---F------GAFEATYRD---
-----------GSSSRGKASSSNVGVSYVPNFAGIPGNST--------------TQGAPFDALVSTT-DAN--
ELQRI----DPVTLEPG--EL-------F----------T-------------------YEASNKLLV--NSG---
-----QSAAHPVVG-E------DGAVYN------------Y--VLDQKTS---------PP---TYYIFGI-----
-------------------------------SPPK---------GE----------TKILATITD--A--PAS---
----YIHALF-GSEKHLVLVVWQA--DFAKEAI----TI----------LDSIG----------------------
---DWDPERKTL-FYVIDRA-------NG-------GLL--RKY---------------E---SPD--
AFFAFHEINTF----EN-EAG---DIFVDLPRMD--NYSF-----------------LSA--AKISNL--------
--------RANLGTPNANSSNDLAG----------------------AFTRYRLPY--------------------
------------------------------------HDSHAPLADGSTLP--------------------------
-----TYKA---


```
----------------------------------------------------------------------------
----------------------------------------------------------------------------
----------------------------------------------------------------------------
-----------------------------EIDISLPLAQANI-----------ELPRIH-PGKMGRPYRFSYGIH-
VEKTGNFA---------------DSIIKIDGDEK-----------------------------TW-------
LVWAPATRH-VPSEPIFVPR---P-GATD---EDDG--VLLTVVMDV-------H--------VKQSSL---
VVIDVKTMKELG--RARM--P--IVM-TYGFHGTW-----------------------------------------
---GQGPIQPRAGEL-------------------------------------------------------------
----------------------------------------------------------------------------
----------------------------------------------------------------------------
----------------------------------------------------------------------------
----------------------------------------------------------------------------
----------------------------------------------------------------------------
----------------------------------------------------------------------------
----------------------------------------------------------------------------
----------------------------
>CCO_Aspergillus_oryzae_RIB40
----------------------------------------------------------------------------
-------------------------------------------------------------------
MTIATGATESQ------------------------------------------------------THFNNWPN---
---DLGFDAN-YEER--------S---PV-E--LIV------------------TGH--IPTYA-AGVLYRTGPG-
----KY------KIDREN-GD--TFQVSHWFDGFSETHRFQLVAPDATHS-SLR------V------
FYNSRFSTD------YLIEQARKTGRL--DKIS-FG-------QKRDPCKTV---F------
QKAQSDFNPDPSSANVGVTLSVNMPGLDKALDENRPNIGRWN--------GS-----------------
SGIQTLYAKT-DST--SYKKL----DPETLEPV--GLASQK--------------D-------------------
LH--PDLDG--PLS----------ASHARSDPI------TGDVYN------------F--NLAFGKC---------
-S---TYRVFHV------------------------------------SAST---------GK----------
TTILATFEG-----TPA-------YLHSLF-LTKDYVILCVWNS--HINPAML------------EKGSFLQAIQ-
-----------------------SFDASQPTK-WYVVDRT-------QG-------QGL-VATFE-----------
---S-----L--PFFCFHTINAW---QESSASGTGVDIVAELVRYD--NADT-----------------LHA--
LYYEKL-------------VSSSAEAKAHAQIKKDTYRS----------------------EFARFRLPN------
------------------------------------------------------IPVSPSTEIK------------
-----------------TAAAEW-----------------------------------------------------
----------------------------------------------------------------------------
----------------------------------------------------------------------------
----------------------------------------------------------------------------
----------------------------------------------------------------------------
----------------------------------------------------------------------------
-------------------------------------------MSCKAFSP----------------ELPTMN-
PKLVTQKHRYTYAIT-ARDKSTFF---------------DGIVKFDSQTK--------E-----------------
---TL--------LWNDHAQS--PGEPIFVPR---P-DGLE---EDDG--VLLSVVLDG-------Y--------
SGKSYL---LCLDAQTLKELG--RAHVNGP----I-GFGFHGQH--------------------------------
------------IPSNGVPTGDY-----------------------------------------------------
----------------------------------------------------------------------------
----------------------------------------------------------------------------
----------------------------------------------------------------------------
----------------------------------------------------------------------------
----------------------------------------------------------------------------
----------------------------------------------------------------------------
----------------------------------------------------------------------------
-------------------------------------
>torulene_oxygenase_Beauveria_bassiana_ARSEF2860
----------------------------------------------------------------------------
---------------------------------------------------------
MALNDPGVILRKAADEGEDAADIQNYRLQ----------------------------------------------
ESWKEWPN------EAGFEGL-EEHR------------GPIA--LNL------------------KGS--IPDWA-
AGALYRTGPG-----QS------SVETNSNGT---HNITHWFDGLAHSHKFDI---VPSSTPGGK----TSV----
```

--MYSSRRQSK------RLEQHIKETGKR--GGAS-FG-------QRADPCMGI---F------AKAMGMFAP---
------------------RDQYNNNVVLLPNFPGIDKDRK---------HTDQTGHRPGLDNLYIST-DTA--
GLQKI----DPQTLEPL--GFTDQS--------------S-------------------LH--PDLKG--PLS---
-------CAHAQRDPE------NGDLYN------------Y--NLEFGRF----------A---TYRFFRV-----
-------------------------------RAAT---------GK----------TEIFATVSEPSL--APA---
----YIHSFF-LTPNYMVLCVPTT--HLGWKGA--------KILWEQSVLGAIT----------------------
---PFDDSTPCR-WLIVDRT-------GD-------RGV-VARFS--------------T-----P--
ASFFFHSINAFEETILDENNEMRTDLSFEYVRYD--NTDI-----------------LKI--LSYDIV--------
---MDRDDAAKKFFFTDERYKKGHI----------------------RLVRYRFRL--------------------
---------------------------------------PRDGMQQTEEE--------------------------
---ARTAAG---

-----------------------------EEVLSIPAPHAG------------DLPIIN-
SRMVHKPHRYVYSVTSRGISTFLD----------------CLVKTDVQTR--------E-----------------
---AL--------IWRGDKGH-TPGEPIFVER---P-GATE---EDDG--VLLSVVLDG-------V--------
SKTSYL---LCLDAHSLEEVG--RAEADFP----I-GFGFHGMH--------------------------------
-------------QSQF---

>CarT_torulene_oxygenase_Fusarium_fujikuroi

MALNGPGVYHRTREHEQEDASDITKNILA--
ESWKSWPN------EAAFDRL-EEHR------------GPLR--LTL------------------KGT--IPSWA-
AGSLYRTGPG-----QS------RVEDTARGT---HFTTHWFDGFAQTHRFDIIPSE-----DGE----TQV----
--WYSSRRQA-------DEWIADVKKKGW--RSGMTFG-------QKADPCVGI---F------
AKVMTVFEPKLGNHNVALLANVPGVPKDEETEALNGVPGPLG--------HR-----------------
VNTSNLFVST-DYT--GIRRI----DPSTLQPLAETT------------------Q-------------------
YDLHPSLSG--PCS----------CSHAQRDPD------SGDLFN------------F--NLAFGRV---------
-P---TYRIFRV------------------------------------DAAS---------GE----------
TEVLATISDLNVP--PA-------YMHSFF-LTENHVVICIPAS--HYAWRGL--------KTQWEGNIIDSMK--
-----------------------PFDKERKCK-WLVVDRR-------HG-------KGL-VATFS-----------
---T-----P--AAFFFHSINAFEKNIEDEDGTEQTDLFFDLAKYN--NMDI-----------------IKG--
FYYDVL-----------MDRDDATKKYWFKNDRYKNCAP----------------------TLTRYRFRL------
--PSAPTPDTTFSASAEQ------------
-----------------VLAIPS---

---PH----AG----------------ELPTIH-
PLRNGKPYRYVYSASLRGLTTSVD----------------ALVKTDLDTS--------E-----------------
---AF--------IWTGPEGH-TPGEPVFVPR---P-GAEA---EDDG--IVFSLVVDG-------V--------
NEKAYI---LCLNGKTMEELG--RAEA--D--FAI-GQGFHGIH--------------------------------
------------LPAA--

>CarX_CCO_Fusarium_fujikuroi

MKFLQQNSFTQTSMSQPHEDVS--
PAIRHPYL------TGNFAPI-HKTT------------NLTP--CTY------------------SGC--
IPPELTGGQYVRNGGN-----PV------SH-----QD--LGKDAHWFDGDGMLSGVAF---R-----KAS-----
-IDGKTIPEFVNQYILT------DLYLSRKTTSIASPI----MP----SITTLVNPLSTM---F------
QIMFATFRT---IFLVILSNLPGSQQAIKRISVANTAVLYHD--------GR----------------------
ALATC-ESG--PPMRI----QLPSLDTV--GW-------F----------
DGVEAEGEPEISQAGSDDSPFGGSGIFSF--MKE--------WTTGHPKVDPV------TGEMLL-----------
-Y--HNTFMPP---------------YVHCSV------------------------------------LPKS----
-----NE-------KAPGHRLVNQPVLGVS-GAR-------MMHDFG-ASRSHTIIMDLPL--SLDPLNT----
MK------G----KEVV-------------------------AYDPTKPSR-FGVFPRH--------L-------
PSS--VRWF--------------H---T-A--PCCIFHTANTW-DSQS--SEGELSVNLLACRMTS-
STLVYTAGNIRPPVRSRCTQARVWS---------------DE-------
REETACRYKEAPALESPGESTGLADYFPITAESDDYDQCRLYYYEFDL----------------------------
------------------------------------AMESRN--------------------H--------V-
KSQW--

------------------------AL-SAIPF----------------EFPSVR-PDREMQEARYIYGCS-T--
STSCF-----GVALGRADKVDLLVKMDAKTL---
IQRGKKMNATSITGCVDRRSVCEILQEQRKDDPIYIFRLPPNHY--AQEPRFVPR----ACSTE---EDDG--
YLLFYVFDESQLLPSGD----CPPSA-TSEL---WILDAKNM-RDV--VAKVRLP--QRV-PYGLHGTW-------
----
FSSQDIESQRSVESLRSLEVVQRKKEEWVNSGGQIRKSWMVLRE--------------------------------
----
--KLEKAV------

-------------------------------------G--------------------------------------
----

>CAO2_torulene_oxygenase_Neurospora_crassa_OR74A

MSPHEVIGTVPKNSTTFRTQADEHDDHEEALQNLRT--
---GKYEDWPN------EAAFDGL-TEER--------G---PI-K--IAV------------------TGN--
IPTWA-AGSLYRTGPG-----LY------KIDTDA-GT--TFEMSHWFDGLAHTHRFDIIPNE-----EGS-----
-V----DIFYSSRRQAE------EMMDVIKKQGTW--PYYS-FG-------QKADPCLGF---F------
AKAMAAFKG----------
LREPSGEKWHNNVNVAVHVNPPGLEAVRNIVGTRKPAVAENDANVLGHRPELPKSIWVST-DNS--TMKQI----
DPQTLEPI--GWATQD---V------------------------------LH--PELTG--AMS----------
CAHAQRDPD------TGDFFN------------F--NLEFGPK----------P---TYRVFRV------------
------------------------DASS---------GK----------TEILATIREPSV--SPA-------
YIHSLF-LSPSFVILCIPTS--HFGLSGT--------QIPWERNLVDAIK-------------------------

```
PYDPSRKTQ-WIVIDRK-------HS-------KGV-VARFE--------------T-----D--
GRFFFHTVNSFEEKAG--SDSSDINLYCDVIDFG--SHEF-----------------IHS--LYLDVI--------
---LNRDSAAKKFYEDEQRARNSLA----------------------HLTRYHFII--------------------
----------------------------------------NPDSPTTNLP--------------------------
---V-TPTP-------------------------------------------------------------------
----------------------------------------------------------------------------
----------------------------------------------------------------------------
----------------------------------------------------------------------------
----------------------------------------------------------------------------
----------------------------------------------------------------------------
-----------------------------DPKNHEAF--RIPAPHAG------EIPTIN-
PLYATRKHRYVYSLPFRGRGTITD----------------AIVKTDTVTR--------E-----------------
---AL--------FWDNPKGH-TPGEAIFIPR---P-GGEE---EDDG--VLLSLVLDG-------E--------
KGKSYL---LCLDAKTMAEMG--RAEVDFA----I-ALGFHGAH--------------------------------
------------VPSGRTLTRVEGPEY-------------------------------------------------
----------------------------------------------------------------------------
----------------------------------------------------------------------------
----------------------------------------------------------------------------
----------------------------------------------------------------------------
----------------------------------------------------------------------------
----------------------------------------------------------------------------
----------------------------------------------------------------------------
-------------------------------------
>CAO1_CCO_Neurospora_crassa_OR74A
----------------------------------------------------------------------------
----------------------------------------------------------
MAEYVFSDAPKDSHGNGVKDAVPGKQPE----------------------------------------------
ELPPAPRYFQGENTAGFMRPV-RFEG--------D---IT-N--LEV------------------VGE--IPKSI-
EGTFYRVMPE------P------HL-----PS--FIPNDPWFNGDGNISGFYF---K-----DGH------V----
--DLKQRYVRT------EKFVREAEARRS--L----LG----KY-RNRYTDLVE---F------------------
----------------KIRSTANTNIVYWR--------GQ----------------------LLALK-EDS--
PPYAM----DPETLETF--GV-------Y----------D-------------------FD--GQLPS--LTF---
-------TAHPKFDPV------TREMVC------------F--GYEAKGD--------GTR---DICYYSF-----
--------------------------------GPD---------------------GKIAETVWLVSP--VCG---
----MIHDFA-VTENFVIFPIIPL--VCDVERM------------KQG--GDHW----------------------
---QWDYSIPMY-IGVLPRR------GAQ-------GSD--VKWF--------------E---A-P--
HGFAGHVANAF----ED-DKG---HIQLQMAYAK--DNVF----------------------FWWPDA--------
-------------NGKGPRPGEVEA----------------------HFANFVLDY--------------------
--------------------------------------------QSDKLP--------------------------
-----LAEP-------------------------------------------------------------------
----------------------------------------------------------------------------
----------------------------------------------------------------------------
----------------------------------------------------------------------------
----------------------------------------------------------------------------
----------------------------------------------------------------------------
-----------------------------TYLVDDDM----------------EFPRID-
DRVATRKHKHTFFCIFDRKPGVTDFEFVMPRAGGGAPMSNGLAHLNHETG--------DI----------------
---QR--------YLPGPRKL--TGECIFIPR---N-SEAA---EGDG--YVMVLLANY-------E--------
DMCSEL---AVLDTKDLTNE---VALIKLP--VRL-RPGLHGNW--------------------------------
------------VDKSDVDGHPAPL---------------------------------------------------
----------------------------------------------------------------------------
----------------------------------------------------------------------------
----------------------------------------------------------------------------
----------------------------------------------------------------------------
----------------------------------------------------------------------------
----------------------------------------------------------------------------
```

```
----------------------------------------------------------------------------
-------------------------------------
>CCD8_Arabidopsis_thaliana
----------------------------------------------------------------------------
---------------------
MASLITTKAMMSHHHVLSSTRITTLYSDNSIGDQQIKTKPQVPHRLFARRIFGVTRAVINSAAPS-----------
-----------------------------------PLPEKEKVEGERRCHVAWTSV-QQEN------------
WEGE--LTV------------------QGK--IPTWL-NGTYLRNGPG-----LW------NI-----GD---
HDFRHLFDGYSTLVKLQF---D-----GGR------I------FAAHRLLES------DAYKAAKKHNRL--
CYRE-FSETPKSVIINKNPFSGI----------GEIVRLFSG------------------
ESLTDNANTGVIKLGD-------GR----------------------VMCLT-ETQ--KGSILV---DHETLETI-
-GK--------------------------------------FEYDDVLSD--HMIQ---------SAHPIVT----
----ETEMWT------------L--IPDLVKP--------------GYRVVRM-----------------------
--------------EAG---------SN---------KREVVGRVRCRSGSWGPG-------WVHSFA-
VTENYVVIPEMPL--RYSVKNL----LRA-----EPT-PLYKF-------------------------
EWCPQDGAF-IHVMSKL-------TG-------EVV--ASVE--------------V-----P--
AYVTFHFINAY--EEDKNGDGKATVIIADCCEHN-ADTRI-----------------LDM--LRLDTL--------
-------------RSSHGHDVLPDA----------------------RIGRFRIPL--------------------
-----------------------------------------DGSKYGKLE--------------------------
-----TAVE-------------------------------------------------------------------
----------------------------------------------------------------------------
----------------------------------------------------------------------------
----------------------------------------------------------------------------
----------------------------------------------------------------------------
----------------------------------------------------------------------------
-----------------------------AEKHGRAM----------------DMCSIN-PLYLGQKYRYVYACG-
AQRPCNFP---------------NALSKVDIVEK--------K--------------------VK--------
NWHEHGMI--PSEPFFVPR---P-GATH---EDDG--VVISIVSEE----------------NGGSFA---
ILLDGSSFEEIA--RAKF--P--YGL-PYGLHGCW-----------------------------------------
---IPKD---------------------------------------------------------------------
----------------------------------------------------------------------------
----------------------------------------------------------------------------
----------------------------------------------------------------------------
----------------------------------------------------------------------------
----------------------------------------------------------------------------
----------------------------------------------------------------------------
----------------------------------------------------------------------------
----------------------------
>CCD8_Zea_mays
----------------------------------------------------------------------------
------------------
MSPTMASSLCVFAAMSGASGRPSAGGSAVPGRLSSSTQGGKGKRAVVQPLAASVVTETPAPAVAPARPVV------
--------------------------------------DAPPRRRGGRGTVEHAAWKSV-RQER------------
WEGA--LEL------------------EGE--LPLWL-DGTYLRNGPG-----LW------NL-----GD---
YGFRHLFDGYATLVRVSF---R-----DGQ------A------VGAHRQIES------EAYKAARAHGKV--
CYRE-FS----EVPKAEGFLSHV----------GQLATLFSG------------------
SSLTDNSNTGVVRLGD-------GR----------------------VLCLTETIK--GSIVV----DPDTLDTI-
-GK-------F----------E-------------------YT--DRLGG--LIH----------SAHPIVT----
----DTEFWT------------L--IPDLIRP--------------GYSVVRM-----------------------
-------------DAGT---------NE----------RRFVGRVDCRGGP-APG-------WVHSFP-
ITDHYVVVPEMPL--RYCARNL----LRA-----EPT-PLYKF-------------------------
EWHLESGSY-MHVMCKA-------SG-------RVV--ATVE--------------V-----P--
PFVTFHFINAY---EEKDDEGRVTAIIADCCEHN-ANTSI-----------------LDK--LRLQNL-------
RS--------------STGQDVLPDA----------------------RVGRFRIPL-------------------
------------------------------------------DGSPFGELE-------------------------
------PALD------------------------------------------------------------------
----------------------------------------------------------------------------
```


------------------------------PDQHGRGM----------------DMCSIN-
PAHVGKKYRYAYACG-AHRPCNFP---------------NTLTKIDLVEK--------T-----------------
---AK--------NWYEEGAV--PSEPFFVPR---P-GAVE---EDDG--VAISMVSAK----------------
DGSAYA---LVLDAKTFHEIA--RAKF--P--YAM-PYGLHCCW--------------------------------
------------VPRSTSDA--

>CCD7_Zea_mays

MAAMHAIVHHRAPPPGRCCRGHGRSVVVRASAATVTTSIPGSAATVPDS---------------------------
---------------PSAAFWDY------NLLFRSQ-RAES------------PD-PVVLRV--------------
---TEGA--IPPDFPAGTYYLAGPG-----MF------TD-----DH---GSTVHPLDGHGYLRSFRF---DA---
-SGA------A------HYSARYVET------AAKREEHDAGGA--SWRF-------TH-RGPFSVLQG-------
---GSRVGNVKV--------------------MKNVANTSVLRWG--------GR---------------------
-VLCLW-EGG--EPYEL----DPRTLETI--GPFDILGSLCGGTDEVARDASGEAAHHGRRQPWLQEAGIDVA-
ARLLRP--VLSGVYSMPAKRLLAHYKIDPK------KNRLLM------------V--ACNAEDM---------
LLPRSNFTFYEF-------------------------------------DAN---------------------
FALVQKREFVLP--DHL-------MIHDWA-FTDSQYVLLGNRI--RLDIPGS----LLALT---GTHPMIAAL--
-----------------------AVDPSRQSTPVYLLPRT-------PE-----AEAEASGRDWS-----------
---VPIEAPS--QMWSMHVGNAFEERNA--RGGINIQLHMSGCSYQ--WFNF-----------------HRM--
FGYNWL--------------NKKLDPSFMNIAKGREWLP----------------------RLVQVSIDL------
--DKRGTRRGCAVR------------
-----------------RLSDQW---

---TR----PA----------------DFPAIN-
PGFANRRSRFVYAGAASGSRRFLP-----------YFPFDSVVKVDVSDG--------S-----------------
---AR--------LWSVAGRK-FVGEPVFVPT------GSG---EDDG--YVLLVEYAV-------Y--------
DHRCHL---VLLDARKIGERNAVVAKLEVPKHLTF-PMGFHGFW--------------------------------
-------------ADE--

>CCD1like_Zea_mays

MPLLSLPACCGYDAMFRAGLKPTTSSSRCRSSRLIRSHGGAPDHAPPFTQPNSVKVPPSPGLKKQALSVQDMSRAL
KSLSSDLLQRFVDRAY--SFSEEPSL-----
NEGNFRPV-DETD--------E---AVSLSLSSL------------------DGEEVVPADFPEGVYIRNGPN---

--PL------NPTQTVAGSVFGSTSYTYYEGHGMLHAVYF---SRSGGAGGW-----EV------SYRNKYVGS--
----DTLQLERRKKDD--GVAF-VP----SVDGQPYATLAA---F------ALNILRFGK----------------
---AVKDSANTNIFEHA--------GR----------------------AFAVT-ENH--LPYEI----
NISNLSTL--GP--------------------------------------YSVNGAWNQ--PF-----------
TSHPKKIQG------SGELVM------------M--GTNTEKP---------------YYVLGV------------
-------------------------ISS---------DG----------ERLVHKVDLKFEEGK---------
LIHDIG-VTKRYNIIMDHPL--KFGISRV----FL----------KKPLI-------------------------
ENDMDGKSR-FGVMPRF-------GD-------AES--IIWF--------------D---V-K--
NHCSYHLFNCF-------EDGN--EVVIRGCRLL--GSIL-----------PSGRHRADKSKWYGRAF--------
-----LQPDKDSEDFDPSLDGILFS----------------------RPYEWRLNL--------------------
--ENHSVY--------------------------
---EGYITS---

-----------------------------EKVAM-------------------DFPVIN-
DKFMGIQNKYGYAQVADSLETSKT----------GLFKFNMIAKLHFDTP--------DK----------------
---ENEQLIFVEYHTLQEQQF--CSGVQFVVK---E-NGID---EDDG--WLVTYVHDE-------R--------
TNISQV---YIIDAKRFSEEP--VAKITLP--QRV-PYGFHGNF--------------------------------
------------FYTSNQR---

>CCD1_Zea_mays

MDSHRNDGVVVVPAPRPRKGLASWALDLLESLAVRLGH--------------------------------------
----------DKTKPLHW-----LSGNFAPV-VEET------------PPAPN-LSV------------------
RGH--LPECL-NGEFVRVGPN----PKF-------------AP---VAGYHWFDGDGMIHAMRI---K-----
DGK------A------TYVSRYVKTARLKQ-EEYFGGAKFMKIGDLKGF-FG----LFMVQMQQLRKK--------
--FKVLDFTYG---------------------FGTANTALIYHH--------GK----------------------
LMALS-EAD--KPYVVKVL-EDGDLQTL--GL-------L----------D-------------------YD--
KRLKH--SF-----------TAHPKVDPF------TDEMFT------------F--GYSHEPP-------------
--YCTYRV--------------------------------------IN---------KE----------
GAMLDPVPITIP--ESV-------MMHDFA-ITENYSIFMDLPL--LFRPKEM----VKN---------GEFIY--
-----------------------KFDPTKKGR-FGILPRY-------AK-------DDK-LIRWF-----------
---------QLPNCFIFHNANAW-------EEGD--EVVLITCRLE--NPDL------------------------
---------------------DKVNGYQSDKLENFGN----------------------ELYEMRFNM--------
--KTGAAS--------------
---------------Q--

---KQLSVSAV----------------DFPRVN-
ESYTGRKQRYVYCTILDSIAKVTG-----------------IIKFDLHAEPESGVKELEVGGNVQ-----------
---GI--------YDLGPGRF--GSEAIFVPK--HPGVSGE---EDDG--YLIFFVHDE-------N--------
TGKSEV---NVIDAKTMSADP--VAVVELP--NRV-PYGFHAFF--------------------------------
------------VTEDQLARQAEGQ---

>NCED1_Zea_mays

MQGLAPPTSVSIHRHLPARSRARASNSVRFSPRAVSSVPPAECLQAPFHKPVADLPAPSRKPAAIAVPGHAAAPRK
AEGGKKQLNLFQRAAAAALDAFEEGFVANVLER-----------------------
PHGLPSTADPAVQIAGNFAPV-GERP------------PVHE--LPV------------------SGR--IPPFI-
DGVYARNGAN----PCF-------------DP---VAGHHLFDGDGMVHALRI---R-----NGA------A----
-ESYACRFTET------ARLRQERAIGRP--V----FP----KAIGELHGHSGI----------ARLALFYAR---
-------AACGLVDPSAGTGVANAGLVYFN--------GR----------------------LLAMS-EDD--
LPYHVRVA-DDGDLETV--GR-------Y----------D-------------------FD--GQLGC--AM----
-------IAHPKLDPA------TGELHA------------L--SYDVIKR--------PYL---KYFYFRP-----
--------------------------------DGT---------------------KSDDVEIPLE----QPT---
----MIHDFA-ITENLVVVPDHQV--VFKLQEM----LR------G----GSPV----------------------
---VLDKEKTSR-FGVLPKH------AAD-------ASE--MAWV--------------D---V-P--
DCFCFHLWNAW----E--DEATGEVVVIGSCMTP--ADSI-----------------FNE----------------
------------------SDERLES----------------------VLTEIRLDA--------------------
--RTGRST--------------------------
---RRAVLP---

-----------------------------PSQQVNL-----------------EVGMVN-RNLLGRETRYAYLAV-
---AEPWP-------------KVSGFAKVDLSTG--------EL-------------------TK--------
FEYGEGRF--GGEPCFVPM--DP-AAAHPRGEDDG--YVLTFVHDE-------R--------AGTSEL---
LVVNAADMRL----EATVQLP--SRV-PFGFHGTF---
---ITGQELEAQAA--

>VP14_Zea_mays

MASSISAPAPPAAPATAPASGRPKKPSQFNPSTGKTRTPVPARPMRAAAPKWNPLQRLAAAALDALEEGLVAGVLE
RAHPLPR--TADPAVQI------AGNYAPV-
GERP------------PTGE--LPV------------------SGR--VPACL-DGVYVRNGAN----PLH-----
--------AP---RAGHHLFDGDGMLHAVRL---R-----GGR------A-----ESYACRFTET------
ARLRQERAIGRA--V----FP----KAIGELHGHSGV----------ARLLLFGAR----------
SLCGVLDASQGIGVANAGLVYHN--------NR----------------------LLAMS-EDD--LPYHVRVT-
ADGDLETA--GR-------Y----------D-------------------FG--GQLDT--AM-----------
IAHPKLDPA------TGELFA------------L--SYNVVSK----------P---FLKYFYF------------
------------------------TADG---------RK-----------SPDVEIPVD----APT-------
MMHDFA-VTENHAIIPDQQI--VFKLQEM----VL------G----GSPV-------------------------
VYDRNKTAR-FGVLPKR------ATD-------ASR--LRWV--------------D---V-P--

DCFCFHLWNAW----E--DEATGEVVVIGSCMTP--ADAV-----------------FNE----------------
---------------SAAGEESFRS----------------------VLSEIRLDP--------------------
--RTGTSS--------------------------
---RRAVLS---

-----------------------------DADQVNL-----------------EAGMVN-RQLLGRRTRYAYLAI-
---AEPWP-------------KVSGFAKVDLEAG--------TV-------------------DK--------
FIYGDGRY--GGEPCFVPRPDAP-AGAA---EDDG--YVLCYVHDE-------A--------RGASEM---
LVVNARDMRA----EAAVKLP--GRV-PYGLHGTF---
---IAGKELQRQA---

>NCED9_Arabidopsis_thaliana

MASTTLLPSTSTQFLDRTFSTSSSSSRPKLQSLSFSSTLRNKKLVVPCYVSSSVNKKSSVSSSLQSPTFKPPSWKK
LCNDVANLIPKTTNQNPKLNPVQRTAAMVLDAVENAMISHERR-----------------
RHPHPKTADPAVQIAGNFFPV-PEKP------------VVHN--LPV------------------TGT--VPECI-
QGVYVRNGAN----PLH-------------KP---VSGHHLFDGDGMVHAVRF---D-----NGS------V----
--SYACRFTET------NRLVQERECGRP--VFPKAIG----ELHGHLGIAKLMLFNT------RGLFGLVDP---
---------------TGGLGVANAGLVYFN--------GH----------------------LLAMS-EDD--
LPYHVKVT-QTGDLETS--GR-------Y----------D-------------------FD--GQLKS--TM----
-------IAHPKIDPE------TRELFA------------L--SYDVVSK-------------PYLKYFRF-----
--------------------------------TSD---------------------GEKSPDVEIPLD--QPT---
----MIHDFA-ITENFVVIPDQQV--VFRLPEM----IR------G----GSPV----------------------
---VYDEKKKSR-FGILNKN------AKD-------ASS--IQWI--------------E---V-P--
DCFCFHLWNSW----E--EPETDEVVVIGSCMTP--PDSI-----------------FNE----------------
------------------HDETLQS----------------------VLSEIRLNL--------------------
--KTGEST--------------------------
---RRPVIS---

-----------------------------EQ---VNL----------------EAGMVN-RNLLGRKTRYAYLAL-
---TEPWP-------------KVSGFAKVDLSTG--------EI-------------------RK--------
YIYGEGKY--GGEPLFLPS-----GDGE---EDGG--YIMVFVHDE-------E--------KVKSEL---
QLINAVNMKL----EATVTLP--SRV-PYGFHGTF---
---ISKEDLSKQALC---

>NCED3_Arabidopsis_thaliana

MASFTATAAVSGRWLGGNHTQPPLSSSQSSDLSYCSSLPMASRVTRKLNVSSALHTPPALHFPKQSSNSPAIVVKP
KAKESNTKQMNLFQRAAAAALDAAEGFLVSHEKLHPLPK-------------------TADPSVQI------
AGNFAPV--NEQ------------PV-RRNLPV------------------VGK--LPDSI-KGVYVRNGAN----
PLH-------------EP---VTGHHFFDGDGMVHAVKF---E-----HGS------A------SYACRFTQT---
---NRFVQERQLGRP--V----FP----KAIGELHGHTGI----------ARLMLFYAR----------
AAAGIVDPAHGTGVANAGLVYFN--------GR----------------------LLAMS-EDD--LPYQVQIT-
PNGDLKTV--GR-------F----------D-------------------FD--GQLES--TM-----------
IAHPKVDPE------SGELFA------------L--SYDVVSK-------------PYLKYFRF------------
-------------------------SPD---------------------GTKSPDVEIQLD--QPT-------
MMHDFA-ITENFVVVPDQQV--VFKLPEM----IRG----------GSPV-------------------------
VYDKNKVAR-FGILDKY-------AE-------DSS-NIKWI--------------------
DAPDCFCFHLWNAW----E--EPETDEVVVIGSCMTP--PDSI-----------------FNE-------------
---------------------SDENLKS----------------------VLSEIRLNL-----------------
---KTGEST-----------------------
------RRPIIS--

--------------------------------NEDQQVNL----------------EAGMVN-
RNMLGRKTKFAYLAL----AEPWP-------------KVSGFAKVDLTTG--------EV----------------
---KK--------HLYGDNRY--GGEPLFLPG-----EGGE---EDEG--YILCFVHDE-------K--------
TWKSEL---QIVNAVSLEV----EATVKLP--SRV-PYGFHGTF--------------------------------
------------IGADDLAKQVV---

>NCED5_Arabidopsis_thaliana

MACSYILTPNPTKLNLSFAPSDLDAPSPSSSVSFTNTKPRRRKLSANSVSDTPNLLNFPNYPSPNPIIPEKDTSRW
NPLQRAASAALDFAETALLRRER----------------------------SKPLPKTVDPRHQISGNYAPV-
PEKS------------VKSS--LSV------------------DGK--IPDCI-DGVYLRNGAN----PLF-----
--------EP---VSGHHLFDGDGMVHAVKI---T-----NGD------A------SYSCRFTET------
ERLVQEKQLGSP--I----FP----KAIGELHGHSGI----------ARLMLFYAR----------
GLFGLLNHKNGTGVANAGLVYFH--------DR----------------------LLAMS-EDD--LPYQVRVT-
DNGDLETI--GR-------F----------D-------------------FD--GQLSS--AM-----------
IAHPKIDPV------TKELFA------------L--SYDVVKK-------------PYLKYFKF------------
-------------------------SPE---------------------GEKSPDVEIPLA--SPT-------
MMHDFA-ITENFVVIPDQQV--VFKLSDM----FL------G----KSPV-------------------------
KYDGEKISR-FGILPRN------AKD-------ASE--MVWV--------------E---S-P--
ETFCFHLWNAW----ESPETDE--VVVIGSCMTP--ADSI-----------------FNE----------------
------------------CDEQLNS----------------------VLSEIRLNL--------------------
--KTGKSK--------------------------
---RRTIIP---


```
----------------------------------------------------------------------------
-----------------------------GSVQMNL-----------------EAGMVN-RNLLGRKTRYAYLAI-
---AEPWP-------------KVSGFAKVDLSTG--------EV-------------------KN--------
HFYGGKKY--GGEPFFLPR---GLESDG---EDDG--YIMSFVHDE-------E--------SWESEL---
HIVNAVTLEL----KATVKLP--SRV-PYGFHGTF-----------------------------------------
---VNSADMLNQA---------------------------------------------------------------
----------------------------------------------------------------------------
----------------------------------------------------------------------------
----------------------------------------------------------------------------
----------------------------------------------------------------------------
----------------------------------------------------------------------------
----------------------------------------------------------------------------
----------------------------------------------------------------------------
----------------------------
>NCED2_Arabidopsis_thaliana
----------------------------------------------------------------
MVSLLTMPMSGGIKTWPQAQIDLGFRPIKRQPKVIKCTVQIDVTELTKKRQLFTPRTTATPPQHNPLRLNIFQKAA
AIAIDAAERALISHEQDSPLPK----------------------------------------------
TADPRVQI------AGNYSPV-PESS------------VRRN--LTV------------------EGT--IPDCI-
DGVYIRNGAN----PMF-------------EP---TAGHHLFDGDGMVHAVKI---T-----NGS------A----
--SYACRFTKT------ERLVQEKRLGRP--V----FP----KAIGELHGHSGI----------ARLMLFYAR---
-------GLCGLINNQNGVGVANAGLVYFN--------NR----------------------LLAMS-EDD--
LPYQLKIT-QTGDLQTV--GR-------Y----------D-------------------FD--GQLKS--AM----
-------IAHPKLDPV------TKELHA------------L--SYDVVKK----------P---YLKYFRF-----
-------------------------------SPDG------------------------VKSPELEIPLETPT---
----MIHDFA-ITENFVVIPDQQV--VFKLGEM----ISG----------KSPV----------------------
---VFDGEKVSR-LGIMPKD-------AT-------EAS-QIIWV--------------------
NSPETFCFHLWNAW----ESPETEE--IVVIGSCMSP--ADSI-----------------FNE-------------
---------------------RDESLRS----------------------VLSEIRINL-----------------
-----------------------------------------------RTRKTT-----------------------
------RRSLLV----------------------------------------------------------------
----------------------------------------------------------------------------
----------------------------------------------------------------------------
----------------------------------------------------------------------------
----------------------------------------------------------------------------
----------------------------------------------------------------------------
--------------------------------NE--DVNL----------------EIGMVN-
RNRLGRKTRFAFLAI----AYPWP-------------KVSGFAKVDLCTG--------EM----------------
---KK--------YIYGGEKY--GGEPFFLPGNSGN-GEEN---EDDG--YIFCHVHDE-------E--------
TKTSEL---QIINAVNLKL----EATIKLP--SRV-PYGFHGTF--------------------------------
------------VDSNELVDQL------------------------------------------------------
----------------------------------------------------------------------------
----------------------------------------------------------------------------
----------------------------------------------------------------------------
----------------------------------------------------------------------------
----------------------------------------------------------------------------
----------------------------------------------------------------------------
----------------------------------------------------------------------------
-------------------------------------
>NCED4_Arabidopsis_thaliana
-------------------------------------------------------------------
MDSVSSSSFLSSTFSLHHSLLRRRSSSPTLLRINSAVVEERSPITNPSDNNDRRNKPKTLHNRTNHTLLSSPPKLR
PEMTLATALFTTVEDVINTFI--------------------------------------------
DPPSRPSVDPKHVLSDNFAPV-LDEL------------PPTD--CEI-----------------IHGS--LPPSL-
NGAYIRNGPN----PQF-------------LP---RGPYHLFDGDGMLHAIKI---H-----NGK------A----
--TLCSRYVKT------YKYNVEKQTGAP--VMPNVFSGFNGVTASVARGALTA----------ARVLTGQYN---
--------------PVNGIGLANTSLALFS--------NR----------------------LFALG-ESD--
```

LPYAVRLT-ESGDIETI--GR--------------------------------------YDFDGKLAM--SM----
-------TAHPKTDPI------TGETFA------------F--RYGPVPP--------------FLTYFRF-----
--------------------------------DSA---------------------GKKQRDVPIFSMT-SPS---
----FLHDFA-ITKRHAIFAEIQLGMRMNMLDL----VLE---------GGSPV----------------------
---GTDNGKTPR-LGVIPKY-------AG-------DES-EMKWF--------------------
EVPGFNIIHAINAW----DEDDGNSVVLIAPNIMSIE----------------------------HTLERM-----
-----------------------DLVHA----------------------LVEKVTIDL-----------------
---VTGIVT-----------------------
------R-HPIS--

--------------------------------AR----NL----------------DFAVIN-
PAFLGRRSRYVYAAI----GDPMP-------------KISGVVKLDVSKG--------DRDDCTV-----------
---AR--------RMYGSGCY--GGEPFFVAR---D-PGNPEAEEDDG--YVVTYVHDE-------V--------
TGESKF---LVMDAKSPELEI--VAAVRLP--RRV-PYGFHGLF--------------------------------
------------VKESDLNKL---

>NCED6_Arabidopsis_thaliana

MQHSLRSDLLPTKTSPRSHLLPQPKNANISRRILINPFKIPTLPDLTSPVPSPVKLKPTYPNLNLLQKLAATMLDK
IESSIVIPMEQNRPLPK--PTDPAVQL------
SGNFAPV--NEC------------PVQN-GLEV------------------VGQ--IPSCL-KGVYIRNGAN----
PMF-------------PP---LAGHHLFDGDGMIHAVSI---GF----DNQ------V------SYSCRYTKT---
---NRLVQETALGRS--V----FP----KPIGELHGHSGL----------ARLALFTAR----------
AGIGLVDGTRGMGVANAGVVFFN--------GR----------------------LLAMS-EDD--LPYQVKID-
GQGDLETI--GR-------F----------R-------------------FD--DQIDS--SVI-----------
AHPKVDAT------TGDLHT------------L--SYNVLKK----------P---HLRYLKF-------------
------------------------NTC---------GK--------KTRDVEITLP------EPT-------
MIHDFA-ITENFVVIPDQQM--VFKLSEM----IR------G----GSPV-------------------------
IYVKEKMAR-FGVLSKQ------DLT-------GSD--INWV--------------D---V-P--
DCFCFHLWNAW---EERTEEGDPVIVVIGSCMSP---PDT-----------------IFS---------------
ES------------------GEPTRV----------------------ELSEIRLNM-------------------
---RTKESN-------------------------
----RKVIVT--

------------------------------GV----NL----------------EAGHIN-
RSYVGRKSQFVYIAI----ADPWP-------------KCSGIAKVDIQNG--------TV----------------
---SE--------FNYGPSRF--GGEPCFVPE-----GEGE---EDKG--YVMGFVRDE-------E--------
KDESEF---VVVDATDMKQ----VAAVRLP--ERV-PYGFHGTF--------------------------------
------------VSENQLKEQVF---


```
----------------------------------------------------------------------------
----------------------------------------------------------------------------
----------------------------------------------------------------------------
----------------------------------------------------------------------------
-------------------------------------
>CCD1_Arabidopsis_thaliana
----------------------------------------------------------------------------
----------------------------------------------------------------------------
--------------------------------------------------------MHDASLPL---
HYLSGNFAPI-RDET------P-----PVKD--LPV------------------HGF--LPECL-NGEFVRVGPN-
---PKF------DA----------VAGYHWFDGDGMIHGVRI---K-----DGK------A------
TYVSRYVKTSRLKQ-EEFFGAAKFMKIGDLKGF-FG----LLMVNVQQLRTK----------LKILDNTYG-----
----------------NGTANTALVYHH--------GK----------------------LLALQ-EAD--
KPYVIKVL-EDGDLQTL--GI-------I----------D-------------------YD--KRLTH--SF----
-------TAHPKVDPV------TGEMFT------------F--GYSHTPP---------------YLTYRV-----
------------------------------ISKDG------------------------IMHDPVPITISEPI---
----MMHDFA-ITETYAIFMDLPM--HFRPKEM----VKE---------KKMIY----------------------
---SFDPTKKAR-FGVLPRY-------AK-------DEL-MIRWF--------------------
ELPNCFIFHNANAW----E--EED---EVVLITCRLE--NPDL---------------------------------
------------DMVSGKVKEKLENFGN----------------------ELYEMRFNM-----------------
-----------------------------------------------KTGSAS-----------------------
------Q-KKLS----------------------------------------------------------------
----------------------------------------------------------------------------
----------------------------------------------------------------------------
----------------------------------------------------------------------------
----------------------------------------------------------------------------
----------------------------------------------------------------------------
--------------------------------AS----AV----------------DFPRIN-
ECYTGKKQRYVYGTILDSIAKVTG-----------------IIKFDLHAEAETGKRMLEVGGNIK-----------
---GI--------YDLGEGRY--GSEAIYVPR-----ETAE---EDDG--YLIFFVHDE-------N--------
TGKSCV---TVIDAKTMSAEP--VAVVELP--HRV-PYGFHALF--------------------------------
------------VTEEQLQEQTLI----------------------------------------------------
----------------------------------------------------------------------------
----------------------------------------------------------------------------
----------------------------------------------------------------------------
----------------------------------------------------------------------------
----------------------------------------------------------------------------
----------------------------------------------------------------------------
----------------------------------------------------------------------------
-------------------------------------
>CCD4a_CsZCD_Crocus_sativus
----------------------------------------------------------------------------
---------
MDYRLSSSSLFHFPSPGNRIFLKQSQVLAFQNQPSHQDHPTTKKKSISINKGGSISRNRSLAAVFCDALDDLITRH
SFD--------------------------------------------PDALHPSVDPHRVLRGNFAPV--SEL---
---P-----PT-P--CRV-----------------VRGT--IPSALAGGAYIRNGPN-----PN------PQ----
-YL--PSGAHHLFEGDGMLHSLLLPSSE-----GGR------A-----AIFSSRFVET------
YKYLVTAKSRQA--I----FL----SVFSGLCGFTGI----------ARALVFFFR----------
FLTMQVDPTKGIGLANTSLQFSN--------GR----------------------LHALC-EYD--
LPYVVRLSPEDGDISTV--GR-------I----------E-----------------------NNVST--KST---
-------TAHPKTDPV------TGETFS------------F--SYGPIQP--------------YVTYSRY-----
-------------------------------DCDG---------------------KKSGPDVPIFSFK-EPS---
----FVHDFA-ITEHYAVFPDIQI--VMKPAEI----VR------G----RRMI----------------------
---GPDLEKVPR-LGLLPRY------ATS-------DSE--MRWF--------------D---V-P--
GFNMVHVVNAW----E--EEGGEVVVIVAPNVSP-----------------------IEN----------------
---------------AIDRFDLLHV----------------------SVEMARIEL--------------------
--------------------------------------------KSGSVS--------------------------
```

---R-TLLS---

-----------------------------AE---NL-----------------DFGLIH-RGYSGRKSRYAYLGV-
---GDPMP-------------KIRGVVKVDFELA--------GRGECVV--------------AR--------
REFGVGCF--GGEPFFVPA--SEGSGGE---EDDG--YVVSYLHDE-------G---------KGESSF---
VVMDARSPELEV--VAEVVLP--RRV-PYGFHGLI---
---VTEAELLSQQ---

>CCD4b_CsZCD_Crocus_sativus

MEYRLSSSLFHFPSPGNRIFLKHYPSHQDHPITKKKSISINKGGSISRNRSLAAVFCDALDDLITRHSFD------
---------------------------------------PDALHPSVDPHRVLRDNFAPV--SEL-----------
-PPTP--CRV-----------------VRGT--IPSALAGGAYIRNGPN--------------------
PNHLPSGAHHLFEGDGMLHSLLLPSSE-----GGR-----AA------IFSSRFVET------YKYLVTAKSRQA-
-IFLSVFS----GLCGFTGIARAL----------VFFFRFLTM--------------
QVDPTKGIGLANTSLQFSN--------GR----------------------LHALC-EYD--
LPYVVRLSPEDGDISTV--GR--------------------------------------IE--NNVST--KST---
-------TAHPKTDPV------TGETFS------------F--SYGPIQP--------------YVTYSRY-----
-------------------------------DCDG---------------------KKSGPDVPIFSFK-EPS---
----FVHDFA-ITEHYAVFPDIQI--VMKPAEI----VR------GRRMIGPDL----------------------
-------EKVPR-LGLLPRY-------AT-------SDS-EMRWF--------------------
DVPGFNMVHVVNAW----E--EEGGEVVVIVAPNVSP-----------------------IEN-------------
------------------AIDRFDLLHV----------------------SVEMARIEL-----------------
---KSGSVS-----------------------
------R-TLLS--

--------------------------------AE---NL-----------------DFGLIH-
RGYSGRKSRYAYLGV----GDPMP-------------KIRGVVKVDFELA--------GRGECVV-----------
---AR--------REFGVGCF--GGEPFFVPA--SEGSGGE---EDDG--YVVSYLHDE-------G--------
KGESSF---VVMDARSPELEV--VAEVVLP--RRV-PYGFHGLI--------------------------------
------------VTEAELLSQQ--

>ZCDlong_Zea_mays

```
MRFLQRIFTTRMNHNIYLSMNSIFHFLDPQVLRLYIVTYICHIRSASFYHTNLVSRFIQVYTHRSTTSCRMERTLI
TSNLSMSAHASRSSRRVHYISPAASAAAHNSSYRKKKKNAPSPPPSAAATDTVVISPPATDNVQSTAPKQAGRQEL
EELVAAKTNTSRIATARTSASSQSRAKLRRRPAPAITSLPMAFCSALEEAINTFVDPPVLRPSVDPRNVLSTNFAP
V--DEL------------PPTP--CPV-----------------VRGA--IPRCLAGGAYIRNGPN----------
----------PQHLPRGPHHLFDGDGMLHSLLL---PTAESPSSD------P------VLCSRYVQT------
YKYLVERDAGAP--VMPNVFS----GFHGVAGLARGAVVAA------RVLTGQMNP------------------
AEGVGLANTSLVFFG--------GR----------------------LYALG-ESD--LPYTVRV--
DPATGEVTTHGR-------C----------D-------------------FGGRLFMGM---------------
TAHPKKDPV------TGEVFA------------F--RYGPVPP--------------FVTYFRF------------
-------------------------DRA---------------------GNKGPDVPIFSVR-QPS-------
FLHDFA-VTERYAIFPEIQI--VMQPMGM----VTG----------GAPV-------------------------
GSDAGKVPR-LGVLPKY-------AT-------DES-EMRWF--------------------
EVPGFNIMHSLNAW----EEADGEELVLVAPNVLSLE----------------------------HALERM-----
-----------------------ELVHS----------------------CVEKVRINL-----------------
-----------------------------------------------RTGAVS-----------------------
------RTPLSA----------------------------------------------------------------
----------------------------------------------------------------------------
----------------------------------------------------------------------------
----------------------------------------------------------------------------
----------------------------------------------------------------------------
----------------------------------------------------------------------------
--------------------------------ENL---------------------DFGVIH-
PGYLGRRNRYGYLGI----GDPMP-------------KIAGVAKLDLDRA--------GTGDCTV-----------
---AR--------RVFGPGCF--AGEPFFVPD-----DVEGNSNEDDG--YLVCYVHNE-------R--------
TGENQF---VVMDARSAQLDI--VAEVQLP--ARV-PYGFHGIF--------------------------------
------------ITQAELQAQQQ-----------------------------------------------------
----------------------------------------------------------------------------
----------------------------------------------------------------------------
----------------------------------------------------------------------------
----------------------------------------------------------------------------
----------------------------------------------------------------------------
----------------------------------------------------------------------------
----------------------------------------------------------------------------
-------------------------------------
>ZCD_Zea_mays
------------------------------------------------------------------------
MERTLITSNLCLSAHASRSSRRVHYIPPSASAAAHNSSYRKKSAPSLPPSAAASATVVTAPPSTDNVQSTAPKQTG
RQELEDLVAAKTSTSRTARAPSQARARARAEPRRRPAPAATSLPMAFCSALEEAINTFVDPPALRPSVDPRNVLST
NFAPV--DEL------------PPTP--CPV-----------------VRGA--IPRCLAGGAYIRNGPN------
--------------PQHLPRGPHHLFDGDGMLHSLLL---P-----TAG------SPPSSDPVLCSRYVQT-----
-YKYLVERDAGAP--VMPNVFS----GFHGVAGLARGAVVAA------RVLTGQMNP------------------
AEGVGLANTSLAFFG--------GR----------------------LYALG-ESD--LPYTVRV--
DPATGEVTTHGR-------C----------D-------------------FGGRLFMGM---------------
TAHPKKDPV------TGEVFA------------F--RYGPVPP--------------FVTYFRF------------
-------------------------DPA---------------------GNKGPDVPIFSVQ-QPS-------
FLHDFA-VTERYAIFPEIQI--VMQPMGM----VTG----------GAPV-------------------------
GSDSGKVPR-LGVLPKY-------AT-------DES-EMRWF--------------------
EVPGFNMMHSLNAW-EEAD--GEE---LVLVAPNVLS-VEHAL---------------------------------
--ER------------------MELVHS----------------------CVEKVRMNL-----------------
-----------------------------------------------RTGAVS-----------------------
------R-TPLS----------------------------------------------------------------
----------------------------------------------------------------------------
----------------------------------------------------------------------------
----------------------------------------------------------------------------
----------------------------------------------------------------------------
----------------------------------------------------------------------------
--------------------------------AG----NL----------------DFGVIH-
```

```
PGYLGRRNRYGYLGI----GDPMP-------------KISGVAKLDLDRA--------GTGDCTV-----------
---AR--------RDFGPGCF--AGEPFFVPD-----DVEGDGNEDDG--YLVCYVHNE-------R----T---
G-ENQF---VVMDARSPQLDI--VAEVQLP--ARV-PYGFHGMF--------------------------------
------------VTQAELQAQQQ-----------------------------------------------------
----------------------------------------------------------------------------
----------------------------------------------------------------------------
----------------------------------------------------------------------------
----------------------------------------------------------------------------
----------------------------------------------------------------------------
----------------------------------------------------------------------------
----------------------------------------------------------------------------
-------------------------------------
>LSD_Aspergillus_kawachii_IFO4308
----------------------------------------------------------------------------
----------------------------------------------------------------------
MSLPKTL-------------------------------------------------------HFPDRPQF------
SAFMKPC-RVEG--------E---VN-N--LEV------------------YGD--IPNDI-DGTFFRVMPD----
--P------QM-----PP--FIENDPWFNGDGNVSAFRI---K-----NGI------V------SWQQKFVRT---
---EKFVREREAGKA--L----LG----KY-RNKFTDAVR---F--------------------------------
--KIRSTANTNIVFFN--------GQ----------------------LLALK-EDS--PPYAM----
DPSTLETK--GL-------Y---------D-------------------FE--GQLPS--VTF----------
TAHPKFDPK------TGEMVC------------F--GYEAKGD--------GTP---DVCYYQV------------
-------------------------RPD---------GS----------FSDICWFVAPVCA-----------
MIHDFA-VTENWVLFPLIPQ--LCDIDRL------------KAG--GEHW-------------------------
QWSPETPLY-IGVIPRR--------G-------PKSGDVKWL------------------RWD--
NSFPGHTLNAY----ED-HNG---HVIFDLGISK--QNVF----------------------FWWPDS--------
-------------QGSAPEPSTITS----------------------QLMRFTIDP--------------------
--------------------------------------------NTTSEI--------------------------
-----LSDP-------------------------------------------------------------------
----------------------------------------------------------------------------
----------------------------------------------------------------------------
----------------------------------------------------------------------------
----------------------------------------------------------------------------
----------------------------------------------------------------------------
-----------------------------EILHTGNS----------------EFYRID-
DRFATRKHQHCFFDLMDPSLGTDF-ETIGPRLGGGYPLYNALAHVDLQTG--------IS----------------
---EV--------YFPGQTHL--VQEPVFIPK---A-GSSI---EDGG--YLMALVNNY-------E--------
SMTSEL---HLLDLTDISKA---RAVIPLP--VRL-RPGLHGNW--------------------------------
------------VDSSDMQSV-------------------------------------------------------
----------------------------------------------------------------------------
----------------------------------------------------------------------------
----------------------------------------------------------------------------
----------------------------------------------------------------------------
----------------------------------------------------------------------------
----------------------------------------------------------------------------
----------------------------------------------------------------------------
-------------------------------------
>LSD_Aspergillus_oryzae
----------------------------------------------------------------------------
----------------------------------------------------------------MSDIKV------
--------------------------------------------------------CFPDRPQF------
SGFMKPCRVEG-------------DVSQ--LEV------------------YGE--IPKEI-DGVFYRVMPD----
PQL-------------PP--FIDNDPWFNGDGNVSAFRI---K-----DGR------A------SFRQRYVRT---
---EKFVREREAQRA--LIGK-YR--------------------------NKFTDAVEF-----------------
--KVRSTANTNVVYFN--------GQ----------------------LLALK-EDS--PPYAM----
DPVTLETK--GL--------------------------------------YDFEGQLPS--LTF----------
TAHPKFDPV------TGEMVC------------F--GYEARGD--------GTP---DVCYYSV------------
```

```
-------------------------SPT---------------------GKFTEVVWLVAP--VVA-------
MIHDFA-VTDNWVSEPVPRS--VCDIQRM------------KQG--GEHW-------------------------
QWSPETPLY-LGVISNP---------------------MQWF--------------------
QYKNSFPGHTANAY----ED-EQG---HLVIDLGLSE--KNVF----------------------FWWPDA-----
----------------QGSAPEPSSIVS----------------------QLVRFTIDP-----------------
-----------------------------------------------QSEKRA-----------------------
--------LSKP----------------------------------------------------------------
----------------------------------------------------------------------------
----------------------------------------------------------------------------
----------------------------------------------------------------------------
----------------------------------------------------------------------------
----------------------------------------------------------------------------
--------------------------------EILQHGNS----------------EFYRID-
DRFATCPYRHCYFDLMDPQLGTDL-ERIGPKLGGGFPLYNAVAHFDIVTG--------ET----------------
---EV--------YFPGDVHL--VQEPVFLPR---K-DSTT---EGDG--YILVLVNNY-------E--------
TMASEL---HLLDTRDFTRA---QAKILLP--VRLRQDYMEAGW--------------------------------
------------MEEISTHR--------------------------------------------------------
----------------------------------------------------------------------------
----------------------------------------------------------------------------
----------------------------------------------------------------------------
----------------------------------------------------------------------------
----------------------------------------------------------------------------
----------------------------------------------------------------------------
----------------------------------------------------------------------------
-------------------------------------
>LSD_Colletotrichum_gloeosporioides_Naragc5
----------------------------------------------------------------------------
------------------------------------------------------------
MAGHFRASLAAATAPPSK------------------------------------------------------
SFPDRPQF------SGFMKPC-RFEG--------E---VR-N--LEI------------------AGT--VPTEL-
DGTFYRVMPD----PQF-------------PP--FIDNDPWFNGDGNVSAFSF---H-----NGN------V----
--DFQQKYVRT------EKFVREREANRA--L----AG----KY-RNKYTDAVE---F------------------
----------------KVRTTANTNIYYFN--------KS----------------------LLAIK-EDA--
APYLM----DPVTLETK--GP-------C----------D-------------------FD--GQLPS--LTF---
-------TAHPKLDPV------TGELVC------------F--GYEAKGD--------GTP---DVCYFSI-----
--------------------------------DAD---------------------GKFNQTVWLIAPV--VG---
----MIHDFA-VTDNWVLFPMIPQ--ICDIERL------------KAG--GEHW----------------------
---QWDSNVPIY-IGVLPRR------GAQ-------GSD--VKWF--------------R---A-P--
NAFPGHTTNAY----ET-PAG---KIVFDLPLTD--KNVF----------------------FWWPDA--------
-------------EGNAPDPHDIHA----------------------EYVRFEFDP--------------------
--------------------------------------------TATEHS--------------------------
---LDLPKP-------------------------------------------------------------------
----------------------------------------------------------------------------
----------------------------------------------------------------------------
----------------------------------------------------------------------------
----------------------------------------------------------------------------
----------------------------------------------------------------------------
-----------------------------TVILKHDM----------------EFPRID-
DRFATKAHRHSWFDMMDPSLGTDF-AAIMPVLGGGHPLYNALGHLDHKTG--------KL----------------
---EV--------YYPGSTHM--VQEPVFVPR---G-EDAA---EGDG--FLVVLVNNY-------K--------
TMSSEL---HVIDTRKFTEAA---AVVKVP--MRL-RAGLHGNW--------------------------------
------------VDAKELR---------------------------------------------------------
----------------------------------------------------------------------------
----------------------------------------------------------------------------
----------------------------------------------------------------------------
----------------------------------------------------------------------------
----------------------------------------------------------------------------
```

```
----------------------------------------------------------------------------
----------------------------------------------------------------------------
-------------------------------------
>LSD_Sphingobium_sp_SYK6
----------------------------------------------------------------------------
----------------------------------------------------------------------------
-------------------------------------------------------MSFPATPDY------
TGLNKPV-GQEV------------SIKG--LKA-----------------SEGT--IPADV-RGAFFRAVPD----
PQF-------------PP--FFHPDTALSDDGMISRVLF---NA----DGT------V------DYDIRYVQT---
---PRWKAERAAGKR--L----FG-------RYRNPYTND----------PSAFDLEGT-----------------
------VSNTTPVWHA--------GK----------------------LIMTK-EDG--PGHQV----
DPHTLETI--GA--------------------------------------YDFGGVLRS--KTM----------
TAHVRVDPE------TKEMFV------------Y--GYEADGL--------CSK---TMAYWWT------------
-------------------------DRD---------------------GKLVKEQWFEAP--FCS-------
LVHDFV-ITENYAIFPLQPT--TADLERV------------KGK--GAHW-------------------------
VHQQDLDYH-VGIMPRY-------GD-------VSQ--IRWF--------------K---GPK--
GMSSFHMMNAF----ET-DDG---KVHMDHHVTD-----T----------------------IAFPHI--------
----------QADSGINVPPQMLGG----------------------GFQRWIMDM--------------------
------------------------------------------KGDGESVA--------------------------
----------------------------------------------------------------------------
----------------------------------------------------------------------------
----------------------------------------------------------------------------
----------------------------------------------------------------------------
----------------------------------------------------------------------------
----------------------------------------------------------------------------
-----------------------------VTPLGPPG----------------DMPRIA-
DADQGRPYRRGWYCSMNPQPMGPP-----VMGGVVGVMFSALLRKDFETG--------EI----------------
---VG--------YNLPPAHG--MSETVHVPA------SEP---GHEG--WLIAVVDHQ-------L--------
GPDTFEHALWIWNAGDLPAGP--VAKVPVP--TRM-RPQVHGWW--------------------------------
------------VPMADYEAARA-----------------------------------------------------
----------------------------------------------------------------------------
----------------------------------------------------------------------------
----------------------------------------------------------------------------
----------------------------------------------------------------------------
----------------------------------------------------------------------------
----------------------------------------------------------------------------
----------------------------------------------------------------------------
-------------------------------------
>SpaLSDI_Sphingomonas_paucimobilis
----------------------------------------------------------------------------
----------------------------------------------------------------------------
---------------------------------------------------MA---HFPQTPGF------
SGTLRPL-RIEG--------D---IL-D--IEI------------------EGE--VPPQL-NGTFHRVHPD----
AQF-------------PP--RFEDDQFFNGDGMVSLFRF---H-----DGK------I------DFRQRYAQT---
---DKWKVERKAGKS--L----FG----AY-RNPLTDDAS---V------QGMIR---------------------
-----GTANTNVMVHA--------GK----------------------LYAMK-EDS--PCLIM----
DPLTLETE--GY-------T----------N-------------------FD--GKLQS--QTF----------
CAHPKIDPV------TGNLCA------------F--AYGAKGL--------MTL---DMAYIEI------------
-------------------------SPT---------------------GKLLKEIPFQNP--YYC-------
MMHDFG-VTEDYAVFAVMPL--LSSWDRL------------EQR--LPFF-------------------------
GFDTTLPCY-LGILPRN-----GDA---------RD--LRWF--------------K---T-G--
NCFVGHVMNAF-------NDGT--KVHIDMPVSRNNSFPF-----------------FDV----------------
-------------HGAPFDPVAGQG----------------------FLTRWTVDM--------------------
--------------------------------------------ASNGDS--------------------------
-----FEKT-------------------------------------------------------------------
----------------------------------------------------------------------------
----------------------------------------------------------------------------
```


-----------------------------ERLFDRPD----------------EFPRID-
ERYATRAYRHGWMLILDTEKPYEA------PGGAFYALTNTLGHIDLATG--------KS----------------
---SS--------WWAGPRCA--IQEPCFIPR---S-PDAP---EGDG--YVIALVDDH-------V--------
ANYSDL---AIFDAQHVDQGP--IARAKLP--VRI-RQGLHGNW--------------------------------
------------ADASRLAVAA--

>SpaLSDIII_Sphingomonas_paucimobilis

--MAHFPDTSGM------
TGVLRPL-RIEG------------DILD--LEV------------------EGE--IPAQL-DGTFHRVHPD----
AQF-------------PP--RFEDDQFFNGDGMVSLFRF---H-----DGK------I------DFRQRYAQT---
---DKWKVERKAGKS--L----FG----AY-RNPLTDDAS---V------QGMIR---------------------
-----GTANTNVMVHA--------GK----------------------LYAMK-EDS--PCLIM----
DPLTLETE--GY-------T----------N-------------------FD--GKLKN--QTF----------
SAHAKIDPV------TGNFCN------------F--GYAATGL--------LTT---DCSYFEI------------
-------------------------DPA---------GN--------LLFETEFQVP------YYC-------
MMHDYG-LTEHYAIFHIVPC--SPNWDRL------------KAG--LPHF-------------------------
GFDTTLPVW-LGVVPRG-------PG-------VTNKDVRWF--------------K-----
APKTIFASHVMNAF-------EEGS--KIHFDTPQAE--NNAF----------------------PFFPDI-----
----------------HGAPFDPVAARP----------------------YLHRWTVDL-----------------
---GSNSED-----------------------
--------FAEV--

--------------------------------RQLTSWID----------------EFPRVD-
ARYVGQPYRHGWGLVMDPEMEMEF-----ARGRASGFKMNRIGHWDHATG--------KE----------------
---DS--------WWCGPQSI--IQEPCFVPR---M-ADSA---EGDG--YIIALVDNL-------I--------
TNYSDL---VVLDALNLKDGP--IGRAKLP--IRL-RSGLHGNW--------------------------------
------------ADASKLPIAAA---

>NOV1_LSD_Novosphingobium_aromaticivorans

---MA---QFPNTPSF------
TGFNTPS-RIEA------------DIAD--LAH------------------EGT--IPQGL-NGAFYRVQPD----
PQF-------------PP--RLDDDIAFNGDGMITRFHI---H-----DGQ------V------DFRQRWAKT---

---DKWKLENAAGKA--L----FG----AY-RNPLTDDEA---V------KGEIR---------------------
-----STANTNAFVFG--------GK----------------------LWAMK-EDS--PALVM----
DPATMETF--GF-------E----------K-------------------FG--GKMTG--QTF----------
TAHPKVDPK------TGNMVA------------I--GYAASGL--------CTD---DVTYMEV------------
-------------------------SPE---------------------GELVREVWFKVP--YYC-------
MMHDFG-ITEDYLVLHIVPS--IGSWERL----EQ------G----KPHF-------------------------
GFDTTMPVH-LGIIPRR-----DGVR-------QED--IRWF--------------T---R-D--
NCFASHVLNAW-------QEGT--KIHFVTCEAK--NNMF----------------------PFFPDV--------
-------------HGAPFNGMEAMS----------------------HPTDWVVDM--------------------
--ASNGED--------------------------
-----FAGI---

-----------------------------VKLSDTAA----------------EFPRID-
DRFTGQKTRHGWFLEMDMKRPVEL-----RGGSAGGLLMNCLFHKDFETG--------RE----------------
---QH--------WWCGPVSS--LQEPCFVPR---A-KDAP---EGDG--WIVQVCNRL-------E--------
EQRSDL---LIFDALDIEKGP--VATVNIP--IRL-RFGLHGNW--------------------------------
------------ANADEIGLAEKVLAA---

>NOV2_LSD_Novosphingobium_aromaticivorans

--MGAFPQTIYF------
TGANAPV-GEEP------------DLRG--LKV------------------EGD--LPAEV-RGSFYRAIPD----
PAF-------------PP--RFENDHTLSGDGMVSRLSF---NG----DGT------A------DFIQKYVET---
---ARYKAEKAAGKA--L----FG-------KYRNPFTDD----------PEVQGVDRT-----------------
------VANTTPVWHA--------GR----------------------MLMAK-EDG--RPYRV----
DPRTLATI--GS--------------------------------------YDFGGALKS--ETM----------
TAHVRIDAG------TGELFF------------Y--GYEADGQ--------AST---KVAYCIV------------
-------------------------GPD---------------------GELKREQWFDAP--YCA-------
MMHDFT-ISENYALFPIYPT--TADLDRL--------------KAGGEHW-------------------------
HHQPELDSW-LGVMPRY----------------GDVSEIKWF--------------K-----
GPKGCHSYHMMNAW----ED-ADG---MLHFDACLNN--TNAF----------------------A---FI-----
-------------REPSGIHMGPQDIKG----------------------ALTRWTVDP-----------------
---RADGGDVV-----------------------

--------------------------------ETVIGPPG----------------DFPVIP-
AKLQGRPYKTGWMLS-MNPELQGP----PLFAGPVGVSFNLLLRLDGMDT--------PAPQVTG-----------
---AL--------ALPPMAGF---NEPVHVPA------ADP---AKDG--WLVFLVDQQ-------V--------
GDNQFVHEAWVVDAGNIGAGA--VAKVHIP--TRL-RPQVHGWW--------------------------------
------------VPQAQLDALEGSAA--

>Blr5790_pLSD_Bradyrhizobium_diazoefficiens

--M---QHDAIAER------
RNNIAPI-PFEA------D-A---PF----LKI------------------IGE--LPREL-NGTLYRNGPN----
---------PQ-----FD---ASGAHWFVGDGMLHAFHL---E-----NGR------A------SYRNRWVRT---
---PKWLAEHDAGRA--LFGG-FG----R--KLPDAPADL------------------------------------
--TDGGVANTNIIFHA--------GK----------------------LLALE-EAH--LPTEI----
EPGTLATR--GY-------H----------N-------------------YQ--GRIAS--SF-----------
TAHPKIDPV------TGEMLF------------F--GYNAAGP--------LTP---ALSFGSI------------
-------------------------DAC---------GK----------ATRFERFEAPYAS-----------
MVHDFI-VTANHVLFPILPI--AGSMERA----MS------G----RPPY-------------------------
AWEPDKGAY-VGVMKRN-------GT-------AKD--IVWF--------------R---G-E--
ACYVFHIMNAW-------EEGN--RIVADVMQFE--EAPL-----------------FPH----------------
------------PDGRPTDPEKSRA----------------------RHCRWTFDL--------------------
---SGNTD--------------------------
-----RFQQ---

-----------------------------TYLDDLTG----------------EFPRID-
DRRAGLKSRHGWYACANPKLPMFG-------------ALSGIVHVDGNGK--------RL----------------
---GH--------YLLPAGDT--ISEPVFVER---S-SDAA---EGDG--WLLAVVWRA-------R--------
ENRSDL---AVFNATDIEAGP--AALVQLG--HRV-PDGFHGNW--------------------------------
------------VGAA--

>MtLSD_Mycobacterium_tuberculosis

MDITIVGKYLSTLP--
EDDDHPYR------TGPWRPQ-TTEW------DAD---DL----TTV------------------TGE--VPADL-
DGIYLRNTEN-------------PL-----HP--AFATYHPFDGDGMIHVVGF---R-----DGK------A----
--FYRNRFIRT------DGFLAENEAGGP--LWP-----------GLAEPVQLA----------KREHGWGAR---
-------G--------LMKDASSTDVIVHR--------GI----------------------ALTSFYQCG--
DLYRI----DPYSANTL--GK-------E----------S-------------------WH--GRFPF--DWG---
-V-----SAHPKVDNK------TGELLF------------F--NYSKQEP---------------YMRYGV-----
-------------------------------VDQN---------------------NELVHYVDVPLP--GPR---
----LPHDMA-FTENYVILNDFPL--FWDPRLL----ER-----------DVHLP---------------------
---RFYPEIPSR-FAVVARR-------G---------ND--IRWF--------------E---A-D--
PTFVLHFTNAY-------EQGD--EIVLDGFYEG--DPQP-----------------LDTGGTKWEKL--------
--------------FRFLALDRLQS----------------------RLHRWRLNM--------------------

---VTGAVHE--------------------------

-----------------------------EQLSESIT----------------EFGTIN-ADYAASSYRYTYAAT-
--GKPSWF-------------LFDGLVKHDLLTG--------NH-------------------EC--------
YSFGDGVY--GSETAMAPR---V-GSSA---EDDG--YLVTLTTDM-------N--------DDASYC---
LVFDAARPGDGP--ICKLALP--ERI-SSGTHSAW---
---VPGAELRRWDHAESPAAAVGL--

>ACO_Synechococcus_sp._PCC73109

MASIFSNKT---KSYNQTMW----
--QKGYESQ-RQEL------------AYEV--TDI------------------DGE--IPPEL-MGTLFRNGPG--
---LL------DI-----GG---TPIKHPFDGDGMVCSVSF---Q-----EGR------A------YFQNRFVQT-
-----AGFVAEQKAGQP--IYRGVFG--------TQKPGGIF----------NNMFDLRLK---------------
-------NIANTNVIYWG--------KK----------------------LLALW-EAA--EPHSL----
NPENLETR--GL------------------D-------------------YLGETLKPG--DSF----------
SAHPRIDPASIWDGGEPCFVN------------F--AVKPGLP-------------TRLVIYEI------------
-------------------------SPA---------------------GKLLRYREHEAK--GFA-------
FIHDFV-ITPNYVIFFQASV--KFNPLPY----VL------GFKGAGECV-------------------------
KFEANKPTQ-MIVIPRD-------PA-------RND-VQTLE--------------A-----E--
AGFVFHHANAF----E--RDK---QIIVDSVCYQ--SIPQ-----------------VQA----------------
----------DVNYKNVDFDALDPG----------------------QLWRFTLDL--------------------
--ETKKVT--------------------------
---R-ELLA---

-----------------------------TR----CC----------------EFPVVH-
PERVGRDYRYAYIGATHAKTGNAP--------------LQAVWKVDHQGE--------DS----------------
---QV--------YSFAPDGF--TGEPIFVPR---P-GATA---EDEG--WVLLMVYDS-------A--------
QHRSDV---VIFDAQNFAQP---LATLHLK--QHI-PYGLHGSW--------------------------------
------------TPEVFVNF--

>ACO_Synechococcus_sp._KORDI52

--MTVAPA-
---RSYDRSDW------
ASAFVNV-DEEL--------T---DVAL--TPV-------------------RGT--VPAEL-QGTFYRNGPG----
-RL------ER-----DG---HRVHHPFDGDGMIAAMRF---E-----NGR------V------QLSNRYVRT---
---EGWLAEEKAGRV--LYRGVFG----SQKPGGRLANAF-----------DLRLK---------------------
-----NIANTNVVRLG--------DQ-----------------------LLALW-EAA--EPYAL----
DPRTLETR--GL-------S----------R--------------------LD--GVLNK--GEA----F-----
SAHPRFDPG---HNGSPCMVT------------F--GVNTGPR--------------STIRLMEF------------
-------------------------ATE---------GP---------EAGQLLHERSDTFS--GFA-------
FLHDFA-ITPNWAIFLQNAI--AFNPLPF----VL------GEKGAAQCL-------------------------
RSKPDGQAK-FWLVPRD-------SG-------SFAGQPPRI---------------I-----
DAPEGFVFHHLNAW----E--DCG---DVVVESIYYS--DFPG------------------IGP-------------
-------------DEDFMDVDFDLIPEG-----------------------LLEQCRIRL-----------------
--DSGEVE-----------------------
------T-TRLS---

--------------------------------ER----CC----------------EFAMVN-
PDREGLDCRYAWMAAAAREQGNDP--------------LQVVKKLDLQTG--------ER----------------
---VI--------WDAGPRGF--VSEPLMVPD---P-STDQ---EDAG--WVLDLVWNG-------A--------
RSASDL---VILDACDLSE----QAVLELP--LAI-PHGLHGSW--------------------------------
------------APQR---

>ACO_Synechocystis_sp._PCC6803

MVTSPPTSSPSQ---RSYSPQDW----
--LRGYQSQ-PQEW------------DYWV--EDV-------------------EGS--IPPDL-QGTLYRNGPG--
---LL------EI-----GD---RPLKHPFDGDGMVTAFKF---PG----DGR------V------HFQSKFVRT-
-----QGYVEEQKAGKM--IYRGVFG---------SQPAGGW-------------LKTIFDL---------------
----RLKNIANTNITYWG--------DR-----------------------LLALW-EGG--QPHRL----
EPSNLATI--GL-------D----------D--------------------LG--GILAEGQPL-----------
SAHPRIDPASTFDGGQPCYVT------------FSIKSSLSST----------------LTLLEL------------
-------------------------DPQ----------------------GKLLRQKTETFP--GFA-------
FIHDFA-ITPHYAIFLQNNV--TLNGLPY----LF------GLRGAGECV-------------------------
QFHPDKPAQ-IILVPRD-------GG-----------EIKRI---------------------
PVQAGFVFHHANAF----E--ENG---KIILDSICYN--SLPQ------------------VDT-------------
-------------DGDFRSTNFDNLDPG-----------------------QLWRFTIDP-----------------
--AAATVE-----------------------
------K-QLMV---

--------------------------------SR----CC----------------EFPVVH-

```
PQQVGRPYRYVYMGAAHHSTGNAP--------------LQAILKVDLESG--------TE----------------
---TL--------RSFAPHGF--AGEPIFVPR---P-GGVA---EDDG--WLLCLIYKA-------D--------
LHRSEL---VILDAQDITAPA--IATLKLK--HHI-PYPLHGSW--------------------------------
-------------AQT------------------------------------------------------------
----------------------------------------------------------------------------
----------------------------------------------------------------------------
----------------------------------------------------------------------------
----------------------------------------------------------------------------
----------------------------------------------------------------------------
----------------------------------------------------------------------------
----------------------------------------------------------------------------
-------------------------------------
>RpsCCO_Rhodopseudomonas_palustris
----------------------------------------------------------------------------
----------------------------------------------------------------------------
------------------------------------------------------MLQVTGIPDA------
CDNLAPI-PMEC------------DAPF--LSI------------------KGE--LPREL-NGTLYRNGAN----
PQF-----------------VSPNAHWFFGDGMLHAFHL---E-----NGR------A------SYRNRWVRT---
---PKWLAEHEAGRP--LYGE-FN------LKLPDAPRSV------------------------------------
-PDDGNVANTNIVFHA--------GR----------------------LLALE-EAH--LPMQI----
ERDTLETR--CY-------C----------D-------------------YG--GALKG--PF-----------
TAHPKIDPV------TGEMLF------------F--GYNASGP--------LTR---TMSFGAI------------
-------------------------DAS---------GN----------VTRLEHFKAPFAA-----------
MVHDFI-VTEHHVLFPILPL--TGSIWRA----MR-----------GRPPY------------------------
AWDPRKGSY-VGVMKRS-------GS-------TRD--VRWF--------------R---G-E--
ACFVFHVMNAW-------EDGT--RIVADVMQSE--EAPL-----------------FTH----------------
------------PDGRRTDPEKGRA----------------------RLCRWSFDL--------------------
---------------------------------------------AGNTN--------------------------
-----AFKR-------------------------------------------------------------------
----------------------------------------------------------------------------
----------------------------------------------------------------------------
----------------------------------------------------------------------------
----------------------------------------------------------------------------
----------------------------------------------------------------------------
-----------------------------SYLDEISG----------------EFPRID-
ERRAGLRSGHGWYACASPETPMLG-------------MLTGLVHVDGNGH--------RR----------------
---TR--------YLLPTGDT--IGEPVFVPR---A-ADAN---EAEG--WLLAVVWRG-------C--------
ENRSDL---AVFNATDIAAGP--IALVQLG--HRI-PDGFHGNW--------------------------------
------------VPAG------------------------------------------------------------
----------------------------------------------------------------------------
----------------------------------------------------------------------------
----------------------------------------------------------------------------
----------------------------------------------------------------------------
----------------------------------------------------------------------------
----------------------------------------------------------------------------
----------------------------------------------------------------------------
-------------------------------------
>Sala_1008CCO_Sphingopyxis_alaskensis
----------------------------------------------------------------------------
-------------------------------------------------------------------
MTAQLVETIQSTL----------------------------------------------------QPNDHPYM---
---QGAWRPT-YNEW--------NAIFSNGD--AEV------------------IGT--IPDDI-DGVYVRTGEN-
------------QI-----HE--PIGRYHPFDGDGFIHAISF---K-----GGR------A------
SYRSRFVRT------KGFAAEKEAGRS--LWAGLME----PPHKSTRPGWGA----------QEWLK---------
-----------------DSSSTDVAIHA--------GK----------------------IISTFYQCG--
EAYRL----DPFTLEQF--GT------------------E-----------------------SWVPL--DGI---
-------SAHCKVDLA------TGELMF------------F--NYSKHAP---------------YMHYGV-----
```

-------------------------------VGAD---------------------NKLKHYIPVPLP--GPR---
----LPHDMA-FTEHYTILNDMPL--YWNEELL----KK-----------NLHVV---------------------
---QFHPDQKTR-FAIIPRH-------GQ-------PED--IRWF--------------E---A-E--
PTYTLHWLNAW-------EEGD--EIILDGYYQEEPMPKS-----------------YPNAPEGLERM--------
--------------MAYLDQGLLKP----------------------RLHRWRFNL--------------------
---KTGATVE--------------------------

-----------------------------ERLDDRDL----------------EFGMFN-HRYAGKPYRYAYSAI-
--PEPGWF-------------LFRGIVKHDLDSR--------TS-------------------EA--------
YEFGRGRF--GSEAPFAPR---I-GARD---EDDG--YLVSFIADL-------E--------TDQSEC---
VLIDAKNITAGP--VCRIILP--ERI-CSGTHSVW---
---ASGNDIGMGENSVLAA---

>SaCCO_Sphingopyxis_alaskensis

MASQVETLIRSAVVKTIGKVADFNRRRLPR--
PTDAHPFL------SGIHRPM-TEEL------------TIEA--LRV------------------DGE--IPAAL-
RGRYLRNGPN------P------AM-----PP--DPASYHWFIGAGMVHGIRI---E-----GGQ-----AV----
--WYRNRWVRG------------SEACAA-------LG----EE-LPPGPREER----------------------
------------------NDAPNTNVVGLA--------GR----------------------TFAIVEAGG--
TPVEL-----DHELGTIAHNP--------------------------------------FD--GTLAG--AF----
-------TAHPHADPF------TGETHA------------I--TYRSDEP---------------NKVWHV-----
--------------------------------VLD---------EQ----------AHVVREEPIAVSD-GPS---
-----IHDCA-LTENYVLVFDLPV--TFSMKRL----LA------GY---RFPY----------------------
---MWNENHPAR-VGLLPRE-------GR-------GDD--IVWV--------------P---V-D--
PCYVFHPANAF----ET-ADG---RVIVDVVAHE---TMF-----------------ATS---------------
KR-----------------GPDSEKS----------------------RMERWTIDP-------------------
---VARTTT-------------------------
----R-TVIH--

------------------------------DH----AQ----------------EFPRYD-
ERLTTRPYRYVYSIAIPDGRSAEW-----------ALADTRLFRHDLETG--------TT----------------
---AI--------HDFGSGRH--PGEFVFVPR---K-AAGA---EDDG--WLIGLVVDM-------N--------
DETTDL---VILNADDFTGPP--QAVVHLP--HRV-PPGFHGNW--------------------------------
------------VAD---


```
----------------------------------------------------------------------------
----------------------------------------------------------------------------
-------------------------------------
>PpCCO_Plesiocystis_pacifica
----------------------------------------------------------------------------
----------------------------------------------------------------------------
--------------------------------------------------------
MQAPRTDRPHAALGENLVRGH-GFED------------------LRV------------------EGE--LPKGL-
RGTLYRTGPG-----LY------ER-----FG---KRLEHPFEADAVLSAVRF---DG----EGG------A----
--RGAARVLET------PGYLEEEAAGRM--LYSS----------GAPWLRRVI----------HGLQQ-------
----------------KQKNTGNTAIWAWR--------DQ----------------------VYALL-ETA--
PPIAF----DAETLAFE--RE-------D----------D-------------------LD--GAVVR--SF----
-------SAHPTRVAK------LRTWFN------------F--GLRFGKE-------------TVLDLYAF-----
-----------------------------PERPED---------GA----------ARRIGCVDLPWPG-------
----FVHDFV-ATENYLVFFVCPG--RLAMGRA----LL------QMGSFADWF----------------------
---QWRPQDGTQ-IIVVPLA------SAD-------DPSTHRRFD--------------A-----E--
SFWVWHFVNGF----E--DRG---ELVIDYCRYP-NLDSL-----------------------------------
DA--------------IGSGRAIAPP----------------------ILHRARVNP-------------------
-------------------------------------------QGKGSRVF-------------------------
----VSEARW------------------------------------------------------------------
----------------------------------------------------------------------------
----------------------------------------------------------------------------
----------------------------------------------------------------------------
----------------------------------------------------------------------------
----------------------------------------------------------------------------
------------------------------DV----AC----------------EFPRIL-
PARSGLAHGPTWMQT-SSDPDHYG-----------------LARVALGSDADPSGDAVS-----------------
---VQ--------RWIPEAHQ-RPSEPIPVAR------------AGEGS-WVVSLCHDE-------L--------
ARASFV---AVLDGDAMEAGP--VAKVWFD--HPV-PQTFHGAW--------------------------------
------------VG--------------------------------------------------------------
----------------------------------------------------------------------------
----------------------------------------------------------------------------
----------------------------------------------------------------------------
----------------------------------------------------------------------------
----------------------------------------------------------------------------
----------------------------------------------------------------------------
----------------------------------------------------------------------------
-------------------------------------
>PPSIR1_17230_CCO_Plesiocystis_pacifica_SIR-1
----------------------------------------------------------------------------
----------------------------------------------------------------------------
--------------------------------------------------------
MAGARDFIDSDIYLNGDYEPV-RAES--------A---PGSA--LEV-----------------LEGQ--IPAGL-
RGTFARNSSN----PRF-------------EP---PRPYHWFDGDGMIHAVRF---A-----DGQ------A----
--HYRNRWVRT------AGFAEDEAAGEA--RW---TG----LL-QRPDP--------------ARLRSPEGP---
-----------------YKNTANTDLIHWQ--------GE----------------------LLALW-
WMGGGTPYRV----DASTLETK--GR--------------------------------------FE--GAGSF---
------------TAHAKLDPR------SGDLVF------------I--DYAERPP--------------
FLTHGVL-------------------------------------TPE---------GQ-------------
LTRTPVELPGSRP---------QHDIA-LTERYTVLLDVSM--FADPEAL----AR------G----KVRM-----
--------------------RFFPDQPTR-VGLFDRR--------S-------HEL--VRWF--------------
E---V-P--ACYVYHYANAW----EVREADGGEQVVIHVCRIR--DPLM-----------------YQA-------
--------MP-------GRSERTVPRIAHLRLEP----------------------ELVRWTLDL-----------
-----------------------------------------------------GTGEAT-----------------
------------E-TVVD----------------------------------------------------------
----------------------------------------------------------------------------
----------------------------------------------------------------------------
```

```
----------------------------------------------------------------------------
----------------------------------------------------------------------------
----------------------------------------------------------------------------
--------------------------------------DS----LA----------------EFPRVD-
DRLLGAPCPAVYLAM-LAERESFR--------------FSGIRRVDLRGG--------PP----------------
---VE--------RRYPEGWY--GGEVSVAPL---E-GASA---DSDADCVLVTFVGEA-------A--------
TGRSEL---WLLRADTLETVA--RLAV--P--ARV-PSGFHSHW--------------------------------
------------VPAAATDAAREAAQ--------------------------------------------------
----------------------------------------------------------------------------
----------------------------------------------------------------------------
----------------------------------------------------------------------------
----------------------------------------------------------------------------
----------------------------------------------------------------------------
----------------------------------------------------------------------------
----------------------------------------------------------------------------
-------------------------------------
>NSC1_NosCCD_Nostoc_sp._PCC_7120
----------------------------------------------------------------------------
-----------------------------------------------------------------
MVKDSLTFSSNTMT-----------------------------------------------------TTAVNPYL-
-----EGNFAPV-HEEI------------TTDT--LKV------------------IGE--LPPEL-
SGMFVRNGPN----PQW-------------TP---IGQYHWFDGDGMLHGVRI---S-----NGK------A----
--TYRNRYVRT------QRWQIEHEAGQA--IWTG----------LMEPPQTEL----------------------
----------------PSRNTGNTALIWHA--------GQ----------------------LLALW-EGG--
APYAI----QVPDLASI--GE--------------------------------------YTYNNQLSS--AF----
-------TAHPKVDPV------TGEMMF------------F--GYSFAPP---------------YLHYSV-----
-------------------------------VSAT---------------------GELVRTVPIDLPMGV-----
----MMHDFA-ITANYTIFMDLPL--TFSVERM------------QRG--EPML----------------------
---MFESDRPSR-FGILPRH----------------GDNSQIRWF--------------------
EAPSCYVFHTLNAY----E--DKD---EVVLFACPMR-STTVL-----------------ASP-------------
--DS---------------QTDPEADIP----------------------RLHRWRFHL-----------------
-----------------------------------------------KTGKVH-----------------------
------E-EMLD----------------------------------------------------------------
----------------------------------------------------------------------------
----------------------------------------------------------------------------
----------------------------------------------------------------------------
----------------------------------------------------------------------------
----------------------------------------------------------------------------
--------------------------------DV----AS----------------EFPRIN-
ENFLGQPTQYGYTSR-LAKGSIPL--------------FEGLIKYDLSNA--------KS----------------
---QN--------YEYGQGRY--GSEAVFVPR---P-GATV---EDDG--WLITYVYDT-------G--------
EESSEL---VVINAQDINSEP--IARVLLP--QRV-PYGFHGIW--------------------------------
------------VTEEQLNFRRI-----------------------------------------------------
----------------------------------------------------------------------------
----------------------------------------------------------------------------
----------------------------------------------------------------------------
----------------------------------------------------------------------------
----------------------------------------------------------------------------
----------------------------------------------------------------------------
----------------------------------------------------------------------------
-------------------------------------
>NSC2_NosACO_Nostoc_sp._PCC_7120
----------------------------------------------------------------------------
-------------------------------------------------------------MQSYKYQQKKNLE--
--------------------------------------------------------KSYTREDW------
QGGYKSL-RQEF------------DYWI--DDV------------------EGE--IPTEL-QGTLFRNGPG----
-LL------DI-----KG---QSIHHPFDGDGMISRISF---V-----NGR------A------HYRNSFVKT---
```

---EGYLAEKNAGKI--LHRGVFG----TQKPGGWLANIF----------DFKLK---------------------
-----NIANTNVIYWG--------EK----------------------LLALW-EAA--EPHRL----
DPKTLDTL--GK-EY-----------------------------------FH--GVLSA-GEAF----------
GAHPRFDPSCEQDNGAPCLVN------------F--SIKPGLS-------------TTITIFEL------------
-------------------------NVA---------------------GEVVRKHAHHVP--GFC-------
FIHDFV-ITPNHCIFFQNPV--SFNPIPF----AL------GLRGAGECI-------------------------
QVQPNQPTR-IIVIPRF-------PQ-------SGQKEIKTL--------------------
EVGSGFIFHHVNAF-VVGE--------EILVDSLCYD--SLPE-----------------VEP-------------
-------------ESDFRQVDFEAIAPG----------------------QLWRFYLNL-----------------
---KDGTVQ-----------------------
------K-KLIE--

--------------------------------SR----CC----------------EFPAIH-
PRNVGRSYRYLYMSAAHAATGNAP--------------LQALLKIDLESG--------EK----------------
---QI--------WSVAPRGF--TGEPIFVPR---P-GSEK---EDDG--WVLALVYDA-------A--------
HHRSDV---VILDASDFTKGA--IARLHLQ--HHV-PYGLHGNF--------------------------------
------------TPQVFV--

>NSC3_NosACO_Nostoc_sp._PCC_7120

---MQIVD--
--KRSNKKAW------
ASALTEP-AKEF------------PLTQ--LTI-----------------ISGN--IPEGL-RGTLYRNGPA----
-RL------ER-----GG---MLAGHWFDGDGAILAVNF---T-----DGG------V------HATYRYVQT---
---VGYQVENTADKF--LYGN-YG----M--TAPGPVWNQ---W------RKPIK---------------------
-----NAANTSVLALP--------DK----------------------LLALW-EGG--RPYAL----
DLQTLETW--GE-------D----------N-------------------LG--GLTNG--LTY----------
SAHYKQDPQ------TKEIFN------------F--GISLGLN-------------AKLNVYKS------------
-------------------------DST---------------------GKIIQKSAYSLD--GLP-------
LIHDFV-LAGKYLIFFISPV--RLNMLPV----LL------GTSNYSDSM-------------------------
QWRPELGTQ-ILVIDRE----------------SLSLVSRGE--------------T-----E--
PWYQWHFANGY-----VDASG---AVIVDIARYK----DF-----------------QTN--QYLKEV--------
--------------ATGQTHTPAPS----------------------TLSRVNLHP--------------------
--QSGKVA--------------------------
-------AI---

-----------------------------EQLLDRHC----------------EFPHVP-
RQNVGQASRYTYMSGFRQGTDISQ------------ELLNTIACFDHKTQ--------TF----------------
---IE--------AYPGENCY--PSEPILADN------------------WVLTVVYDG-------N--------
SHSSQV---WIYDSDRLHEEP--ICKLELP--KVI-PHSFHGTW--------------------------------
------------KSEKR---

>MtCCO_Mycobacterium_tuberculosis

--MTTAQA--
--AESQNPYL------
EGFLAPV-STEV------------TATD--LPV------------------TGR--IPEHL-DGRYLRNGPN----
---------PV-----AE-VDPATYHWFTGDAMVHGVAL---R-----DGK------A-----RWYRNRWVRT---
---PAVCAALGEPIS----------------ARPHPRTGI------------------------------------
---IEGGPNTNVLTHA--------GR----------------------TLALV-EAG-VVNYEL-----
TDELDTV--GP-------C----------D-------------------FD--GTLHG--GY-----------
TAHPQRDPH------TGELHA------------V--SYSFARG--------HRV---QYSVIGT------------
-------------------------DGH---------------------ARRTVDIEVAGSP-----------
MMHSFS-LTDNYVVIYDLPV--
TFDPMQVVPASVPRWLQRPARLVIQSVLGRVRIPDPIAALGNRMQGHSDRLPYAWNPSYPAR-VGVMPRE------
-GG-------NED--VRWF--------------------DIEPCYVYHPLNAY----SECRNGAE-
VLVLDVVRYS----RM-----------------FDR---------------DR---------------
RGPGGDSRP----------------------SLDRWTINL------------------------------------
----------------------------ATGAVT-----------------------------A-ECRD-------
----

-------------DR----AQ----------------EFPRIN-ETLVGGPHRFAYTVG-IEGGFLVG--------
-AGAALSTPLYKQDCVTG--------SS-------------------TV--------ASLDPDLL--
IGEMVFVPN---P-SARA---EDDG--ILMGYGWHR-------G--------RDEGQL---LLLDAQTLES----
IATVHLP--QRV-PMGFHGNW--APTT-------

>isoeugenol_monooxygenase_Pseudomonas_putida

--MAT---FDRNDPQL------
AGTMFPT-RIEA--------N---VF-D--LEI------------------EGE--IPRAI-NGSFFRNTPE----
---------PQ-----VT--TQPFHTFIDGDGLASAFHF---E-----DGQ------V------DFVSRWVCT---
---PRFEAERSARKS--L----FG----MY-RNPFTDDPS---V------EGIDR---------------------
-----TVANTSIITHH--------GK----------------------VLAAK-EDG--LPYEL----
DPQTLETR--GR-------Y----------D-------------------YK--GQVTS--HTH-----------
TAHPKFDPQ------TGEMLL------------F--GSAAKGE--------RTLDMAYYIVDRY------------
---GKVTHETWFKQP--YGA-------
FMHDFA-VTRNWSIFPIMPA--TNSLERL------------KAK--QPIY-------------------------
MWEPERGSY-IGVLPRR-------GQ-------GKD--IRWF------------------RAP--
ALWVFHVVNAW-------EEGN--RILIDLMESE-----------------------ILP--FPFPNS--------

```
-------------QNLPFDPSKAVP----------------------RLTRWEIDL--------------------
--------------------------------------------NSGNDE--------------------------
------MKR-------------------------------------------------------------------
----------------------------------------------------------------------------
----------------------------------------------------------------------------
----------------------------------------------------------------------------
----------------------------------------------------------------------------
----------------------------------------------------------------------------
-----------------------------TQLHEYFA----------------EMPIMD-
FRFALQDHRYAYMGVDDPRRPLAH------QQAEKIFAYNSLGVWDNHRK--------DY----------------
---EL--------WFTGKMSA--AQEPAFVPR---S-PDAP---EGDG--YLLSVVGRL-------D--------
EDRSDL---VILDTQCLAAGP--VATVKLP--FRL-RAALHGCW--------------------------------
------------QSKN------------------------------------------------------------
----------------------------------------------------------------------------
----------------------------------------------------------------------------
----------------------------------------------------------------------------
----------------------------------------------------------------------------
----------------------------------------------------------------------------
----------------------------------------------------------------------------
----------------------------------------------------------------------------
-------------------------------------
>isoeugenol_monooxygenase_Pseudomonas_nitroreducens
----------------------------------------------------------------------------
----------------------------------------------------------------------------
-----------------------------------------------------MARLNRNDPQL------
VGTLLPT-RIEA------------DLFD--LEV------------------DGE--IPKSI-NGTFYRNTPE----
----------------PQVTPQKFHTFIDGDGMASAFHF---E-----DGH------V------DFISRWVKT---
---ARFTAERLARKS--L----FG-------MYRNPYTDD----------TSVKGLDRT-----------------
------VANTSIISHH--------GK----------------------VLAVK-EDG--LPYEL----
DPRTLETR--GR--------------------------------------FDYDGQVTS--QTH----------
TAHPKYDPE------TGDLLF------------F--GSAAKGE--------ATP---DMAYYIV------------
-------------------------DKH---------------------GKVTHETWFEQPYGA---------
FMHDFA-ITRNWSIFPIMPA--TNSLSRL--------------KAKQPIY-------------------------
MWEPELGSY-IGVLPRR----------------GQGSQIRWL--------------------
KAPALWVFHVVNAW-------EVGT--KIYIDLMESE-----------------------ILP--FPFPNS-----
----------------QNQPFAPEKAVP----------------------RLTRWEIDL-----------------
---------------------------------------------DSSSDEIK-----------------------
-------RTRLH----------------------------------------------------------------
----------------------------------------------------------------------------
----------------------------------------------------------------------------
----------------------------------------------------------------------------
----------------------------------------------------------------------------
----------------------------------------------------------------------------
--------------------------------DF----FA----------------EMPIMD-
FRFALQCNRYGFMGVDDPRKPLAH------QQAEKIFAYNSLGIWDNHRG--------DY----------------
---DL--------WYSGEASA--AQEPAFVPR---S-PTAA---EGDG--YLLTVVGRL-------D--------
ENRSDL---VILDTQDIQSGP--VATIKLP--FRL-RAALHGCW--------------------------------
------------VPRP------------------------------------------------------------
----------------------------------------------------------------------------
----------------------------------------------------------------------------
----------------------------------------------------------------------------
----------------------------------------------------------------------------
----------------------------------------------------------------------------
----------------------------------------------------------------------------
----------------------------------------------------------------------------
-------------------------------------
>ACO_Cyanobacterium_aponinum
```

```
----------------------------------------------------------------------------
---------------------------------------------------------------------
MQQLEKPIQ------------------------------------------------------KPYSLEDW-----
-RKGYESQ-KQEL------E-Y---WI----EDI------------------EGE--IPQEL-SGTLYRNGPG---
--FL------DV-----YG---TPLQHPFDGDGMITAFTF---N-----EGK------C------FFRNRFVKT--
----KEFLEEQKAGKM--LYRGVFG----SQKPGGWLANLF----------DLKIK--------------------
------NIANTNVLHWG--------GK----------------------LLALW-EAA--HPYHL----
NPENLETV--GL-------D----------N-------------------LD--GILEA--GEV----F-----
SAHPRIDPASSFNNGKPSLVN------------F--GVKTGLS-------------STITIYEL------------
-------------------------DEK---------------------GKLLQKYSHIID--GFS-------
FIHDFV-ITPNYCIFFQNPT--TYNPFPF----LF------GLRGAGECV-------------------------
KFLDNQPTK-IILIPRN-------AP-------HKD-VITLE--------------T-----E--
AGFIFHHANAF----EVNEQ----EIVVDSICYA--KLSQ-----------------IDP----------------
----------DTSYKEVDFDKLAPG----------------------QLWRFQLNL--------------------
--------------------------------------------QDGKVT--------------------------
---KKLIDP-------------------------------------------------------------------
----------------------------------------------------------------------------
----------------------------------------------------------------------------
----------------------------------------------------------------------------
----------------------------------------------------------------------------
----------------------------------------------------------------------------
----------------------------------RCV----------------EFPSIH-
PNKVGRDYRYLFIGSAHHPTSNAP--------------LQALLKLDLQTN--------EQ----------------
---QF--------YSFAPKGF--AGEPVFVPK---A-NPQS---EDDG--WVFNLIYDS-------S--------
RHKSDL---VIFDGQDISSP---VATLHLK--QHI-PYGLHGSW--------------------------------
------------KSKS------------------------------------------------------------
----------------------------------------------------------------------------
----------------------------------------------------------------------------
----------------------------------------------------------------------------
----------------------------------------------------------------------------
----------------------------------------------------------------------------
----------------------------------------------------------------------------
----------------------------------------------------------------------------
-------------------------------------
>ACO_Microcystis_aeruginosa
----------------------------------------------------------------------------
------------------------------------------------------------------MVLTPTIGE-
--------------------------------------------------------KSYNRQDW------
QKGYQSQ-PNEY------------DYEV--EDI------------------EGQ--IPPDL-QGTVFKNGPG----
-LL------DI-----AG---TAIAHPFDGDGMISAISF---N-----HGR------V------HYRNRFVKT---
---QGYLQEQKAGKP--LYRGVFG--------TKKPGGVF----------GNAFDLRLK-----------------
-----NIANTNVIYWG--------DK----------------------LLALW-EAA--EPHRL----
DSKTLDTI--GL------------------D-------------------YL--DGILEKGDAF----------
AAHPRIDPACIFDNHQPCLVN------------F--AIKTGLS-------------SAITLYEI------------
-------------------------SPT---------------------GKLLRRHTHSIP--GFC-------
FIHDFV-ITPHYAIFFQNPV--GYNPFPF----LF------GLKGAGECV-------------------------
INQPDKLTK-IIIIPRD-------TN-------KGE--VKVL--------------------
ETTSGFVFHHSNAF----E--QGG---KIYIDSICYQ--SLPQ-----------------LDS-------------
-------------NSNFQSVDFDSLAPG----------------------HLWRFTLNL-----------------
-----------------------------------------------SENTVT-----------------------
------R-ECIL----------------------------------------------------------------
----------------------------------------------------------------------------
----------------------------------------------------------------------------
----------------------------------------------------------------------------
----------------------------------------------------------------------------
----------------------------------------------------------------------------
--------------------------------EH----CC----------------EFPSIN-
```

PAKVGRDYRYLYIAATHHATGNAP--------------LQAILKLDLSTG--------EK----------------
---QL--------HSFAPRGF--AGEPIFVPK---P-DGIA---EDDG--WLLVVTYDA-------A--------
NHRSNM---VILDAKDITNS---LAVIHLK--HHI-PYGLHGSW--------------------------------
------------TRQCF---

>ACO_Hapalosiphon_sp._MRB220

MQTFETQEQISQVST--
KPYNRNDW------QGGYKSL-KQEF------------DYWI--DDI------------------EGQ--IPPEL-
NGTLFRNGPG-----LL------DI-----NG---QFIHHPFDGDGMISRITF---A-----NGR------A----
--HFRNRFVHT------DAYLKEQKAGKL--LYRGVFG----TQKPGGWLANAF----------DFKLK-------
-------------------NIANTNVIYWG--------EK----------------------LLALW-EAA--
EPHRL----DPQTLDTL--GK--------------------------------------EYFHGVLSE-GEAF---
-------SAHPRFDPSCEFDDGEPCLVN------------F--SIKPGLS-------------TTITIFEL-----
--------------------------------DTT---------------------GTIVRKHAHSVP--GFA---
----FIHDFA-ITPNYCVFFQNPV--SFNPIPF----AL------GMRGAGECI----------------------
---KFQPNQPTR-VIIIPRR-------TPLKSPLDKGGLRGVQIL--------------------
ETHSGFVFHHANAF-------EQGN--EIIIDSICYE--SLPE-----------------VKP-------------
-------------ESDFRQTDFDALKPG----------------------QLWRFHLNL-----------------
---QDKTVR-----------------------
------R---

--------------------------------KLIESRCC----------------EFPTLH-
PQKVGRSHRYLYIGAAHSPSGNAP--------------LQAFLKMDLHSG--------ER----------------
---QL--------WSAAPKGF--ASEPIFVPR---P-HSQA---EDDG--WVLALVYDA-------S--------
CDRSDV---VILDARNFNQSP--VARLHLK--HHI-PYGLHGNF--------------------------------
------------TSEVFV--

>ACO_Leptolyngbya_valderiana

MQTYKPSERSP---LSYSREDW------
KRGYLSQ-TQES------A-Y---WI----DDI------------------EGE--IPPDL-TGTLFRVGPG----
-LL------NI-----GD---YPVNHPFDGDGMACAIAF---K-----DGR------A------FFQNRFVRT---
---QGFVEEREAGKP--LYRGVFG----TQKPGGWLANLF----------DLRLK---------------------
-----NIANTNIIYWG--------GK----------------------LLALW-EAA--EPHRL----
DPHTLETE--GL------------------D-------------------RL-DGLLDT--GDA----F-----
SAHPHIDPASQFDDGKPVLVN------------F--SVNPGLS-------------TDITIYEF------------

-------------------------APD---------------------GTLLRQHTHAIP--GFA-------
FLHDMA-ITPNYCVFFQNPV--TFNPLPY----IL------GFRGAAECV-------------------------
KFDPDAPTK-IVLIPRD-------GS-------RNVKIVETE-----------------------
PCFVFHHANAW----E--DNG---EVVVDSICYE--SFPQ-----------------PDS----------------
----------DSDFREVDFSQIPEG----------------------QLWNFRVNL--------------------
--QNENAR--------------------------
--------H---

-----------------------------EVIERRCC----------------EFPVLH-
PETVGRKNRYLFMGIAHPERGNAP--------------LQGVMKVDRETG--------TS----------------
---QV--------WSFAPRGF--GGEPAFVPR---P-GATE---EDDG--WVLLLVYDA-------E--------
KHRSKL---AIFDAKSIDAGP--IAQLHLK--YHI-PYGLHGSF--------------------------------
------------TSEYFGR---

>ACO_Scytonema_hofmannii_PCC_7110

--MQSFQLYERASTVPE-
--KSYTRANW------
QKGYQSL-KQEC------------DYWI--SDI------------------EGE--IPSEL-HGTLFRNGPG----
-LL------DI-----NG---QHIHHPFDGDGMISRVTF---I-----DGR------A------HFSNRYVRT---
---AAYLEEQKAGKI--LYRGVFG--------TQKPGGVF----------ANAFDFKLK-----------------
-----NIANTNVIYWG--------EK----------------------LLALW-EAA--DPHRL----
DPYTLETL--GK------------------E-------------------HFNGVLLEG--EAF----------
SAHPRFDPS---RDGGRRLVN------------F--SIKPGLS--------------TTITIFEL------------
-------------------------NDK---------------------GEVVTKHSHKVP--GFA-------
FIHDFA-ITPNYCIFFQNPV--TFNPLPF--------VL--GMRGAGECI-------------------------
KFQPHQPTR-IIIISRNPKQKN---------------VQIIE--------------T-----H--
SGFVFHHANAF-------EVGD--EVVVDSICYE--SLQQ-----------------VEP----------------
----------ESDFREVDFDALQPG----------------------QLWRFNLNL--------------------
--QQKTVQ--------------------------
---R-KLLE---

-----------------------------SQ----CC----------------EFPTMH-
PQKVGRPYRYLYTGAAHASSGNAP--------------LQAFLKVDLESG--------KR----------------
---QL--------WSAAPHGF--ASEPIFVPR---P-GSER---EDDG--WLLALVYDS-------T--------
HHRSDV---VILDAKDFERGA--IAKLHLK--HHV-PYGLHGNF--------------------------------
------------VSKCFL--


```
----------------------------------------------------------------------------
----------------------------------------------------------------------------
-------------------------------------
>Caenorhabditis_remanei_BCOa
----------------------------------------------------------------------------
----------------------------------------------------------------------------
----------------------------------------------------------METEGF------
ARLFHNF-ENVL--------E---PKEC--RKI-------------------GT--VPSYV-KGTMMRNGPG----
-MF------EI-----GK---DKYKHWFDGLGFMQRYHF---E-----DGKVSHAGRM------FYSARYLES---
---EAYDLNVAAQRI--VTSS-FG----TA-TFPDPCKTI---F------SKYFSTFMH-----------------
DTEKHDNANVAFTPVG--------DG----------------------LYACT-ETP--YMYRI----
DLDTLKSL--EP-------A----------D-------------------FS--KYVAL--HSC----------
TAHQLYD-E------NGDVYN------------I--GSRFGPD--------SAH---VFTVTRN------------
-------PKN---LPSE-S-----DHSW---------EH----------TLKIGEIKCTDPM-YPT-------
YMHSFG-MSENYLVMFESPV--RVNLQKF----ILR-NFI-NAT-YRDCL-------------------------
VWQEDKEVN-VFILNKK-------TG-------EQV-PLTLK--------------M-----N--
PFFTFHHANTF----E--KDG---CLVVDYCRIE--NAGS-----------------FET--LNIDNM-------
RN-------------GEFQNATTFLP----------------------YLTRVVIPL-------------------
-----SIPATAKPSDNLL-------------------GSIPWAKGYSAVLQ-------------------------
-EDGSIKLTE------------------------------------------------------------------
----------------------------------------------------------------------------
----------------------------------------------------------------------------
----------------------------------------------------------------------------
----------------------------------------------------------------------------
----------------------------------------------------------------------------
------------------------------QRTCSTSM----------------
EFPRYHWEKINMKEYKYVFGSTVFGKVEDNK---------------AGVIKADVSTG--------N----------
----------HL--------VWDRENPHQICGEPIFVPD---P-AGKE---EDDG--ILIVPIMSI------SE--
------KQPPFV---LILDAKTLKETA--RFEI--PE-ERI-PLGFHAFY--------------------------
------------------QSR-------------------------------------------------------
----------------------------------------------------------------------------
----------------------------------------------------------------------------
----------------------------------------------------------------------------
----------------------------------------------------------------------------
----------------------------------------------------------------------------
----------------------------------------------------------------------------
----------------------------------------------------------------------------
-------------------------------------------
>Caenorhabditis_remanei_BCOb
----------------------------------------------------------------------------
-------------------------------------------
MTSRGANTLRTARTKKWSTTKILRIMFATVPSHRNPPSLSISRFP-------------------------------
-------------AKMEKEGF------ARLFHNF--ENV------I-E---PK----LCS----------------
-KSGT--VPAYL-KGTMVRNGPG-----MF------KI-----GD---NEYKHWFDGLGYIQRYHF---E-----
DGK------V--SLSMYYSARYLES------EAYQKNMEAQRI--VAGS-FG----TA-AFPDPCKTI---F----
--SRFFSNFVA----------S-------DEKHDNANVAFTPVG--------DG----------------------
LYACT-ETP--HMYRV----DLDTLKTM--EP-------A----------D-------------------FS--
KYVSL--HTC----------TAHQLYD-S------NGDIYN------------I--GSRFGPD--------SAH--
-VFTVTRN-------------------PKT---AEKSIS-----DHSW---------EH----------
TSMIGEIKCSEAF-YPT-------YMHSFG-MSENYLVMFESPI--RIDIKKF----VMR-HFI-TTT-YRDCM--
-----------------------KWHEDKDVR-VFIMDKK-------TG-------GQL-KMKLK-----------
---M-----A--PFFTFHHANTF----E--KDG---CLVVDYCRME--RTGN-----------------FDA--
LLIENM--------------------KTGEFQNDPNFLP----------------------YLTRLIIPL------
------------------SIPEGAQPGDNLL-------------------KSLDWTSGCSAILQ------------
--------------ENGEIRLKE-----------------------------------------------------
----------------------------------------------------------------------------
----------------------------------------------------------------------------
```


---KRTCDISM----------------
EFPRYHWEKINMQEYKYVYGSSVLGTQKSED--------------LPGIVKADLANG--------R----------
----------HK--------VWRRENVQQVCGEPIFVPD---P-DGVE---EDDG--ILIVPVMTI------SD--
------SQRPFV---LILDARNVTEIA--RFTI--PE-ARI-PLGFHAFY--------------------------
------------------QGRPGI--

>LSD_Rco_Ustilago_maydis

MAPTATQDPVAVAVPASKAAKHGYVHP---
TDMLPSGWPTATDLSGGAQPR-RFEG------------TIFD--VMV------------------RGT--IPKEL-
YGTFYRIMPDYAEAPTY------YK-----GG----ELNAPIDGDGTVAAFRF---K-----DGK------V----
--DYRQRFVET------DRFKVERRARKS--MYGL-YR----NP-YTHHPCVRQ----------------------
----------------TVDSTANTNVVMHA--------GR----------------------FLAMK-ENG--
NAYEL----DPHTLQTL----------------------G-------------------YN--PFKLP-SKTM---
-------TAHPKQCSV------TGNLVG------------F--GYEAKGL--------ATK---DVYYFEV-----
--------------------------------DPS---------------------GKVVHELWLEAP--WCA---
----FIHDCA-LTPNYLVLMLWPF--EANLDRM------------KAG--GHHW----------------------
---AYDYDKPIT-WITIPRG-------AK-------SKDQVKCWY--------------W-----K--
NGMPIHTASGF---ED--DQG---RIIIDSSLVH-GNAFP-----------------FFP---------------
PD--------SAEQRQKQQSDGTPKA----------------------QFVRWTIDP-------------------
---RNDSNE-------------------------
----R-LPDP--

------------------------------EVILDTPS----------------EFPQID-
NRFMGVEYSSAFINV-FVPDRSDG-------NKNVFQGLNGLAHYRRKQG--------TT----------------
---EW--------YYAGDNCL--IQEPVFSPR---S-ADAP---EGDG--FVLAIVDRL-------D--------
LNRSEV---VIIDTRDFTTA---IAAVQLP--FAI-RSGIHGQW--------------------------------
------------IPGQVTPDFDSRGLIDLPKQQHWAPPSQSAFDPNM-----------------------------
----

>LSD_Rco_Aspergillus_fumigatus

--MSEMRT------
--HFPDRPQF------
SGFMKPC-RVEG------------DISQ--LEV------------------YGE--IPKEI-DGVFYRVMPD----
PQL-------------PP--FIENDPWFNGDGNVTAFRI---Q-----DGR------A------SFRQRYVRT---

---EKFMRERKAQRA--LLGK-YR--------------------------NKFTDAVEF-----------------
--RVRSTANTNVVFFN--------GQ----------------------LLALK-EDS--PPYAM----
DPITLETK--GL--------------------------------------YDFEGQLPA--LTF----------
TAHPKFDPV------TGEMVC------------F--GYEARGD--------GTP---DVCYYRV------------
------------------------SPTG---------QF----------KEVVWLVAP-----VVA-------
MIHDFA-VTDNWVVFPIIPQ--VCDIERM------------KQG--GEHW-------------------------
QWSPETPLY-LGVIPRR-------GA-------KGE-DVKWF--------------------
QYKNSFPGHTANAY----ED-KEG---HLVIDLGLSE--KNVF----------------------FWWPDA-----
----------------QGNAPEPSSIHS----------------------QLVRFTLNP-----------------
---HAEDLA-----------------------
--------LPEP--

--------------------------------KILHQGNS----------------EFYRID-
DRFATHSYRHCYFDLMDPQLGTDF-ERIRPNLGGGYPLYNSLAHFDNATG--------QT----------------
---EV--------YFPGNTHL--VQEPVFIPR---K-DSTT---EGDG--WVLALVNNY-------A--------
TMASEL---HLLDTRDFTHA---QAKILLP--IRL-RHGLHGSW--------------------------------
------------VDGRDIGSQTEQLQ--

>LSD_Rco_Chaetomium_globosum

MAHIHDLAASLAPNYTANRLTTPTPT--
RFPRTPTF------ASMNKPC-RFEG------------TVDD--LEV------------------TGT--IPADL-
DGTFFRVQPD----HRF-------------PP--LFEDDIHFNGDGAVTAVRI---E-----NGR------A----
--ALRQRYVRT------DRFEKEARAGRS--L----FG----RY-RNPWTDNES---V------KGVIR-------
-------------------TASNTNVVFWR--------GV----------------------LLAMK-EDG--
PPFAM----DPVTLETL--GR-------Y----------D-------------------FE--GQIVS--PTF---
-------TAHPKVDPR------TGEMVC------------F--AYEAGGD--------GSDCSVDVAVWTV-----
--------------------------------NGD---------------------GRKVEECWYKAPFAG-----
----MIHDCG-ISENWVVLALTPI--KMNLERM----KR------G----GNKF----------------------
---AWDPNEDQW-YGVVPRR------GAK-------SED--IIWF--------------R---A-D--
NAFHGHVAGCY----EL-PSG---EIVFDLTVAD--GNVF----------------------FFFPPD--------
--------DNITPPDGIAKRNKLSS----------------------PTTRWIFDP--------------------
-------------------------------------KAKKSAIRTLDAR--------------------------
-DADVWVAD---

-----------------------------ERVKPTLTWLTNG-----------EFSRID-
ERYNTKPYRHFWQAVVDPTKPYDF----AACGPPAGGLFNCLGHYTWSGKNYHTGESTENGASGEGEGDGKFGLE-
---DV--------YFSGPTMT--FQEPTFIPR-----EGGA---EGEG--YLIALLNHL-------D--------
QLRNDV---VIFDAQNLAKGP--VAVIHLP--LKL-KLGLHGNW--------------------------------
------------VDHRDIDAWQKRRSEDGDIGPVQVAKEPLPWQKKLAEQKGETGL--------------------
----

>LSD_Rco_CAO1_Neurospora_crassa

MAEYVFSDAPKDSHGNGVKDAVPGKQPE--
ELPPAPRYFQGENTAGFMRPV-RFEG------------DITN--LEV------------------VGE--IPKSI-
EGTFYRVMPE-PHLPSF------------------IPNDPWFNGDGNISGFYF---K-----DGH------V----
--DLKQRYVRT------EKFVREAEARRS--LLGK-YR--------------------------NRYTDLVEF---
----------------KIRSTANTNIVYWR--------GQ----------------------LLALK-EDS--
PPYAM----DPETLETF--GV-------Y----------D-------------------FD--GQLPS--LTF---
-------TAHPKFDPV------TREMVC------------F--GYEAKGD--------GTR---DICYYSF-----
--------------------------------GPD---------------------GKIAETVWLVSP--VCG---
----MIHDFA-VTENFVIFPIIPL--VCDVERM------------KQG--GDHW----------------------
---QWDYSIPMY-IGVLPRR------GAQ-------GSD--VKWF--------------E---A-P--
HGFAGHVANAF----ED-DKG---HIQLQMAYAK--DNVF----------------------FWWPDA--------
-------------NGKGPRPGEVEA----------------------HFANFVLDY--------------------
--QSDKLP--------------------------
-----LAEP---

-----------------------------TYLVDDDM----------------EFPRID-
DRVATRKHKHTFFCIFDRKPGVTDFEFVMPRAGGGAPMSNGLAHLNHETG--------DI----------------
---QR--------YLPGPRKL--TGECIFIPR---N-SEAA---EGDG--YVMVLLANY-------E--------
DMCSEL---AVLDTKDLTNE---VALIKLP--VRL-RPGLHGNW--------------------------------
------------VDKSDVDGHPAPL---

>Sea_urchin_BCOc

MEKGEIKTSTDPAINGG---
SESEKDLL------KKIYQSV-KQET------------PI-PIQTEI------------------IGT--IPSWL-
KGNLLRNGPG-----QF------EV-----GE---ESVDHLFDGLSFLHRFII---E-----GGE------V----
--KYQGRFLRS------DIYEKAMKHQRL--VFSG-FG--------------------------------------
----
------TPVDIEKL-----------V----------------------------------GVHTA-----------
----TAHPHCG-R------DGTTYN------------L--GTTFSMR----------C---KYCVIQV--------
-----------PPI---SKKNAG-----ENAM---------SG----------ASILSSIPASS-Y-NPT------
-YYHSFG-ISENYIIFVEQPL--HINLFKV----MLFSKLL-GTRPMTECM-------------------------
EYHPDSKVR-FHLIRRD-------DG-------TVI-STHYT--------------A-----D--
AFFCFHHCNAY-------ETDDGKDVVVDMCCFE--DDDV-----------------LKR--STMDNI--------
---------------RKGNLHVEDC----------------------VVRRYVLPL--------------------

--------------------------GAETKEGGDNLVTMVDSEATATLS--------------------------
PDGSIHCIP---

-----------------------------EQLCDVSL----------------
ELPQVNYADFNGFAEKCENAMSGASILSSIP-------------------ASSYNPT-------------------
--------------------YYHSFGIS--ENYIIFVEQ---P-LHIN---LFKG--VILSCVTNL-----ADD--
------KQPPFL---LVLDARTLTEIA--RAVIPLP--ISL--FGIHGIF--------------------------
------------------V---

>Lamprey_RPE65

---MATC---VEHPAKGY------
TALYETA--QEV------K-T---PM-P--TQV------------------QGR--IPSWL-SGSLMRLGPG----
-LF------EV-----GS---EEFLHLFDGQALLHKFEI---S-----NGA------V------TYACRFLKT---
---DTYMRAMTENRI--AITE-FG----TV-AYPDPCKNI---F------SRFFSYFQK----------I------
--EITDNALVNVYPVG--------ED----------------------FYACT-ETN--FITKI----
NPETLETV--AK-------V----------D-------------------LC--KYISI--NGA----------
TAHPHIER-------DGTVYN------------I--GNCFGKG--------LSF---AYNIIKI------------
-------------------------PPL---------LK--DKVNPLNKMSVAVQLPCSERF-KPS-------
YVHSFG-MTENYFVFVEQPV--KINLWKF----LSAWGPR-GAT-YMDCF-------------------------
ESHHTMGVW-VHVADKR-------RG-------EYL-DIRFR--------------A-----A--
AFNIFHHINTF----E--KEG---HIVMDVCCWK-GFEFI-----------------YNY--LYLANM--------
--------RENWEELKRNAYKAPQP----------------------EVRRYVLPI--------------------
-----NFDEEEYGKNLV--------------------RLGDTTATATLRN--------------------------
-DGTIWLEP---

-----------------------------EVLFSGPRQAF-------------EFPRINYEKNNGRDYTFAYGLG-
--LNHFVP---------------DKIYKLNVKTK--------E--------------------HW--------
VWQIPGTY--PSEPIFIAR---P-DAQD---EDDG--VLLTTVVSP------------GPAGRTPAFL---
LILNAKDLTEVA--RAEVD----VNI-PVSFHGMF---
---KESSKH---

>Ciona_BCOa

MFAIQRRPFFAVFRNFNKMS---
APSKTKSY------VKLLQKA--EER------------ANAE--CVV------------------TGC--IPEWL-
NGDVLRNGPA-----EF------DI-----GP---DTFKHWFDGHALLHKFSM---F-----EGK------V----
--TYSSKFLRS------GTYKTNHENSRI--IIGE-FG----TA-SRPDPCKNM---F------SRFFTNFVE---
--------------IAPRSDNANVSVAQLG--------EA----------------------YYAIT-DGP--
TAYGF----DPETLETK--NL--------------------------------------ITDCGPANM--TVT---
-------AAHPHYD-R------NGDYLN------------L--GTTFGRT----------P---HYHVIKV-----
--------------PAA---KM--TS-----PDPM---------NE----------LEVFMKFPSTTS--NAS---
----YHHSFG-LSENWIIFHEQPF--SFSTPKL----LIGLKLW--NP-ILSSF----------------------
----YEDKQTIS-FHIINKT-------TG-------EKI-ATKYE--------------A-----R--
GMFCFHHINAY----ETKENDGKRFIVVDMCGSD---RSL-----------------VWL--LGLDTL--------
------LDEEAHDKVVSNLDEKYLT----------------------RPRRIVIPL--------------------
----DISSDTPNDTNLV--------------------TIPGCKATAMLNK--------------------------
-SGVVSLTY---

-----------------------------ELLVPDDFPNTELGI---------ELPRINYDGYNGREYKFIYAIS-
--SEYILP---------------SHLVKINVETK--------E--------------------IK--------
YWKEKDKY--TSEPIFVPR---P-GSQD---EDDG--VVLSTVISP-------T--------DDKTFL---
LILDGQSFKEIA--RAEIE----TKM-SYPLHGLF---
---SKRD---

>Ciona_BCMOb

--MDF---PVSAFPHL------
TALATTK-NIEY------A-E---AV-Q--GKV------------------QGE--VPNWL-NGSWYRNGPG----
-VV------HF-----RE---ESVKHWFDGMALARKFCI---E-----DGK------V------SYMSRLVDG---
---ESLQKNTAAGRV--VVAE-FG----TT----THSEGF---L------GRVKSALTM-----------------
-PEFTDNCLINFMNLG--------DH----------------------LFAIT-ESN--FIRQI----
DPVTLDTK--DK-------V----------D-------------------LA--KHLPI--NIM----------
SSHPLVD-G------EGNVYT------------F--SSSIFNM--------GRT---KYNLLKF------------
-------TAAAPGTP----L----ETIL---------SQ----------SESICSIDSSWRV-SPS-------
YHHSFA-MSEKYAVFVEMPL--KIDIPKM----AVA-HLR-HMC-YSDCI-------------------------
EVLEDTKTR-IYLVNKE-------TG-------KQH-PITFL--------------C-----D--
PLIVYHHVNAY-------DDGD--HVVLDLSCYK--KNSF-----------------YDK--FTMSNL--------
--------EKTPQEFSKLFDSDEQA---------------------VKAMRIVLPL--------------------
---------------------------ANDSKTTGNLVSVANTSCTAEFQ--------------------------
-GNNIFCTS---

-----------------------------EMLSVGT-----------------ECAVIN-

NKYIGKKYKYFYSPGGLKLPPGEM-----------------LTKIDVETK--------QR----------------
---VQ--------TWQEKGCW--ASQPVFVAK---P-GATQ---EDEG--ILMSSVVNE----------------
NGNPFL---LMLDAKSFTE----VARIHFD--ANI-PPDVHGVF--------------------------------
------------VPKA--

>Lancelet_BCOa_lan

---MGV------
EQIYKTY-TEHP--------D---PV-K--AVI------------------KGN--FPKWL-SGGLVRNGPG----
-MF------DI-----GE---DKISHWFDGLGLMHRFNF---K-----DGE------V------TYRSKYLRG---
---ETYERCMKENRI--AFTG-FG----SV-HYPDPCKNI---F------RRFLSYFRM-----------------
-EEPTDNCNVNFINIG--------DD----------------------YFVCT-ETK--MMRKI----
DIETLDTK--EK-------V----------D-------------------VS--NYVTV--QVS----------
TAHPHYGLE------DGATFN------------M--GSQYGKE-------------TSYNIIKV------------
---------------------PRQKDSM---------EK----------ADILYKIPATYPR-SPS-------
YYHSFG-ITDNYIVFVEQPL--TMNLLRL----FTA-RIR-GVG-MNTCF-------------------------
DFSSTTPVR-FHLVNRE-------TA-------EHV-STKYM--------------A-----D--
SFFVFHHINAY----E--DDG---NVVVDLCAYT--DDTI-----------------MNS--LYMVDL-------
RK--------SGEEGAKRWREKSPDV----------------------FPRRYVFPI-------------------
-----NVTEDTPKGENLV--------------------KLAYTNATAVLQE-----------------------
DGS---V-HCVY--

--------------------------------DD----LV--------
STGDVPGYEAPSVNYWKYSGRKYQFAYGLC-VHDRGTVM---------------NKLVKLDVMNK--------KS-
------------------AG--------FWHEKDMY--PSEPIFIAS---P-DSKS---EDDG--VIVSAVTSV--
-----L----P---EKPGFL---LVIDAKTFTEVA--RAEV--P--IDV-PWGIHGTY------------------
--------------------------APPQNKA---

>Lancelet_BCOb

---MAAPLGS------
ARMFFSV--DEF------P-E---PV-P--TTI------------------KGE--IPAWI-SGSLMRTGPG----
-KF------EV-----GK---EAYRYWFDGLAIVHKFNI---K-----DGK------V------TYQSRFLET---
---EAYREAMKAQRI--VLSE-YG----TM-AYPDPCKNI---F------ARFFSYFFP-----------------
-PDMSDNDLVNTFPMS--------DE----------------------FYCVN-ETY--RWTKL----
DPRTLDTL--GQ-------I----------D-------------------LT--KYIAV--NAL----------
TAHPHHE-S------DGTVYN------------M--GSSYSYK----------TGC-QYNIVRF------------

--DPLDRKKCGTEFYDLSSPEA---TVL---------EN----------ASIVCSIPASYSL-SAS-------
YYHSFG-MTPNYFVFIEQPL--YMNIPKI----LLA-RIQ-DVG-VTECF-------------------------
DWYTEIPCR-FVVVRRK-------DG-------EII-STKYTDHSDTFITFTLYPSA-----D--
SFFCFHHINTY----E--EAG---HLVLDLCCFE--DARI-----------------VKL--LYLSHL--------
-------------RRPDDEKSFPEP----------------------QCRRYCLPI--------------------
----DLGQDEKVNNNTV--------------------KLTYTTATACLQQ--------------------------
-DGSVHCQP---

-----------------------------EHMSHVEKGF--------------EFPTINYTKYNGKPYRYFYGTG-
--LAGAFT---------------DALFKMDVKTK--------K--------------------LW--------
TWREKHCY--GSELIFVPS---P-DGVD---EDDG--VLLATVVDV------KD--------EKGAFL---
LVLDGKTFTELG--RAVI--PAHVGV-GYGLHGCY---
---VPEAPPGEIFQKL--

>Lancelet_BCOc_mito

MLKLLVSQRSSGRLLSAPVGRRIISLSRLNPGVSVSLFR-------------------------------------
-------TSSQHFGM------DALFKSV-EETS------------QPIQ--ADV------------------KGQ-
-IPDWL-KGSLLRVGPG-----KF------EI-----GD---QSYNHLFDGLSLIHRFNI---E-----GSK----
--V------TYQNRFLRS------DSYVLAQQQNRI--VLTE-FG----TN-SYPDPCKNI---F------
SRIFSYFWD-----------------TSGSSDNCNVNLMQVR--------DE----------------------
VYAMT-EPP--VMRRV----DPQTLETD--PE------------------K-------------------
VDRSKYVAL--NLM----------TAHPHTD-Q------DGTIYN------------M--ATRYGKD--------
G-----LFGLVRM-------------------PLPDEGER---------NNPM---------QK----------
AYMVGSIPHQVKM-RPN-------YFHSFG-LTEKYVVFVEQPM--YINVWKM----MFA-KFTSGKS-VSAAM--
-----------------------EFDGNTPAR-FHVIEKD-------TG-------KVL-PTVYL-----------
---S-----D--PFFTFHHINTY----E--EDG---QLVVDLCCNG--PGDS-----------------VAA--
LYLENL---------------------RGSEGTFDDIDT----------------------EARRYVLPL------
--QPTQATAVLQ------------
--------------EDGTVFCTP---

---ERLCPDTCTM--------------
ELPRINYDHYNGKKYQFFYGIS---------------------GQIDMLIKGDTQTK--------A----------
----------SK--------TWKEAGCY--PSEPVFVPA---P-GATA---EDEG--VVLSLVVKS----------
--SSGEDRSVFL---LVLDGQTFSEVA--RAEVPSP--AQA-CMTFHGIF--------------------------
------------------LPNM--

>Lancelet_BCOd

------------------MF----LLL------------------TGQ--IPKWL-SGSLLRNSPG-----KF---
---EQ-----GD---EKYRHWFDGMALIHKFHI---Q-----NGD------V------SYQSKFLRS------
DAYVQGLEQKRI--VMSE-FG----TT-AYPDPCKSI---F------SRMFSYFTP----------------
MSRPRTDNGNVHLMQVG--------EE----------------------YYAHT-ELP--YIRKV----
DPRTLDSGKLKV------------------D-------------------YQ--KYVAV--NGA----------
TAHAQID-V------DGTVYN------------M--GTTYGKD--------G-----GYSLIKI------------
-------PLPDKGEV---------ENPL---------QK----------ASIIAKIPQKYGA-FPN-------
YFHSFA-MTENYFVFVEQPF--FLNVLKI----MAG-PLF-GKG-VDWAF-------------------------
QFHKEIPTQ-FHVIEKA-------TG-------KVW-TTKYT--------------A-----D--
AMMTFHHINAY----E--DDG---HLVMDLCAFA--KMDA-----------------VFQ--FYLHNL--------
--------HTWSKEEADKKLGDTDN----------------------YIARFVLPL--------------------
----DVSQDGPDDENLV--------------------KLSDTLASAIRKQ--------------------------
--DSIYCVP---

-----------------------------EILTDANF----------------DLPRVN-EKYNGRKYRYIYAVD-
-VYRTPFR-----------------LVKVDAETK--------E--------------------NK--------
YWTEENCY--AAEPVFVEA---P-NPTS---EDDG--VVLSAVVRV-------GKG------KSTCFL---
LVLDGKTFTELG--RAELSQP--NKV-PMQTHGIY---
---VANM---

>Human_BCMO1

---M------
DIIFGRN-RKEQ------L-E---PV-R--AKV------------------TGK--IPAWL-QGTLLRNGPG----
-MH------TV-----GE---SRYNHWFDGLALLHSFTI---R-----DGE------V------YYRSKYLRS---
---DTYNTNIEANRI--VVSE-FG----TM-AYPDPCKNI---F------SKAFSYLSH----------T------
IPDFTDNCLINIMKCG--------ED----------------------FYATS-ETN--YIRKI----
NPQTLETL--EK-------V----------D-------------------YR--KYVAV--NLA----------
TSHPHYD-E------AGNVLN------------M--GTSIVEK--------GKT---KYVIFKI------------
-------PATVPEGK--KQG----KSPW---------KH----------TEVFCSIPSRSLL-SPS-------
YYHSFG-VTENYVIFLEQPF--RLDILKM----ATA-YIR-RMS-WASCL-------------------------
AFHREEKTY-IHIIDQR-------TR-------QPV-QTKFY--------------T-----D--
AMVVFHHVNAY----E--EDG---CIVFDVIAYE-D-NSL-----------------YQL--FYLANL-------
NQ-------DFKEN-----SRLTSVP----------------------TLRRFAVPL-------------------
-----HVDKNAEVGTNLI--------------------KVASTTATALKEE-----------------------
DGQ---V-YCQP--

--------------------------------EF----LY---------EGL----ELPRVN-
YAHNGKQYRYVFATG-VQWSPIPT----------------KIIKYDILTK--------S-----------------
---SL--------KWREDDCW--PAEPLFVPA---P-GAKD---EDDG--VILSAIVST-------D----P---
QKLPFL---LILDAKSFTELA--RASV--D--VDM-HMDLHGLF--------------------------------
------------ITDMDWDTKKQAASEEQRDRASDC-------------------HGAPLT---------------
----

>Dog_BCMO1

---M------
DIIFGRN-KKEQ------L-E---PV-R--AKV------------------TGR--IPTWL-QGTLLRNGPG----
-MH------TV-----GE---TRYNHWFDGLALLHSFTI---R-----DGE------V------YYRSKYLRS---
---DTYKDNIEANRI--VVSE-FG----TM-AYPDPCKNI---F------SKAFSYLSH----------T------
IPDFTDNCLINIMKCG--------ED----------------------FYATT-ETN--YIRKI----
NPHTLETL--EK-------V----------D-------------------YR--KFVAV--NLA----------
TAHPHYD-E------AGNVLN------------M--GTSIMDK--------GKT---KYVIFKI------------
-------PATVPEDK--KE-----KNPL---------KH----------TEVFCSITSRSLL-SPS-------
YYHSFG-VTENYVIFLEQPF--KLDILKM----STA-YIR-GVN-WASCM-------------------------
AFHKEDKTY-IHIIDQR-------TR-------KPV-PTKFY--------------T-----D--
PMVVFHHVNAY----E--EDG---CLLFDVIAYE-D-SSL-----------------YQL--FYLANL-------
NQ-------DFEEN-----SRLTSIP----------------------TLRRFAVPL-------------------
-----NVDKNAEVGSNLI--------------------KLASTTARALKEK-----------------------
DDQ---V-YCQP--

--------------------------------EL----LY---------EGL----ELPRIN-
YAHNGKRYRYIFAAE-VQWSPIPT----------------KILKYDVLTK--------S-----------------
---SL--------KWGQEHCW--PAEPLFVPT---P-GAKD---EDDG--IILSAIVST-------D----P---
QKLPFL---LILDAKSFTELA--RASI--D--VEM-HLDLHGLF--------------------------------
------------IPDTDCNTGKQAPSQEEQDRAPDC-------------------HAAPQI---------------
----

>Cow_BCMO1

---M------
EIIFGRN-KKEQ------L-E---PV-R--ARV------------------TGK--IPAWL-QGILLRNGPG----
-MH------TV-----GE---TRYNHWFDGLALLHSFTI---R-----DGE------V------YYRSKYLRS---

```
---DTYTANIEANRI--VVSE-FG----TM-AYPDPCKNI---F------SKAFSYLSH----------T------
IPDFTDNCLINIRRCG--------ED----------------------FYATT-ETS--YIRRI----
NPQTLETL--EK-------V----------D-------------------FR--KYVAV--NLA----------
TSHPHYD-A------AGNVLN------------V--GTSIVDK--------GKT---KYVIFKI------------
-------PAPVPGGR--KEG----RSPL---------KD----------TEVFCSIAAHSLL-SPS-------
YYHSFG-VSENYIIFLEQPF--KLDILKM----ATA-YIR-GVS-WASCL-------------------------
AFHGEDKTH-IHIIDRR-------TR-------KPV-PTKYH--------------T-----D--
PMVVFHHVNAY----E--EDG---CLLFDVITYE--DGSL-----------------YQL--FYLANL--------
-----------NEDFKENSRLTSMP----------------------TLKRFVLPL--------------------
----HVDKNAEVGSNLI--------------------KLSSTTARALKEK--------------------------
-DDQVYCQP-------------------------------------------------------------------
----------------------------------------------------------------------------
----------------------------------------------------------------------------
----------------------------------------------------------------------------
----------------------------------------------------------------------------
----------------------------------------------------------------------------
-----------------------------ELLCEGL-----------------ELPHIN-
YAHNGQPYRYIFAAGVQWSPRPLI-----------------YAAIRLAKS--------------------------
---SL--------TWKEEHCW--PAEPLFVPT---P-GAKD---EDDG--IILSAIVST-------DP-------
QKSPFL---LVLDARTFTELA--RASVD----VEM-HLDFHGLF--------------------------------
------------
IPDAGRDPGKQAPSQEAPARAAAGRAAPRTDSLEALVLGTSSAQLTAVPAPGEGRESGPSFHFAHILSAAALSQNS
ETET------------------------------------------------------------------------
----------------------------------------------------------------------------
----------------------------------------------------------------------------
----------------------------------------------------------------------------
----------------------------------------------------------------------------
----------------------------------------------------------------------------
----------------------------------------------------------------------------
-------------------------
>Mouse_BCMO1
----------------------------------------------------------------------------
----------------------------------------------------------------------------
---------------------------------------------------------------M------
EIIFGQN-KKEQ------L-E---PV-Q--AKV------------------TGS--IPAWL-QGTLLRNGPG----
-MH------TV-----GE---SKYNHWFDGLALLHSFSI---R-----DGE------V------FYRSKYLQS---
---DTYIANIEANRI--VVSE-FG----TM-AYPDPCKNI---F------SKAFSYLSH----------T------
IPDFTDNCLINIMKCG--------ED----------------------FYATT-ETN--YIRKI----
DPQTLETL--EK-------V----------D-------------------YR--KYVAV--NLA----------
TSHPHYD-E------AGNVLN------------M--GTSVVDK--------GRT---KYVIFKI------------
-------PATVPDSK--KKG----KSPV---------KH----------AEVFCSISSRSLL-SPS-------
YYHSFG-VTENYVVFLEQPF--KLDILKM----ATA-YMR-GVS-WASCM-------------------------
SFDREDKTY-IHIIDQR-------TR-------KPV-PTKFY--------------T-----D--
PMVVFHHVNAY----E--EDG---CVLFDVIAYE-D-SSL-----------------YQL--FYLANL-------
NK-------DFEEK-----SRLTSVP----------------------TLRRFAVPL-------------------
-----HVDKDAEVGSNLV--------------------KVSSTTATALKEK-----------------------
DGH---V-YCQP----------------------------------------------------------------
----------------------------------------------------------------------------
----------------------------------------------------------------------------
----------------------------------------------------------------------------
----------------------------------------------------------------------------
----------------------------------------------------------------------------
--------------------------------EV----LY---------EGL----ELPRIN-
YAYNGKPYRYIFAAE-VQWSPVPT----------------KILKYDILTK--------S-----------------
---SL--------KWSEESCW--PAEPLFVPT---P-GAKD---EDDG--VILSAIVST-------D----P---
QKLPFL---LILDAKSFTELA--RASV--D--ADM-HLDLHGLF--------------------------------
------------IPDADWNAVKQTPAETQEVENSDHPTDPTAPELSHSENDFTAGHGGSSL---------------
```


>Rat_BCMO1

---M------
EIIFGRN-KKEQ------L-E---PL-R--ATV------------------TGS--IPAWL-QGTLLRNGPG----
-MH------TV-----GD---SKYNHWFDGLALLHSFSI---R-----DGE------V------FYRSKYLQS---
---DTYNANIEANRI--VVSE-FG----TM-AYPDPCKNI---F------SKAFSYLSH----------T------
IPDFTDNCLINIMKCG--------ED----------------------FYATT-ETN--YIRKI----
DPQTLETL--EK-------V----------D-------------------YR--KYVAV--NLA----------
TSHPHYD-E------AGNVLN------------M--GTSIADK--------GGT---KYVMFKI------------
-------PATAPGSK--KKG----KNPL---------KH----------SEVFCSIPSRSLL-SPS-------
YYHSFG-VTENYVVFLEQPF--KLDILKM----ATA-YMR-GVS-WASCM-------------------------
TFCKEDKTY-IHIIDQK-------TR-------KPV-PTKFY--------------T-----D--
PMVVFHHVNAY----E--EDG---CVLFDVIAYE-D-NSL-----------------YQL--FYLANL-------
NK-------DFEEK-----SRLTSVP----------------------TLRRFAVPL-------------------
-----HVDKDAEVGSNLV--------------------KVSSTTATALKEK-----------------------
DDH---V-YCQP--

--------------------------------EV----LY---------EGL----ELPRIN-
YAHNGKPYRYIFAAE-VQWSPVPT----------------KILKYDVLTK--------S-----------------
---SL--------KWSEESCW--PAEPLFVPT---P-GAKD---EDDG--VILSAIIST-------D----P---
QKLPFL---LILDAKSFTELA--RASV--D--VDM-HLDLHGLF--------------------------------
------------IPDAGWNAVKQTPAKTQEDENSDHPTGLTAPGLGHGENDFTAGHGGKSL---------------
----

>Chicken_BCMO1

---M------
ETIFNRN-KEEH------P-E---PI-K--AEV------------------QGQ--LPTWL-QGVLLRNGPG----
-MH------TI-----GD---TKYNHWFDGLALLHSFTF---K-----NGE------V------YYRSKYLRS---
---DTYNCNIEANRI--VVSE-FG----TM-AYPDPCKNI---F------AKAFSYLSH----------T------
IPEFTDNCLINIMKTG--------DD----------------------YYATS-ETN--FIRKI----
DPQTLETL--DK-------V----------D-------------------YS--KYVAV--NLA----------
TSHPHYD-S------AGNILN------------M--GTSIVDK--------GRT---KYVLFKI------------
-------PSSVPEKE--KK-----KSCF---------KH----------LEVVCSIPSRSLL-QPS-------
YYHSFG-ITENYIVFIEQPF--KLDIVKL----ATA-YIR-GVN-WASCL-------------------------
SFHKEDKTW-FHFVDRK-------TK-------KEV-STKFY--------------T-----D--
ALVLYHHINAY----E--EDG---HVVFDIVAYR--DNSL-----------------YDM--FYLKKL--------

```
-----------DKDFEVNNKLTSIP----------------------TCKRFVVPL--------------------
----QYDKDAEVGSNLV--------------------KLPTSATAVKEKD--------------------------
--GSIYCQP-------------------------------------------------------------------
----------------------------------------------------------------------------
----------------------------------------------------------------------------
----------------------------------------------------------------------------
----------------------------------------------------------------------------
----------------------------------------------------------------------------
-----------------------------EI----LCEGI-------------ELPRVN-
YDYNGKKYKYVYATEVQWSPVPTK-----------------IAKLNVQTK--------E-----------------
---VL--------HWGEDHCW--PSEPIFVPS---P-DARE---EDEG--VVLTCVVVS-------EP-------
NKAPFL---LILDAKTFKELG--RATVN----VEM-HLDLHGMF--------------------------------
------------IPQNDLGAETE-----------------------------------------------------
----------------------------------------------------------------------------
----------------------------------------------------------------------------
----------------------------------------------------------------------------
----------------------------------------------------------------------------
----------------------------------------------------------------------------
----------------------------------------------------------------------------
----------------------------------------------------------------------------
-------------------------------------
>Zebrafish_BCMO1
----------------------------------------------------------------------------
----------------------------------------------------------------------------
--------------------------------------------------------------ML------
SFFWRNG--IET------P-E---PL-K--ADV------------------SGS--IPPWL-QGTLLRNGPG----
-LF------SV-----GN---TSYKHWFDGMALIHSFTF---K-----DGE------V------FYRSKYLKS---
---ETYKKNIAADRI--VVSE-FG----TM-VYPDPCKNI---F------SRAFSYMMN----------A------
IPDFTDNNLINIIKYG--------ED----------------------YYASS-EVN--YINQI----
DPLTLETL--GR-------T----------N-------------------YR--NHIAI--NLA----------
TAHPHYD-E------EGNTYN------------M--GTAIMNL--------GRP---KYVIFKV------------
-------PANTS-DK--ENK----KPAL---------SE----------VEQVCSIPIRPSL-YPS-------
YFHSFG-MTENYIIFVEQAF--KLDIVKL----ATA-YFR-DIN-WGSCL-------------------------
KFDQDDINV-FHLVNKK-------TG-------KAV-SVKYY--------------T-----D--
PFVTFHHINAY----E--DDG---HVVFDLITYK--DSKL-----------------YDM--FYIQNM--------
--------KQDVKRFIETNKDFAQP----------------------VCQRFVLPV--------------------
----NVDKETPQDINLV--------------------KLQDTTATAVLKE--------------------------
-DGSVYCTP-------------------------------------------------------------------
----------------------------------------------------------------------------
----------------------------------------------------------------------------
----------------------------------------------------------------------------
----------------------------------------------------------------------------
----------------------------------------------------------------------------
-----------------------------DIIFKGL-----------------ELPAIN-
YKFNSKKNRYFYGTRVEWSPYPNK-----------------VAKVDVVTR--------T-----------------
---HK--------IWTEEECY--PSEPVFIAS---P-DAVD---EDDG--VILSSVVSF-------NP-------
QRPPFL---VVLDAKSFKEIA--RATI--D--ASI-HMDLHGLF--------------------------------
------------IHDKST----------------------------------------------------------
----------------------------------------------------------------------------
----------------------------------------------------------------------------
----------------------------------------------------------------------------
----------------------------------------------------------------------------
----------------------------------------------------------------------------
----------------------------------------------------------------------------
----------------------------------------------------------------------------
-------------------------------------
>Human_BCO2
```


MFFRVFLHFIRSHSATAVDFLPVMVHRLPVFKRYMGNTPQKKAVFG------------------------------
--------------QCRGLPCV------APLLTTV--EEA------P-R---GI-S--ARV---------------
---WGH--FPKWL-NGSLLRIGPG-----KF------EF-----GK---DKYNHWFDGMALLHQFRM---A-----
KGT------V------TYRSKFLQS------DTYKANSAKNRI--VISE-FG----TL-ALPDPCKNV---F----
--ERFMSRFEL----------P----GKAAAMTDNTNVNYVRYK--------GD----------------------
YYLCT-ETN--FMNKV----DIETLEKT--EK-------V----------D-------------------WS--
KFIAV--NGA----------TAHPHYDL-------DGTAYN------------M--GNSFGPY--------GFS--
--YKVIRV-------------------------------------PPE---------KV--
DLGETIHGVQVICSIASTEKG-KPS-------YYHSFG-MTRNYIIFIEQPL--KMNLWKI----ATS-KIR-
GKA-FSDGI-------------------------SWEPQCNTR-FHVVEKR-------TG-------QLL-
PGRYY--------------S-----K--PFVTFHQINAF----E--DQG---CVIIDLCCQD--NGRT--------
---------LEV--YQLQNL----------------RKAGEGLDQVHNSAAKS----------------------
FPRRFVLPL------------------------NVSLNAPEGDNLS--------------------
PLSYTSASAVKQA-----------------------DGT---I-WCSH----------------------------
----

ENLHQEDL------EKEGGI----EFPQIYYDRFSGKKYHFFYGCG---FRHLVG---------------
DSLIKVDVVNK--------T--------------------LK--------VWREDGFY--PSEPVFVPA---P-
GTNE---EDGG--VILSVVITP-------N----Q---NESNFI---LVLDAKNFEELG--RAEV--P--VQM-
PYGFHGTF--IPI---------------------

-
>Dog_BCO2

MFSQTFLHFVSNLSTIAVGFLSVVVHWIPVLKKYMEGTHQKKAVFH------------------------------
--------------KRERLPCI------APLLTTV-EEMP------------QMVS--AQV---------------
---RGQ--FPEWL-SGYLLRIGPG-----KF------EF-----GK---DEYSHWFDGMALLHQFRM---E-----
KGT------V------TYRSKFLQS------DTYKANSAHDRI--VISE-FG----TL-ALPDPCKNV---F----
--ERFMSKFEL------------------PAITDNTNVNYVQYK--------GD----------------------
YYVST-ETN--FMNKV----DIKTLEKT--EK-------V----------D-------------------WS--
KFIAV--NGA----------TAHPHYDP-------DGTAYN------------M--GNSYGPH--------GSC--
--YNVIQI-------------------------------------PPE---------KV--
DLGETVHGAQVICSIASAERM-KPS-------YYHSFG-MTRNYIIFIEQPL--KMNLWKI----VTS-RIR-
GKA-FSDGI-------------------------SWEPQYNTR-FHVVDKH-------TG-------QLL-
PGMYY--------------T-----K--PFVTFHQINAF----E--DQG---CVVIDLCCQD--DGRS--------
---------LEV--YQLQNL----------------RKAGEGLDQVYNSVGRS----------------------
FPRRFVLPL------------------------HVSLNDPEGENLS--------------------
PLSYSSASAVKHA-----------------------DGK---I-WCAY----------------------------
----

ENLHPEDL------EEEGGV----EFPQINYGQFSGKKYRFFYGCG---FRHLVG---------------
DSLIKVDVVNK--------T--------------------LT--------IWREDGFY--PSEPVFVPA---P-
GTRE---EDGG--VILSVVITP-------D----Q---NENNFL---LVLDAKNFEELG--RAEV--P--VRM-
PYGFHGTF--VAT---------------------

-
>Cow_BCO2

MILRRIPVLKKYMEKTHQKTVIFG--
QEKTLPCI------APLLTTV-EETP------------QVIS--AGV------------------QGH--FPEWL-
SGYLLRVGPG-----KF------EF-----GK---DKYNHWFDGMALLHQFKV---E-----KGT------V----
--TYRSKFLQS------DTYKANSDRDRI--VISE-FG----TL-ALPDPCKNV---F------ERFMSKFEK---
---------------PAITDNTNVNYVLYK--------GD----------------------YYLST-ETN--
FMNKV----DIETLEKT--EK-------V----------N-------------------WT--KFIAV--NGA---
-------TAHPHYDP-------DGTTYN------------M--GNSYGKH--------GSC----YNVIRV-----
--------------------------------PPE---------KS--DPGETIHGAQVICSIASEEGM-RPS---
----YYHSFG-MTRNYIIFIEQPL--KINLWRI----ISS-KIR-GKA-FSDGI----------------------
---SWEPQYNTR-FHVVDKH-------TG-------QLL-PGMYF--------------S-----K--
PFVTFHQINAF----E--DQG---CVVIDLCCQD--DGGI-----------------LEV--YQLQNL--------
--------RKTGKELDQVYNLIARN----------------------SPRRFVLPL--------------------
----LGNLNAPEGENLS--------------------PLTYSSASAVKQA-----------------------
DGK---I-WCSY--

--------------------------------ENLYPEDL------KEEGSI----
EFPQINYGQFNGKKYQFFYGCG---FRHLVG---------------DSLIKVDVVNK--------T----------
----------RR--------VWREDGFY--PSEPVFVPV---P-GASK---EDDG--VILSVVITP-------N--
--Q---NKKKFS---SCLGCQEL---

>Mouse_BCO2

---MLGPKQSLPCI------
APLLTTA-EETL------------SAVS--ARV------------------RGH--IPEWL-NGYLLRVGPG----
-KF------EF-----GK---DRYNHWFDGMALLHQFRM---E-----RGT------V------TYKSKFLQS---
---DTYKANSAGGRI--VISE-FG----TL-ALPDPCKSI---F------ERFMSRFEP-----------------
-PTMTDNTNVNFVQYK--------GD----------------------YYMST-ETN--FMNKV----

```
DIEMLERT--EK-------V----------D-------------------WS--KFIAV--NGA----------
TAHPHYDP-------DGTAYN------------M--GNSYGPR--------GSC----YNIIRV------------
-------------------------PPK---------KK--EPGETIHGAQVLCSIASTEKM-KPS-------
YYHSFG-MTKNYIIFVEQPV--KMKLWKI----ITS-KIR-GKP-FADGI-------------------------
SWEPQYNTR-FHVVDKH-------TG-------QLL-PGMYY--------------S-----M--
PFLTYHQINAF----E--DQG---CIVIDLCCQD--DGRS-----------------LDL--YQLQNL--------
--------RKAGEGLDQVYELKAKS----------------------FPRRFVLPL--------------------
----DVSVDAAEGKNLS--------------------PLSYSSASAVKQG-----------------------
DGE---I-WCSP----------------------------------------------------------------
----------------------------------------------------------------------------
----------------------------------------------------------------------------
----------------------------------------------------------------------------
----------------------------------------------------------------------------
----------------------------------------------------------------------------
--------------------------------ENLHHEDL------EEEGGI----
EFPQINYGRFNGKKYSFFYGCG---FRHLVG---------------DSLIKVDVTNK--------T----------
----------LR--------VWREEGFY--PSEPVFVPV---P-GADE---EDSG--VILSVVITP-------N--
--Q---SESNFL---LVLDAKSFTELG--RAEV--P--VQM-PYGFHGTF--------------------------
------------------VPI-------------------------------------------------------
----------------------------------------------------------------------------
----------------------------------------------------------------------------
----------------------------------------------------------------------------
----------------------------------------------------------------------------
----------------------------------------------------------------------------
----------------------------------------------------------------------------
----------------------------------------------------------------------------
-------------------------------------------
>Rat_BCO2
----------------------------------------------------------------------------
----------------------------------------------------------------------------
-----------------------------------------------------MLGPQQSLPCI------
APLLTTV-EETL------------STVS--ARV------------------RGH--IPEWL-NGYLLRVGPG----
-KF------EF-----GK---DRYNHWFDGMALLHQFKM---E-----KGT------V------TYKSKFLQS---
---DTYKANSAGDRI--VISE-FG----TL-ALPDPCKSI---F------ERFMSRFEP-----------------
-PTMTDNTSVNFVQYK--------GD----------------------YYMST-ETN--FMNKV----
DIETLERT--EK-------V----------D-------------------WS--KFVAV--NGA----------
TAHPHYDP-------DGTAYN------------M--GNTYGPR--------GSC----YNIIRV------------
-------------------------PPK---------KK--EPGETIHGAQVVCSIASSEKM-KPS-------
YYHSFG-MTKNYIVFVEQPL--KMKLWKI----ITS-KIR-GKS-FADGI-------------------------
SWEPQYNTR-FHVVDKH-------TG-------QPL-PGVYY--------------S-----K--
PFLTYHQINAF----E--DQG---CIVIDLCCED--DGRS-----------------LDI--YQLQNL--------
--------RKAGEELDQVYKAKAKS----------------------FPRRFVLPL--------------------
----DISVGAPEGENLR--------------------PLPYSSASVVKQG-----------------------
DRE---I-WCSP----------------------------------------------------------------
----------------------------------------------------------------------------
----------------------------------------------------------------------------
----------------------------------------------------------------------------
----------------------------------------------------------------------------
----------------------------------------------------------------------------
--------------------------------ENLHQEDL------EEEGGI----
EFPQINYGRFSGKKYSFFYGCG---FRHLVG---------------DSLIKVDVVNK--------T----------
----------LR--------VWREEGCY--PSEPVFVPV---P-GADE---EDSG--AILSVVITP-------N--
--Q---GESNFL---LVLDAKNFTELG--RAEV--P--VRM-PYGFHGTF--------------------------
------------------VPI-------------------------------------------------------
----------------------------------------------------------------------------
----------------------------------------------------------------------------
----------------------------------------------------------------------------
```


>Chicken_BCO2

MPSSEQRMFSKIALSAVSILLANLRHLLSSLMQFVPGLKNSLPSPLEEQF--------------------------
------------------TLPTLQCI------SPLLQTV--EET------P-E---PI-P--AKI-----------
-------KGH--IPGWI-NGNLLRNGPG-----KF------EF-----GE---EKYNHWFDGMALLHQFQL---R-
----NGT------V------TYQSKFLQS------NSYLINNQHNRI--VVSE-FG----TL-AMPDPCKSV---
F------ARFMSRFDP----------P--------KPSDNANVSYVVYK--------GD-----------------
-----YYVTG-ENN--CMYKV----DPETLEMK--EK-------V----------D-------------------
WT--KFVAV--NGA----------TAHPHYAP-------DGTAYN------------M--GNSYGKF---------
---GTTYNIIEV-------------------------------------PPQ---------KS--
NCNETLEGAKVLCSIAPTDNM-KPS-------YYHSFG-MSENYIIFIEQPI--KLNLLRI----ITS-KFR-
GKP-ISEGI-------------------------NWEPQYNTR-FHVVDKR-------TG-------KVL-
PGQWY--------------T-----K--PFVTFHQINAF----E--DRG---CVVLDLCCQD--DGKT--------
---------LAV--YKLQNM----------------RKSGADLDQIFGSVART----------------------
FPRRFVLPL------------------------KVNSDTPVGKNLN--------------------
PLSYTSAKAVKDS---------------------------DGKVWCTH----------------------------
----

ENLHPDGF------ENFGGL----EFPQINYSQYSGRKYRYFYGCG---FRHFIG---------------
DSLMKVDVETK--------N--------------------FK--------IWQEDGSY--PSEPVFVPV---P-
NATA---EDSG--VILSVVISP-------DE-------NRSAFL---LVLDAETFRELG--RAEV--P--VQM-
PYGFHGIF--SSR---------------------

-
>Zebrafish_BCO2a

MSSMNRLNSSKSGWKKTRPKNKDQYFT--
DVHGLPSI------EKLICSA--DET------P-E---PI-T--TTI------------------TGN--VPSWI-
KGNFLRNGPG-----KF------EI-----GR---SSFNHWFDGMALLHQFHI---E-----DGK------V----
--TYMSRFLNS------DSYKENLEHNRI--IVSE-FG----TV-AMPDPCKNF---F------QRFLSRFEL---
-------P--------KLSDNANVSFVQYK--------GD----------------------YYVST-ETN--
FMHKI----DPETLETK--EK-------V----------D-------------------WS--KFIAV--NGA---
-------TAHPHVDS-------DGTTYN------------M--GNSYTVK------------GAFYNIIMV-----
--------------------------------PPN---------KE--NPNDTLEGASVVCSIPSEDKS-KPS---
----YYHSFA-MSENYVVFIEQPI--KMDLLKI----VTG-KLR-GKG-INEGV----------------------
---YWDPKRNTV-FHVINKR-------TG-------KLS-LVKYY--------------T-----K--
PLSTFHQINCY----E--DNG---FLIMDMCSSD--DGQA-----------------INN--YVIQNL--------
--------KKSGSALDEVYNTLCRV----------------------FPRRFVLPL--------------------
----NVDSDTPHGENLI-----------------TCFNSTATAIKTDNNK--------------------------

```
---V-FCTY-------------------------------------------------------------------
----------------------------------------------------------------------------
----------------------------------------------------------------------------
----------------------------------------------------------------------------
----------------------------------------------------------------------------
----------------------------------------------------------------------------
-----------------------------EDLHGEDL------HEYGGL----EFPHINYSMCNSHPYRYFYGCG-
--FRHLVG---------------DSLIKMDLQGK--------Q--------------------IK--------
VWKHAGMF--PSEPVFVSS---P-GAVE---EDDG--VILSVVITP------------NQ--DKSTFL---
LVLDAKTFEELG--RAEV--P--KNI-PYGFHGTF-----------------------------------------
----QS----------------------------------------------------------------------
----------------------------------------------------------------------------
----------------------------------------------------------------------------
----------------------------------------------------------------------------
----------------------------------------------------------------------------
----------------------------------------------------------------------------
----------------------------------------------------------------------------
----------------------------------------------------------------------------
----------------------------
>Zebrafish_BCO2b
----------------------------------------------------------------------------
--------------------------------------------------------------------
MSCIKSQTIAAAQKTYFT----------------------------------------------KVQGLPNI----
--TPLVSSV--KET------P-E---PI-P--TTV------------------TGT--IPTWI-HGSLLRNGPG--
---KF------EF-----GN---QSYNHWFDGMALMHRFQI---E-----DGQ------V------TYRSRFLQS-
-----DSYKKCSERNRI--MVSE-FG----TV-ALPDPCKNI---F------QRFLSRFEV----------L----
----KPTDNASVNFVKYK--------GD----------------------YYVST-ETN--YMHRV----
DPETLESK--QK-------V----------D-------------------WS--NFVAV--NGA----------
TAHPHFDP-------DGTAYN------------M--GNSYRNK------------GAMYNIIRV------------
-------------------------SPE---------RD--GPKDTLEGAKILCSIAPRDKS-KPS-------
YYHSFG-MSENYVVFIEQPI--IMDLFKI----ITG-RLW-GNS-INRGI-------------------------
YWDPNQETI-FHLIDKQ-------TG-------KEM-PVKYY--------------T-----K--
ALSTFHQINAF----E--QDG---FLMLDMCCSD--DGEA-----------------INN--FLIQNM--------
--------RQSGEALDEMYNTMSRP----------------------LPRRFVLPL--------------------
----HISSETPLEQNLN-----------------TRLDSTATAVCRTKTQ--------------------------
---V-FCTF-------------------------------------------------------------------
----------------------------------------------------------------------------
----------------------------------------------------------------------------
----------------------------------------------------------------------------
----------------------------------------------------------------------------
----------------------------------------------------------------------------
-----------------------------EDLHGEDL------KDYGGL----EFPHINYAKYNTKPYRYYYGCG-
--FRHIVG---------------DSLIKMDLETK--------K--------------------LK--------
IWRQPDLY--PSEPVFIPS---P-NAED---EDDG--VILSVIITP------VK--------DKSTFL---
LVLDAKTFEELG--RAEV--P--VNI-PYGFHGVF-----------------------------------------
---NSSV---------------------------------------------------------------------
----------------------------------------------------------------------------
----------------------------------------------------------------------------
----------------------------------------------------------------------------
----------------------------------------------------------------------------
----------------------------------------------------------------------------
----------------------------------------------------------------------------
----------------------------------------------------------------------------
----------------------------
>Human_RPE65
----------------------------------------------------------------------------
----------------------------------------------------------------------------
```

---MSIQ---VEHPAGGY------
KKLFETV--EEL------S-S---PL-T--AHV------------------TGR--IPLWL-TGSLLRCGPG----
-LF------EV-----GS---EPFYHLFDGQALLHKFDF---K-----EGH------V------TYHRRFIRT---
---DAYVRAMTEKRI--VITE-FG----TC-AFPDPCKNI---F------SRFFSYFRG----------V------
--EVTDNALVNVYPVG--------ED----------------------YYACT-ETN--FITKI----
NPETLETI--KQ-------V----------D-------------------LC--NYVSV--NGA----------
TAHPHIEN-------DGTVYN------------I--GNCFGKN--------FSI---AYNIVKI------------
-------------------------PPL---------QA--DKEDPISKSEIVVQFPCSDRF-KPS-------
YVHSFG-LTPNYIVFVETPV--KINLFKF----LSSWSLW-GAN-YMDCF-------------------------
ESNETMGVW-LHIADKK-------RK-------KYL-NNKYR--------------T-----S--
PFNLFHHINTY----E--DNG---FLIVDLCCWK-GFEFV-----------------YNY--LYLANL--------
--------RENWEEVKKNARKAPQP----------------------EVRRYVLPL--------------------
-----NIDKADTGKNLV--------------------TLPNTTATAILCS--------------------------
-DETIWLEP---

-----------------------------EVLFSGPRQAF-------------EFPQINYQKYCGKPYTYAYGLG-
--LNHFVP---------------DRLCKLNVKTK--------E--------------------TW--------
VWQEPDSY--PSEPIFVSH---P-DALE---EDDG--VVLSVVVSP-------GAG------QKPAYL---
LILNAKDLSEVA--RAEVE----INI-PVTFHGLF---
---KKS--

>Dog_RPE65

---MSIQ---
--VEHPAGGY------
KKLFETV-EELS------------SPLT--AHV------------------TGR--IPLWL-TGSLLRCGPG----
-LF------EV-----GS---EPFYHLFDGQALLHKFDF---K-----EGH------V------TYHRRFIRT---
---DAYVRAMTEKRI--VITE-FG----TC-AFPDPCKNI---F------SRFFSYFRG-----------------
-VEVTDNALVNVYPVG--------ED----------------------YYACT-ETN--FITKI----
NPETLETI--KQ-------V----------D-------------------LC--NYVSV--NGA----------
TAHPHIEN-------DGTVYN------------I--GNCFGKN--------FSI---AYNIVKI------------
-------------------------PPL---------QA--DKEDPISKSEVVVQFPCSDRF-KPS-------
YVHSFG-LTPNYIVFVETPV--KINLLKF----LSSWSLW-GAN-YMDCF-------------------------
ESNETMGVW-LHIADKK-------RK-------KYL-NNKYR--------------T-----S--
SFNLFHHINTY----E--DNE---FLIVDLCCWK-GFEFV-----------------YNY--LYLANL--------
--------RENWEEVKKNARKAPQP----------------------EVRRYVLPL--------------------
-----NIDKADTGKNLV--------------------TLPNTTATATLRS--------------------------
-DETIWLEP---

-----------------------------EVLFSGPRQAF-------------EFPQINYQKSGGKPYTYAYGLG-
--LNHFVP---------------DRLCKLNVKTK--------E--------------------TW--------
VWQEPDSY--PSEPIFVSH---P-DALE---EDDG--VVLSVVVSP-------GAG------QKPAYL---

LILNAKDLSEVA--RAEVE----INI-PVTFHGLF---
---KKS--

>Cow_RPE65

--MSSQVEHP------
AGGYKKL-FETV--------EELSSPLT--AHV------------------TGR--IPLWL-TGSLLRCGPG----
-LF------EV-----GS---EPFYHLFDGQALLHKFDF---K-----EGH------V------TYHRRFIRT---
---DAYVRAMTEKRI--VITE-FG----TC-AFPDPCKNI---F------SRFFSYFRG-----------------
-VEVTDNALVNIYPVG--------ED----------------------YYACT-ETN--FITKV----
NPETLETI--KQ--------------------------------------VDLCNYVSV--NGA----------
TAHPHIEN-------DGTVYN------------I--GNCFGKN--------FSI---AYNIVKI------------
-------------------------PPL---------QA--DKEDPISKSEIVVQFPCSDRF-KPS-------
YVHSFG-LTPNYIVFVETPV--KINLFKF----LSSWSLW-GAN-YMDCF-------------------------
ESNETMGVW-LHIADKK-------RK-------KYI-NNKYR--------------T-----S--
PFNLFHHINTY----E--DHE---FLIVDLCCWK-GFEFV-----------------YNY--SYLANL--------
--------RENWEEVKKNARKAPQP----------------------EVRRYVLPL--------------------
-----NIDKADTGKNLV--------------------TLPNTTATAILCS--------------------------
-DETIWLEP---

-----------------------------EVLFSGPRQAF-------------EFPQINYQKYGGKPYTYAYGLG-
--LNHFVP---------------DRLCKLNVKTK--------E--------------------TW--------
VWQEPDSY--PSEPIFVSH---P-DALE---EDDG--VVLSVVVSP-------GAG------QKPAYL---
LILNAKDLSEVA--RAEVE----INI-PVTFHGLF---
---KKS--

>Mouse_RPE65

---MSIQ---IEHPAGGY------
KKLFETV--EEL------S-S---PL-T--AHV------------------TGR--IPLWL-TGSLLRCGPG----
-LF------EV-----GS---EPFYHLFDGQALLHKFDF---K-----EGH------V------TYHRRFIRT---
---DAYVRAMTEKRI--VITE-FG----TC-AFPDPCKNI---F------SRFFSYFKG----------V------
--EVTDNALVNIYPVG--------ED----------------------YYACT-ETN--FITKI----
NPETLETI--KQ-------V----------D-------------------LC--NYISV--NGA----------
TAHPHIES-------DGTVYN------------I--GNCFGKN--------FTV---AYNIIKI------------
-------------------------PPL---------KA--DKEDPINKSEVVVQFPCSDRF-KPS-------
YVHSFG-LTPNYIVFVETPV--KINLFKF----LSSWSLW-GAN-YMDCF-------------------------

ESNESMGVW-LHVADKK-------RR-------KYF-NNKYR--------------T-----S--
PFNLFHHINTY----E--DNG---FLIVDLCCWK-GFEFV-----------------YNY--LYLANL--------
--------RENWEEVKRNAMKAPQP----------------------EVRRYVLPL--------------------
-----TIDKVDTGRNLV--------------------TLPHTTATATLRS--------------------------
-DETIWLEP---

-----------------------------EVLFSGPRQAF-------------EFPQINYQKFGGKPYTYAYGLG-
--LNHFVP---------------DKLCKLNVKTK--------E--------------------IW--------
MWQEPDSY--PSEPIFVSQ---P-DALE---EDDG--VVLSVVVSP-------GAG------QKPAYL---
LVLNAKDLSEIA--RAEVE----TNI-PVTFHGLF---
---KRS--

>Rat_RPE65

---MSIQ---
--IEHPAGGY------
KKLFETV-EELS------------TPLT--AHV------------------TGR--IPLWL-TGSLLRCGPG----
-LF------EV-----GS---EPFYHLFDGQALLHKFDF---K-----EGH------V------TYYRRFIRT---
---DAYVRAMTEKRI--VITE-FG----TC-AFPDPCKNI---F------SRFFSYFRG-----------------
-VEVTDNALVNIYPVG--------ED----------------------YYACT-ETN--FITKI----
NPETLETI--KQ-------V----------D-------------------LC--NYVSV--NGA----------
TAHPHIES-------DGTVYN------------I--GNCFGKN--------FTV---AYNIIKI------------
-------------------------PPL---------KA--DKEDPINKSEVVVQFPCSDRF-KPS-------
YVHSFG-LTPNYIVFVETPV--KINLFKF----LSSWSLW-GAN-YMDCF-------------------------
ESNESMGVW-LHVADKK-------RR-------KYF-NNKYR--------------T-----S--
PFNLFHHINTY----E--DNG---FLIVDLCCWK-GFEFV-----------------YNY--LYLANL--------
--------RENWEEVKRNAMKAPQP----------------------EVRRYVLPL--------------------
-----TIDKADTGRNLV--------------------TLPHTTATAILCS--------------------------
-DETIWLEP---

-----------------------------EVLFSGPRQAF-------------EFPQINYQKCGGKPYTYAYGLG-
--LNHFVP---------------DKLCKLNVKTK--------E--------------------IW--------
MWQEPDSY--PSEPIFVSQ---P-DALE---EDDG--VVLSVVVSP-------GAG------QKPAYL---
LVLNAKDLSEIA--RAEVE----TNI-PVTFHGLF---
---KKP--

>Chicken_RPE65

--MYSQVEHP------
AGGYKKL-FETV--------EELSSPVT--AHV------------------TGR--IPTWL-RGSLLRCGPG----
-LF------EV-----GA---EPFYHLFDGQALLHKFDF---K-----EGH------V------TYHRRFVRT---
---DAYVRAMTEKRI--VITE-FG----TY-AYPDPCKNI---F------SRFFSYFKG-----------------
-VEVTDNALVNVYPVG--------ED----------------------YYACT-ETN--FITKI----
NPDTLETI--KQ-------V----------D-------------------LC--KYVSV--NGA----------
TAHPHVEN-------DGTVYN------------I--GNCFGKN--------FSL---AYNIIRI------------
-------------------------PPL---------QA--DKEDPMNKSEVVVQFPCSDRF-KPS-------
YVHSFG-LTPNYIVFVETPV--KINLLKF----LSSWSLW-GAN-YMDCF-------------------------
ESNETMGVW-LHVAEKK-------KG-------RLL-NIKYR--------------T-----S--
AFNLFHHINTF----E--DNG---FLIVDLCTWK-GFEFV-----------------YNY--LYLANL--------
--------RANWDEVKKQAEKAPQP----------------------EARRYVLPL--------------------
-----RIDKADTGKNLV--------------------TLPYTTATATLRS--------------------------
-DETVWLEP---

-----------------------------EVIFSGPRHAF-------------EFPQINYKKYGGKPYTYTYGLG-
--LNHFVP---------------DRLCKLNVKTK--------E--------------------TW--------
VWQEPDSY--PSEPIFVSH---P-DALE---EDDG--VVLSIVISP-------GSG------PKPAYL---
LILNAKDMSEVA--RAEVE----VNI-PVTFHGLF---
---KRA--

>Zebrafish_RPE65a

---MVSR---LEHPAGGY------
KKVFESC--EEL------A-E---PI-P--AHV------------------SGK--IPAWL-SGSLLRMGPG----
-LF------EI-----GD---EPFNHLFDGQALIHKFDL---K-----DGR------V------TYHRKFIRT---
---DAYVRAMTEKRV--VITE-LG----TA-AYPDPCKNI---F------SRFFTYFQG----------T------
--EVTDNCSVNIYPIG--------ED----------------------FYACT-ETN--FITKV----
NPDTLETI--KK-------V----------D-------------------LC--NYLSV--NGL----------
TAHPHIEA-------DGTVYN------------I--GNCFGKN--------MSL---AYNIVKI------------
-------------------------PPL---------QE--EKSDPLAMSKVLVQFPSSERF-KPS-------
YVHSFG-MTENHFVFVETPV--KINLLKF----LTSWSIR-GSN-YMDCF-------------------------
ESNDRMGTW-FHLAAKN-------PG-------KYI-DHKFR--------------T-----S--
AFNIFHHINCF----E--DQG---FIVVDLCTWK-GHEFV-----------------YNY--LYLANL--------
--------RQNWEEVKKAALRAPQP----------------------EVRRYVLPL--------------------
-----DIHREEQGKNLV--------------------SLPYTTATAVMCS--------------------------
-DGTVWLEP---

-----------------------------EVLFSGPRQAF-------------EFPQINYSKFNGKDYTFAYGLG-
--LNHFVP---------------DRICKLNVKSK--------E--------------------TW--------
IWQEPDAY--PSEPLFVQS---P-DAED---EDDG--VLLSIVVKP-------GVS------QRPAFL---
LILKATDLTEIA--RAEVD----VLI-PLTLHGIY---
---KP---

>Zebrafish_RPE65b

--MVSRLEHP------
AGGYKKV-FESC--------EELAEPIP--AHV------------------SGE--IPAWL-SGSLLRMGPG----
-LF------EV-----GD---EPFYHLFDGQALLHKFDL---K-----DGR------V------TYHRRFIRT---
---DAYVRAMTEKRV--VITE-FG----TT-AYPDPCKNI---F------SRFFTYFQG-----------------
-IEVTDNCLVNIYPIG--------ED----------------------FYACT-ETN--FITKV----
DPDTLETV--KK-------V----------D-------------------LC--NYLSV--NGL----------
TAHPHIEA-------DGTVYN------------I--GNCFGKN--------MSL---AYNIVKI------------
-------------------------PPL---------QE--DKSDQFEKSKILVQFPSSERF-KPS-------
YVHSFG-ITENHFVFVETPV--KINLLKF----LTSWSIR-GSN-YMDCF-------------------------
ESNDKMGTW-FHLAAKN-------PG-------KYI-DHKFR--------------T-----S--
AFNIFHHINCF----E--DQG---FIVVDLCTWK-GHEFV-----------------YNY--LYLANL-------
RQ--------NWEE-VKKAALRAPQP----------------------EVRRYVLPL-------------------
DIHR---EE---QGKNLV--------------------SLPYTT-ATAVMR--------------------S--
DGT---V-WLEP--

--------------------------------EV----LF---S--GPRQAF----
EFPQINYSKFNGKDYTFAYGLG-L--NHFVP------------DR---ICKLNVKSK--------E----------
----------TW--------IWQEPDAY--PSEPLFVQS---P-DAED---EDDG--VLLSIVVKP-------
GVS--Q---R-PAFL---LILKATDLTEIA--RAEV--D--VLI-PVTLHGIY-----------------------
---------------------KP---

>Zebrafish_RPE65c

---MVSR---FEHPAGGY------
KKIFETA--EEL------N-E---PL-P--ATV------------------TGR--IPSFI-KGSLLRLGPG----
-LF------EA-----GA---EPFYHLFDGQALMHKFDF---S-----NGQ------V------TYFRKFVKT---
---DAYVRAITEKRV--VITE-FG----TC-AYPDPCKNI---F------SRFFSYFKG----------V------

--EVTDNCLVNVYPIG--------ED----------------------FYAVT-ETN--YITKV----
NVDTLETL--KK-------V----------D-------------------MC--NYVNI--NGV----------
TAHPHIER-------DGTVYN------------I--GNCMGKG--------ASL---AYNIVRI------------
-------------------------PPT---------QK--DKSDPIEKSKVVVQFPSAERF-KPS-------
YVHSFG-MTENYFVFVETPV--KINLLKF----LSAWSIR-GSN-YMDCF-------------------------
ESDEEKGTW-IHIARKH-------PG-------EYI-DYKFR--------------T-----S--
AMGLFHHINCY----E--DSG---FIVFDLCAWK-GFEFV-----------------YNY--LWLANL--------
--------RANWEEVKRNAMIAPQP----------------------EVRRYVIPL--------------------
-----DPFREEQGKNLI--------------------SLPYTTATATMRA--------------------------
-DGTIWLEP---

-----------------------------EVLFSGPRQAF-------------EFPQINYRMVNGKNYTYAYGLG-
--LNHFVP---------------DRICKLNVRTK--------E--------------------TW--------
VWQEPDSY--PSEPLFVQT---P-DGVD---EDDG--ILMTIVVSP-------GA-------QRPTYC---
LILNAKDLSEIA--RAEV-----EILTPVTFHGMY---
---KP---

>Pufferfish_BCMO1a

---M------
TTNFMVN-AVES------P-E---EQ-R--AEV------------------KGK--IPGWL-QGTLLRNGPG----
-IF------SV-----GD---TSYQHWFDGMAIMHSFTF---R-----DGE------V------FHKSRFLKS---
---DTYKANMAANRI--VVSE-MG----TM-AYPDPSKNF---I------FKAITFLNH----------T------
MPDFTDNGASNFIKYG--------ND----------------------YYATS-ETN--YIRMV----
DPETLETK--DK-------V----------D-------------------YM--KYLPV--NLV----------
SSHPHYD-R------EGNAYN------------I--GTSIAEK--------GKT---KYVLFKV------------
-------PAAATKDQ--GKK----VPAL---------KN----------VEVIASLPCRSML-SPS-------
YYHSFG-MTDDYFIFLEQPF--KLDILKM----ATA-YMR-GVN-WASCL-------------------------
KFSPEENTL-IHVIDRK-------TG-------KEV-ELKYY--------------T-----G--
SMVVYHHVNAY----E--EDG---HLVFDVIAYK--DNSL-----------------YDM--FYLSKL--------
-----------KENTGNPDENYSKP----------------------HYKRFVLPL--------------------
----TADKDTAVGENTV--------------------KLRNTRATAVKEK--------------------------
-EGKLLCQP---

-----------------------------EV----LCEGF-------------ELPRMN-
YDANGKRHRFVYGNCVEHSVVSKQ---------------MKIAKFNTETK--------E-----------------
---MV--------YWSEKNCW--PSEPVFVSR---P-HGES---EDDG--VALSSVINS------------NP--
DQLCYL---LILDGRTFKEVG--RAYVG----VKL-QKDMHGYF--------------------------------
------------IPR---

>Pufferfish_BCMO1b

---M------
ESVFGAN-GSET------P-E---PV-Q--AEV------------------RGS--IPSWL-QGTLLRNGPG----
-LF------SV-----GN---SEYNHWFDGLSLIHSFTF---K-----HGE------V------TYRSKFLRS---
---ETYKKNCKSNRI--VVSE-FG----TM-AYPDPCKNI---F------SRAFTHLCN----------A------
IPDFTDNNLINIIRYG--------QD----------------------YYAAS-EIN--YINQI----
DPVTLETV--GR-------V----------N-------------------YR--NHIAL--NLA----------
TAHPHYD-D------QGNTYN------------M--GTALMGL--------GPP---KYVIFKT------------
-------PAEAS-DQ--ERR----KPAL---------RK----------VQQVCSIPFRSTL-FPS-------
YFHSFG-MSENYIVFVEQPF--KLDMVKL----ATA-YFR-GVT-WGSCL-------------------------
KFEKDDATF-FHVINRT-------TG-------KAV-SSRFY--------------G-----D--
ALVTFHHINTY----E--DGG---HLVCDLITYR--DSSL-----------------YDL--FYIRNI--------
--------RQDTSTFVQSNHSFSPP----------------------VCKRFVLPL--------------------
----EANKDSPRGSNLV--------------------TLTDTTARAEMQE-----------------------
DGS---I-YCQP--

--------------------------------DT----LF---------EGL----ELPGIN-
YKFNSRKYRFFYGSRVEVSPHPYK-----------------LAKIDVVTR--------E-----------------
---HI--------EWKQESCF--PSEPVFVAS---P-GAVE---EDDG--VILSSVVSS-------DP-------
NVSSFM---LVLDAKTFTEVG--RASI--P--ASV-HLDLHGLF--------------------------------
------------IPASV---

>Stickleback_BCMO1a

--V---LQKKDQSF------
GAYLFLN-GFET------P-E---PV-K--AQV------------------NGS--IPPWL-QGTLLRNGPG----
-LF------SV-----GD---SEYNHWFDGMSLIHSFTF---S-----QGQ------V------TYRSKFLKS---
---ETYKRNIKANRI--VVSE-FG----TM-IYPDPCKNI---F------SRAFTHLSN----------I------
IPDFTDNNLINIVRYG--------QD----------------------YYASS-EVN--YMNQI----
DPDTLETI--GR-------I----------N-------------------YR--NHIAL--NLA----------
TAHPHYD-N------EGNTYN------------M--GTAIMGM--------GLP---KYVIFKV------------
-------PADAS-DN--KNK----KPAL---------RK----------VQQVCSIPFRSTL-FPS-------
YYHSFG-MTENYVVFVEQPF--KLDIVKL----ATA-YFR-GVN-WGSCL-------------------------
KFDKDDITL-FHVVDKK-------TG-------KAV-STRFH--------------G-----D--
PLVVFHHINAY----E--ADG---HVVLDLIAYR--DSNL-----------------YDM--FYLRNM--------
--------RQESEALVESNKDCSPP----------------------VCQRFVLPL--------------------
----HTSKDAPGGSELV--------------------TLTDTTARAVKHE-----------------------

```
DGS---V-YCRP----------------------------------------------------------------
----------------------------------------------------------------------------
----------------------------------------------------------------------------
----------------------------------------------------------------------------
----------------------------------------------------------------------------
----------------------------------------------------------------------------
--------------------------------ET----LF---------EGL----ELPAIN-
YGFNGKKYRYFYGSRMEWSPHPNK-----------------IAKFDIVTR--------E-----------------
---HV--------EWRQENCF--PSEPVFVAS---P-GAVE---EDDG--VILSSVISP-------DP-------
NISPFM---LVLDARSLEEVA--RASI--P--APV-HMDLHGHF--------------------------------
------------IAAPV-----------------------------------------------------------
----------------------------------------------------------------------------
----------------------------------------------------------------------------
----------------------------------------------------------------------------
----------------------------------------------------------------------------
----------------------------------------------------------------------------
----------------------------------------------------------------------------
----------------------------------------------------------------------------
-------------------------------------
>Stickleback_BCMO1b
----------------------------------------------------------------------------
----------------------------------------------------------------------------
---------------------------------------------------------------M------
TTNFSQN-AEES------P-E---PR-K--AEV------------------KGN--IPSWL-QGTLLRNGPG----
-IF------SV-----GV---TSYDHWFDGMAIMKSFAI---K-----DGE------V------TYRSRFLES---
---DTYKANMAANRI--VVSE-MG----TM-AYPDPSKNF---I------VKAITFLNH----------T------
VPDFTDNGASNFIKYG--------KD----------------------YYATS-ETN--YIRKI----
DPVTLETQ--DK-------V----------D-------------------YM--KYLPV--NLA----------
SSHPHYD-K------EGNAYN------------F--GTSIAEK--------AKT---KYILFKV------------
-------PAVS--ET--DKD----SPAL---------KK----------VEILCTVPCRSLL-TPS-------
YYHSFG-LTENYFIFIEQPL--KLDILKM----ATA-YMR-GVN-WASCL-------------------------
KFCPEESTL-IHLIDRK-------TG-------KVV-DTKYY--------------T-----G--
AMVVYHHVNAF----E--DDG---HVIFDVIAYK--DNSL-----------------YDA--FYLNRM--------
----------KENPGSEDDDGYSKP----------------------SYKRFVLPI--------------------
----RSDKGVAVGEDLV--------------------KLKYTTASAVKEK--------------------------
-GGKLLCQA-------------------------------------------------------------------
----------------------------------------------------------------------------
----------------------------------------------------------------------------
----------------------------------------------------------------------------
----------------------------------------------------------------------------
----------------------------------------------------------------------------
-----------------------------EV----VCEGF-------------ELPRLN-
YDFNGKKHRFVYGCSVEKCAVAKG-----------------IAKLDTETK--------E-----------------
---RI--------YWSEDHCS--PSEPIFIPR---P-NGES---EDDG--VVLATVINY------------NP--
GQSSFI---LILDGRTFEEVA--RAYVN----TTL-NRDMHGFF--------------------------------
------------IPLQN-----------------------------------------------------------
----------------------------------------------------------------------------
----------------------------------------------------------------------------
----------------------------------------------------------------------------
----------------------------------------------------------------------------
----------------------------------------------------------------------------
----------------------------------------------------------------------------
----------------------------------------------------------------------------
-------------------------------------
>Zebrafish_BCMO1a
----------------------------------------------------------------------------
----------------------------------------------------------------------------
```

---M------
QYDYGKN-KEEH------P-E---PI-K--TEV------------------KGS--IPEWV-QGTLIRNGPG----
-MF------SV-----GE---TTYNHWFDGMALLHSFAI---N-----KGE------V------TYRSRYLRG---
---DTYNSNMQANRI--VVSE-MG----TM-AYPDPCKNI---F------SKVITFLSH----------T------
IPDFTDNCGNNIIKYG--------ND----------------------FHATS-ETN--YIRKI----
DPVTLETQ--EK-------I----------D-------------------YL--KYLPV--SIV----------
ASHTHYD-K------EGNSYS------------M--GTCIAEK--------GKT---KYMLFKV------------
-------PGE---SR--PDG----SPPL---------KS----------AEAVCTLPCRSLL-TPS-------
YYHSFG-MTDNYFIFIEQPL--KLDILKM----ATA-YLR-RVS-WASCM-------------------------
KFHPEDSTL-IHLIDRN-------TK-------KEV-ATKFY--------------T-----D--
AMTVYHQVNAF----E--DDG---HVVFDVIAYD--DNNL-----------------YEF--FYLNKL-------
KE-------TMGAT------NLYCKP----------------------KFTRFVFPL-------------------
-------SDQGETGENLV--------------------KLKYTTASAVKEK-----------------------
DGK---I-MCQG--

--------------------------------EV----LC---------EGV----ELPRIN-
YNFNGKKYRYSYMCC-VDESPVAT----------------RIVKFDADTK--------Q-----------------
---QI--------EWKGDDGF--ASEPVFIPR---P-GAVD---EDDG--VVLTVIINN-------K----P---
LQGGFL---LVLDAKSFKEIA--RACL--D--VEI-HMDMHGYF--------------------------------
------------IPGSS---

>Medaka_BCMO1a

SLVIEQNQFVRIVCPVLLLLFCLLSVVF--
SRRLVIPA------LADFSKN-ATER------P-E---PC-N--AEV----------------VGTGS--IPDWL-
QGTLVRNGPG-----LF------SV-----GE---TSYDHWCDGMSFLNSFSF---K-----NGK------V----
--THRSRFLRS------DTYNSNMAANRI--VVSE-MG----TM-AYPDPSKNF---I------FKAITFLNH---
-------T------VPDFTDNAASNIIKYG--------ND----------------------YYATS-ETN--
YIRKI----DPFTLETQ--DK-------V----------D-------------------YM--KFLPV--NLV---
-------SSHPHYD-R------EGNVYN------------V--GTSIAEK--------GKT---KYIFFQI-----
--------------PAVTEKEK--DKN----TPAL---------KK----------VKVICTVPCHSLL-SPS---
----YYHSFG-MTENYIIFIEQPF--KLDILKM----ATA-YMR-GVN-WASCM----------------------
---KFFPEENTL-IHLIEKK-------TG-------KKV-EMKYH--------------T-----G--
SLVVYHHVNAF----E--DDG---HVVLDVIAYN--DTSL-----------------YEM--FYMSRL--------
-----------KKDISFQSNGYTKP----------------------NYRRFVLPI--------------------
----RSDKGIAVGENMV--------------------KLRYTTATAVKQK--------------------------
-EGKLTCQA---

-----------------------------EVLCEVYLSYSF------------ELPRIN-
YDFNGKKHQFVYGNIVEESVLSTQ-----------------VAKFDTETK--------K-----------------
---MV--------HWSEENCW--PSEPVFVPR---P-NGES---EDDG--VVLVSVINC--------------

IPGQSSFI---LILDGRNFKEVA--RANVN----TEL-HRDMHGFF------------------------------
--------------IPHTN---

>Medaka_BCMO1b

--LVH---CGSEDANM------
QSFIAKN-GNET------P-E---PV-K--AQV-----------------KAGS--LPSWL-QGTLLRNGPG----
-LF------SV-----GD---SEYNHWFDGSSLIHSFTF---S-----NGE------V------SYRSKFLKS---
---DTYKRNIKANRI--VVSE-FG----TM-VYPDPCKNI---F------ARTATHLSN----------I------
VPNFTDNNLINIIRYG--------ND----------------------YYASS-EVN--YINQI----
DPDTLETV--GR-------V---------N-------------------YR--NHIAL--NLA----------
TAHPHYD-D------EGNTYN------------M--GTAIIGL--------GRP---KYIIFKV------------
-------PAKAT-GK--DQK----KPAL---------RQ----------VQQICSIPFRSAL-FPG-------
YFHSFG-MTENYIVFVEQPF--KLDIVKL----ATA-YFR-GVN-WGSCL-------------------------
KFDEKDTTL-FHIIDRK-------TG-------KKI-SAHYY--------------A-----D--
ALVVFHHINAY----E--ENG---HVVFDMITYK--DSNL-----------------YNM--FYLHNM--------
--------KKDTNAFIQTNQDFSPP----------------------VGQRFVLPL--------------------
-DVHKVEQESPKGTNLV--------------------TLTDTSAEAVMQE-----------------------
DGS---V-YCKP--

--------------------------------DN----IF---------VGLASSMELPGIN-
YKFNARKYRYFYGSRLEWSPHPNK-----------------IAKVDIATR--------T-----------------
---HL--------EWYQENCY--PSEPVFVAS---P-GAVE---EDDG--VILSSVVSS-------DP-------
DITPFM---LVLNAKTFEEIA--RASI--P--TTV-HMDLHGLF--------------------------------
------------IP--

>Fugu_BCMO1a

---M------
EAIFAKN-GSET------P-E---PV-E--AEV------------------RGS--IPSWL-QGTLLRNGPG----
-LF------SV-----GN---SQYNHWFDGLSLIHSFTF---R-----NGE------V------TYRSKFLRS---
---ETYKKNCTSNRI--VVSE-FG----TM-VYPDPCKNI---F------SRAFTHLCN----------A------
IPDFTDNNLINIIRYG--------KD----------------------YYASS-EIN--YINQI----
DPVTLETV--GR-------V---------N-------------------YR--NHIAL--NLA----------
TAHPHYD-E------EGNTYN------------M--GTALIGL--------GLP---KYVIFKI------------
-------PAETS-DK--GHK----KPAL---------RK----------VQQICSIPFRSTM-FPS-------
YFHSFG-MTENYIVFVEQPF--KLDIIKL----ATA-YFR-GVT-WGSCL-------------------------

```
KFDKDDATF-FHIINRT-------TG-------KAV-STRFY--------------G-----D--
ALVVFHHINAY----E--DDG---HLVCDLITYS--DSSL-----------------YDM--FYIRNV--------
--------KQDTSNFVQSNQSFCPP----------------------VCKRFVFPL--------------------
----NVNKDSPRGSNLV--------------------TLTDTTAQAAMQE-----------------------
DGS---I-YCQP----------------------------------------------------------------
----------------------------------------------------------------------------
----------------------------------------------------------------------------
----------------------------------------------------------------------------
----------------------------------------------------------------------------
----------------------------------------------------------------------------
--------------------------------DT----LF---------EGL----ELPGIN-
YKFNSKKYRFFYGSRVEVSPHPYK-----------------LAKIDVVTR--------K-----------------
---HL--------EWKQELCF--PSEPVFVAS---P-GADE---EDDG--VILSSVISS-------DP-------
KVSPFM---LILDAKTFTEIA--RASI--P--TSV-HLDLHGLF--------------------------------
------------IPDHWLDAETE-----------------------------------------------------
----------------------------------------------------------------------------
----------------------------------------------------------------------------
----------------------------------------------------------------------------
----------------------------------------------------------------------------
----------------------------------------------------------------------------
----------------------------------------------------------------------------
----------------------------------------------------------------------------
-------------------------------------
>Fugu_BCMO1b
----------------------------------------------------------------------------
----------------------------------------------------------------------------
----------------------------------------------------------------------------
----------------------------------------------------------------------------
------------------------MAIMHSFTF---K-----DGE------V------SHRSKFLRS------
DTYNANMAANRI--VVSE-MG----TM-AYPDPSKNF---I----VKLRAITFLNH----------T------
MPDFTDNGASNFIKYG--------ND----------------------IYATS-ETN--YIRMI----
DPVTLETK--DK-------V----------D-------------------YM--KYLPV--NLA----------
SSHPHYD-K------EGNAYN------------M--GTSIAEK--------GFL---KKIMLVL------------
-------NPA---DQ--GKN----TPAL---------KN----------VEVIASVPCRSML-TPS-------
YYHSFA-MTDNYFIFLEQPF--KLDILKM----ATA-YMR-GVN-WASCL-------------------------
KFCPEESTL-IHLINRK-------TG-------KEV-ETKFH--------------T-----G--
SIIIYHHVNAY----E--EDD---HVVFDVIAYK--DNHL-----------------YDM--FYLSKL--------
-----------KENTGKPDENYSKP----------------------NYNRYVLPL--------------------
----ISDKGIAVGEDLV--------------------KLPNTRATAVKEK--------------------------
-EGKLLCQP-------------------------------------------------------------------
----------------------------------------------------------------------------
----------------------------------------------------------------------------
----------------------------------------------------------------------------
----------------------------------------------------------------------------
----------------------------------------------------------------------------
-----------------------------EVLCEAGF----------------ELPRIN-
YDINGKRHRFVYGNCVEHSVVSKQ-----------------IAKFDTESK--------E-----------------
---MV--------FWTEENCW--PSEPVFVPR---P-NGES---EDDG--VVLSSVINT------------NP--
GQSCYL---LVLDGKTFKEVG--RAYVG----AKL-QKDMHGYF--------------------------------
------------IPH-------------------------------------------------------------
----------------------------------------------------------------------------
----------------------------------------------------------------------------
----------------------------------------------------------------------------
----------------------------------------------------------------------------
----------------------------------------------------------------------------
----------------------------------------------------------------------------
```


>Pufferfish_BCO2a

EPDPISSHTAPGKSGKKVRRFT---
EARGLPCI------ERMVSSV--EET------P-E---AV-D--AHV------------------VGQ--IPEWV-
NGNFLRNGPG-----KF------EI-----GN---QRFNHWFDGMALLHQFKI---A-----SGQ------V----
--TYKSRFLAS------DSYQSNTENNRI--TVSE-FG----TV-TLPDPCRNF---F------QRFLSRFEL---
-------P--------KPTDNANVSVVTYK--------GN----------------------YYVST-ETK--
IIHKL----DPEKLETT--QK-------V----------D-------------------WS--KFIAV--NGA---
-------TAHPHTEP-------DGTTYN------------M--GNSYTSK------------GAYYNIIRV-----
--------------------------------PAA---------KK--REEETLEGATVLCTIPSSDKT-KPS---
----YYHSFA-MTENYVVFVEQPI--RMDLFKI----VTG-KLR-GRS-ISDGF----------------------
---LWNPKRNTV-FHLVDKR-------TG-------EVS-SVKYL--------------A-----K--
ALSTFHQINAY----E--DDG---FLVLDMCASD--DGLA-----------------IAN--YNLQNL--------
--------RKSGEALDEVYNTLCRV----------------------FPRRFVLPL--------------------
----HVDGATAYNQNLN-----------------ARHSSTATAVRTGRNK--------------------------
---V-CCTH---

-----------------------------EDLHGEDL------HRYGGL----EFPQIHYRRYNARPYRYFYACG-
--FRHLVG---------------DSLIKMDVGSK--------S--------------------MK--------
VWEQAGFY--PSEPVFVPA---P-NARA---EDDG--VVLSVVVTP-------D----K---ERSAFL---
LVLDARRFQELG--RAVV--P--ATI-PYGFHGTF---
----SAT---

>Pufferfish_BCO2b

---GLETI------
APLVRSV--EET------P-E---PI-S--TEV------------------QGT--IPSWI-HGNLLRNGPG----
-KF------EF-----GN---RHYNHWFDGMAMLHQFRI---S-----EGR------V------TYMSRFLHS---
---DVYKKNSEQDRI--VMSE-FG----TL-ALPDPCKNV---F------QRFLSRFEM----------I------
--EPTDNASVNFVKYK--------GD----------------------YYVST-ETN--FMHRV----
DPENLETL--EK-------V----------D-------------------WS--KFVAV--NGA----------
TAHPHYDP-------DGTSYN------------M--GNSYGRK------------GALYNIIRV------------
-------------------------PPE---------KS--EATETLHGAKVLCSIVPQHKS-RPS-------
YYHSFA-MSENYVVFIEQPI--KMDLLKI----VTC-RLR-GRA-LCEGI-------------------------
YWDGGQDTV-FHLVSKH-------TG-------EVS-TVKYH--------------T-----K--
AFATFHQINAF----E--EDG---FLMIDLCCSD--NGQA-----------------INN--YLIQNL--------
--------RKSGEALDEVYNSTGRA----------------------FPRRFVLPL--------------------
-------------------------------------HVTSQTAAGHNLN--------------------------
--TRETCVK---

-----------------------------TDKDTVFCQHEDLHGDDLLEFGGLEFPQINYGRCNTKPYRYFYGCG-
--FRHLVG---------------DSLLKMDLKDKTFKLQRPAG--------------------AL--------
RWT-------PVTLVFTPH------SRQ---RVVG--CLQKRLLTS-------T--------DDLCLFR--
QVLDGR-LPELG--RANV--P--VNM-PYGFHGIY---
---EA---

>Pufferfish_BCO2c

LVTSV--EET------P-D---AI-P--TAI------------------SGT--IPTWI-HGSFLRNGPG-----
KF------EF-----GE---DSYTHWFDGMALMHRFHI---Q-----EGN------V------TYSSRFLRS----
--DSYVSNSEKNRI--VVSE-FG----TM-AAPDPCKNI---F------ARFFSRFQS----------S-------
TEATDNANVNLXRI---------------------------------YYLFV------RCSPV----SNDTKTST-
-GS-------R----------P-------------------WN-WGTLVY--VNQ----Q-----GSLPAVHEF--
----YFNFFP------------L--GFFYNIV--------CVPPYDEQTVAKD-----------------------
-------------FPDL---------SG----------AKVICSIRASEPR-KPS-------YYHSFA-
MSENYIVFIEQPI--KMDLLKF----MLY-KIQ-GKS-FHRVM-------------------------
SWQPHYGTI-FHLVNRH-------TG-------EES-KVKYS--------------A-----A--
AMFTLHQINAF----E--ENG---FLVMDMCCGD--NGEI-----------------IGD--FTLENL-------
RR--------ESGEDVDKFYNSLCRN----------------------LPRRYVLPL-------------------
-----NVDGQTPSDQNLV--------------------TLHYHKATAQMIQ-------------------------
--PGEVYLTH--

------------------------------EELHDDEL------LLYGGL----
EFPQINYDRCNGRPYRYFYACG---FGHVFA---------------DSLLKMDVHTK--------K----------
----------MK--------VWRHPGLY--PSEPVFVAS---P-GAAE---EDDG--VVLSVIITP-------R--
--K---EKSTFL---LVLDAKTFTELG--RAEV--P--VNI-PYGTHGIF--------------------------
-------------------TNS--

>Fugu_BCO2a

RQRKSQNSSGKTTWCRNKGISHVPAHQQRHYT--
RLHGLETI------APLVRSV--KET------P-E---PI-P--TEV------------------EGT--IPSWI-
DGNLLRNGPG-----KF------EF-----GN---RHYNHWFDGMAMLHQFRI---R-----GGG------V----
--TYMSRFLRS------DVYKKNSEQDRI--VISE-FG----TL-ALPDPCKNF---F------QRFLSRFEM---

```
-------I--------QPTDNASVNFVKYK--------GD----------------------YYVST-ETN--
FMHRV----DPEKLESL--EK-------V----------D-------------------WS--KFIAV--NGA---
-------TAHPHYDP-------DGTSYN------------M--GNSYGHK------------GALYNIIRV-----
--------------------------------PPE---------KT--EATETLHGAKVLCSIVPKDKS-RPS---
----YYHSFA-MSENYVVFIEQPI--KMDLLKI----VTC-KLR-GKA-LCEGI----------------------
---YWDAGQDTV-FHLVHKH-------TG-------EVS-AVKYH--------------T-----K--
ACSTFHQINAF----E--EDG---FLMIDLCCSD--DGEA-----------------INN--YLIQNL--------
--------RKSGDALDEVYNSTGRA----------------------FPRRFVLPL--------------------
----HVTSEMATGQNLN-----------------TRASSQATCVKTGKDT--------------------------
---V-FCQH-------------------------------------------------------------------
----------------------------------------------------------------------------
----------------------------------------------------------------------------
----------------------------------------------------------------------------
----------------------------------------------------------------------------
----------------------------------------------------------------------------
-----------------------------EDLHGADL------CEFGGL----EFPQINYSRCNTKPYRYFYGCG-
--FRHLVG---------------DSLLKMDLKDK--------T--------------------FK--------
VWYQKGFY--PSEPVFVPA---P-DAED---EDDG--VILSVVLTP-------S----R---DKGTFL---
LVLDGKTFQELG--RANV--P--VNM-PYGFHGIY-----------------------------------------
----EA----------------------------------------------------------------------
----------------------------------------------------------------------------
----------------------------------------------------------------------------
----------------------------------------------------------------------------
----------------------------------------------------------------------------
----------------------------------------------------------------------------
----------------------------------------------------------------------------
----------------------------------------------------------------------------
----------------------------
>Fugu_BCO2b
----------------------------------------------------------------------------
---------------------------------------------------------
FSSIGHMQFHTNSAASALKKSKSPQKVQRFT--------------------------------------------
DVRGLPCI------ERMVSSV-EETP--------E---PI-C--TDI------------------VGQ--IPEWI-
DGNFLRNGPG-----KF------EI-----GN---QKFNHWFDGMALLHQFKI---A-----RGQ------V----
--TYKSRFLSS------DSYQANKENNRI--TVSE-FG----TI-TLPDPCKNI---F------QRFLSRFEL---
---------------PKPTDNANVSFVTYK--------GD----------------------YYVST-ETT--
IIHKL----DPEKLETT--QK-------V----------D-------------------WS--KFIAV--NGA---
-------TAHPHTEP-------DGTTYN------------M--GNSYTSK------------GAYYNIIRV-----
--------------------------------PPT---------KR--TEEETLEGTTVLCTIPSSDKT-KPS---
----YYHSFA-MTENYVVFVEQPI--KMDLFKI----VTG-KLR-RKS-ISDGF----------------------
---LWDPKRNTI-FHVIHKK-------TG-------EVS-SVKYL--------------A-----K--
PLSTFHQINAY----E--EDG---FLVLDMCASD--DGLA-----------------IAN--YNIQNL--------
--------RKSGEALDEVYNTLCRV----------------------FPRRFVLPL--------------------
----HVDQDTAYDQNLN-----------------ARHSSTATAIRIARNK--------------------------
---V-CCTH-------------------------------------------------------------------
----------------------------------------------------------------------------
----------------------------------------------------------------------------
----------------------------------------------------------------------------
----------------------------------------------------------------------------
----------------------------------------------------------------------------
-----------------------------EDLHGEDL------HHYGGL----EFPQINYAKYNTRPYRYFYGCG-
--FRHLVG---------------DSLIKMDVHSK--------S--------------------MK--------
VWEQPGFY--PSEPIFVPS---P-GAAE---EDDG--VILSVVITP-------N----K---DRSTFL---
LVLDARTFEELG--RARV--P--VNI-PYGFHGTF-----------------------------------------
----------------------------------------------------------------------------
----------------------------------------------------------------------------
----------------------------------------------------------------------------
```

```
----------------------------------------------------------------------------
----------------------------------------------------------------------------
----------------------------------------------------------------------------
----------------------------------------------------------------------------
----------------------------------------------------------------------------
----------------------------
>Fugu_BCO2c
----------------------------------------------------------------------------
--------------------------------------------------------------------
MSSEPQHIPSWQRCP-------------------------------------------------RGSGLESL----
--AALLSSV--EET------P-D---PI-P--TTI------------------KGT--IPTWI-KGSFLRNGPG--
---KF------EF-----GE---ESYTHWFDGMALMHRFHI---Q-----EGN------V------TYSSRFLRS-
-----DSYVSNSEKNRI--VVSE-FG----TL-AMPDPCKNI---F------ARFFSRFQV----------P----
------------NYWRQS--------QR----------------------------NSE-KTFLRF----
HFPALAFV----------------------P-------------------TE----------------------
GRCLVIV--------------------------R--IYRPRNP--------PGF---FYHILRV------------
-------PPRDEPADATAD-----SPDL---------SG----------ARIVCSIRAPESR-KPS-------
YYHSFA-MSENYVVFIEQPI--KMDLLKF----MLY-KIQ-GKS-FHRIM-------------------------
SWQPQYGTV-FHLVNRH-------TG-------EES-DVKYR--------------A-----A--
AMFTLHQINAF----E--ENG---FLVMDMCCGD--NGEV-----------------IGD--FTLENL--------
--------RRGSGDIDKFYNSLCRN----------------------LPRRYVLPL--------------------
----NVDRQAPLDENLV--------------------TLHHCKATAKMIK--------------------------
-PGEVYMTH-------------------------------------------------------------------
----------------------------------------------------------------------------
----------------------------------------------------------------------------
----------------------------------------------------------------------------
----------------------------------------------------------------------------
----------------------------------------------------------------------------
-----------------------------EELHDAEL------LQYGGL----EFPQINYEEYNGRPYRFFYSCG-
--FGHVFA---------------DSLLKMDVHTK--------E--------------------LK--------
VWRHPGLY--PSEPVFVAS---P-NAAE---EDDG--VVLSVIVTP-----------------RKASI---
TLPQINTLKKLE-LKKIVHVS--VSVCRRKARSSW-----------------------------------------
-FWMPRPSLNWAERRSLSISPVGRMASLATRAKR------------------------------------------
----------------------------------------------------------------------------
----------------------------------------------------------------------------
----------------------------------------------------------------------------
----------------------------------------------------------------------------
----------------------------------------------------------------------------
----------------------------------------------------------------------------
----------------------------------------------------------------------------
----------------------------
>Medaka_BCO2a
----------------------------------------------------------------------------
--------------------------------------------------------
LINRNPALFLQKTGKCLHLFKLFNTQRKYYFL--------------------------------------------
SLKGLETI------APLVRTV--EET------P-E---PI-P--TTI------------------EGT--IPSWI-
NGLFLRNGPG-----KF------EF-----GN---TYYNHWFDGMAMLHQFKI---Q-----KGD------V----
--TYMSRFLQS------DTYKKNSERDRI--VMSE-FG----TL-AMPDPCKNF---F------QRFLSRFEM---
-------I-----VTTEPTDNASVSFVKYK--------GD----------------------FYVST-ETN--
FMHKV----NLENLDTL--EK-------V----------D-------------------WS--KFIAV--NGA---
-------TAHPHYDP-------DGTTYN------------M--GNSYGRK------------GALYNIIKV-----
--------------------------------PPE---------KA--DIKETLQGAEVLCSISPANKS-HPS---
----YYHSFA-MSENYVVFIEQPV--KMDLLKI----VTC-NIR-GKA-LSQGI----------------------
---YWDSNQETV-FHLVDKH-------TG-------KVS-PVKYY--------------T-----K--
AISTFHQINAF----E--EDG---FLMIDLCCAD--GGQA-----------------ISN--YLIQNL--------
--------RKTGEALDEVYNTTERS----------------------FPRRFVLPL--------------------
----NLTSETPTNQNLN--------------------TRPFSKASCTKIS--------------------------
```

-PDKVFCQH---

-----------------------------EDLHGDDL------HEYGGL----EFPQINYSRCNTKPYRYFYGCG-
--FRHMVG---------------DSLLKVDLQDK--------T--------------------LK--------
VWQLKGFY--PSEPIFVPS---P-EAVE---EDEG--VILSVVLTP-------SQ-------DKGTFL---
LVLDAKTFEEVG--RANV--P--INI-PYGFHGIF---
---TATS---

>Medaka_BCO2b

GASMSDPEYKIPSPSQKISSVS--
DVHGLPCI------EKMVSSV--EET------P-E---PI-S--CFI------------------TGT--IPDWI-
KGNFLRNGPG-----KF------EI-----GN---QRFNHWFDGMALLHQFKI---S-----NGQ------V----
--TYKSRFLSS------DSYQSNQEHNRI--AVSE-FG----TV-TMPDPCKNF---F------QRFLSRFEL---
-------P--------KLSDNANVSFVTYK--------GD----------------------YYVST-ETN--
IMHKL----DPETLQSTEKKV------------------D-------------------WS--KFIAV--NGA---
-------TAHPHTEP-------DGTTYN------------M--GNSYTPK------------GAYYNIIRV-----
--------------------------------PAS---------KQ--AEGETLEGASVLCAIRSSEKT-KPS---
----YYHSFA-MSENYVVFIEQPI--KMDLLKI----ATG-KLT-GKS-ISDGF----------------------
---SWEPKSNTV-FHLIHKD-------TG-------KVS-PIKYF--------------A-----K--
ALSTFHQINAF----E--EGG---FLIIDMCASD--DGRA-----------------ITN--YKVQNL--------
--------RKSGEALDEVYNTLCRV----------------------FPRRFVLPL--------------------
----NIDSQTPFNQNLV-----------------HLANITATCVRIGSNK--------------------------
---V-FCTH---

-----------------------------EDLHGEEL------HHYGGL----EFPQINYGKYNTRPYRYFYGCG-
--FRHLVG---------------DTLIKMDLHRK--------S--------------------MK--------
VWEHAGLY--PSEPIFVPS---P-DATE---EDDG--VILSVVITP-------N----K---DKSTFL---
LVLDAKSFEELG--RAAV--S--VNI-PYGFHGAF---
----SATT--

>Medaka_BCO2c


```
RQTSSVSAVSSRERCP-------------------------------------------------KADDLESL---
---EHLVQSG--EET------P-E---PI-P--TTI------------------KGS--IPTWI-NGSFLRNGPG-
----KF------EF-----GE---DKYIHWFDGMAMMHRFHI---R-----DGS------V------
TYSSRFLRS------DSYVQNLEKNRI--VVSE-FG----TV-ASPDPCQSI---F------ARFFSYFQT-----
-----P--------QPTDNANVNFVKYK--------GD----------------------YYVST-ETN--
YMRRV----DPQSLETK--EK-------V----------D-------------------WS--QYIAV--NSA---
-------TAHPHYDR-------EGATYN------------M--GNSYGRN---------GKFYMDFDILKG-----
------------------------AKHHCDLSKIL---------------------STILCCHSQSQCL-KAS---
----SGFFLV-MSENYIVFIEQPI--KLNLLKF----MLY-KIQ-GKS-FHNIL----------------------
---SWEPQYGTV-FHLVHRR-------TG-------KKS-PVKYR--------------A-----A--
PMFTLHQINAY----E--DNG---FLVMDMCCGD--DGVV-----------------IGE--FTLENL-------
RR--------ESGQAMDEFYNSLCRN----------------------LPRRYVLPL-------------------
-----NVDENTPLDENLI--------------------TLHDCKATAMKTN-------------------------
--PEEVYMTH------------------------------------------------------------------
----------------------------------------------------------------------------
----------------------------------------------------------------------------
----------------------------------------------------------------------------
----------------------------------------------------------------------------
----------------------------------------------------------------------------
------------------------------EELHDDQL------LQYGGL----
EFPQINYAKYNGRPYRYMYACG---FGHVFA---------------DSLLKMDVHTK--------E----------
----------LK--------VWRYPGLY--PSEPVFVAS---P-GAAE---EDDG--VVLSVVVTP-------RK-
------EKSTFL---LILDARTFTELG--RAEV--P--VNI-PCGTHGVF--------------------------
------------------NEMG------------------------------------------------------
----------------------------------------------------------------------------
----------------------------------------------------------------------------
----------------------------------------------------------------------------
----------------------------------------------------------------------------
----------------------------------------------------------------------------
----------------------------------------------------------------------------
----------------------------------------------------------------------------
-------------------------------------------
>Stickleback_BCO2a
----------------------------------------------------------------------------
----------------------------------------------------------------------------
-----------------------------------------------------------GLPCI------
EKIVSSV--EDT------P-E---PI-G--TDI------------------TGS--IPVWI-RGSFLRNGPG----
-KF------EI-----GN---QKFNHWFDGMALLHQFKI---A-----EGR------V------TYKSRFLSS---
---DSFTANKEHNRI--VVSE-FG----TI-TMPDPCKNF---F------QRFLSRFEL----------P------
--KPTDNANVSFVTYK--------GD----------------------YYVST-ETN--IMHKV----
DPETLKTT--KK-------V----------D-------------------WS--KFIAV--NGA----------
TAHPHTEP-------DGTTYN------------M--GNSYGAK------------GASYNIIKV------------
-------------------------PPT---------KA--TAEDTLEGVSVLCSIPSSDKS-KPS-------
YYHSFA-MSENYVVFIEQPI--KMDLLKI----VTG-KLR-GKS-ISDSF-------------------------
LWDPTLNTI-FHLIHKQ-------TG-------TVS-AVKYL--------------A-----K--
PLSTLHQINAY----E--EDG---FLVLDMCASD--DGEA-----------------INN--YNVQNL--------
--------RKSGEALDEVYNTLCRV----------------------FPRRFVLPL--------------------
----NVDQETPYGRNLN-----------------PK--GSATSVRFAKNK--------------------------
---V-FCTH-------------------------------------------------------------------
----------------------------------------------------------------------------
----------------------------------------------------------------------------
----------------------------------------------------------------------------
----------------------------------------------------------------------------
----------------------------------------------------------------------------
-----------------------------EDLHGEDL------SEYGGL----EFPQINYTKYNTRPYRYFYGCG-
--FRHLVG---------------DSLIKMDLSGK--------R--------------------MK--------
VWEEPGLY--PSEPVFVPS---P-NATE---EDDG--VIMSVVITP-------N----K---DKSTFL---
```

LVLDAKTFAELG--RAAV--P--VNI-PYGFHGTF---
----SATA--

>Stickleback_BCO2b

MPPVKLETADDAKPVDNGKATEFITS--
ALKGLETI------EPLVRSV--EET------P-E---PI-S--AEV------------------QGT--IPSWI-
SGKLLRNGPG-----KF------EF-----GN---THYNHWFDGMAMLHQFKI---H-----EGQ------V----
--TYMSRFLDS------DAYKNNREKDRI--VMSE-FG----TL-AMPDPCKNV---F------QRFLSRFEM---
-------I-------TPTDNASVSFVKYK--------GD----------------------FFVST-ETN--
FMHRV----NPENLESL--EK-------V----------D-------------------WS--KFIAV--NGA---
-------TAHPHYDP-------DGTTYN------------M--GNSYGSK------------GALYNIIRV-----
--------------------------------PPE---------KT--DAKDTLQGTKILCSIVPANKS-HPS---
----YYHSFA-MSENYVVFIEQPI--KMDLMKI----VTC-KLR-GKA-MSDGI----------------------
---YWDPKLETV-FHVVNKK-------NG-------EVS-SVKYH--------------T-----K--
AISTFHQINAF----E--EDG---FLMIDLCCSD--NGQA-----------------INN--YLIQNL--------
--------RKSGDALDEVYNTMGRV----------------------FPRRFVLPL--------------------
----NVTGETPTDQNLN--------------------TRLSSSATCVKVS--------------------------
-NDKVFCQH---

-----------------------------EDLHGDEL------HQYGGL----EFPQINYGRNNTRPYRYFYGCG-
--FRHLVG---------------DSLLKMDLKDK--------T-------------------FK--------
VWQQKGFF--PSEPAFVAS---P-DAVE---EDEG--LILSVVLSP-------SQ-------DKGAFL---
LVLDSKSFEELA--RAYV--P--VNM-AYGFHGTF---
---DASA---

>Stickleback_BCO2c

MEHPIKCFSSHPPLEEERPAMSKTAEHQTNSMSSRQRCP-------------------------------------
-------KAKELENI------EALVRSV--EET------P-D---PV-P--TTI------------------QGT-
-IPAWI-NGSFLRNGPG-----KF------EF-----GK---DSYTHWFDGMALMHRFHI---C-----EGN----
--V------TYSSRFLRS------DSYVSNSEKNRI--VVSE-FG----TI-AMPDPCKNI---F------
QRFFSRFQV----------P-------KATDNASVNFVKYK--------GD----------------------
YYVST-ETN--YMRRV----DPQSLETK--EK-------V----------D-------------------WS--
KYIAV--NSA----------TAHPHYD-R------EGATYN------------M--GNSYGKS--------GFF--
--YNIIRV-------------------PPPEEKAAVEDS------ADL---------TG----------
ASVICSIPASEPR-KPS-------YYHSFV-MSENYIVFIEQPI--KLDLLKF----MLY-TIL-GKS-FHKVM--

-----------------------TWEPQYDTI-FHLVNRH-------TG-------QVS-EVQYR-----------
---A-----A--PMFTLHQINAF----E--DNG---LLVMDMCCGD--DGDV-----------------IGD--
FTLENL-------RR--------GSGEEMDKFYNSMCRN----------------------LPRRYVLPL------
------------------TVDGQTPLDQNLV------------------TLSNYKATAKKTKPQ------------
-----------------EVYMTH---

---EELYDDEL------LQYGGL----
EFPQINYDQHNGRAYRYFYSCG---FGHVFG---------------DSLLKMDVHTK--------E----------
----------LK--------VWRYPGLY--PSEPVFVAL---P-GATE---EDDG--VILSVIITP------RE--
------EKSTFL---LVLDAKTFTELG--RAEV--P--VNV-PYGTHGVF--------------------------
-------------------NRM--

>Sea_urchin_BCOa

MASERLSTKAYSQMHIEEHPEGVQATIKGTSSHPRDLYLDSDAYKAAMKENRIVYTEFGTAGFPDPCLNIFSRAMS
TFLTRL--KFTDNANVNWVRSGDQFYTIT-ETPL----
--I-QKIDPV-T--LEKEETVRFGNNPNKLSYFHNKGT--IPGWL-KGSLLRTGPG-----RF------EI-----
GE---SKYKHFFDGLALLHRFTF---D-----NGS------V------KYYSRYLDS------DAYKAAMKENRI-
-VYTE-FG----TA-GFPDPCLNI---F------SRAMSTFLT-----------------
RLKFTDNANVNWVRSG--------DQ----------------------FYTIT-ETP--LIQKI----
DPVTLEKE--ET-------V----------D-------------------IS--KIISV--FTG----------
TAHPHQS-A------DGTIYN------------L--GVNFGAR----------S---TYNLIKL------------
-------PPP---TE--VT-----NESI---------TK----------EASIALTISTTDT-YPS-------
YSHSFF-MTPNYFVLVEQPL--YINMIKL----ASL-AVM-GYS-FLDVL-------------------------
EFCPDKPTR-FHVVRRS-------DT-------VPV-ATKYI--------------T-----E--
AQFFFHQINAF----E--KEG---HIVIDMCSYK--DNEI-----------------MKR--LYLKTL---------
---------------EEEGLVDVGV----------------------QCKRYYLPI--------------------
--------------------------DVEGFQPGDTVSTLHKNETTAYME--------------------------
SEGTIFCKP---

-----------------------------FVLCDVGI----------------ELPRINYEKYNGQPYQYVYGVL-
--GTGEHD---------------NRIAKIDVEAG--------T--------------------SK--------
IWYEDGCY--PSEPVFIGT---P-EGQE---EDDG--VVLSTVLNF-------NE-------GGSPFL---
VVLDAKTFTELG--RAELPI---KDM-AYAIHGFF--
---AEGI---

>Sea_urchin_BCOb

--MANSDPNF------
GCLFNSV-QREY------P-T---PV-A--AQV------------------KGK--LPGWL-RGSLVRIGPG----
-LF------EV-----GD---TEYKHWFDGLALMHKFAF---H-----NGK------V------SYQNRFLRS---
---NAYKKAMKYNKI--ILSE-FG----TV-GIPDPCKNV---L------ERFATHFLP----------L------
--NVTDNNLINMYQLG--------DE----------------------VYTAT-ETH--TPRKI----
NLQDLSTEDQTI------------------D-------------------LL--KMFGM--MTA----------
SAHAQVD-R------DGCSYN------------M--GTSYLT---------GCF----
YNIIKFFPKSNSASSSSPSSPSLESSTTSTSESSTDT-----SSPS---------MS----------
AKLLCSIRARNPL-KPS-------YFHSFG-MTDNYIVFSEQTL--HINVAKL----ASS-QIL-GKP-VSSCF--
-----------------------EFDHNGTTL-FHLVEKQ-------TG-------RQM-TTRYE-----------
---T-----G--PLFGMHVINSY----E--EDG---HVVFDICSYD--DDEL-----------------LKK--
FYLDYL-------------------RHGDQDGKRHFPIT----------------------EVRRFVLPL------
-------------------RINKVTVGTNLV--------------------TLNYTSASAQLRP------------
---------------DGVVELTY---

---EVASDIGI----------------DMPCIN-
SRVNGRPYTYAYGTS-GKCKGDFM---------------NTIVKCNMRTK--------S-----------------
---CK--------TWHRPNHY--PSEPIFVGA---P-DAVD---EDEA--NYTEDYTTR----------------
DQQSFL---IVF--------------------AIV-FNGITGIM--------------------------------
------------AGANMS--

>Nematostella_BCOa

--MASSKY------
TGIVESC-IELD------R-----PI-Q--AKV------------------IGQ--IPPWL-NGTLLRNGPG----
-KF------EF-----GD---TSYNHWFDGQSLLHRFTI---H-----NGE------V------EYFNRFLRS---
---KAYVENTKANRI--TRSE-FG----TN-ALPDPCKNI---F------DRYFSYYFG----------G------
-DDITDNGLVNVVEIK--------EK----------------------MYAVT-ETP--FLTQI----
DPQSLDVQ--GRLDAS---K----------D-------------------MK--DPHPL--HSS----------
IAHPHQE-S------DGTFYN------------F--GHTRGRF----------A---KFNIYMV------------
-------PPK---SKENTT-----EDPF---------DG----------AKVLCSIDAKAG---ET-------
YVHSFG-MTENFFILLENPY--FMSVPKV----LTK-NVF-GWA-FSKCL-------------------------
YWDPKCPTR-IHVMCKK-------TG-------EEM--ATFT--------------T-----D--
PVFVFHHINAF----E--NKEEIVEIVVDVVGYK--DTKL-----------------VDD--LYLHEL--------
--------KRRLKSEDQNEGRVSLG----------------------EFRRYRLPI--------------------
--PNKKIP--------------------------
-----STNP---

-----------------------------EVQHHVPK--------
FEVLYSNLELPQINYERCNGRKYTYVFALTAANSSVMDT-----------------LVKINTVTK--------E--
------------------TK--------TWGNPGLV--ASEPVFVPK---P-DAED---EDEG--VVLSAVIDV--
-----V--------NGNTFL---LLLDGKTFEELG--RAEV--S--VMM-PMNIHGRF------------------
--------------------------VPKS--

>Nematostella_BCOb

--MTTTAGNF------
VEIFTSH--SEL------E-L---PV-Q--AKI------------------TGQ--IPPWL-SGTLIRNGPG----
-KF------EF-----GE---FEYNHIFDGPALLYRFTI---D-----NGK------V------EYFNKFLRS---
---KSFLENTKANRI--THME-YG----TN-AVPDPCKSI---F------HRYFSYYFG----------S-----
DKEKITDNCLVNVIELK--------KR----------------------FYAVS-ELP--VLWQI----
DSQSLDVI--GK-------V----------D-------------------VS--TDMDD--PLD---NS-----
LAHPHEE-P------DGTVYN------------Y--GIKRGRF----------T---KYNIYKV------------
-------PPR---SKESPL-----EKTM---------AG----------AQVICSLSPTKA---EA-------
YVHSFG-MSESYFILLENPF--FFSIPRF----LAR-SFF-GWT-LDKCF-------------------------
YWDPTQLSR-IHVLCRK-------TG-------KEL--AVFT--------------T-----D--
PMFVFHHINAF----E--KNG---EIILDVVAYP--DGDI-----------------MNG--LLIQDM--------
--------RDFCNKKGRTEHQIPAG----------------------QFRRYHLPN--------------------
--PAERGR--------------------------
-----STSS---

-----------------------------EE----PHHIFNFEVLYDKM----ELPRINYEHCNTKEYTYVYGLT-
NTSSSLLY---------------DEIVKINTFSK--------E--------------------VK--------
TWRFPNHF--PSEPVFVPK---P-GGVR---EDEG--VVLSMVIDT-------A--------NGNTYL---
LVLDAQTFDELG--RATV--P--EIG-ASTIHGRF---
---VKN--

>Nematostella_BCOc

---M------
EDVYQTF-PEQP------------TAQE--ASI------------------SGN--VPAWI-KGNLLRNGPG----
-VY------EI-----GK---EHYNHWFDGLAVLHNYKI---E-----EGK------V------TYNSRYLRS---
---HAFDEAQSKNGI--VYAE-FA----TP-IPPDPCKNI---F------ARFFSYFVP-----------------

```
-PKRTDNCSVNVVALK--------GK----------------------TYAVS-DSP--FLIGF----
DENSLQVL--SSYNMRK-------------D-------------------LQ--GPIRM--FSL----------
TPHPHED-E------HGYVYN------------V--AVTMDKG----------T---RFNIVKI------------
-------PPDPKRSLPGEST----CHPM---------EG----------LKVLASFQPKNKL---C-------
YYHSFA-MTPNYFVFIENPF--VVNVFAL----LTM-KVK-GRS-FHDCM-------------------------
KWDATQPSR-FYLVERK-------SG-------KCI--ARYD--------------A-----D--
CFFSFHHVNAF----E--SDA---DVFVDIVCYP--DARI-----------------VYQ--YYLHHL-------
RT-----------KPEHEISKGYADP----------------------ALRRYRIPI-------------------
-----------------------------------PTNHAPKSKRKKSFQQ-------------------------
------YKMP------------------------------------------------------------------
----------------------------------------------------------------------------
----------------------------------------------------------------------------
----------------------------------------------------------------------------
----------------------------------------------------------------------------
----------------------------------------------------------------------------
------------------------------KYSDDRDY-----KLLYYGI----
ELPQINYQFANGKPYRYVYGVG-PRRRGDFL---------------NQIIKVDVLAK--------N----------
----------AK--------FWYEQDCY--PSEPVFIAA---P-RAEE---EDEG--CVMSAVVGT-------K--
------GKKSFL---LFLDGKTFKELA--RAVVDCP----I-AYSLHGMF--------------------------
----------------------------------------------------------------------------
----------------------------------------------------------------------------
----------------------------------------------------------------------------
----------------------------------------------------------------------------
----------------------------------------------------------------------------
----------------------------------------------------------------------------
----------------------------------------------------------------------------
----------------------------------------------------------------------------
-------------------------------------------
>Fugu_RPE65a
----------------------------------------------------------------------------
----------------------------------------------------------------------------
----------------------------------------------------------MISKFW------
NFVVRPK-------------N---PF----SIV-----------------CKCR--IPPFL-KGSLLRLGPG----
-LF------EI-----GD---EPFYHLFDGQALMHKFDF---K-----NGQ------V------TYFRKFIRT---
---DAYVRAITEKRV--VITE-FG----TF-AYPDPCKNI---F------SRFFSYFKG----------V------
--EVTDNCLVNVYPIG--------ED----------------------YYAVT-ETN--YITKV----
NVETLETL--KK-------V----------D-------------------MC--NYVNI--NGV----------
TAHPHIEK-------DGTVYN------------I--GNCMGKG--------ASL---AYNIVRT------------
-------------------------PPT---------QKGPDKSDPIEKSKVVVQFPSAERF-KPS-------
YVHSFG-MSENYFVFVETPV--KINLLKF----LSAWSIR-GSN-YMDCF-------------------------
ESNESQGTL-FHIAKKD-------PG-------EYI-DVKFK--------------G-----A--
AIGMFHHINTF----E--DQG---FIVFDLCSWK-GFEFV-----------------YNY--LWLANL--------
--------RANWDEVKKAAMMAPQP----------------------EVRRYVIPL--------------------
-----DVHKEEQGKNLV--------------------SLPYTTATATMHS--------------------------
-DGTVWLEP-------------------------------------------------------------------
----------------------------------------------------------------------------
----------------------------------------------------------------------------
----------------------------------------------------------------------------
----------------------------------------------------------------------------
----------------------------------------------------------------------------
-----------------------------EVLFSGPRQAF-------------EFPQINYQKFSGKNYTYAYGLG-
--LNHFIP---------------DRICKLNVKTK--------E--------------------TW--------
VWQEPDSY--PSEPLFVQT---P-EGVD---EDDG--VLLTIVAAP-------GS-------QRPAYL---
LILNAKDLSEVA--RAEVECS----I-PVTFHGMY-----------------------------------------
---KH-----------------------------------------------------------------------
----------------------------------------------------------------------------
----------------------------------------------------------------------------
```


>Fugu_RPE65b

---FVVS---VEHPAAGY------
KKIFETV--EEL------N-E---PI-A--ATI------------------SGV--IPTWL-SGSLLRMGPG----
-LF------EV-----GD---QPLHHLFDGQALIHKFDL---K-----DGR------V------TYYRKFIRT---
---DAYVRAMTENRV--VITE-FG----TA-AYPDPCKNI---FSSPFCLSRFFTYFKG----------I------
--EVTDNCLVNIYTIG--------ED----------------------FYAVT-ETN--FITKV----
DPDSLETL--KK-------V----------D-------------------LS--KYVSV--NGI----------
TAHPHKDP-------DGTVYN------------I--GNCFGKN--------MSL---AYNIVKI------------
-------------------------PPA---------PK--DSTDAFEKSQVMVQLPSSERL-KPS-------
YIHSFG-MTENYFVFVEPPV--KINLLKF----LSAWSVR-GAT-YMDCF-------------------------
ESNETLGTW-FHLATKE-------PA-------EYLSSHKFR--------------T-----S--
AFNVFHHINTY----K--DQD---FIVVDLCTWK-GHDFV-----------------YNY--LYLANL--------
--------RQEWEEVKKAAMRAPQP----------------------EVRRYVLPL--------------------
-----DIHREDMGKNLV--------------------SLSYTTATAVLRS--------------------------
-DGTIWLEP---

-----------------------------EVLFSGPRHAF-------------EFPQINYSEYSGKMYRFAFGLG-
--LNHFIP--------------DRIMKLNVQTK--------E--------------------MR--------
EWREEECY--PSEPLFVAT---P-GATD---EDDG--VLLSVVVKP-------GA-------DRPGSL---
LVLDAKTLTEVG--RAEVS----VNI-PVTLHGTY---
---T--

>Pufferfish_RPE65a

--MVSRFEHP------
AGSYKKI-FETC--------EELAEPVP--ATV------------------TGR--IPPFL-KGSLLRLGPG----
-LF------EV-----GD---EPFYHLFDGQALMHKFDF---K-----NGQ------V------TYYRKFIKT---
---DAYVRAITENRV--VITE-FG----TF-AYPDPCKNI---F------SRFFSYFKG-----------------
-VEVTDNCLVNVYPIG--------ED----------------------YYAVT-ETN--YITKV----
NTDTLETL--KK-------V----------D-------------------MC--NYVNI--NGV----------
TAHPHIEK-------DGTVYN------------I--GNCMGKG--------ASL---AYNIVRT------------
-------------------------PPT---------QK--DKSDPIEKSKVVVQFPSAERF-KPS-------
YVHSFG-MSENYFVFVETPV--KINLLKF----LSAWSIR-GSN-YMDCF-------------------------
ESNENQGTL-FHIAKKN-------PG-------EYI-DLKFK--------------G-----A--
AIGMFHHINTF----E--DQG---FIVFDLCSWK-GFEFV-----------------YNY--LWLANL--------
--------RANWEEVKKAAMMAPQP----------------------EVRRYVIPL--------------------
-----DVHKEEQGKNLV--------------------SLPYTTATATMHS--------------------------

-DGTIWLEP---

-----------------------------EVLFSGPRQAF-------------EFPQINYKKFSGKNYTYAYGLG-
--LNHFIP---------------DRICKLNVKTK--------E--------------------TW--------
VWQEPDSY--PSEPLFVQT---P-DGVD---EDDG--VILTIVAAP-------GS-------QRPAYL---
LILNAKDLSEVA--RAEVECS----I-PVTFHGMY---
---KH---

>Pufferfish_RPE65b

--IMARR-----
--VEHPAGGY------
KKIFETV-EELD--------E---PV-A--AKI------------------SGT--LPAWL-SGSLLRMGPG----
-LF------EV-----GD---EPFRHLFDGQALIHKFDL---K-----EGH------V------TYHRKFIRT---
---DAYVRAMTENRV--VITE-LG----TA-AYPDPCKNI---F------SRFFTYFKG-----------------
-VEVTDNCMVNIYTIG--------ED----------------------FYAVT-ETN--FITKV----
DPDSLETL--KK-------V----------D-------------------LS--KYLSV--NGV----------
TAHPHKDA-------DGTVYN------------I--GNCFGKN--------MSL---AYNIVKI------------
-------------------------PPA---------QK--DSLEPFEKARVVVQLPSSERL-KPS-------
YIHSFG-MSDNYFVFVEPPV--KINLIKF----LSAWSIR-GAT-YMDCF-------------------------
ESNETLGTW-FHLATKE-------PA-------EYLSNYKFR--------------T-----S--
AFNIFHHINTY----E--DQG---FMVVDLCTWK-GHEFV-----------------YNY--LYVANL--------
--------RQEWEEVKKAAMKAPQP----------------------EVRRYVLPL--------------------
-----DISSEDLGKNLV--------------------SLSYTTATAVLRR--------------------------
-DGTIWLEP---

-----------------------------EVLFSGPRLAF-------------EFPQINYSEYSGKMYHFTYGLG-
--LNHFIP---------------DRIMKLNVQTK--------E--------------------TQ--------
EWREEECY--PSEPLFVPT---P-GATD---EDDG--VLLSVVVKP-------GA-------GRPGFL---
LVLDAKTLSELG--RAEV--S--VNI-PVTLHGMF---
---KP---

>Medaka_RPE65a

---FINR---FEHPAGSY------
KKIFETC--EEL------A-E---PL-P--TTV------------------TGR--IPSYL-RGSLLRLGPG----
-LF------EV-----GD---EPFYHLFDGQAFMHKFDF---K-----NGQ------V------TYFNKFIRT---
---DAYVRAITEKRV--VITE-FG----TF-AYPDPCKNI---F------SRFFSYFKG----------V------
--EVTDNCLVNVYPIG--------ED----------------------FYAVT-ETN--YITKV----
NTDTLETL--KK-------V----------D-------------------MC--NYVNI--NGV----------
TAHPHIEA-------DGTVYN------------I--GNCMGKG--------ATL---AYNIVKI------------
-------------------------PPT---------QK-VDKSDPIEKSKVVVQFPSAERF-KPS-------
YVHSFG-MSENYFVFVETPV--KINLLKF----LSAWSIR-GSN-YMDCF-------------------------
ESNEAQGTN-FHIAKKS-------PG-------EYI-DLKFK--------------G-----A--
AIGMFHHINTY----E--DDG---FLVVDLCAWK-GFEFV-----------------YNY--LWLANL--------
--------RANWDEVKKAAMMAPQP----------------------EVRRYVIPL--------------------
-----DVHKEEQGKNLV--------------------SLPYTTATATMHS--------------------------
-DGMVWLEP---

-----------------------------EVLFSGPRQAF-------------EFPQIDYKRHGGRNYTYAYALG-
--LNHFIP---------------DRICKLNVKTK--------E--------------------TW--------
VWQEPDSY--PSEPLFVQN---P-DGVD---EDDG--ILLTIVAAP-------GA-------QKPAYL---
LILNAKDLSEVA--RAEVD----YSI-PVTFHGMY---
---KP---

>Medaka_RPE65b

HNPEASSQSLSNGRGCLELISEKKKLWFKSKS--
KSLQATSY------KKIFETV-EELD--------E---PI-P--AQI------------------SGV--IPSWL-
GGSLLRMGPG-----LF------EV-----GD---EPFHHLFDGQALMHKFDL---R-----NGQ------V----
--TYNRKFIKT------DSYVRAMTENRV--VITE-FG----TI-AYPDPCKNI---F------SRFFSYFKG---
---------------IEVTDNCLVNIYPIG--------ED----------------------FYAVT-ETN--
YITKV----DPDSLETL--EK-------V----------D-------------------VC--KYLSV--NGL---
-------TAHPHIEA-------DGTVYN------------I--GNCFGKD--------MSL---AYNIVKI-----
--------------------------------PPA---------QK--DKSDPIEKSQIVVQLPSSERL-KPS---
----YVHSFG-MTNNYFVFVEQPV--KINLLKF----LSAWSIR-GTT-YMDCF----------------------
---ESCESMGTW-FHLTTKN-------PA-------AYMSSHKFR--------------T-----S--
AFNLFHHINTY----E--DQG---FIVVDLCTWK-GHEFV-----------------YNY--LYLANL--------
--------REEWEEVKKAASRAPQP----------------------EVRRYVLPL--------------------
-----DIYKEEQGKNLV--------------------SLSYTTATAVLCS--------------------------
-DGTIWLEP---

-----------------------------EVLFSGPRQAF-------------EFPQINYSQCCGKKYFFAYGLG-
--LNHFIP---------------DRIVKLNVQTK--------E--------------------TW--------
VWQEEDCY--PSEPIFVAT---P-GATQ---EDDG--VLLSIVVKP-------GT-------ERPGFL---

LVLDAMKLTEVA--RAEV-----NAIIPVTLHGLY---
---KP---

>Stickleback_RPE65a

---MVSR---FEHPAGGY------
KKMFETC--EEL------S-E---PV-P--ATV------------------IGR--IPSFL-KGSLLRLGPG----
-LF------EV-----GD---EPFYHLFDGQALMHKFDF---K-----NGQ------V------TYYRKYLKT---
---DAYVRAITEKRV--VITE-FG----TF-AYPDPCKNI---F------SRFFSYFKG----------V------
--EVTDNCLVNVYPIG--------ED----------------------YYAVT-ETN--YLTKV----
NTDTLETL--KK-------V----------D-------------------MC--NYVNI--NGV----------
TAHPHIER-------DGTVYN------------I--GNCMGKG--------ATL---AYNIVRV------------
-------------------------PPT---------QK--DKSDPIEKSKVVVQFPSYERF-KPA-------
YVHSFG-MSENYFVFVETPV--KINLLKF----LSAWSIR-GSN-YMDCF-------------------------
ESNESQGTL-FHIARKE-------PG-------EYI-DHKYK--------------G-----A--
AIGMFHHINTY----E--DQG---FIVVDLCGWK-GFEFV-----------------YNY--LWLANL--------
--------RANWEEVKKAAMMAPQP----------------------EVRRYVIPL--------------------
-----DIHKEEQGKNLV--------------------SLPYTTATATMHA--------------------------
-DGTIWLEP---

-----------------------------EVLFSGPRQAF-------------EFPQINYKKYGGKNYTYAYGLG-
--LNHFIP---------------DRICKLNVKTK--------E--------------------TW--------
VWQEPDSY--PSEPLFVQT---P-DGID---EDDG--VLLTIVVSP-------GS-------QRPGYL---
LILNAKDMSEIA--RAEVDCP----M-PVTFHGMY---
---KP---

>Stickleback_RPE65b

---MFSH-
--VEHPAAGY------
KKIFETV-EELN--------E---PI-P--ARV------------------IGV--LPSWL-GGSLLRMGPG----
-LF------EV-----GG---EPFNHLFDGQALIHKFDL---K-----NGQ------V------TYYRKFIRT---
---DTYVRAMTENRV--VITE-FG----TA-AYPDPCKNI---F------SRFFTYFKG-----------------
-IEVTDNCLVNICPIG--------ED----------------------FYAVT-ETN--YITKV----
DPDSLETL--KK-------V----------D-------------------LC--KYVSV--NGV----------
TAHPHTDA-------DGTVYN------------I--GNCFGKN--------FSL---AYNIVKI------------
-------------------------PPP---------QE--DKSDPLERAQVVLQLPSSERL-KPS-------
YLHSFG-MTDNHFVFVEQPV--KINVLKY----LSAWSVR-GTT-YMDCF-------------------------

ESNDSMGTW-FHLATKD-------PA-------DYLSSYKFR--------------T-----S--
AFNIFHHINAY----E--DQG---FIVVDLCTWK-GHDFV-----------------YDY--LYLANL--------
--------KGEWEEVKKAAMRAPQP----------------------EVRRYVLPL--------------------
-----DIHREEQGRNLV--------------------SLSYTTATAALRS--------------------------
-DGTIWLEP---

-----------------------------EVLFSGPRQAF-------------EFPQINYSLCRGKKYSFAYGLG-
--LNHFIP---------------DRIVKLNVQTK--------E--------------------TW--------
VWQEDECY--PSEPLFVPT---P-AATE---EDDG--VLLSIVVKP-------GA-------ERPSFL---
LVLDAVKLTELA--RAEVN----TII-PVTLHGTY---
---KP---

>Atlantic_cod_RPE65a

---MVSR---IEHPAGSY------
KKIFETC--EEL------S-E---PI-P--AVI------------------TGR--IPSFL-RGSLLRLGPG----
-LF------EV-----GD---EPFYHLFDGQALMHKFDF---K-----NGQ------V------TYFRKFIKT---
---DAYVRAITEKRV--VITE-FG----TF-AYPDPCKNI---F------SRFFSYFKG----------V------
--EVTDNCLVNVYPIG--------ED----------------------YYAVT-ETN--FITKV----
DTDTLETT--KK-------V----------D-------------------LC--NYVNV--NGL----------
TAHPHIER-------DGTVYN------------I--GNCMGKG--------ATL---AYNIVRM------------
-------------------------PPA---------QK--DKSDPIEKSKVVVQFPSAERF-KPS-------
YVHSFG-MSENYFVFVETPV--KINLLKF----LSAWSIR-GSN-YMDCF-------------------------
ESNETLGTW-FHLATKD-------PG-------EYI-DHKFR--------------A-----A--
AIGLFHHINTY----E--DDG---FVVVDVCAWK-GFEFV-----------------YNY--LWLANL--------
--------RANWDEVKKAAMMAPQP----------------------EVRRYVIPL--------------------
-----DIHKEEQGKNLV--------------------SLPYTTATAVMHS--------------------------
-DGTIWLEP---

-----------------------------EVLFSGPRQAL-------------EFPQINYEKYAGKNYTYAYGLG-
--LNHFIP---------------DRIVKLNVKTK--------E--------------------TW--------
VWQEPDSY--PSEPLFVQT---P-DGID---EDDG--VLLTIVVAP-------GA-------QRPGYM---
LILNARDLSEIA--RAEVD----CTM-PVTFHGMY---
---KP---

>Atlantic_cod_RPE65b

--VEHPAAGY------
RKIFETV--EEL------N-E---PL-P--TEV------------------TGV--LPSWL-SGSLLRMGPG----
-LF------EV-----GA---EPFHHLFDGQALIHKFDL---R-----EGH------V------TYHRRYIRS---
---DAYVRAMAEKRV--VITE-FG----TA-AYPDPCKNI---F------SRFFTYFQG----------I------
--EVTDNCLVNIYPIG--------ED----------------------FYAVT-ETN--FITKV----
DPDSLETL--KK-------V----------D-------------------LC--DYLSV--NGV----------
TAHPHTDA-------DGSVYN------------I--GNCFGKN--------MSL---AYNIIKI------------
-------------------------PPP---------QK--DGSDPLKRSEVVVQLPSSERF-KPS-------
YVHSFG-MTENHFVFVEQPV--KINLLKF----LSSWSLR-GTN-YMDCF-------------------------
ESNEYIGTW-FHVATRD-------PA-------AYLSSHKFR--------------T-----S--
AFNVFHHINAY----E--DQG---FIVVDLCTWK-GSDFV-----------------YNY--LYLANM--------
--------RAEWDKVKESAMRAPQP----------------------EVRRYVLPL--------------------
-----DIYREEQGKNLV--------------------SLSYTTATAVLRS--------------------------
-DGTVWLEP---

-----------------------------EVLFSGPRQAF-------------EFPQINYSRCSGKNYRYVYGLG-
--LNHFIP---------------DRIVKLNVRTK--------D--------------------TR--------
LWQEEDSY--PSEPLFIPT---P-EAID---EDDG--VLLSIVVKP-------GA-------ERPSFL---
LVLDARQMTELA--RASV-----NTIIPVTLHGTY---
---KPR--

>Atlantic_cod_BCMO1

--LVHDGVLK------
YPVPPNN-IEER------P-E---PY-T--AEV------------------KGS--LPDWL-TGTLLRNGPG----
-MF------TV-----GE---TTYNHWFDGMALMQSFTF---K-----DGQ------V------IYRSRYLQG---
---DTYNANMAAKRI--VVSE-MG----TM-AYPDPNKNF---I------VKAITFLNH----------T------
VPDFTDNGASNFIRYG--------NE----------------------YFATS-ETN--YIRKI----
DPFTLATQ--EK-------V----------D-------------------YL--KYIAV--NMV----------
TSHPHYD-K------EGNTYN------------I--GTSIAEK--------GKT---KYTLFKV------------
---------PSAPETETGK------SPL---------KK----------AEVLCTVPCRSLL-SPS-------
YYHSFG-MTDNYFIFIEQPF--KLDILKM----ATA-YMR-GVN-WASCL-------------------------
KHCPEENTL-IHLIDRK-------TG-------KEV-ATKYF--------------A-----G--
QMVIYHHVNAF----E--ENG---HVIFDVIGYD--DCSL-----------------YDM--FYLQKQ-------
KD--------------KPESKGWSKP----------------------VYRRYALPL-------------------
---HVDSGLQGAAGQNLV--------------------KLLTTATAVKEAD-------------------------
---GKLLCMP--

------------------------------EV----LCEGL-------------ELPRIN-
YDFNGKKHRFVYAACTEESAVATE-----------------LGKLDTETK--------E-----------------
---IV--------RWGEDNCW--PSEPVFIPR---P-NAES---EDDGGRVVLASVHRP----------------
GKLGFI---LVLDGKTFKEVA--RAYMN----NEL-HRDMHGYF--------------------------------
------------IPQ---

>Atlantic_cod_BCO2a

SIHRPQIKKPESNMASPCRFT---
DVHGLPSI------ENLVRSA-EHTP------------EVIS--TRV------------------TGT--IPDWI-
TGNFLRNGPG-----KF------EY-----GN---QTFNHWFDGMALLHQFKI---A-----KGR------V----
--TYRSRFLSS------DCYQANNENNRI--MVSE-FG----TV-ALPDPCKNF---F------HRFLSRFEL---
-----------PIPSAEATDNASVSFVTYK--------GD----------------------YYVST-ETT--
FMHKV----DPETLETT--GK-------V----------D-------------------WS--KFIAV--NGA---
-------TAHPHNDP-------DGTTYN------------M--GNSYTPK------------GAFYNIIRV-----
--------------------------------PPT---------KS--SPEETLEGATVVCSIPSTDKT-KPS---
----YYHSFA-MSKNYVVFIEQPI--KINLLKV----VTG-KLT-GKG-ISDSV----------------------
---YWEPKLDTI-FHLIDKK-------TG-------KLS-LVKYH--------------T-----K--
AMSTFHQINAF----E--ENG---FLLMDMCVSD--DGQA-----------------INN--YMVQNL--------
--------RKSGEALDEVYNTMCRV----------------------FPRRFVLPL--------------------
----NVGTDTPCGTDLN-----------------KETDSTATAIKISNNK--------------------------
---V-FCTH---

-----------------------------EDLHGEDL------HFYGGL----EFPQINYAKYNTRPYRYFYGCG-
--FRHLVG---------------DTLIKMDIHSK--------Q--------------------MK--------
VWERPGLY--PSEPVFVPS---P-DATE---EDDG--VVMAVVITP------------NKKQDKSTFL---
LVLDAKTFKELG--RAEV--P--VNI-PYGFHGTF---
----NAT---

>Atlantic_cod_BCO2b

DTMLSDGVGEEQRASG---VLKGLECI-
-----APLVRSV-EETL--------E---PI-P--TAV------------------QGT--IPSWI-
RGNLLRNGPG-----KF------EF-----GN---THYNHWFDGMALLHQFKI---Q-----EGE------V----
--TYRSRFLQS------DSYRKNSEKDRI--MVSE-FG----TL-ALPDPCQNF---F------QRFLSRFEM---

---------------IKPTDNASVSFVKYK--------GD----------------------YYVST-ETN--
LMHKV----DPETLESI--EK-------V----------D-------------------WS--KFVAV--NGA---
-------TAHPHCDP-------DGTVYN------------M--GNSYGSK------------GALYNIVRV-----
--------------------------------PPE---------KK--DSTDSLQGATVLCSIMPANKS-HPS---
----YYHSFA-MSENYVVFIEQPI--KMDLLKI----VTC-KLR-GKG-LSEGI----------------------
---YWDPKQDTV-FHLVDKK-------TG-------QAS-SVRYH--------------T-----K--
AISTFHQINAF----E--EDG---FLMLDMCCSE--DGQA-----------------INN--YLIQNL--------
--------RKSGDALDQVYNEMTRV----------------------FPRRFVLPL--------------------
-------------------------------------GVTSDTQSNQNLN------------------------
TRPDSLATCLK---

TGQDKVLCQHEDLHGDDLLDYGGLEFPQINYGQNNTRPYRYFYGCG---FRHLVG---------------
DSLLKMDLKDK--------S--------------------LK--------VWQQQGFY--PSEPVFVPS---P-
DAVD---EDQG--VILSVVLTP----------------SQ------------------------------------
----

>Atlantic_cod_BCO2c

--ETPQPVST------------
------------------HI-------------------------KGT--IPSWI-NGSFLRNGPG-----KF---
---EF-----GK---DKYSHWFDGMAMMHRFYV---K-----DGD------V------TYNSSFLRS------
DSYTKNSEKDRI--VVSE-FG----TI-AMPDPCKNI---F------ARFFSRFKL----------------
PHAEATDNASVNFVKYK--------GD----------------------YFVST-ETN--YMRRV----
DPDSLETK--EK-------V----------D-------------------WT--QYVAV--NSA----------
TAHPHYG-R------DGATYN------------M--GNSYGKS--------GKSVCXXPGFFYN------------
-------------------------------------NL--------YGAKVICSIPASEPR-RPS-------
YFHSFV-MSENYIVFIEQPI--KLDMLKA----MLY-QVQ-GKS-FNKIM-------------------------
TWDPKCDTI-FHLVNRH-------TG-------EFS-STR-S--------------T-----Q--
PMFTLHQINAY----E--EGG---CLVMDMCCGD---DQF-----------------
YNSMCRNLPRRYVIPLTVDEDTPMDQNLVNLSLCTATAKKTGPG------------ESLSLPNDGSLLKRKMFPL-
---SPLAQV-------
------------------------YLTY--

--EELFNDEL------LQYGGL----
EFPQINYAENNGRPYRYVYACG---FGHVFS---------------DSLLKMDVRTK--------E----------
----------LK--------AWRHHGLF--PSEPVFVPA---P-NATE---EDDG--VVMSVIINP-------K--
--E---DKSTFL---LVLDAKTFTELG--RAEV--P--VNI-PYGTHGVF--------------------------
-------------------NDMNQ--

>Lancelet_BCOLa_1

MAFYTVSACVVLFATLSV--
TTAKPDGV------LTLFQTN-TEEV------------QHVP--MKY------------------DSKNAVPKWL-
EGTLVRNAPA-----LF------EL-----GG---RSAIHVFDGFAKLHSVEI---G-----PHL------V----
--NFSAAFLDS------SAYTRSKKANRYAPALTF-FG----VD----PPFSEL----------ERLDAFRYP---
------------------YDNTDVNVWKYGH-------GE-------------------DSVYAALT-DGW--
VYAKF----DSDTLATI--GVSIPSVSGI----------K-------------------MG--HVVQS--------
-------CAHPIVEPG------TTHNIN------------Y--IQIPGMI------RGQKQ---DHYVVRI-----
--------------------------------KDL---------DT----------YEVLATFQTD----RAS---
----YMHSFA-LTENYAIFFAQPA--YFDFIKI----VET---------AMVSI----------------------
---DWVPEDKTT-IILANIK-------TG-------EIQ---RLQ--------------T-----D--
AIFFTHHVNAY----ET-ANG---TIVADVVQFE-EGFAI-----------------FAD--YKLPQL-------
RN-------------ITSLHDKPAKS----------------------RLYRYTIDL-------------------
--ANKTVQTKTFR-------------------------
------SKSP--

------------------------------MEDFLHKI----------------EFPIIN-
ENYRTKHYCYVFGVI-IDFSPSNP-----------LNGSFAMVKKDLCTP--------GN----------------
---DL--------YWYHPNHY--VSEPSFVAD---P-DGKA---EDDG--VLLSAVFDA-------E--------
LGKSYL---LILDCKTMKAIN--TAYM--P--TYI-PFGFHGRF--------------------------------
----

>Lancelet_BCOLa_2

---FGK----DWPVSKKANRYAPA----LTF---
---------------FGVDPPFSELERLDAFRY-------------------------PY----------------
----
DNTDVNVWKYGH-------GE-------------------DSVYAALT-DGW--VYAKF----DSDTLATI--
GVSIPSVSGI----------K-------------------MG--HVVQS---------------CAHPIVEPG---
---TTHNIN------------Y--IQIPGMI------RGQKQ---DHYVVRI------------------------
-------------KDL---------DT----------YEVLATFQTD----RAS-------YMHSFA-
LTENYAIFFAQPA--YFDFIKI----VET---------AMVSI-------------------------
DWVPEDKTT-IILANIK-------TG-------EIQ---RLQ--------------T-----D--
AIFFTHHVNAY----ET-ANG---TIVADVVQFE-EGFAI-----------------FAD--YKLPQL-------
RN-------------ITSLHDKPAKS----------------------RLYRYTIDL-------------------
--ANKTVQTKTFR-------------------------

```
------SKSP------------------------------------------------------------------
----------------------------------------------------------------------------
----------------------------------------------------------------------------
----------------------------------------------------------------------------
----------------------------------------------------------------------------
----------------------------------------------------------------------------
------------------------------MEDFLHKI----------------EFPIIN-
ENYRTKHYCYVFGVI-IDFSPSNP-----------LNGSFAMVKKDLCTP--------GN----------------
---DL--------YWYHPNHY--VSEPSFVAD---P-DGKA---EDDG--VLLSAVFDA-------E--------
LGKSYL---LILDCKTMKAIN--TAYM--P--TYI-
PFGFHGRFFAPLPHKAVVDGRGDLRNRRCSRGDQAAQAAGHLAGVHGTAPSRTTQQSSSSGDDPQYSTIPDEYYNR
QNTGTTNDYSQIPDEYYNYYNTRPGAQHPYWEIPDEYYNYYNTRPGAQHPYWEIPDEYYNYYNTRPGAQHPYWEIP
DEYYNRYSTYPPTRRVPQDEKDYSVRFNSTAAEVVLPSSTRLGGKHPSYDTAPQVWRDPQNYQIPARGRDTNIRAQ
GMPVAGNSIGPRYMGLIDNRRRLSYPLTLRVPQDNKDYSSRINSTAAEVALPSSTRLGGKHPSYDTAPQVWRDPQN
YQIPARGRQTNIRAHRMPLKGNSLHRYMGLFGNYKYKKRRLSYPLTLRVPEEHENYSAGFNATSTAAEVVLPSSTR
LGGKHPSYDTAPQVWRDPQNYQIPARCRQTNIRAQGMPVSGSSSDPRYMGLIGNYSKTMRAAKFRMQAMPLYNPLA
HHSRHQRSTKYQASIKAEPTENKNHPNLS-----------------------------------------------
----------------------------------------------------------------------------
----------------------------------------------------------------------------
-
>Lancelet_BCOLb
----------------------------------------------------------------------------
-------------------------------------------------------------
MAWWTTIAAWLLCCVFPGQPTTA------------------------------------------------
TVNAKDGV------LYCFTEN-LKEF--------H---KV-P--MSY------------------TSSRPVPQWL-
SGTLVRNAPS-----QF------SV-----GR---RSVVNYFDGFAKLHSLDI---N-----QHS------V----
--NFSASFLKT------GVYNRSIEANDI--L----PG----PTFMGVDPPFSL---L------ERLRALSSP---
-------G-----------DNAIINVWNFG--------GD----------------------FAALT-DAW--
VFAQF----DLDTLDTI--GVSVPDPILE----------N-------------------RG-CQTTEV--YMS---
-------CAHPMVEPG------TGHSIN------------F--VMKVSLF------PGQRD---TFRVIRI-----
--------------------------------KDL---------RT----------IETLASFEID----KIW---
----YMHSFS-LTENFAIFFAQPC--YYDFIRF----FT------TVE-AQHSI----------------------
---YWAPDDGMK-IYVVNLK-------TG-------NVT---TLH--------------T-----
EAAAAIYTHHVNAY-------ETGDG-RVVNDVVILTNTKAFT-----------------TGLARSRLTNI-----
------------------TAIREMQSPT----------------------RLFRYILDL-----------------
-----------------------------------------------KTGKVE-----------------------
---VHPFVSKSP----------------------------------------------------------------
----------------------------------------------------------------------------
----------------------------------------------------------------------------
----------------------------------------------------------------------------
----------------------------------------------------------------------------
----------------------------------------------------------------------------
--------------------------------TEDFPNTL----------------DLPVIN-
EKCRGRRYCYTYGTV-TTFHSNSP-----------VKGAVPIVKKNVCLS--------HN----------------
---DT--------LWSRPNHY--AGEPIFVAD---P-NGTE---EHDG--VILSSVLDG-------D--------
RGLNYL---LILDARTMKEIN--TAYM--P--TWI-PFGFHGQF--------------------------------
------------FPRSFPDAPVTGTHYEL-----------------------------------------------
----------------------------------------------------------------------------
----------------------------------------------------------------------------
----------------------------------------------------------------------------
----------------------------------------------------------------------------
----------------------------------------------------------------------------
----------------------------------------------------------------------------
----------------------------------------------------------------------------
-------------------------------------
>Lancelet_BCOLc
```


MTFSTRRACVLLVLTLSV--
VSATPKHL------MDLFKTN-VQEL--------Q---HV-P--MKY------------------DSKNTIPKWL-
EGTLVRNAPA-----MF------EL-----GG---RSAIHVFDGFAKIHSIAI---G-----QQA------I----
--NFTSAFLQT------SAYTRSKAANRY--A----PA----ITFYGVDPGFNP---V------ERLEAFEFP---
-------Y-----------DNTDVNVWKYGH-------GD-------------------NSVYAALT-DAW--
VYSKF----DLESLATQ--GVDIPEVSGI----------H-------------------WS--RLVQS--------
-------CAHPLIEPG------TNYSIN------------Y--LMFPSLI------PGQKQ---QYYVIRI-----
--------------------------------KDL---------NT----------FEVLANFQSD----KAS---
----YMHSFA-LTENYAVFFVQPA--FFDFMKM----V-------NTAMVGDAL----------------------
---GWFPDEKTQ-VVVVNLK-------TG-------KID---TVE--------------T-----D--
PIFYTHHANAY----ET-ADG---KIVADVCQFG-EGASV-----------------FAD--YKLPML--------
------------RNLTTLHQRPSKS----------------------RLYRYTIDI--------------------
--VNKSVQ--------------------------
---TKTFRS---

-----------------------------DDPLQDYLHKI-------------DVPIIN-
ENYRGRHYCYLYGVVFDFSPSNAL------------NGTFALVKKNLCTP--------GK----------------
---DL--------YWYHPNHF--VSEPSVVPN---P-EGKA---EDDV--VVLSAVFDA-------D--------
LGKSYL---LILDGKSMKPIN--TAYM--P--TYI-PFGFHGKY--------------------------------
------------FPKE--

>Lancelet_BCOLd_hybrid

MAFYTVGACVVLFATLSV--
TTAKSEGV------LTLFQTN-TEEV------------QHVP--MKY------------------DSKNAVPKWL-
EGTLVRNAPA-----LF------EL-----GG---RSAIHVFDGFAKLHSVEI---G-----PHL------V----
--NFSAAFLDS------SAYTRSKKAN----LYAP-AL----TFFGVDPPFSEL----------ERLDAFRYT---
------------------YDNTDVNVWKFGH-------GE-------------------DSVYAALT-DGW--
VYAKF----DSDTLASL--GVSVPSVTGV----------K-----------------------KSIAV--QS----
-------CAHPIVEPG------TTNNIN------------Y--IQTPNMI------PGQKQ---DYYVVRI-----
--------------------------------KDL---------DT----------YEVLATFQTD----RAS---
----YMHSFA-LTENYAIFFAQPA--YFDFIKI----VET---------AMVSI----------------------
---DWVPEDKTT-IILANIK-------TG-------EIQ---RLQ--------------T-----D--
AIFFTHHVNAY----ET-ANG---TIVADVVQFE-EGFAV-----------------FAD--YKLPQL-------
RN-------------ITSLHDKPAKS----------------------RLYRYTIDL-------------------
--ANKTVKTKTFR-------------------------
------SKSP--

------------------------------MEDFLHKI----------------EVPIIN-

ENYRTKHYCYVYGCI-IDFSPSNP-----------LNGSFAMVKKDLCTP--------GN----------------
---DL--------YWYHPNHY--VSEPSFVAD---P-DGKA---EDDG--VLLSAVFDA-------E--------
LGKSYL---LILDCKTMKAIN--TAYM--P--TYI-
PFGFHGRFFGKDWPVPRVLRGVRHVGKNRPETGKWRDDNFRFHVPRNNFRFHVPRNVRCSDENKQPPPPDPPRLLA
VFGAVPVETQHPCRTRPWWTGGATSVADLEQGVLPKFPQLQYLYIDFNNLTHVKKNWFDDAFKSLSRLTHLDLSRN
VLTCLSRETVEGLHRLDTLSLGGNRLFALADSAHLVMNWRLDYRHFKHAGHRVAVRVNKVLFCITEIPKLRQSYHV
QMQHDTHTLTAPPSVEPHSQCTVLNAGHRGTKYSLPLIIISVNTESDKHAKNIAHLCKHAWDNVSLVKVALRGDIT
LQIAPMGKDRSCNPQIVAIVLSDAVSNENNTKLYADRHSNNVSAFGHEEMTNVTCLVNTWEETYQHVFTTPLSSTP
DGTVCAEKTQATTTVSSTPGGTVCPREGMTLTTREPKIKITKFTLNQTNPSTNGTVKEKSPRVAIITMVVVVGVVL
VALFVMHVVRRCSRGDQAAQAAGHLAGVHGTAPSRTTQQSSSSGDDPQYSVIPDEYYNQQNTGTTNDYSQIPDEYY
NYYNTRPGAQHPYWEIPDEYYNYYNTRPGAQHPYWEIPDEYYNYYNTRNVWGTASLLGDMGLIDNNRYSTYPLTRR
VPQDNKDYSVRLNTTTAEVALSSSTRLGGKHPSYDTAPQVWRDPQNYQIPARGRDTNIRAQGMSVAQHPYWPRYGF
D
>Lancelet_BCOLe

MLVRNKVIFLSTVVLALACC--
IQASTVGL------EYFFTDN-FEEF------------RDRP--IQW-----------------SNSTPVPHWV-
HGTFVRNGPG-----RF------NI-----GG---RSVIHTFDGFAKLHSFKI---N-----NGT-----KI----
--LFSAKFLGT------STYTRSIAENDY--ATSITFD--------GVNPAFTF---------EERAESLVNG---
------------------IDNTNANIWKIGT-------GR------------------NAEFVGLT-DGA--
VFSKF----DIGTLNTI--RLVKP---------------D-------------------IT-HPIISLLSLMS---
-------TAHPLYEPK------TGHTIN------------L--VYTVNIV------PGLKH---TLTLYRM-----
--------------------------------KDT---------AT----------QEIITNVKLE----RMS---
----YMHSFS-ITDNYAVISLYPL--YVSVSKM----L-------NSAEAGKCL----------------------
---EWEGKDDTK-FLIISLK-------DG-------KVS---EMK--------------T-----P--
GFFAVHHVNAF----E--QDD---DIIVDMITYP--DNSM-----------------LYQ--FEIATM--------
------------LDAQKRSKLTNHA----------------------LLKRFTLNM--------------------
---KTSSVGVST--------------------------
---FSPKTP---

-----------------------------ELNFVNRM---------------ELPVIN-ENHRSGNYCYVYGVV-
FSFDSQTP----------TVHDNFAIVKKDLCNG--------GKG------------------DK--------
YWYLPNHY--PNEPYFIPE---P-NAKA---EDQG--VLIATVLDG-------P--------RKESYL---
LILDSQTLNVVN--YAYAK----TYI-PFAIHGRF---
---FPGCPELVNPVGFMM--

>Lancelet_BCOLf

MAFYTVRVGTLLLAMTLAAV--
VASAPDGV------LNLFRTN-VEEL--
--RHVRNAPA-----MF------EL-----GG---RSVIHVFDGFAKMHSIAI--GQ-----EGL-----------
--NFSSAFLQS------SVYTRSKKAGRYAPALTF----------LGVDPSFSE---L------EKIEALTYK---
------------------FDNTDVNVWKFGH-------GK------------------NSVYAGLT-DDW--
VYDKF----DLETLATT--GVSIPPIPGV----------------------------------TFLTH-FVQS---

```
-------CAHPLIEPG------TNNSLN------------Y--VLELGLL------PGQKQ---NYYVVRM-----
--------------------------------MNL---------TS----------FEILANFQTDKSV-------
----YMHSFG-LTKNYVIFFAHPA--IVNVEKM----VT------TVE-VAESM----------------------
---QWFPDEKTT-IVVANIK-------TG-------KVE---YL---------------Q---H-D--
ALFVTHHANAY----ET-QDG---KIIVDICASE-MGVSI-----------------LTQ--YQIPML--------
------------RNLSGLHQSVAQT----------------------RVYRYTIDL--------------------
--------------------------------------------ANKSVE--------------------------
---AKPFRS-------------------------------------------------------------------
----------------------------------------------------------------------------
----------------------------------------------------------------------------
----------------------------------------------------------------------------
----------------------------------------------------------------------------
----------------------------------------------------------------------------
-----------------------------KDPMQDFLHQI-------------EVPIIN-ENYRARHYCYLYGVV-
LDFSPSNP-----------VNGSLALVKKNLCTP--------GK-------------------DQ--------
VWYHPNHY--ASEPSFIPN---P-DGTD---EDDG--VILSSVFDA-------M--------LEKNYL---
LILDSKTMKKIN--TAYM--P--TYI-PFGFHGRF-----------------------------------------
---FPRQ---------------------------------------------------------------------
----------------------------------------------------------------------------
----------------------------------------------------------------------------
----------------------------------------------------------------------------
----------------------------------------------------------------------------
----------------------------------------------------------------------------
----------------------------------------------------------------------------
----------------------------------------------------------------------------
----------------------------
>Lancelet_BCOLg
----------------------------------------------------------------------------
----------------------------------------------------------------
MMLKTPVLLSLLCVVLWQGALS----------------------------------------------
VTPGKDGV------GFAFQPN-LHEF------------HKTP--MKF------------------ESNSPIPKWL-
SGTLVRNAPA-----RY------EV-----GD---RSVVDIFDGFAKLHSIDI---T-----PQS------L----
--NFSASFIKS------GIYNRSIEANTIAPMVTF----------LGVDPPFSL---Y------ERLEALAKT---
------------------LDNNDINVWKFGA-------GD-------------------SARYAALT-DGW--
VFPEF----NIDTLDTL--GV-------VQPDPLGDGQSG-------------------FG-VGPPTV--YLS---
-------CAHPVVEPS------TGHSIS------------Y--VIKPSYL------PGQSS---VLSVIRI-----
--------------------------------KDL---------GH----------IETIGSFEIQKNS-------
----YMHSFA-LTENYAIFFLQPL--YFDFIKL----M-------TTVEMQYAM----------------------
---EWVSGDKMA-IHAVNLK-------SG-------SVS---TLS--------------A-----D--
PRFYTHHVNAY-------ETGEG-HVVADVITFP-DPTPF-----------------LTA--LSLDKL--------
------------RDLTHLRKMDSYA----------------------RLTRYYLNL--------------------
--------------------------------------------TAETVK--------------------------
---VEPFVS-------------------------------------------------------------------
----------------------------------------------------------------------------
----------------------------------------------------------------------------
----------------------------------------------------------------------------
----------------------------------------------------------------------------
----------------------------------------------------------------------------
-----------------------------KTALGDFMNKL-------------DLPIIN-EKYRTKRYCIFYGTV-
TSFATSSP-----------FNGSIPIVKKNVCVP--------GS-------------------DA--------
HWSRPNHF--AGEANFVAD---P-SGTH---EDDG--VILSSVLDA-------D--------RGLNYL---
LILDARTFEEIN--TAYM--P--TWI-PFGFHGQF-----------------------------------------
---FPRSFQ-------------------------------------------------------------------
----------------------------------------------------------------------------
----------------------------------------------------------------------------
----------------------------------------------------------------------------
----------------------------------------------------------------------------
```


>Drosophila_melanogaster_NinaB

MAAGVFKSFMRDFFAVKYDEQRNDPQAE--
RLDGNGRLYPNCSSDVWLRSC-EREI------V-D---PI-E--GHH------------------SGH--IPKWI-
CGSLLRNGPG-----SW------KV-----GD---MTFGHLFDCSALLHRFAI---R-----NGR------V----
--TYQNRFVDT------ETLRKNRSAQRI--VVTE-FG----TA-AVPDPCHSI---F------DRFAAIFRP---
-------D--------SGTDNSMISIYPFG--------DQ----------------------YYTFT-ETP--
FMHRI----NPCTLATE--AR-------I------------------------------CT-TDFVGV--VNH---
-------TSHPHVLP-------SGTVYN------------L--GTTMTRS--------GPA----YTILSF-----
--------------------------------PHG-----EQMFED----------AHVVATLPCRWKL-HPG---
----YMHTFG-LTDHYFVIVEQPL--SVSLTEY----IKA-QLG-GQN-LSACL----------------------
---KWFEDRPTL-FHLIDRV-------SG--------KL-VQTYE--------------S-----E--
AFFYLHIINCF----E--RDG---HVVVDICSYR--NPEM-----------------INC--MYLEAI--------
----------ANMQTNPNYATLFRG----------------------RPLRFVLPL---------
GTIPPASIAKRGLVKSFSLAGLSAPQVSRTMKHSVSQYADITYMPTNGKQATAGEESPKRDAKRGRYEEENLVNLV
TMEGSQAEAFQGTNGIQLRP--

--EMLCDWGC----------------
ETPRIYYERYMGKNYRYFYAIS-SDVDAVNP---------------GTLIKVDVWNK--------S----------
----------CL--------TWCEENVY--PSEPIFVPS---P-DPKS---EDDG--VILASMVLG-------G--
------LNDRYV-GLIVLCAKTMTELG--RCDFHTN--GPV-PKCLHGWF--------------------------
------------------APNAI---

>Bombyx_mori_NinaB

MAAETQNLYPNCDSSVWLRSC-EEEV------T-E---PL-D--GNI------------------TGT--LPPWL-
RGTLLRNGPG-----SL------KV-----GA---MRFKHLFDSSALLHRFAI---H-----DGI------V----
--TYQCQFLKS------NTLKKNRAANRI--VVTE-FG----TK-SVHDPCHTI---F------DRVAAIFKP---
-------G-------DSMSDNAMISLYPFG--------DE----------------------IYAFT-EGP--
IIHRI----DPVTLDTL--DR-------K----------N-------------------MT--DCIAL--VNH---
-------TSHPHVMP-------NGDVYN------------V--GMSIVKG------------GLRHVVVKF-----
--------------------------------PYT---------EK----GDMFERAKVVASLKPRWPL-HPA---
----YMHTFG-ITENFFIIVEQPM--SVSLCGV----VRN-QLA-NKP-LASSL----------------------
---HWYPNHETN-IVLLSRK-------DG--------KE-WKRYR--------------T-----Q--
TLFFLHIINCF----E--KNG---SVIVDLCAYK--DPKA-----------------LDA--MYVHAI--------
----------ETMQSNADYAEWFRG----------------------RPKRLELPL--------------------
--DAPRLA--------------------------
-----KVVP---

-----------------------------TILADLGC----------------ETPRIHYDLYNGKPYRYFYAIS-
SDVDAENP---------------GTVIKADTVTG--------V--------------------TK--------
TWCEPNCY--PSEPVFVPT---P-DAKN---EDDG--VLLCALVWG----------------
GDREHEVALLVLDARTMLEVA--RAQFRTP--SPA-PKCLHGWF--------------------------------
------------LPQTTNI---

>Acromyrmex_echinatior_NinaB

MEYINYLVTSLNDSLFHTSNVLDNFEHAKCGKLKSEFYHDENENKV------------------------------
--------------PVDAKGNYYYANCDASVWMRS-CEKE------VIE---PLRS--SKV---------------
---TGA--IPKWL-KGTLLRNGPG-----NL------KV-----GK---YHYQHLFDSSALLHKFGI---A-----
DGE------V------TYQRRFVQT------EVYKRNMAAQRI--VYNE-FG----TN-ATPDPCQTI---F----
--HRVAAVFNP----------N-------KTLSDNSMISIYPFG--------DE----------------------
YYTFT-EAP--VMHRI----DPKNLETM--GR-------V----------N-------------------MS--
KYVNI--VNH----------TSHPHIM-A------DGTIYN------------V--GLSVTPM--------GPR--
--YSIVCF-----------YPNCVTVDNSG---KE--KE-----LSMF---------DQ----------
ATIVASVPCRWLL-NPS-------YMHTFG-ITENFFIIVEQPL--SISIVQM----MKN-HFK-QEP-MMNSF--
-----------------------KWHEDKGTL-IHVISRK-------TS--------QI-VKTFV-----------
---A-----E--TFFYLHIINQF----ETHDKE---YIVLDICCYR--NPKM-----------------LQY--
MYFDAM------------------KDMHGNANFSKLFRA----------------------RPLRFVLPM------
----
RKSSSNTPSEHNLITTETVHQSTEFRDVNGESVDHRSNYDVNGNEDGVEYDSEAKFWRDEHRSVLQKKPAAHRLLD
GKIFVKP---

---------------------------ELLCDVGC----------------ETPRINTDFHLGKEYRYFYAIS-
CDMDLNNP---------------GTLIKIDTFQK--------T--------------------KK--------
LWQQNGVY--PSEPIFVPD---P-NGKN---EDDG--VIVSSIVWA-------DK-------ETRVGL---
LILDAVTFTEIA--RATFDTP--GPV-PKCLHGWF---
---SLNK---

>Anopheles_gambiae_NinaB
--------------------------------MDVTV----
EDFCEETNFPEESPCPSPFEPAKQLLNIGFLQINGRFTPHSGPSSVCSTRSCSLAATPVPPDATDATDEQDEQR--
-HTEPEAPQ-MFELTGRATPVFTWNPMPTSSNVIYALSDKNQQIVFSQAELEVDEEPATEPTKAAKD-------

TTQPKDD----------MYPNCDVN------VWLRSCE--QEI------V-E---PI-E--GKV------------
------QGT--VPEWL-NGSLLRNGPG-----SL------KV-----GD---MMFNHLFDSSALLHRFNI---D--
---NGK------I------TYQCRFLKS------DAYKKNNAARRI--VVTE-FG----TS-AVPDPCQTI---F-
-----QKIAAVFNK----------P------GVNNSDNAMISIYPFG--------DE-------------------
---FFAFT-ESP--IIHRI----DPETLDTE--AK-------L----------N-------------------VS-
-DYVGI--VNH----------TSHPHVMS-------DGTVYN------------L--GCSITKT--------GPA-
---YTIICF-------------------------------------PHG---------PG------
MFENARIVASVPARWKF-HPG-------YMHTFG-ITENYFVIVEQPL--SVSVPTM----VVS-QIR-NRP-
MAAAL-------------------------KWFESQQTY-IYLLDRD-------TG--------EL-RHTFH----
----------T-----E--PFFYLHIINQY----E--REG---HVVLDICCYK--DPAM-----------------
LNC--MYVDTM------------------RNMQNNPDYAKMFRG----------------------RPLRFVLPL-
-----NCGTERAKGRPAAGPKGSSAAADASWSDMWQNLGSFSAGDSTDSGLVLQNLVQIEDSKATAYVM-------
-------------------PNRTIFCKP--

--ELLCDLGC----------------
ETPRIYYERHLGRPYRYFYAIS-SDVDASNP---------------GTLIKVDVVTK--------G----------
----------KL--------TWCEENVY--PSEPIFVPA---P-DPQS---EDDG--VVLAAMVWG-----REE--
------QNRAGL---LVLDAKSFTELG--RCEFVTP--GPV-PKCLHGWF--------------------------
------------------QPTATTTKGD--

>Megachile_rotundata_NinaB

MKSSCSRSKSEYDLSSVKLNELDDLLHGKRLKLSRTSFRLNDAEDILSTNENESEEKPD-----------------
-----------------------------YFPNCDAS------VWMRSCE--KEV------I-E---PI-P--
GKV------------------TGK--IPCWL-KGTLLRNGPG-----SL------KV-----GE---
YSFNHLFDSSALLHRFAI---A-----DGK------V------TYQCRFVQT------DVYKKNKAAQRI--
VVTE-FG----TR-AVPDPCKSI---F------QRIAAVFTP----------G--------EDSDNSMISVYPFG-
-------DE----------------------YYTFA-ETA--VIHRI----DPETLETK--GK-------V-----
-----N-------------------VS--DYVRI--VNH----------TSHPHVM-N------DGTVYN------
------L--GLSVTPR--------GPM----YNIVCF-----------SPSRIIVDDSG---DE--QE-----
LSMF---------DQ----------AVVVASVPSRWLL-NPS-------YMHTFG-ITENYFIIVEQPL--
AVSLTTV----LSC-KVK-QQP-MHAAL-------------------------KWHENENTF-IHVVSKE------
-TG--------SL-ERTFI--------------S-----E--AFFFLHIINQF----ETRDRD---
YIVLDICCYR--DAKM-----------------LDC--LFVDAM------------------
KNLHKNPDYAKMFRG----------------------RPLRFVLPM-------------------------
KRPQSDVPLEYNLITAKTVNQGLESFQDVTDVKNRSEIIDGSKVENSVDVSKEKRHDGKNILRRRAAAHRLPDGNI
VVKP--

------------------------ELLCDLGC----------------ETPRLNYDSYLGREYRYFYAIS-
SDVDLENP---------------GTIIKVDILEK--------T--------------------RK--------
TWCEKNVY--PSEPIFVPD---P-NGKT---EDDG--VVLSAIVWS-------DI-------ETRVGL---

LILDSVTLTEIA--RATFDTP--GPV-PKCLHGWF---
---TLDK---

>Culex_quinquefasciatus_NinaB
--------------------------------MDTISVEVEEEFCEETNF--ESPCPSPLE-
GDKVLNMDFLTINGRFTPMSGQASACSTRSVSLATTPIPPEMLGEGEQEEQGEIVINVQPKPSRHLFESSGRTTPV
FTWNPLPTGNSIVYAKG-KDNEVKFKKFEDDSTDTESSAVSDAEGDESAITAETTHKKHDPSN-------
CYPNCDVN------VWLRSCE--QEI------V-E---PI-A--GEV------------------RGV--VPEWL-
NGSLLRNGPG-----SL------KV-----GD---MTFNHLFDSSALLHRFNI---E-----NGH------V----
--TYQCRFLKS------DAYKKNKAAQRI--VVTE-FG----TS-AAPDPCHTI---F------HRIAAIFGK---
-------P------GENVSDNAMISIYPFG--------DE----------------------YFTFT-ESP--
IIHRI----DPETLETE--AK-------L----------N-------------------LS--DYVGI--VNH---
-------TSHPHVMS-------DGTVYN------------L--GMSVTKT--------GPT----YTIICF-----
--------------------------------PNG---------EH------MFENARIVASVPARWKF-HPG---
----YMHTFG-ITENFFVVVEQPL--SVSVPHM----LVS-QIK-NEP-MVSSM----------------------
---KWFEKQQTY-IYLLDRD-------TG--------EL-KHTFH--------------A-----E--
PFFYLHIINQY----E--KEG---HVVLDICCYK--DPAM-----------------LNC--MYVDTM--------
----------KNMQQNPDYARMFRG----------------------RPLRFVLPL--------------
NFEESLKRSSSVFSASKSWTGLMKRLSVGDDLDADDSLTLKNLVTLEGMSATAYVM--------------------
------PDSTIFCKP---

-----------------------------------ELLCNLGC----------------
ETPRIYYEEHLGREYRYFYAIS-SDVDASNP---------------GTLIKVDVINK--------T----------
----------RL--------TWCEENCY--PSEPIFVPA---P-DPQS---EDDG--VVLAAMVWG---------
RDE----ENRVGL---LVLDGKTFTEFA--RSEFETP--GPV-PKCLHGWF-------------------------
-------------------QSSTSKKSQ--

>Nasonia_vitripennis_NinaB

MNIYKRSYSSYDLNVSKLNIIENLLNENTPKLKSSKTTIFRKEDDSKSSTGVFNS---------------------
-----------------------KHNAIDDY------VNFYPNV-DTSVWMRSCEH-EVIEPI-T--GKI------
------------SGS--VPDWL-KGSLLRNGPG-----SL------KV-----GE---
YRFDHLFDSSALLHRFEI---N-----NGS------V------TYQRRFIQT------DVYKKNHAAKRI--
VITE-FG----TK-AVPDPCQSI---F------ERVSAAFNP----------A-------ENMSDNSMISVYPFG-
-------DE----------------------YYTFT-ESP--VIHRI----DPKSLETL--RK-------V-----
-----N-------------------VA--NFVNI--VSH----------TSHPHVM-S------DGTVYN------
------L--GMSITKR--------GPA----YNIIKF-----------TPNIFTDDNRA---KK--RK-----
LTMF---------EQ----------AEIVSTIPSRWIF-NPS-------YMHTFG-ITQNYFIIVEQPL--

```
AIALMSM----VAC-QIK-NEP-MCTCL-------------------------KWHENENTR-IHLISRD------
-SG--------KV-EKTFI--------------A-----E--AFFYLHMINQY----ETRDGN---
YVVLDLCCYR--DAKM-----------------LDC--MYVDAM------------------
QSMHKNPDYSKLFRG----------------------RPLRFILPL-
NEPAQNTSPDVNLVKINNAYQRPDTFNDRLDDKKLMNKRRELDENNNSTHDSNKYENLHQRKAAAFRLP-------
--------------------NGNIFVKP------------------------------------------------
----------------------------------------------------------------------------
----------------------------------------------------------------------------
----------------------------------------------------------------------------
----------------------------------------------------------------------------
----------------------------------------------------------------------------
------------------------------------------------ELLCDLGC----------------
ETPRINNDSNLGNEYRYFYAIS-SDVDIENP---------------GSVIKVDTKHK--------T----------
----------KK--------IWCEKGVY--PSEPIFVPD---P-SGKN---EDDG--VVLSALVWG-------Q--
------ERETEV-GLLVLNAATFEEIA--RVTFDTP--GPV-PKCLHGWF--------------------------
------------------SNVIS-----------------------------------------------------
----------------------------------------------------------------------------
----------------------------------------------------------------------------
----------------------------------------------------------------------------
----------------------------------------------------------------------------
----------------------------------------------------------------------------
----------------------------------------------------------------------------
----------------------------------------------------------------------------
-------------------------------------------
>Caenorhabditis_elegans_BCOa
----------------------------------------------------------------------------
----------------------------------------------------------------------------
----------------------------------------------------------MEAEGF------
PRLFHNF-ENVP--------E---PKEC--KKV-------------------GS--VPSYL-TGTMLRNGPG----
-MF------TV-----GE---EEYKHWFDGLGFMQRYHF---E-----DGK------M------FYSARYLES---
---EAYTKTVEAQRI--VAGT-FG----TL-SFPDPCKTI---F------SKYFSEFMN-----------------
HSEKHDNSNVAFTPVG--------DS----------------------LYACT-ETP--HMYRV----
DLDTLKTL--EA-------A----------D-------------------FS--KFVAV--HSC----------
TAHQLYD-E------NGDVYN------------I--GSRFGPE--------SAH---VFTVTKN------------
-------PKN---QKSE-N-----DHSW---------EH----------TSKIGEIKASDPL-YPT-------
YMHSFG-MSENYLVMFESPV--RLHLQKY----LLS-EFV-RAT-YHDCL-------------------------
EWHGDKDVS-IFILNKK-------TG-------EQL-PLTLK--------------M-----N--
PFFTFHHANTF----E--KDG---CLVMDYCRIE--NAGK-----------------FDT--LLISNM--------
------------KTGEFQYDAKFLP----------------------YLTRVIVPM--------------------
----SVSSSAKPGDNLL-------------------KSVPWASGCTSILQ--------------------------
DDGSIRLTE-------------------------------------------------------------------
----------------------------------------------------------------------------
----------------------------------------------------------------------------
----------------------------------------------------------------------------
----------------------------------------------------------------------------
----------------------------------------------------------------------------
-----------------------------RRVCETSM----------------
EFPRYHWEKINMKEYRYVFGSTVFGRIDGNL---------------AGVVKADLKFG--------N----------
----------HL--------IWNRENPHQICGEPIFVPN---P-EGIE---EDDG--ILIVPIMSS------SE--
------KQVPFV---LILDAKTLEE----TARFEIPE-ARI-PLGFHAFY--------------------------
------------------KPKN------------------------------------------------------
----------------------------------------------------------------------------
----------------------------------------------------------------------------
----------------------------------------------------------------------------
----------------------------------------------------------------------------
----------------------------------------------------------------------------
----------------------------------------------------------------------------
```


>Caenorhabditis_elegans_BCOb

MSAASSNCQNFNLEKILQISEPAR--
HSMEKEGF------ARLFHNF-DNVI------------EPKL--CST------------------SGS--VPSYL-
KGTMLRNGPG-----MF------EI-----GD---TKYQHWFDGMGFIQRYHF---E-----DGK------M----
--YYSARYLES------ENYKKNMEAQRI--VTGS-FG----TA-SFPDPCKSI---F------SRFFSSFVQ---
--------------SEGIHDNANVAFAPVG--------DG----------------------LYACT-ETP--
NMHRV----DLDSLDTL--EP-------V----------D-------------------FS--KYVAL--HTC---
-------TAHQLFD-E------NGDVYN------------I--GSRFGPD--------AAH---VFTVTKN-----
--------------PKN---LQSD-S-----DRSW---------EH----------TTKIGEIRCSETF-YPT---
----YMHSFG-MSENYLIMFESPI--RIDIKKF----IMK-RFI-TTT-FRDCM----------------------
---KWHADKDVK-IFILNKK-------TG-------EQV-PLKLK--------------M-----A--
PFFTFHHANTF----E--RDG---CLVVDYCRIE--QAGN-----------------FDA--LLIENM--------
------------KTGNFQNDALFLP----------------------YLTRVIIPL--------------------
----SIPDGAQPGDDLL-------------------KPLGWAKGCSAIFQ--------------------------
DDGKIRLKE---

-----------------------------KRVCDISM----------------
EFPRYHWEKINMKPYNYVYGSSVLGAQKSET--------------LPGIVKADLENG--------D----------
----------HK--------VWRRENDKQICGEPIFVPN---P-EGVR---EDDG--ILIVPVMTI------SD--
------GQRPFV---ILILEAKNLTEIA--RYTI--PE-ARI-PLGFHAFY-------------------------
------------------QGRT--

>Caenorhabditis_elegans_BCOL

MSPIIALLLVGVPTILAQSFNQTHLGFP--
EAWDGDKY------RELYCPS-----------------KN------------------------------IPKWL-
DGYFLCQLSA-----SY------GNSSAPEGE----KLNHMIDAIGAVGSFHV---S-----NGQ------V----
--VFSAQYYPARPYKIWEFYDRNMSKASV--PWAG-WS----DY-----NLTAM----------SRWEQVPAN---
--------------PDSARFHPNLDFWKVG--------NR----------------------IVAGT-
EAPYWVGYEF----DVRTLQKF--KL--------------------------------------FPFKEENDI--
FSTPRHTMIPISMAIHERND-A------DGTIWGSFSAMNFEEQRFF--QGIFTVDTNGVRRVVGLY---
DYGVWDT------------------------------------NACG---------SN----------
DEYIGDKTL-----LPG-------YIHSIT-STENFIILPITSL--LINPCKFKEPPLNNVRSAIQKG-GLWGM--
-----------------------DFYDMVPMR-FLIFNKK-------TL-----EFTTSKPLEVF-----------
---------P--SMFVTHQLNAF----EA-DDG---NFVADMVVYDSHDPYV------------------KY--
FYTDFL------------------------TKQLYPSTA----------------------RVLRFTLDS------
--KKQRVM------------
-----------------YNYLVP---

---QETIAA------------------DFPQFN-
HNYEQKPYQWGYLVQ---HPFASG---------------NSIIKINVDEP--------AGNRN-------------
---LE--------FRAEPTLV--LHEPWFVQK---P-DSKK---EDEG--VLIVRGLDT-------A--------
ENKGVL---IVIDAEKMVELG--RAYV--P--ISI-PFGFHNRF--------------------------------
----
FSKKDLGLPEGFQVGQSQYRPIEKKQGFATLPLRKVSTVPPTTSTSSTTTQKPSTTTTVPSTTPTSTTTTTTTTPK
PTTSTSTSTSTTT-----------TTTTSTTATTTPQPTTTTTSEKPVTLTTQTWTAPPTTTVKRTT---------
------
PQTVPTTTPKIPRWWPLAGSGSTEQPWWQKVQTGQNTLPPLFPVSKRVEEKVEKVSAKPNESDNKIPEQKPVTATG
SMDEIYEQTLGALCSWLPKVFTSISNELCWKQGKTAAK--------------------------------------
----

>Saccoglossus_kowalevskii_BCOLa

MNGQRLQILIIVFSVIVVST--
TCWKLPDN----IRDTYFGTI-SKEH------------SYTS--TKV------------------HGN--IPDWI-
EGYFLHQSCG-----TYGNQSNPEI-----GA----KAVHSFDGLGAVASFKF---K-----EGA------V----
--DFGMRFYETQSFKIWDYYDWDMDKSKI--AWSTSFS---------PVNQKQF----------RKWENFQDY---
-----------------TGDNPFVTFWKIG--------DN----------------------VEAMS-EGP--
AGVWF----DLESLETK--GEYPFKDN------------N-------------------FGFPDYMTP--INN---
-------PAHEHYGED------GVTVYG------------V--TAMMNVS--------DVT---NINIMRM-----
---------IYKIQDEERIPMVLFQE-----AGPL---------DS--------RLCNDEGTYPKLKD--RSG---
----YLHSFA-MTQNYFVLPISTF--FIDLCKI----FKSKPSDPVLPGFASYY----------------------
---NYTKDIPTR-FVIVSKQ-------SN-------QVV--GEFT--------------T---S-D--
GIFVTHQLNSY----E--KDG---LMYLDMLTYH---ANI-----------------YAL--LTIKNI-------
VE----------------KPTTPIDT----------------------HVERFVIDM-------------------
---SDWSLV-------------------------
----KRIDLY--

------------------------------PSAEFKTV----------------
EFSTINYEHYSSKPYKYAYMTG------LNP-------------AVTTLIKLNVETN--------K----------
----------AV--------YWGPHDGL-LPSEPIFIPS---P-NAVD---EDDG--VIVTNVLDT-------N--
------TSKGIV---VVLDAKSFTELG--RAVS--S--ELM-PFGLHSRF--------------------------
----
IARKFERRSRHSLLRFLSDSSVHKVTHIYTIGYHGYNISTMIALYHEEELLASRSCERGSISSCSARNVGKNQPDM
HDGEQF--

>Saccoglossus_kowalevskii_BCOb

--
MTRQVMLFILIGAMASPVLVKSACLDGWSEYLGHCYGCMQPATTWEHADYICKNHFGYLASIHTLDENEFVAGACS

SDWDLWIGFN--KKDDSLTWQWSDGTDLVYTNW-
EEGQ------------PDGE--SSGLLDGDVSCAMQKNEAVVGSGT--IPKWL-TGTLLRNGPG-----KF-----
-EV-----GD---EPYKHWFDGLALIHRFSF---H-----DGQ------V------TYQNRFLRS------
DAYNKAMKYNRI--ILSE-FG----TL-GIPDPCKTI---F------ERFATHLVP------------------
LNITDNDLINVFTIG--------DE----------------------IYVNT-ETC--FFRRIS---NTETLEFD-
-KK------------------R-------------------INQFRTLGV--LTA----------SAHPHVD-R--
----DGTTYN------------M--ASSYMT---------GSC----YNIVKY-----------------------
GKESSDD--------PS---------KN----------AEIIGTIRAKHCL-KPS-------YYHSFG-
MTENYFIFLEQPL--YINLKKL----ATA-QLR-GSA-ICTCL-------------------------
EFDNNAKSY-FHLMEKK-------TG-------KKL-SIEYE--------------A-----D--
GFFGMHVINAY----E--DEG---HVVFDMCCYH--DDEL-----------------ITK--FYLDYL--------
-----------RNGDHEGKRHFSPS----------------------SSMRFVMPL--------------------
---HVNLSTTPRGRNLV--------------------TLKNCKAVAVLQK--------------------------
-NGTVHITP---

-----------------------------EKISEAIT----------------DMPRINYDKCNQRPYTYYYGVA-
ACKPGDFT---------------NGLVKVNVRQK--------S--------------------FE--------
LWSDDDCY--PSEPVFIES---P-GAVM---EDDG--VIVSTVINT-------R--------KRSAFL---
LVLDASTFTELG--RANI--PDEVEC-PVGFHAMY---
---LSKQQTQLANQSHKQNGLPSV--

>Saccoglossus_kowalevskii_BCOa

---------------------T----------------------------IPEWL-NGSFLRNGPG-----LF---
---EI-----GE---DSFNHLFDGMALLHKFTV---K-----DGK------V------TYQNRFLTS------
DTYRKNKAANRI--VVSE-FG----TL-SNPDPAANF---V------EKMLSYVWP------------------
PVPEDNCNISWFKIS--------EK----------------------FCVCT-ETP--FVWQV----DPHTLVAT-
-KK-------H----------D-------------------LA--KKISI--HTM----------SSHPHTL-K--
----DGTLLN------------V--GNHYGPA----------P---TYNVIKI-------------------SPS-
------EGG----NDPF---------EN----------TQIICSIPARYRL-HPG-------YYHSLS-
ITENYVVFLEQPL--MTNVAKL----MTG-RFT-STP-LSGAL-------------------------
EYDDNISSI-FYLIRHD-------TG-------ELV-ASKYV--------------A-----E--
PFFSFHHVNAF-------EDGD--HVVVDLCCHR--DMDI-----------------VNK--MSRNSI--------
---------------EANNTDPANI----------------------ELRRYVFPL--------------------
----NVNTKSSTGTNLI--------------------TLDYTSCCATKRD--------------------------
DGNDVYVTY---

-----------------------------EKMIEKGM----------------ELPITNYERYNGRKYRYVYGVS-
--------------------DSATELMKVDLQNK--------T--------------------YK--------
LVNHDAYF--PAEPVFVEA---P-NATS---EDDG--VVMACMVSA------KE--------DTWSYL---

LVLDGKTFTEIA--RAEI--P--VAL-TSGLHGMF---
---FKGIV--

>Daphnia_pulex_BCOa

--MAGKVDHP------
STWVRTL-DKET------E-S---PV-D--GII------------------IGK--IPEWL-QGSLMRNGSG----
-IL------EI-----GE---TKMDHLFDGLSVINRFAIDGQG-----EGK------A------TYQNCILKS---
---DTYKDSKAANRL--TMHQ-FG----TF-AYPDPCKSL---L------GKYACFFKA-----------------
AIDCLYNCAVNLCYFG--------DK----------------------LYALT-ETP--FIRQI----
DSETLDII--GEK------C----------D-------------------IS--KLVAV--NHA----------
TAHPHVN-E------DGTVYN------------M--GNSVGSK--------GPA----YNVIEF------------
-------PAG--------------DGEF---------CR----------QKIVATIPSRWKM-SHS-------
YYHSFG-ITENYFVFIEYPL--IANTAKL----LTM-NLR-QKA-FDSSL-------------------------
DWTPNEHTR-IILCERK-------TG-------DLV-ETVYE--------------T-----P--
PFFSFHIGNCY----E--KDG---YLVMDLSHCK--DDTV-----------------ISG--LSVKSL--------
---------------RENKGDFTNT----------------------YYTRFVLPL--------------------
-----------------------AGIEPPVDKTFQKRENLIKLEGSKCIS-----------------------
YWISDKIIFCEP--

--------------------------------DRLSDLSL----------------ELPRIN-
YSRNGLEYRFAYGIS-QTDGYAES---------------EKILKLDVTSR--------D-----------------
---VK--------IWAKTEFS--PSEPVYVSR---P-GSTD---EDDGV-ILFSAVHQV-------D--------
VKKVLL---VILNAATFEEEA--VVEYVAS--GVV-TKDFHGLF--------------------------------
------------SRTGDALHGY--

>Daphnia_pulex_BCOb

--MAYPNCDL------
NIWLRSC-EIEV------I-N---PL----EGI-----------------TTGA--IPLWV-NGSLYRNGPG----
-KQ------DY-----GR---QRVNHLFDAAGLLHKFNV---R-----NGK------V------TYQSRYVNS---
---TSYVLNTAAGQL--VVPE-FT----TP-AAPDPCKSI---F------HRISSLFVL----------D------
-QVVSDNAMISIYPLG--------KD----------------------LYAFA-ETP--FIHRI----
DPVTMETT--RR-------E----------S-------------------LH--ETLSV--FNQ----------
SSHPHIT-E------SGEAYQ------------L--GQKIGAK--------GPS----YVVIHY------------
--------PA---DD--QE-----SKAT---------ER----------ARVVTTVPCRSLK-EPS-------
YMHSFS-ITDSYFVLIEQPL--NVSFKTV----FSS-FLS-GKP-LVNSL-------------------------

KWRPNKKTR-IRVISRK-------TG-------AEL-PVQYV--------------T-----E--
AFFFLHTINAF----E--ADD---HLIVDICCYA--NAKM-----------------LDC--MYIDAL--------
----------ENAQSDPNYASLFRG----------------------RPKRFVMPL--------------------
---------------------------------------NPKQGKEGNLN-------------
TYAHSLSEAKWMNGGSSMYIKP--

--DELCPLGC----------------
ETPQINYNKYNGKPYRYFYAIS-SDVDAENP---------------GTLIKVDTIEK--------K----------
----------CR--------TWAEKNVY--ASEPVFVPH---P-DAKS---EDDG--VLLSSMIFG-------
GHN------EKRTGL---LVLDAATFKEIG--RTEFHTP--SPV-PKCLHGYF-----------------------
---------------------SPTK---

>Caenorhabditis_remanei_BCOL

MSPITVLLLVGVPLVLSQSFNQTHLGFP--
EAWDGDKY------RELYCPS-KN--IPKWL-
DGYFLCQLSA-----SY------GNSSAPEGE----KLNHMIDAIGAVGSFHV---S-----NGQ------V----
--VFSAQYYPARPYKIWEFYDRNMTKASV--PWAG-WS----DY-----NLTAM----------SRWEQVPAN---
--------------PDSARFHPNLDFWKVG--------NR----------------------IIAGT-
EAPYWVGYEF----DVRTLQKF--KLFPFKEEND----------I-------------------FSTPRHTMI-
PISM----------AIHERND-A------DGTIWGSFSAMNFEEQRFF--QGIFTVDTNGVRRVVGLY---
DYGVWDT------------------------------------NACG---------SN----------
DEYIGDKTL-----LPG-------YIHSIT-STENFIILPITSL--LINPCKFKEPPLNNVRSAIQKG-GLWGM--
-----------------------DFYDMVPMR-FLIFNKK-------TL-----EFTTSKPLEVF-----------
---------P--SMFVTHQLNAF----EA-DDG---NLVADMVVYDSHDPYV------------------KY--
FYTDFL------------------------TKQLYPSTA----------------------RVLRFTLDN------
--KKQRVM------------
-----------------YNYLVP---

---QETIAA------------------DFPQFN-
HNYEQKAYQWGYLVQ---HPFASG---------------NSILKINVDEP--------AGNRN-------------
---LE--------FRAEPTLV--LHEPWFVQK---P-DTKK---EDEG--VLLVRGLDT-------A--------
ENKGVL---IVIDAEKMTELG--RAYV--P--ISI-PFGFHNRF--------------------------------
----
FSKKDLGLPEGFQVGQSQYRPIEKKQGFATLPLRKVSTIPPTSPTASPTTTTSTTTSTTTTTTPTPTTTTTT--
PKPTTTTSTTTTTTTPRPTTTSTSSPTTTTSMTTTTTPQPTTTTSSEKPVTLTTQTWTAPPTTTVKRTTREPSHPQ
NISYQSFSAQTVPTTTPKIPRWWPLAGSGSTEQPWWQKVQTGANTLPPLFPVSKRVEEKVEKVSAKPNESDNKIPE
QKPVVSTGSMDEIYEQTLGALCSWLPKVFTSISNELCWKQGKTAAKWMAPLAQTYAERFRMGRANRKVSPNDTPNL
QPSLKQMAFDGQQEQQAEPIRFG---

>Lottia_gigantea_BCOL

MIKLKSLKILLVVLFTLLKF--
CCCEDYGF------QLLFTSN-TEEL--------E---DT-E--IKF------------------ERK--LPKWL-
KGTLVRNGFG-----KF------EM-----GM---RQFAHSFDAFAKLSSWKF---DG----NGS------A----
--YFSTRFIES------SFYNDSLATDDI----AP-YL----LF-EGVIPEFSA---Y------DKMKALMRG---
-------I-----------DNMNVNVYKFY--------NE----------------ERNSSEYVALN-DFW--
KIYEI----SPWDLTTL--GAVTPH----------------------------------IG-ERSLGG--SFG---
-FLDLLSSAHPLPEPG------TRNHIT------------F--LSSVSVI------PFFSN---TISLIRI-----
--------------------------------KSA---------VE----------REVIAKWDVG----RVP---
----YMHSFS-VTENYAILFANPF--YVNVLRM----L-------RKAEPFECL----------------------
---DWYADEPTT-VYVVNLN-------SG-------ELI-TMSTI-----------------------
NVFTMHHVNAY----E--RKD---KIIIDVSAYP--SPEF-----------------VKS--LQMEIL-------
RD-------PTQRN------AFDAHA----------------------QLKRFRINL-------------------
---TKNKIN-------------------------
------YVKR--

------------------------------DPKTKIPY--------SMNL----DMPTIN-
ELYRASRYCFVYGIV-LKTDNINL-------------SHISIVKRDMCRR--------GR----------------
---DR--------SLYIKYHY--PSEAWFVPN---P-RRKS---EDDG--VLLVPILDG-------V--------
RQKSYL---AVISAKTMKIIN--RSYL--P--VVI-PFSLHGRF--------------------------------
------------FDEIV---

>Biomphalaria_glabrata_BCOL

MMINFLVQMLLTVCLVGQHCVLSEDKTSVPPFNHRKQEIKSVIQNNRTK---------------------------
-----------SQQTDEGF------NLLFQSN-TEEL------L-----DV-P--VAF------------------
ENP--LPEWL-TGNLVRNGLG-----MF------EI-----GP---RTFLHAFDGFAKLASWKF---------
SGN----STV------LFSTKFIKS------DFYRASKKAKTI----AP-YL----LF-QSVEPAFSW---L----
--ERLQCLMRG----------L-----------DNMNVNILSFRNAKN----GQ--------------------
PEYAALS-DFW--LVYKI----GLENLTTE--YK-------VSPKFISRNGNK-------------------WL-
SGKAFL--DLL----S-----SAHPLPEPG------TQNYLT------------F--LSSVSML------PLTKS-
--IISLVRI-------------------------------------NSL---------KR----------
RSVVAKWEVD----RVP-------YMHSFS-VTKTKAILLASPF--YVNVFCM----A-------RKAEPFSCL--
-----------------------DWYPEENGT-LYVINLK-------SG-------KVR-TISIQ-----------
------------NVFSMHHVNAY----DINNR----KIIMDLSTYT--SPNF-----------------VRF--
LQLKVL-------RD-------------PVERNSFDAHA----------------------
RLRRFYIDLKRLQVHEIDLPPFPVQSLASKLDMPVINESFRSKKYCFVYGLVLKTDNVSLTNIAIVKKDLCNNGTG
DIA--------------------
WQQPGHYPVEPWFVPRPGGTSEDDGLLLVPVLDGQKRQSYLAILDAISLRLINRAYTPTHIPYSLHGRFFANELEV
VRFLFISLLFLEGHDNVLFDDKNAILPSRQRNIGMMSQRRIKLNTADSDSDDKLNQTSDNGFNLLFQSNSEELLDV
PVTFEKSLPSWLKGSLVRNGLGMFEIGPRTFLHAFDGFAKLASWKFSGNSTVLFSTKFIKSDFYRASKKAKTIAPY

LSNQSVSPGFNLIEKLLCLIRGVDNMNVNIYEFRNSNNGKTEYAAMTDVWSVYKIGLENLTTEYKASPKLTSNKNN
AWVLGKAFLDLLSSAHPLPEPGTQNYLTFLTSVAVLPWVKSKISLVRINSLKKRSVVSEWGVDRVPYMHSFAVTET
KAILLANPFYVNTMCIMRKAEPFSCLDWHPKENGTLYVINLTSGKVMTITIHSFFCMHHVNAYDINDQVIIMDIST
YPSPDIINAFQLSVMRDPFARNSFDAQAHLRRFHIDLKNLQVHELQLLPFPVFSLASRLDFPIIN-
ENFRSKKYCYVYGLVLKSDNVSFG--------------NMAIVKKDMCYS--------GTG---------------
---DR--------TWQQPGHY--PVEPWFVPR---P-GGTS---EDDG--LLLVPVLDG-------Q--------
KRQSYL---AILDAVSLRLIN--RAYT--P--THI-PYSLHGRF--------------------------------
------------FAN---

>Aplysia_californica_BCOL

MVRRAPMLWRVPLVLTLCLQASRVVTDAACDVTHGCTDSGQSA---------------------------------
---------------DQAAERGF------SLFFQSN-LKEL------------EDVP--VTF--------------
----VKP--LPPWM-NGTLVRNGLG-----QY------EN-----GP---RRVVHAFDGFAKLASWKF--------
-QGN----STA------LFSTKFIRS------RVYRMSQKTNTI----AP-FL----LF-QSVTPPFSF---F---
---EKVRCLLRG---------------------IDNMNINIFRFPHPKKKR--HE---------------------
-YAALS-DFW--ISYKI----DIDDLSTK--RRVVPVIKKTPKSATA------------------------
LQGSGFLNL---LS----------SAHPVPEPG------TNNYLT------------F--LSSVAFY------
PWDSN---VINLIRV-------------------------------------KSL---------KR----------
RKIIAQWPVERLP-----------YMHSFS-VTQTKAILLASPF--HVNIYCM----A-------QKAEPFSCL--
-----------------------DWDGTQPAT-LYVVGLG-------SG-------HIQ-TVTMP-----------
------------CVFTMHHVNAY----DVNET----TIIMDISTYP--NPDF-----------------VHH--
LELEIL-------RD-------------PVKRNSFAAHA----------------------LLKRLSIDL------
--VKGQVT------------
-----------------ELPIDI---

---DPSNPSLASLL-------------DMPVIN-
EKYRSKHYCFVYGLV-IKTDNITL-------------SATAVVKKDLCGR--------GKA---------------
---DL--------TWQMAGHF--PVEPWFVPT---P-GATA---EDDG--LLLVPVLDG-------V--------
REITYL---SVLDARTMTSVN--RADL--P--TYI-PYSLHGRY--------------------------------
------------FEQEDK--

>Crassostrea_gigas_BCOLa

MAIYGNSLKKASCRSDITFNLRTFLKLLFTEITAMKKTFLCSILLFQLFIQYLGVVATSKNFGNSSGKSSEF----
---KESVDPGF------DLFFASN-DREV--------
E---NV-P--IYF------------------KNP--LPPWI-KGTLIRNGLG-----RY------EI-----GR--

```
-RRFTHAFDAFAKLLSWKF---PG----NGT------A------FFSTKFIQS------DFYKKSIQVNDI----
AP-YL----LF-ETVEPEFNV---F------QRFEALLRG----------I-----------DNMNVNVYKFGE--
-----ND----------------------VAVLN-DFW--KVYTI----DPTSLKTR--TSITPS-----------
-----------------------IH-VPKSPS--AFT----FLNFISSAHPLPEMG------TPHHLS--------
----Y--LSSVNII------PGMKS---RISLVRI-------------------------------------KSS-
--------EE----------REIITSWEVD----RVP-------YMHSFA-ATKNYVVFFACPY--FINVKKM---
-V-------RYAIPERSL-------------------------DWEQESPTI-LYVVHIK-------SG-------
KVV---TMM--------------T-----D--NVFMMHQINAF----E--RDG---VIIVDISAYP--NPDF----
-------------VKN--LEMKNL-------LD-------PVKRN------QFNTRA-------------------
---SVRRYTITL----------------------------------------------------------------
NTQTVV-------------------------------WKTF-----------------------------------
----------------------------------------------------------------------------
----------------------------------------------------------------------------
----------------------------------------------------------------------------
----------------------------------------------------------------------------
----------------------------------------------------------------------------
-------------------------------------------------------------RDTETAPC-------
-ASAL----DLPTIN-EIYRYRPYCFAYGVA-LKCNNVSL-------------SDIAIVKKDLCGR--------
GR-------------------DK--------MWKIANHY--AVEAWFIPE---PGGGKY---EDDG--
YLVLPVLNG-------E--------AKSSYL---AFIDARSMEMVN--SAEI--P--MIV-PFNLHGRF-------
-------------------------------------FDDLL----------------------------------
----------------------------------------------------------------------------
----------------------------------------------------------------------------
----------------------------------------------------------------------------
----------------------------------------------------------------------------
----------------------------------------------------------------------------
----------------------------------------------------------------------------
----------------------------------------------------------------------------
--------------------------------------------------------------
>Octopus_bimaculoides_BCOL
----------------------------------------------------------------------------
---------------------------------------------------------
MCVPATLFASLLLSVCGLPVSSLTAA-------------------------------------------------
SSALADTF------STLYTTN-LKEF--------N---DF-P--IYF------------------DRK--IPEWL-
KGNLIRNGFG-----QF------ES-----GK---RHFLHLFDSFAKLSSWKI---------TGN----NTA----
--LFSTKFVKS------STYNKSIATDDI----AP-YL----TF-IGVEPPFSR---W------KQFKSIING---
-------L-----------DNMNVNVYSYYKTAKANGQDD--------------------KGCFALT-DYW--
KMYEF----NCTTLQTI--SACEAPVPQK------------------------------SS-IGFLNL--IS----
-------TAHPLPEFG------TNNSLL------------F--LSTLSLI------PWVKS---SVKFIRI-----
--------------------------------HTA---------QK----------REVIANVPVD----KIP---
----YMHSFS-TTEHYGILFCHPM--YLNIPKF----LK------EMH-PFDGM----------------------
---DWYGDKPID-VNIINLK-------TG-------EVI---NMK--------------T-----E--
NSFIMHHANAY----ED-ENG---NIIVDAVGYD--DPLF-----------------VKS--MFVENM-------
VN-------------PAKRKNIPFSA----------------------QIRRYIFNI-------------------
-------------------------------------------ENKTSKVH-------------------------
----SFSRPS------------------------------------------------------------------
----------------------------------------------------------------------------
----------------------------------------------------------------------------
----------------------------------------------------------------------------
----------------------------------------------------------------------------
----------------------------------------------------------------------------
------------------------------NETFLTRF----------------DFPTIN-
ENYRSKPYCFLYGVALRVIHDNIT--------------SIALIKKDVCEK--------GR----------------
---DK--------YYYKTGVY--VSEAIFVPT---P-NAKE---EDDG--VLVLPGADA-------A--------
TNKHYL---YVIDAKTMTLIS--QAEL--P--TTV-PYTLHGHF--------------------------------
------------ISEKL-----------------------------------------------------------
----------------------------------------------------------------------------
```


>Crassostrea_gigas_BCOLb

MIRLLLCCLYFVA---SLAQTPGW--
----NLLFGTS-VLNE------T-H---KY-P--LYF------------------DNP--VPKWI-
KGSLIRNGPA-----RY------EM-----GR---RSFLNLFDGFGKLYSWQF---PG----NGS------A----
--FFSAKFLGS------ECYNESLKINDI----AP-YQ----TF-DDLVPPMSF---F------DKELALYHG---
-------L-----------DNMNINVYNFS--------GD----------------------CVVLS-DTW--
KLYVV----DCYTLDTV--RPSKPGIPGV----------E-------------------TG-FPYISI---MS---
-------VAHPVKEYG------KNGHLT------------I--LQSLNIL------PDMRH---KFTLVRT-----
--------------------------------KSA---------DV----------REKIAEWEVK----KMP---
----YLHSFS-TTERYAIIFGCPF--YIDIEKM----L-------RYSLPKYSI----------------------
---SFHKDEPTI-TYVIDLK-------TG-------KVV---TME--------------T-----E--
NIFTMHHVNAY----EL-DDT---TLVMDVVSYP--DASL-----------------TSF--FEMDIL-------
MN-------------KTRRDNVPFKP----------------------YLRRYKIDL-------------------
---KASKMT-------------------------
--PVSFDINP--

------------------------------KFPFVNML----------------EVPTIN-
EKYRSKHYCFVYGVVFKSDNKIWG--------------NFSFVKKDVCNA--------SG----------------
---DL--------SWSIPGHY--PMEGWFVPN---P-EGLS---EDDG--ILMVPVLDG-------N--------
LGKSYI---LLLDPKTMKPIT--KAIS--P--FQV-PFHFHGRF--------------------------------
------------IENVF---

>Crassostrea_gigas_BCOLc

MVSILQWLILSLCVPD---
IFAQTQGW------NLLFGTT-VPEE------V-V---KK-P--LTF------------------DNP--VPKWI-
KGSLIRNGPG-----RF------EM-----GP---HSFLNVFDGYAKLYSWQF---PG----NGS------V----
--YFSAKFVQS------MFYNESLKIKDI--AR---YQ----TFDDLVPPMSQL----------NKEEAFIRG---
-------L-----------DNMNVNIYNYT--------GE----------------------CVLLT-DVW--
KLYVV----DCHTLKTI--RSCDPPIPGD----------H-------------------TT-FPYISG---MS---
-------VAHPVPEVG------TDYHLT------------I--RQSLAIL------PGMSD---KFTLVRV-----
--------------------------------KSA---------DI----------REKVAEWEVKKMP-------
----YQHSIL-STERYALIFATPL--YMDTKAL----L-------QSFVARQSL----------------------
---IFNKDEKTT-VYVVEIK-------TG-------QVH---TIQ--------------T-----E--
TVFSMHYLNAY----EI-DNN---TIVMDVASYP--DGSV-----------------VAF--FEMDNL-------
MN-------------KTRRDSAPYKP----------------------SLKRYKIDI-------------------

```
---------------------------------------------LGEKIK-------------------------
--PMSFGVNP------------------------------------------------------------------
----------------------------------------------------------------------------
----------------------------------------------------------------------------
----------------------------------------------------------------------------
----------------------------------------------------------------------------
----------------------------------------------------------------------------
------------------------------KVPHVNMM----------------DMPTIN-
ERYRSKPYCYAYGVV-FKSDFKTF-------------GNFSIVKKDVCSS--------FG----------------
---DL--------SWNVADQY--PMESWFVPD---P-NGSR---EDDG--VLLTPMLDG-------K--------
LGKSYL---VILDPKTMKPIS--KAQL--P--ILV-PFHFHGRF--------------------------------
------------IDNVY-----------------------------------------------------------
----------------------------------------------------------------------------
----------------------------------------------------------------------------
----------------------------------------------------------------------------
----------------------------------------------------------------------------
----------------------------------------------------------------------------
----------------------------------------------------------------------------
----------------------------------------------------------------------------
-------------------------------------
>Mimachlamys_nobilis_BCOL
----------------------------------------------------------------------------
----------------------------------------------------------------------------
----------------------------------------------------------------------------
---------------------------------------------MGN----------------------------
---------------RQFVNFFDGYAKLNSWKF---PG----NGS------A------FFSTKFIQS------
KVYTESLAKNDI----AP-YL----TFEGVNPPFDEV----------ERYESFLLN--------------------
-MDNTVVNVFNYS--------NK----------------------IVAMN-DIW--KVYEI----NPHTLDTI--
GVVNPPTPPT----------K-------------------YSNLGRLNV---MS----------TAHPVAEYG---
---TGAKFD------------I--LNTFSLL------PGTKE---RLSVVRV------------------------
-------------TSL---------EG----------RELVAEWEID----QTS-------YMHSFA-
VTPNYVVLLAAPY--YVNMDNM----L-------KYASVVGGM-------------------------
KWNAQDNST-FYVVEIK-------TG-------KVH---TLV--------------S-----G--
NAFVIHHINAF----ELPDD----KIVLDAPVHP--NPYA-----------------FSI--YDLSYI-------
HN-------------KTQRVTLPAIP----------------------VLTRFTLDL-------------------
---------------------------------------------KTQSVQ-------------------------
------RVSF------------------------------------------------------------------
----------------------------------------------------------------------------
----------------------------------------------------------------------------
----------------------------------------------------------------------------
----------------------------------------------------------------------------
----------------------------------------------------------------------------
------------------------------DSGPKAPC--------VGAL----EMPVFN-
ENYRHMNYCIVYGQVINYNGKGFS--------------HIALVKKNVCNN--------AG----------------
---DL--------MYTAPHQY--LTEPWFVPN---P-DGKE---EDDG--VVMASAFDG-------D--------
KQESYL---LMLDPKTMTEIN--RSYM--P--TRI-PFNFHGRF--------------------------------
------------FDIV------------------------------------------------------------
----------------------------------------------------------------------------
----------------------------------------------------------------------------
----------------------------------------------------------------------------
----------------------------------------------------------------------------
----------------------------------------------------------------------------
----------------------------------------------------------------------------
----------------------------------------------------------------------------
-------------------------------------
>Capitella_teleta_BCOLa
```

```
----------------------------------------------------------------------------
------------------------------------------------
MEVADVSHLLLKLPVDHNTDMECILKTFVAWFLFVAMA--------------------------------------
--------AGEMPSAY------VLTFTSN-TETY------E-D---HM----VEL------------------
QNP--LPDWV-NGIYVRNGMG-----VF------EM-----GD---RNLTHPFDGLAKLHSFRL---T-----
GGS----SHV------IYNSQFLES------NFYKDSKSEGRL----AP-YL----VL-GSIVPSFAL---W----
--DKIRALYNG----------I-----------DNTNINVVQFN--------GT---------------------
TLHALS-DFW--HSYEI----NKRDLRTV--RKTEPPLPNA----------G-------------------IL--
NYVAP--IPA----------SSHPAKEFG------TNNYIN------------F--YSLVNVL------PWQSH--
-TLNIIRI-------------------------------------ESS---------TK----------
TELLASIPRI----DIP-------YMHSMA-STKDYAIVIAHPV--YVNWMTI----VR------NQD-PLDSL--
-----------------------EYHADKPTD-IYVVHLH-------TN-------KFY---HLK-----------
---T-----D--SYFFMHNVNAF-------QEGD--KLYVDETSYS--DISL-----------------MHA--
MAFHNI-------RS-------------GSPQWPDNMHS----------------------QIKRLTIDL------
--------------------------------------------------------STETVEVH------------
-----------------NFDTIP-----------------------------------------------------
----------------------------------------------------------------------------
----------------------------------------------------------------------------
----------------------------------------------------------------------------
----------------------------------------------------------------------------
----------------------------------------------------------------------------
-------------------------------------------GLEFVNTL----------------DLPVIN-
PDYSFQRYCYIYGQV-FGDRGLED---------------VKLVKKDICGH--------G-----------------
---DK--------VWFKPNHF--PSESWFQPN---P-NGVD---EDDG--ILLNIVLDG-------D--------
TRTSYL---LFLDAKTFEEIN--VGYL--P--TWI-PFTLHGRF--------------------------------
------------FSHSCSDDELSGS---------------------------------------------------
----------------------------------------------------------------------------
----------------------------------------------------------------------------
----------------------------------------------------------------------------
----------------------------------------------------------------------------
----------------------------------------------------------------------------
----------------------------------------------------------------------------
----------------------------------------------------------------------------
-------------------------------------
>Capitella_teleta_BCOLb
----------------------------------------------------------------------------
----------------------------------------------------------------------------
-----------------------------------------------------------------------
MTFKDH-MFEM---------------------------------------SHP--VPDWM-NGIYLKNGMG-----
LF------DY-----GG---RNFTHPFDALAKLHSFKF---------EGT---SKEV------AFNAQFVKS----
--NFYK----------------------------------------------------------------------
--------IPCFVYG--------GK---------------------SVHALS-DFW--TTYEI----DLSTLDTI-
-KQVTPPMPNA----------G-------------------FA-FGPIPS---------------CAHPVKEFG--
----TDNLIN------------F--YSLINPF--------GSH---SVNVIRI-----------------------
--------------KSS---------DE----------TEVLASISRDEIS-----------YMHSLA-
ATNEYVIILAHPV--YINWKQI------------GKD-AIETL-------------------------
EWHPERLTD-IYAVHLA-------TG-------EVT---HLQ--------------T-----E--
ALFFLHNINAY-------QEGD--YIIVDHTTYT--DMSM-----------------MHH--LSMKNI-------
RS-------------AEADWPKSTRP----------------------QIKRIKINL-------------------
-------------------------------------------TVKSVKID-------------------------
----NFSTQP------------------------------------------------------------------
----------------------------------------------------------------------------
----------------------------------------------------------------------------
----------------------------------------------------------------------------
----------------------------------------------------------------------------
----------------------------------------------------------------------------
------------------------------GLEFVNQL----------------DLPVIN-
```

QNFAFKEYCYVYGMA-AGDSGMQD---------------ARLVKKNLCYG--------VG----------------
---DR--------VWSKENHF--PSEPWFIPN---P-NGSG---EDDG--VLLTIVLDG-------H--------
SDVSYL---LILDGRTFEEVN--LGYL--P--TWI-PYTLHGRF--------------------------------
------------FHHMCSDEKQTCS---

>Capitella_teleta_BCOLc

MESRGGLYYCIVLLFVVNSSCH--
RADHRAGF------VKFFISN-PEEF------------VDVE--VKL------------------QKT--LPRWF-
KGVYIRNGVG-----LF------EV-----GG---RRLLHALDGFTKLTSFNF---TS----PNT------L----
--GFSSKFLRS------GFYNDSTKSKSI----AP-YL----LL-GDVEPHFSL---F------EKLRSFANG---
------------------VDNVNVNVIQFS--------ND-------------------TDSMVAVS-DIW--
RVYEM----NTRGLVTT--KRIDANLPGG----------D-------------------LQ--LRKSM--TLS---
-------TAHPLLEEA------SGAHIG------------F--CVSAMPL------PGFKN---GINVVRI-----
--------------------------------FSA---------ER----------RELVAHIPRK----TIP---
----YMHGLA-LASHYAVIFAHPA--FINLWAV----VS------GGS-LLSSI----------------------
---KWKPNYQMS-IYVVDLQ-------TG-------ATI---RIK--------------T-----S--
PMFVMHFVNSF----T--SKD---NLIIDMITYK--DDSF-----------------MHS--LNLDNL-------
RR----------------GLVPPAAG----------------------TLSRFTINI-------------------
---AERSVS-------------------------
----VDSLPS--

------------------------------TLPQTSHF----------------DLPNIH-
EKLRYKPYCYVYGITSFDFGPRKQ-------------YRVCLVKRDLCSN--------GKG---------------
---DR--------MWCHNARV--PTEPWFEPN---P-NATE---EDDG--LLITLTMDN-------D--------
ENKSHL---VFLDAKRMKMVN--EASL--P--TWV-PFTIHGNM--------------------------------
------------FRRDLYV---

>Lamprey_BCMO1

--GS--VPGWL-RGCLIRNGPG-----RH---
---CV-----GP---SCYQHWFDGLALLRKFRF---R-----DGE------V------WFSSRYLQS------
DTYKKNVAANRI--VVPE-FG----TR-VELDPSLGL---L------EKYFTYLRN----------I------
MPDNTDNCLINVVRYG--------QD----------------------VYACT-ETT--IMRRL----
DPDTLDTL--DK-------V----------N-------------------LA--EKVGM--NML----------
TAHPLYEP-------DGTAYN------------V--ATVIGER--------GRT---KYNLLQI------------

-------------------------PPT---------AA--GTRAKRRQSPIVASIPASSRL-KPS-------
YYHSFG--
---------------------------------------YT--------------------D--AGVFIHHANTY-
---N--EEG---HLVADVTSHD--GDGV-----------------YSV--FYFGKG-------EE-------
EEEEQGAELGSNLVTMS----------------------GTTAQALLQ----------------------------
------------------------------------KKGKIE--------------------------------
CIP---

-----------------------EE----LFPG--------------EFSDLH-LQLHPQSHRNAVVTSGV--
DHPVP---------------SSLLKLDLETR--------E--------------------TR--------
LWEADNGW--CGEPVFVAS---P-DASA---EDDG--VLLCVTASH-------D----A---AHGVDI---
VVIDAREMKELG--RART--P--SLL-PMDMHGIF---
---IPD--

>Lamprey_BCMO2a

MGSSGSVVRLGGLVLGLLWIFLQDFASWLSRLLGLSLLFPRRRSASRVYA--------------------------
------------------KCSGLQCI------APIIATM--TET------P-E---TM-E--AEI-----------
-------YGT--IPAWI-KGTLLRIGPG-----KF------EF-----GP---DSYNHWFDGMALMHRFRV---H-
----DGR------V------SYSSRFLRS------DSYLRNEARQRI--VVSE-FG----TM-AVPDPCKTL---
F------DRFFTYFSP----------P--------APTDNDVVNYVEYK--------KD-----------------
-----VYVSS-ETM--QMRKV----DPRTLDTK--EK-------V----------D-------------------
WG--KFVAV--NSA----------TAHPHYDP-------DGTAYN------------M--GNIFGKE---------
---GTYYAIIRV-----------------------------PVQKDSDGDTL---------EG----------
ATIVTRFPAADSR-FPS-------YYHSFA-MTENYVVFLEQPL--CMDVLQL----LTA-RLR-GRA-YSHCM--
-----------------------VWKPQRGSI-MRVANKH-------TG-------EIL-PVAYH-----------
---M-----K--AMMSFHHINAY----E--DDG---HIVNDLCCVD--HYCT-----------------
ADEDSLMLKNL----------------RSSGDALDQLYNRRPMT----------------------YPRRFVMPL-
-----------------------SVGPDSPRGTNLC--------------------SLPYTTASATLRD-------
--------------------DGKVWLEP--

--EPLHGDDV------EEYGGL----
EFPRIHYERFNARPYRYFYSCG---FQHVIP---------------NSLIKVDVTTK--------E----------
----------TK--------HWQVDGYY--PSEPVFVPS---P-GAEG---EDEG--VVLSVVISP-------DK-
------EKPSFL---LVLDASSFMELG--RAVV--P--RDI-PYGFHGIF--------------------------
------------------APNVV---

>Lamprey_BCMO2b

MGSNGSVVRLGGLVLGLLWIFLQDFASWLSRLLGISLLFPRRRSASRVYA--------------------------
------------------KCSGLQCI------APIIATM--TET------P-E---TM-E--AEI-----------
-------YGT--IPAWI-KGTLLRIGPG-----KF------EF-----GP---DSYNHWFDGMALMHRFRV---H-
----DGR------V------SYSSRFLRS------DSYLRNEARQRI--VVSE-FG----TM-AVPDPCKTL---
F------DRFFTYFSP----------P--------APTDNDVVNYVEYK--------KD-----------------
-----VYVSS-ETT--QMRKV----DPHTLDTK--EK-------V----------D-------------------
WG--KFVAV--NSA----------TAHPHYDP-------DGTAYN------------M--GNIFGKE---------
---GTYYAIIRV-----------------------------PVQKDSDGDTL---------EG----------
ATIVTRFPAADSR-FPS-------YYHSFA-MTENYVVFLEQPL--YMDVLQL----LTA-RLR-GRA-YSHCM--
-----------------------VWKPQRGSI-MRVANKH-------TG-------EIL-PVAYH-----------
---M-----K--AMMSFHHINAY----E--DDG---HIVNDLCCVD--HYCT-----------------
ADEDSLMLKNL----------------RSSGDALDQLYNRRPMT----------------------YPRRFVMPL-
-----------------------SVGPDSPRGTNLC--------------------SLPYTTASATLRD-------
----------------DGK---V-WLEP--

--EPLHGDDV------EEYGGL----
EFPRIHYERFNARPYRYFYSCG---FQHVIP---------------NSLIKVDVTTK--------E----------
----------TK--------HWQVDGYY--PSEPVFVPS---P-GAEG---EDEG--VVLSVVISP-------D--
--K---EKPSFL---LVLDASSFVELG--RAVV--P--RDI-PYGFHGIF--------------------------
------------------APNVV---

Comparison of BCOL and BCO1/2 families (DIVERGE3 output)

Main Tree:
((((((Caenorhabditis_remanei_BCOa:0.061516,Caenorhabditis_elegans_BCOa
:0.137171):0.082584,(Caenorhabditis_remanei_BCOb:0.082717,Caenorhabditis_ele
gans_BCOb:0.090430):0.028865):0.591164,(((((((Lancelet_BCOLa_1:0.000000,Lanc
elet_BCOLa_2:0.000000):0.032150,Lancelet_BCOLd_hybrid:0.019403):0.124558,Lan
celet_BCOLc:0.242265):0.067911,Lancelet_BCOLf:0.239233):0.090040,(Lancelet_B
COLb:0.233242,Lancelet_BCOLg:0.269727):0.226635):0.100215,((Lancelet_BCOLe:0
.511723,(((((Lottia_gigantea_BCOL:0.270358,(Biomphalaria_glabrata_BCOL:0.129
804,Aplysia_californica_BCOL:0.266930):0.054711):0.192482,Crassostrea_gigas_
BCOLa:0.287298):0.102512,(Crassostrea_gigas_BCOLb:0.146423,Crassostrea_gigas
_BCOLc:0.347159):0.215785):0.088855,Octopus_bimaculoides_BCOL:0.477142):0.06
9516,((Capitella_teleta_BCOLa:0.357760,Capitella_teleta_BCOLb:0.193766):0.19

5597,Capitella_teleta_BCOLc:0.720318):0.102847):0.019312):0.037436,Mimachlamys_nobilis_BCOL:0.604929):0.065991):0.269809,((Caenorhabditis_elegans_BCOL:0.052816,Caenorhabditis_remanei_BCOL:0.000000):0.966784,Saccoglossus_kowalevskii_BCOLa:0.483065):0.311939):0.162914):0.079892,(Ciona_BCOa:0.612667,(Nematostella_BCOa:0.273104,Nematostella_BCOb:0.258665):0.272688):0.030644):0.033237,Sea_urchin_BCOa:0.521346):0.026369,((Sea_urchin_BCOc:0.651442,Lancelet_BCOa_lan:0.397966):0.074692,((Lamprey_RPE65:0.187416,((((Human_RPE65:0.005232,(Dog_RPE65:0.008981,Cow_RPE65:0.016337):0.004859):0.018958,(Mouse_RPE65:0.000000,Rat_RPE65:0.000000):0.029282):0.034208,Chicken_RPE65:0.045905):0.108233,(((Zebrafish_RPE65a:0.010717,Zebrafish_RPE65b:0.019772):0.083304,(Zebrafish_RPE65c:0.069606,((((Fugu_RPE65a:0.013946,Pufferfish_RPE65a:0.006236):0.016119,Medaka_RPE65a:0.041548):0.010566,Stickleback_RPE65a:0.032968):0.040911,Atlantic_cod_RPE65a:0.038274):0.015351):0.076217):0.023168,(((Fugu_RPE65b:0.058954,Pufferfish_RPE65b:0.036628):0.049616,(Medaka_RPE65b:0.086641,Stickleback_RPE65b:0.074046):0.009743):0.005307,Atlantic_cod_RPE65b:0.114563):0.029908):0.033023):0.004394):0.313661,Lancelet_BCOb:0.363461):0.011199):0.026892):0.006450,(((((Ciona_BCMOb:0.660093,(((((Human_BCMO1:0.077524,(Dog_BCMO1:0.050967,(Mouse_BCMO1:0.042540,Rat_BCMO1:0.014375):0.007705):0.024322):0.027055,Cow_BCMO1:0.118136):0.176554,Chicken_BCMO1:0.195269):0.070551,(((((Pufferfish_BCMO1a:0.085071,Fugu_BCMO1b:0.081821):0.064338,Stickleback_BCMO1b:0.140213):0.012861,Medaka_BCMO1a:0.138423):0.041971,Atlantic_cod_BCMO1:0.163051):0.028760,Zebrafish_BCMO1a:0.318824):0.077412):0.094096,(Zebrafish_BCMO1:0.153567,(((Pufferfish_BCMO1b:0.045240,Fugu_BCMO1a:0.056034):0.065693,Stickleback_BCMO1a:0.095477):0.013136,Medaka_BCMO1b:0.118004):0.049572):0.166864):0.040665):0.088552,(((((Human_BCO2:0.085849,Dog_BCO2:0.032764):0.028884,Cow_BCO2:0.079106):0.005241,(Mouse_BCO2:0.036870,Rat_BCO2:0.036369):0.052523):0.077643,Chicken_BCO2:0.135493):0.086935,((((Zebrafish_BCO2a:0.111263,Atlantic_cod_BCO2a:0.107146):0.011316,((Pufferfish_BCO2a:0.069317,Fugu_BCO2b:0.043472):0.022411,(Medaka_BCO2b:0.083289,Stickleback_BCO2a:0.059081):0.014349):0.014577):0.032651,(Zebrafish_BCO2b:0.153947,((((Pufferfish_BCO2b:0.189148,Fugu_BCO2a:0.003074):0.050595,Stickleback_BCO2b:0.084373):0.013414,Medaka_BCO2a:0.080002):0.009921,Atlantic_cod_BCO2b:0.075284):0.058224):0.010262):0.068208,((((Pufferfish_BCO2c:0.099723,Fugu_BCO2c:0.132115):0.113175,Medaka_BCO2c:0.147131):0.012326,Stickleback_BCO2c:0.056550):0.020356,Atlantic_cod_BCO2c:0.146455):0.116319):0.027496):0.161336):0.014400,(((((Drosophila_melanogaster_NinaB:0.195498,(Anopheles_gambiae_NinaB:0.047096,Culex_quinquefasciatus_NinaB:0.042837):0.139975):0.058826,Bombyx_mori_NinaB:0.354230):0.031966,((Acromyrmex_echinatior_NinaB:0.210367,Megachile_rotundata_NinaB:0.145831):0.035866,Nasonia_vitripennis_NinaB:0.155851):0.067714):0.048893,Daphnia_pulex_BCOb:0.388777):0.275042,Daphnia_pulex_BCOa:0.485793):0.057489):0.014843,((Sea_urchin_BCOb:0.385201,Saccoglossus_kowalevskii_BCOb:0.307170):0.209765,(Nematostella_BCOc:0.541479,Saccoglossus_kowalevskii_BCOa:0.490578):0.068096):0.017946):0.009314,(Lancelet_BCOc_mito:0.335368,Lancelet_BCOd:0.357002):0.084819):0.006450):0.000000

Cluster BCOL:

(((Lancelet_BCOLe:0.511723,(((((Lottia_gigantea_BCOL:0.270358,(Biomphalaria_glabrata_BCOL:0.129804,Aplysia_californica_BCOL:0.266930):0.054711):0.192482,Crassostrea_gigas_BCOLa:0.287298):0.102512,(Crassostrea_gigas_BCOLb:0.146423,Crassostrea_gigas_BCOLc:0.347159):0.215785):0.088855,Octopus_bimaculoides_BCOL:0.477142):0.069516,((Capitella_teleta_BCOLa:0.357760,Capitella_teleta_BCOLb:0.193766):0.195597,Capitella_teleta_BCOLc:0.720318):0.102847):0.019312):0.037436,Mimachlamys_nobilis_BCOL:0.604929):0.065991,(((((Lancelet_BCOLa_1:0.000000,Lancelet_BCOLa_2:0.000000):0.032150,Lancelet_BCOLd_hybrid:0.019403):0.124558,Lancelet_BCOLc:0.242265):0.067911,Lancelet_BCOLf:0.239233):0.090040,(Lancelet_BCOLb:0.233242,Lancelet_BCOLg:0.269727):0.226635):0.100215,((Caenorhabditis_elegans_BCOL:0.052816,Caenorhabditis_remanei_BCOL:0.000000):0.966784,Saccoglossus_kowalevskii_BCOLa:0.483065):0.311939):0.000000

Cluster BCO1_2:

((Ciona_BCMOb:0.660093,(((((Human_BCMO1:0.077524,(Dog_BCMO1:0.050967,(Mouse_BCMO1:0.042540,Rat_BCMO1:0.014375):0.007705):0.024322):0.027055,Cow_BCMO1:0.118136):0.176554,Chicken_BCMO1:0.195269):0.070551,(((((Pufferfish_BCMO1a:0.085071,Fugu_BCMO1b:0.081821):0.064338,Stickleback_BCMO1b:0.140213):0.012861,Medaka_BCMO1a:0.138423):0.041971,Atlantic_cod_BCMO1:0.163051):0.028760,Zebrafish_BCMO1a:0.318824):0.077412):0.094096,(Zebrafish_BCMO1:0.153567,(((Pufferfish_BCMO1b:0.045240,Fugu_BCMO1a:0.056034):0.065693,Stickleback_BCMO1a:0.095477):0.013136,Medaka_BCMO1b:0.118004):0.049572):0.166864):0.040665):0.088552,((((Zebrafish_BCO2a:0.111263,Atlantic_cod_BCO2a:0.107146):0.011316,((Pufferfish_BCO2a:0.069317,Fugu_BCO2b:0.043472):0.022411,(Medaka_BCO2b:0.083289,Stickleback_BCO2a:0.059081):0.014349):0.014577):0.032651,(Zebrafish_BCO2b:0.153947,((((Pufferfish_BCO2b:0.189148,Fugu_BCO2a:0.003074):0.050595,Stickleback_BCO2b:0.084373):0.013414,Medaka_BCO2a:0.080002):0.009921,Atlantic_cod_BCO2b:0.075284):0.058224):0.010262):0.068208,((((Pufferfish_BCO2c:0.099723,Fugu_BCO2c:0.132115):0.113175,Medaka_BCO2c:0.147131):0.012326,Stickleback_BCO2c:0.056550):0.020356,Atlantic_cod_BCO2c:0.146455):0.116319):0.027496,((((Human_BCO2:0.085849,Dog_BCO2:0.032764):0.028884,Cow_BCO2:0.079106):0.005241,(Mouse_BCO2:0.036870,Rat_BCO2:0.036369):0.052523):0.077643,Chicken_BCO2:0.135493):0.086935):0.000000

Da	9.227639
Db	7.476999
N	64.000000
C	64.000000
R	73.000000
Alpha ML	2.196931
Theta-II	-2.201043
Theta SE	1.413632
Ar	1.143538
PIr	0.312130
p	0.681592
d	1.144422
W	0.007771
Z	0.008842
Gr	0.532847
Gc	0.467153
h	0.057090
Q	0.986486
F00,N	0.024876
F00,R	0.000000
F00,C	0.000000

275	0.000000
406	-3.778622
407	-2.985973
408	-3.359637
409	0.174952
410	0.216879
411	-3.322071
412	0.178887
432	0.202989
433	-3.562381
434	-3.761860
435	-3.489449
436	-4.936293

437	0.000000
438	0.000000
439	0.000000
440	0.000000
449	0.218944
450	0.171665
451	0.297963
452	0.000000
453	0.200089
454	-4.004417
455	0.227718
456	0.219133
457	0.202081
458	0.172908
459	-2.804478
472	0.222732
473	0.187484
512	0.000000
513	0.176325
514	0.180772
517	-3.410240
518	-3.651542
519	0.000000
520	0.000000
521	-6.124757
522	0.196392
523	0.289867
524	-3.153110
526	-3.130724
527	-3.335381
528	-3.109924
532	0.000000
533	0.000000
534	0.280149
535	0.000000
536	0.000000
537	0.000000
538	-3.989050
539	0.203842
540	0.325553
541	0.000000
542	0.000000
543	0.000000
544	0.205248
545	0.217145
546	0.354157
547	0.238370
548	-6.088099
549	-4.149359
550	0.000000
551	0.185295
552	-4.529022
553	0.194843
554	-5.003950
555	0.204608
556	-3.010802
557	0.000000

558	0.205361
578	0.205830
580	0.000000
581	-2.789033
582	0.000000
583	-3.105358
584	-2.921759
585	0.170531
586	0.000000
587	-2.953383
588	0.230667
589	-5.087886
590	0.208196
591	0.000000
592	-3.013132
593	-4.236065
594	0.169158
595	0.000000
596	0.000000
597	0.000000
598	0.000000
599	-3.059072
603	-3.258240
604	0.188059
605	-2.797687
620	0.000000
621	-3.354928
625	0.171164
626	0.166122
627	0.299224
628	-3.481176
629	-4.681056
630	0.000000
631	-5.677181
632	0.243202
633	0.000000
634	0.000000
635	0.218281
636	0.000000
639	0.172322
640	0.000000
641	0.000000
645	-3.277534
646	0.220371
647	0.206336
648	0.186809
649	0.000000
650	0.196823
651	-3.812147
652	0.191641
653	-5.055948
654	-3.280950
655	0.000000
656	0.189979
681	-3.275488
682	0.159649
683	-2.977405

684	-3.031167
685	-2.994859
686	0.167030
687	-3.350041
688	-3.091624
689	-4.361367
690	0.172852
691	0.000000
692	0.000000
693	-4.314137
694	0.263530
695	-5.423909
696	0.000000
842	0.345328
843	0.260770
844	0.000000
845	-3.679185
846	0.000000
847	0.000000
852	-5.296206
857	-5.291562
858	0.000000
859	0.195356
860	0.000000
861	0.000000
862	-3.253920
863	-3.953763
870	-3.273065
871	-3.146133
880	0.000000
881	0.223887
882	0.000000
883	-3.674922
884	-6.482488
885	0.201057
886	-3.512089
888	-3.891558
890	-3.029938
893	-3.510110
894	-3.019207
895	0.163892
896	0.000000
897	-2.781275
899	-3.001113
900	-2.903518
901	0.185753
902	-3.852409
903	0.000000
904	0.000000
907	0.000000
908	0.000000
909	0.000000
910	0.230076
912	0.000000
913	0.000000
914	0.000000
915	0.177734

916	0.000000
917	0.175690
918	-4.226335
919	-2.883358
920	0.000000
922	0.000000
923	0.000000
924	0.000000
925	0.000000
929	0.219108
930	0.000000
931	-3.564831
932	-3.984051
933	0.000000
934	0.211366
935	-4.832304
936	0.199906

Comparison of BCOL and NinaB families (DIVERGE3 output)

Main Tree:

(((((((((((((((((Stickleback_RPE65b:0.068399,Atlantic_cod_RPE65b:0.098566):0.023433,Medaka_RPE65b:0.087845):0.011463,(Fugu_RPE65b:0.041910,Pufferfish_RPE65b:0.059152):0.048048):0.079707,(Zebrafish_RPE65a:0.014403,Zebrafish_RPE65b:0.082942):0.066174):0.040581,(Zebrafish_RPE65c:0.068700,(Atlantic_cod_RPE65a:0.061582,(Stickleback_RPE65a:0.037982,(Medaka_RPE65a:0.048462,(Fugu_RPE65a:0.046243,Pufferfish_RPE65a:0.035091):0.040925):0.013698):0.010139):0.023015):0.078251):0.065579,((Mouse_RPE65:0.006208,Rat_RPE65:0.018627):0.031675,(Human_RPE65:0.007908,(Dog_RPE65:0.010112,(Cow_RPE65:0.021034,Chicken_RPE65:0.080586):0.035205):0.007034):0.005327):0.081432):0.053838,Lamprey_RPE65:0.156962):0.279142,(BCOa_Oikopleura_dioica:0.385725,(BCOb_Oikopleura_dioica:0.038100,BCOc_Oikopleura_dioica:0.108348):0.475331):0.815430):0.110567,(Ciona_BCOa:0.697061,(BCOd_Oikopleura_dioica:0.738757,((Caenorhabditis_remanei_BCOa:0.098374,Caenorhabditis_elegans_BCOa:0.137568):0.071588,(Caenorhabditis_remanei_BCOb:0.093518,Caenorhabditis_elegans_BCOb:0.094543):0.070622):0.685540):0.086381):0.046007):0.090757,((Lamprey_BCMO2a:0.010001,Lamprey_BCMO2b:0.000000):0.368181,(((((Human_BCO2:0.078959,Dog_BCO2:0.063425):0.016634,Cow_BCO2:0.107281):0.014975,(Mouse_BCO2:0.030337,Rat_BCO2:0.035459):0.076077):0.146776,Chicken_BCO2:0.170301):0.119362,(((((Pufferfish_BCO2c:0.123886,Fugu_BCO2c:0.218005):0.123015,Medaka_BCO2c:0.160912):0.023871,Stickleback_BCO2c:0.055828):0.041821,Atlantic_cod_BCO2c:0.250613):0.166014,(((((Pufferfish_BCO2a:0.109878,Fugu_BCO2b:0.026817):0.061475,Stickleback_BCO2a:0.070147):0.012303,Medaka_BCO2b:0.093500):0.051329,(Zebrafish_BCO2a:0.138143,Atlantic_cod_BCO2a:0.101265):0.017697):0.091177,(Zebrafish_BCO2b:0.151383,(Atlantic_cod_BCO2b:0.118867,((Pufferfish_BCO2b:0.155487,Fugu_BCO2a:0.049695):0.059085,(Medaka_BCO2a:0.094419,Stickleback_BCO2b:0.091750):0.014665):0.035792):0.095386):0.024917):0.083977):0.073192):0.099133):0.193859):0.064861,((((((((Anopheles_gambiae_NinaB:0.053246,Culex_quinquefasciatus_NinaB:0.072737):0.125444,Drosophila_melanogaster_NinaB:0.346824):0.069614,(Nasonia_vitripennis_NinaB:0.213033,(Acromyrmex_echinatior_NinaB:0.252461,Megachile_rotundata_NinaB:0.119542):0.061391):0.121200):0.103129,Daphnia_pulex_BCOb:0.451745):0.002495,Bombyx_mori_NinaB:0.326058):0.384823,Saccoglossus_kowalevskii_BCOa:0.504167):0.037330,Daphnia_pulex_BCOa:0.676980):0.097126,(Lamprey_BCMO1:0.766243,(Ciona_BCMOb:0.690367,((((Pufferfish_BCMO1b:0.055860,Fugu_BCMO1a:0.044936):0.077133,(Stick

leback_BCMO1a:0.120260,Medaka_BCMO1b:0.115422):0.016085):0.083308,Zebrafish_
BCMO1:0.166726):0.174391,((((((Mouse_BCMO1:0.026224,Rat_BCMO1:0.022086):0.02
7119,Human_BCMO1:0.063069):0.010674,Dog_BCMO1:0.042785):0.033148,Cow_BCMO1:0
.091730):0.145080,Chicken_BCMO1:0.150599):0.089086,(Zebrafish_BCMO1a:0.21314
3,(Atlantic_cod_BCMO1:0.189449,(Stickleback_BCMO1b:0.109402,(Medaka_BCMO1a:0
.164722,(Pufferfish_BCMO1a:0.071483,Fugu_BCMO1b:0.076833):0.069118):0.023701
):0.043795):0.094504):0.137426):0.060970):0.142583):0.071356):0.135389):0.05
9423):0.071319,((Lancelet_BCOc_mito:0.359059,Lancelet_BCOd:0.381356):0.16903
9,((Sea_urchin_BCOb:0.421297,Saccoglossus_kowalevskii_BCOb:0.307805):0.28198
4,(Sea_urchin_BCOa:0.582328,(Sea_urchin_BCOc:0.624705,Lancelet_BCOa_lan:0.43
3963):0.101182):0.025529):0.036530):0.036661):0.014307,Lancelet_BCOb:0.50370
5):0.130960,(Nematostella_BCOc:0.556819,(Nematostella_BCOa:0.235924,Nematost
ella_BCOb:0.338452):0.357926):0.076491):0.172148,(((Caenorhabditis_elegans_B
COL:0.017283,Caenorhabditis_remanei_BCOL:0.017703):0.910407,Saccoglossus_kow
alevskii_BCOLa:0.707730):0.469603,(((((Lancelet_BCOLa_1:0.000000,Lancelet_BC
OLa_2:0.085379):0.026099,Lancelet_BCOLd_hybrid:0.042814):0.208354,(Lancelet_
BCOLc:0.195020,Lancelet_BCOLf:0.248298):0.069831):0.191544,(Lancelet_BCOLb:0
.255158,Lancelet_BCOLg:0.274512):0.162605):0.288094,((((Capitella_teleta_BCO
La:0.281278,Capitella_teleta_BCOLb:0.322075):0.294172,Capitella_teleta_BCOLc
:0.656880):0.205643,Lancelet_BCOLe:0.572981):0.022463,(Mimachlamys_nobilis_B
COL:0.607307,(((Crassostrea_gigas_BCOLb:0.176715,Crassostrea_gigas_BCOLc:0.2
61322):0.331106,Octopus_bimaculoides_BCOL:0.503634):0.051622,(Lottia_gigante
a_BCOL:0.282045,(Crassostrea_gigas_BCOLa:0.411239,(Biomphalaria_glabrata_BCO
L:0.246389,Aplysia_californica_BCOL:0.294151):0.200533):0.044692):0.133471):
0.093595):0.147926):0.078673):0.386853):0.192683):0.087855,(((CCO_Long_Blast
omyces_gilchristii_SLH14081:0.003350,CCO_Blastomyces_gilchristii_SLH14081:0.
007252):0.355941,CCO_Pseudogymnoascus_sp_VKMF4246:0.339285):0.533062,(CCO_As
pergillus_oryzae_RIB40:0.447466,(CAO2_torulene_oxygenase_Neurospora_crassa_O
R74A:0.345740,(torulene_oxygenase_Beauveria_bassiana_ARSEF2860:0.307199,CarT
_torulene_oxygenase_Fusarium_fujikuroi:0.379351):0.160276):0.200831):0.24817
7):0.313545):0.102027,((CCO_Coccomyxa_subellipsoidea:0.506961,CCD8_Monoraphi
dium_neglectum:0.818701):0.161076,(CCD8_moss_Physcomitrella_patens:0.299400,
(CCD8_Arabidopsis_thaliana:0.219495,((CCD8_Saccharum_ROC22:0.030025,CCD8_Zea
_mays:0.052563):0.158550,(CCD8B_Prunus_mume:0.121165,(SiCCD8_Solanum_lycoper
sicum:0.051035,CCD8_DAD1_Petunia_hybrida:0.064298):0.051689):0.046144):0.093
222):0.142349):0.488286):0.266169):0.014082,((BCO1_Halococcus_sediminicola:0
.446075,LSD_Haloterrigena_salina:0.299290):0.166361,((BCO1_Natronomonas_mool
apensis:0.210278,LSDL_Natronococcus_occultus:0.212016):0.380163,(LSDL_Haloqu
adratum_walsbyi_J07HQW2:0.296730,(RPE_Haloarcula_amylolytica:0.052269,RPE_Ha
loarcula_japonica:0.052060):0.173692):0.384941):0.160100):0.188708):0.206633
,((PpCCO_Plesiocystis_pacifica:0.887074,NSC3_NosACO_Nostoc_sp._PCC_7120:0.66
1857):0.358232,(ACO_Auxenochlorella_protothecoides:0.840998,((((((((NSC2_No
sACO_Nostoc_sp._PCC_7120:0.148284,ACO_Hapalosiphon_sp._MRB220:0.118564):0.06
2547,ACO_Scytonema_hofmannii_PCC_7110:0.158399):0.132262,ACO_Microcystis_aer
uginosa:0.251511):0.021960,ACO_Synechococcus_sp._PCC73109:0.319991):0.091788
,ACO_Cyanobacterium_aponinum:0.288193):0.044068,ACO_Leptolyngbya_valderiana:
0.301027):0.075143,ACO_Synechocystis_sp._PCC6803:0.307177):0.113213,ACO_Syne
chococcus_sp._KORDI52:0.539785):0.357503,((((CCD7_Nicotiana_tomentosiformis:
0.034985,CCD7_Petunia_hybrida:0.106436):0.050422,SiCCD7_Solanium_lycopersicu
m:0.058807):0.246828,CCD7_Zea_mays:0.308886):0.877188,((((((((((LSD_Aspergi
llus_oryzae:0.123736,LSD_Rco_Aspergillus_fumigatus:0.088856):0.084942,LSD_As
pergillus_kawachii_IFO4308:0.232950):0.095711,(CAO1_CCO_Neurospora_crassa_OR
74A:0.008708,LSD_Rco_CAO1_Neurospora_crassa:0.002803):0.359338):0.025362,LSD
_Colletotrichum_gloeosporioides_Naragc5:0.223524):0.264679,LSD_Rco_Ustilago_
maydis:0.799605):0.102349,LSD_Rco_Chaetomium_globosum:0.519257):0.143656,(NO
V1_LSD_Novosphingobium_aromaticivorans:0.340764,(SpaLSDI_Sphingomonas_paucim
obilis:0.217654,SpaLSDIII_Sphingomonas_paucimobilis:0.188135):0.141963):0.21

7110):0.120984,(isoeugenol_monooxygenase_Pseudomonas_putida:0.167913,isoeuge
nol_monooxygenase_Pseudomonas_nitroreducens:0.129387):0.432792):0.045160,(LS
D_Sphingobium_sp_SYK6:0.388999,NOV2_LSD_Novosphingobium_aromaticivorans:0.30
8304):0.529265):0.245829,(Blr5790_pLSD_Bradyrhizobium_diazoefficiens:0.14758
5,RpsCCO_Rhodopseudomonas_palustris:0.187105):0.477112):0.231338,((SaCCO_Sph
ingopyxis_alaskensis:0.527825,MtCCO_Mycobacterium_tuberculosis:0.618367):0.3
08636,(PPSIR1_17230_CCO_Plesiocystis_pacifica_SIR-
1:0.882277,(((CCD_Ostreococcus_lucimarinus_CCE9901:0.725621,CCD1like_Zea_may
s:0.961814):0.252061,(MtLSD_Mycobacterium_tuberculosis:0.460148,Sala_1008CCO
_Sphingopyxis_alaskensis:0.333858):0.497316):0.064578,(NSC1_NosCCD_Nostoc_sp
._PCC_7120:0.415816,(CarX_CCO_Fusarium_fujikuroi:1.004756,((CCD1_Zea_mays:0.
107407,CCD1_Arabidopsis_thaliana:0.121380):0.420187,((((ZCDlong_Zea_mays:0.0
54191,ZCD_Zea_mays:0.026960):0.201458,NCED4_Arabidopsis_thaliana:0.331303):0
.116672,(CCD4a_CsZCD_Crocus_sativus:0.058217,CCD4b_CsZCD_Crocus_sativus:0.02
8859):0.305676):0.395002,(NCED6_Arabidopsis_thaliana:0.303802,((NCED1_Zea_ma
ys:0.156037,VP14_Zea_mays:0.184512):0.090039,((NCED9_Arabidopsis_thaliana:0.
195941,NCED3_Arabidopsis_thaliana:0.115309):0.044526,(NCED5_Arabidopsis_thal
iana:0.147493,NCED2_Arabidopsis_thaliana:0.177308):0.065273):0.085851):0.108
712):0.300292):0.252979):0.191806):0.077731):0.108284):0.073833):0.078074):0
.083978):0.242261):0.178638):0.030721):0.068440):0.234904):0.000000
Cluster BCOL:
(((((Capitella_teleta_BCOLa:0.281278,Capitella_teleta_BCOLb:0.322075):0
.294172,Capitella_teleta_BCOLc:0.656880):0.205643,Lancelet_BCOLe:0.572981):0
.022463,(Mimachlamys_nobilis_BCOL:0.607307,(((Crassostrea_gigas_BCOLb:0.1767
15,Crassostrea_gigas_BCOLc:0.261322):0.331106,Octopus_bimaculoides_BCOL:0.50
3634):0.051622,(Lottia_gigantea_BCOL:0.282045,(Crassostrea_gigas_BCOLa:0.411
239,(Biomphalaria_glabrata_BCOL:0.246389,Aplysia_californica_BCOL:0.294151):
0.200533):0.044692):0.133471):0.093595):0.147926):0.078673,((((Lancelet_BCOL
a_1:0.000000,Lancelet_BCOLa_2:0.085379):0.026099,Lancelet_BCOLd_hybrid:0.042
814):0.208354,(Lancelet_BCOLc:0.195020,Lancelet_BCOLf:0.248298):0.069831):0.
191544,(Lancelet_BCOLb:0.255158,Lancelet_BCOLg:0.274512):0.162605):0.288094,
((Caenorhabditis_elegans_BCOL:0.017283,Caenorhabditis_remanei_BCOL:0.017703)
:0.910407,Saccoglossus_kowalevskii_BCOLa:0.707730):0.469603):0.000000
Cluster NinaB:
((((Anopheles_gambiae_NinaB:0.053246,Culex_quinquefasciatus_NinaB:0.072
737):0.125444,Drosophila_melanogaster_NinaB:0.346824):0.069614,(Nasonia_vitr
ipennis_NinaB:0.213033,(Acromyrmex_echinatior_NinaB:0.252461,Megachile_rotun
data_NinaB:0.119542):0.061391):0.121200):0.103129,Daphnia_pulex_BCOb:0.45174
5,Bombyx_mori_NinaB:0.326058):0.000000

	BCOL/NinaB
Da	7.672250
Db	1.831324
N	28.000000
C	20.000000
R	25.000000
Alpha ML	1.297794
Theta-II	-0.435690
Theta SE	0.561515
Ar	0.296119
PIr	0.312130
p	0.616438
d	0.958255
W	0.057249
Z	0.063926
Gr	0.555556
Gc	0.444444

h	0.081487
Q	0.961538
F00,N	0.082192
F00,R	0.000000
F00,C	0.000000

329	-0.517169
330	0.000000
331	-1.148493
332	0.000000
333	0.000000
334	-0.850410
335	0.000000
336	-0.377433
337	-0.557194
363	-0.380811
364	-1.244640
520	-0.390091
521	-0.331941
522	0.000000
523	0.000000
524	-0.375755
525	0.000000
526	-0.342941
594	-1.129901
595	0.000000
596	0.000000
597	-0.411942
598	0.000000
599	-0.715985
626	-0.489564
629	-0.470847
630	-0.351227
631	-0.412054
632	-0.398342
633	-0.366475
634	-0.359010
721	-0.515786
722	0.000000
723	-0.377902
724	-0.483790
725	-0.417972
743	0.000000
744	0.000000
745	-1.203123
746	0.000000
747	-0.480494
882	0.000000
883	-0.388815
884	0.000000
885	-0.401173
886	-0.761002
887	0.000000
888	-0.516538
889	-0.561660
890	0.000000

891 -0.581504
892 -0.513791
1005 0.000000
1006 -0.652992
1007 0.000000
1008 0.000000
1009 0.000000
1010 -0.409944
1011 -0.614663
1670 -0.568003
1671 -0.769533
1672 -0.468086
1673 -0.413648
1674 -0.338533
1675 -0.381077
1726 0.000000
1727 0.000000
1728 0.000000
1729 -0.515306
1730 0.000000
1731 0.000000
1732 0.000000
1733 -0.347082

Comparison of BCOL and fungal CCO families (DIVERGE3 output)

Main Tree:

((((((((Caenorhabditis_remanei_BCOa:0.078308,Caenorhabditis_elegans_BCOa:0.126423):0.059919,(Caenorhabditis_remanei_BCOb:0.083016,Caenorhabditis_elegans_BCOb:0.072495):0.022542):0.707873,(Lamprey_RPE65:0.180365,((((Human_RPE65:0.004410,(Dog_RPE65:0.009123,Cow_RPE65:0.015447):0.005384):0.019800,(Mouse_RPE65:0.002214,Rat_RPE65:0.002634):0.027179):0.030062,Chicken_RPE65:0.053235):0.103305,(((Zebrafish_RPE65a:0.010764,Zebrafish_RPE65b:0.018823):0.088446,(Zebrafish_RPE65c:0.066347,((((Fugu_RPE65a:0.013104,Pufferfish_RPE65a:0.006485):0.015453,Medaka_RPE65a:0.040455):0.010147,Stickleback_RPE65a:0.032051):0.035304,Atlantic_cod_RPE65a:0.035744):0.015792):0.078513):0.018201,(((Fugu_RPE65b:0.059114,Pufferfish_RPE65b:0.039020):0.046808,(Medaka_RPE65b:0.091588,Stickleback_RPE65b:0.075936):0.001685):0.005735,Atlantic_cod_RPE65b:0.111628):0.032886):0.029573):0.025178):0.265573):0.048426,(Sea_urchin_BCOa:0.469961,((Nematostella_BCOa:0.241105,Nematostella_BCOb:0.241858):0.271283,((((((((Lancelet_BCOLa_1:0.009145,Lancelet_BCOLa_2:0.025493):0.030045,Lancelet_BCOLd_hybrid:0.026551):0.122365,Lancelet_BCOLc:0.230811):0.089944,Lancelet_BCOLf:0.212561):0.090327,(Lancelet_BCOLb:0.255103,Lancelet_BCOLg:0.264442):0.241981):0.125034,(Lancelet_BCOLe:0.491746,((((((Lottia_gigantea_BCOL:0.268442,(Biomphalaria_glabrata_BCOL:0.153670,Aplysia_californica_BCOL:0.252391):0.077078):0.183483,Crassostrea_gigas_BCOLa:0.304197):0.072679,(Crassostrea_gigas_BCOLb:0.114956,Crassostrea_gigas_BCOLc:0.350295):0.242208):0.091164,Octopus_bimaculoides_BCOL:0.474052):0.106494,((Capitella_teleta_BCOLa:0.349992,Capitella_teleta_BCOLb:0.217617):0.210471,Capitella_teleta_BCOLc:0.733884):0.084347):0.030811,Mimachlamys_nobilis_BCOL:0.576259):0.035432):0.082251):0.235212,((Caenorhabditis_elegans_BCOL:0.041149,Caenorhabditis_remanei_BCOL:0.000000):0.963833,Saccoglossus_kowalevskii_BCOLa:0.445334):0.342962):0.162669,(((CCO_Long_Blastomyces__gilchristii_SLH14081:0.000000,CCO_Blastomyces__gilchristii_SLH14081:0.000000):0.339034,CCO_Pseudogymnoascus_sp_VKMF4246:0.379464):0.216457,(CCO_Aspergillus_oryzae_RIB40:0.431799,((torulene_oxygenase_Beauveri

a_bassiana_ARSEF2860:0.230975,CAO2_torulene_oxygenase_Neurospora_crassa_OR74A:0.452913):0.073711,CarT_torulene_oxygenase_Fusarium_fujikuroi:0.225791):0.155776):0.217214):0.315790):0.029317):0.045234):0.037690):0.022705,Lancelet_BCOb:0.409482):0.011139,(((((Drosophila_melanogaster_NinaB:0.191190,(Anopheles_gambiae_NinaB:0.035026,Culex_quinquefasciatus_NinaB:0.052139):0.159381):0.040431,Bombyx_mori_NinaB:0.375938):0.027093,((Acromyrmex_echinatior_NinaB:0.196648,Megachile_rotundata_NinaB:0.139061):0.051449,Nasonia_vitripennis_NinaB:0.140099):0.059428):0.050071,Daphnia_pulex_BCOb:0.393630):0.256959,Daphnia_pulex_BCOa:0.465683):0.059653):0.007702,((Ciona_BCMOb:0.651687,(((((Human_BCMO1:0.076166,(Dog_BCMO1:0.054444,(Mouse_BCMO1:0.037389,Rat_BCMO1:0.012617):0.013203):0.013918):0.022900,Cow_BCMO1:0.111606):0.177801,Chicken_BCMO1:0.192667):0.072463,(((((Pufferfish_BCMO1a:0.077019,Fugu_BCMO1b:0.072555):0.064961,Stickleback_BCMO1b:0.152230):0.003176,Medaka_BCMO1a:0.128795):0.048829,Atlantic_cod_BCMO1:0.146217):0.028937,Zebrafish_BCMO1a:0.315472):0.081008):0.083199,(Zebrafish_BCMO1:0.155092,(((Pufferfish_BCMO1b:0.044397,Fugu_BCMO1a:0.053737):0.073097,Medaka_BCMO1b:0.118485):0.006828,Stickleback_BCMO1a:0.093697):0.032151):0.190143):0.044769):0.103806,((Sea_urchin_BCOb:0.397234,Saccoglossus_kowalevskii_BCOb:0.309575):0.200031,(Nematostella_BCOc:0.551231,Saccoglossus_kowalevskii_BCOa:0.524184):0.080672):0.017999):0.019277):0.003421,(((((Human_BCO2:0.083575,Dog_BCO2:0.031319):0.033165,Cow_BCO2:0.080432):0.011441,(Mouse_BCO2:0.044303,Rat_BCO2:0.042863):0.054880):0.074917,Chicken_BCO2:0.139509):0.096246,(((((Zebrafish_BCO2a:0.112115,Atlantic_cod_BCO2a:0.105426):0.007415,((Pufferfish_BCO2a:0.067265,Fugu_BCO2b:0.042000):0.019885,(Medaka_BCO2b:0.077348,Stickleback_BCO2a:0.054687):0.015476):0.014277):0.025156,((((Pufferfish_BCO2b:0.174140,Fugu_BCO2a:0.000000):0.059521,Stickleback_BCO2b:0.077421):0.010048,Medaka_BCO2a:0.068933):0.011863,Atlantic_cod_BCO2b:0.072782):0.054253):0.017336,Zebrafish_BCO2b:0.160777):0.082939,((((Pufferfish_BCO2c:0.094252,Fugu_BCO2c:0.136311):0.094651,Medaka_BCO2c:0.137097):0.019998,Stickleback_BCO2c:0.053263):0.027581,Atlantic_cod_BCO2c:0.154554):0.117859):0.022286):0.171500):0.013318,(((Sea_urchin_BCOc:0.606492,Lancelet_BCOa_lan:0.417876):0.035869,Ciona_BCOa:0.642211):0.057809,(Lancelet_BCOc_mito:0.303004,Lancelet_BCOd:0.336773):0.077525):0.013318):0.000000

Cluster CCO_Fungi:

(((CCO_Long_Blastomyces__gilchristii_SLH14081:0.000000,CCO_Blastomyces__gilchristii_SLH14081:0.000000):0.339034,CCO_Pseudogymnoascus_sp_VKMF4246:0.379464):0.216457,((torulene_oxygenase_Beauveria_bassiana_ARSEF2860:0.230975,CAO2_torulene_oxygenase_Neurospora_crassa_OR74A:0.452913):0.073711,CarT_torulene_oxygenase_Fusarium_fujikuroi:0.225791):0.155776,CCO_Aspergillus_oryzae_RIB40:0.431799):0.000000

Cluster BCOL:

(((((((Lottia_gigantea_BCOL:0.268442,(Biomphalaria_glabrata_BCOL:0.153670,Aplysia_californica_BCOL:0.252391):0.077078):0.183483,Crassostrea_gigas_BCOLa:0.304197):0.072679,(Crassostrea_gigas_BCOLb:0.114956,Crassostrea_gigas_BCOLc:0.350295):0.242208):0.091164,Octopus_bimaculoides_BCOL:0.474052):0.106494,((Capitella_teleta_BCOLa:0.349992,Capitella_teleta_BCOLb:0.217617):0.210471,Capitella_teleta_BCOLc:0.733884):0.084347):0.030811,Mimachlamys_nobilis_BCOL:0.576259):0.035432,(((((Lancelet_BCOLa_1:0.009145,Lancelet_BCOLa_2:0.025493):0.030045,Lancelet_BCOLd_hybrid:0.026551):0.122365,Lancelet_BCOLc:0.230811):0.089944,Lancelet_BCOLf:0.212561):0.090327,(Lancelet_BCOLb:0.255103,Lancelet_BCOLg:0.264442):0.241981):0.125034,Lancelet_BCOLe:0.491746):0.000000

Da	2.682979
Db	7.732862
N	65.000000
C	58.000000
R	84.000000
Alpha ML	2.472222

Theta-II	-2.254916
Theta SE	1.004125
Ar	1.145070
PIr	0.312130
p	0.685990
d	0.685990
W	0.014842
Z	0.016872
Gr	0.591549
Gc	0.408451
h	0.050537
Q	0.988235
F00,N	0.048309
F00,R	0.000000
F00,C	0.000000

360	-12.136168
361	0.388110
362	-4.888587
363	0.346858
532	-4.426967
533	0.237010
534	0.202152
535	-3.101799
536	0.279738
537	0.194003
538	-3.913871
553	0.236523
558	-3.840078
559	0.221794
560	-4.702856
561	0.249992
562	0.000000
563	-6.227069
564	-6.804753
565	0.000000
566	-15.084134
575	-4.393061
576	-4.431382
577	0.386951
578	0.000000
579	0.000000
580	-5.119018
581	-5.227580
582	-4.471446
583	0.267135
584	0.241894
585	-4.037970
586	-3.913630
598	0.289229
599	-4.783691
638	-5.107096
639	-4.089645
640	-3.990673
643	-6.781373
644	0.000000
645	0.000000

646	0.312456
647	0.000000
648	0.283488
649	0.384277
650	-4.767216
652	-4.741535
658	-7.671733
659	0.000000
660	0.488014
661	0.000000
662	0.000000
663	0.379360
664	0.380558
665	0.247266
666	0.000000
667	0.000000
668	0.000000
669	0.000000
670	0.345872
671	0.283860
672	0.000000
673	-6.011464
674	0.347184
675	-5.146848
676	0.520569
677	-4.055540
678	-6.625739
679	0.236292
680	0.344556
681	-3.902906
682	-3.302861
683	-4.476787
684	0.253094
699	-3.943131
700	0.244301
703	-4.773360
707	0.482988
708	-3.849438
709	0.000000
710	-4.365002
711	-4.044340
712	-4.790439
713	0.000000
714	-4.125340
715	0.289351
716	0.000000
717	0.000000
718	0.000000
719	-5.156293
720	-6.683552
721	-4.609488
722	-5.676065
723	0.000000
724	0.000000
725	-6.060427
726	-3.329961
731	0.251575

748	0.354772
749	-3.683235
750	-4.193584
751	0.213563
752	0.000000
753	-4.260293
754	0.480592
755	0.000000
756	-5.935787
757	0.312375
758	0.000000
759	0.000000
760	0.369092
761	0.000000
768	0.229251
769	-3.694897
770	-4.828034
774	-4.098957
775	0.000000
776	0.284390
777	0.000000
778	0.000000
779	0.231478
780	0.000000
781	-4.201548
782	0.000000
783	-4.476960
817	-4.329649
818	0.195352
819	-3.418231
820	0.233082
821	0.227826
822	0.225886
823	-4.796677
824	-4.147159
825	0.432002
826	-4.216443
827	0.000000
828	0.000000
829	-5.186168
830	0.423058
831	-4.958633
832	-4.483624
950	0.272729
951	-4.089235
952	0.218995
953	0.000000
954	-3.932612
955	0.257122
966	0.385544
967	0.000000
968	0.000000
969	-6.122474
970	0.000000
971	0.000000
976	-6.063085
981	-15.101120

982	0.000000
983	-4.535075
984	0.000000
985	0.657573
986	0.259784
987	-5.399322
994	-4.508313
995	-4.080285
1004	0.281257
1005	0.000000
1006	0.000000
1007	-8.484997
1008	0.000000
1009	-4.343961
1010	0.419661
1012	-4.199743
1014	-5.556973
1017	-11.773829
1018	-3.737939
1019	0.218242
1020	0.000000
1023	-3.845234
1024	-4.677952
1025	0.267320
1026	0.000000
1027	-5.458828
1028	0.000000
1031	0.308320
1032	0.000000
1033	0.000000
1034	0.345980
1036	0.000000
1037	0.000000
1038	0.000000
1039	-5.077204
1040	0.000000
1041	0.301203
1042	0.000000
1043	-4.394886
1044	0.000000
1046	0.000000
1047	0.000000
1048	0.000000
1049	0.000000
1053	0.000000
1054	0.000000
1055	-5.569535
1056	-5.497294
1057	0.000000
1058	0.000000
1059	0.000000
1060	0.000000

Comparison of BCOL and plant CCD8 families (DIVERGE3 output)

Main Tree:

(((((((((((((((((Stickleback_RPE65b:0.068399,Atlantic_cod_RPE65b:0.09
8566):0.023433,Medaka_RPE65b:0.087845):0.011463,(Fugu_RPE65b:0.041910,Puffer
fish_RPE65b:0.059152):0.048048):0.079707,(Zebrafish_RPE65a:0.014403,Zebrafis
h_RPE65b:0.082942):0.066174):0.040581,(Zebrafish_RPE65c:0.068700,(Atlantic_c
od_RPE65a:0.061582,(Stickleback_RPE65a:0.037982,(Medaka_RPE65a:0.048462,(Fug
u_RPE65a:0.046243,Pufferfish_RPE65a:0.035091):0.040925):0.013698):0.010139):
0.023015):0.078251):0.065579,((Mouse_RPE65:0.006208,Rat_RPE65:0.018627):0.03
1675,(Human_RPE65:0.007908,(Dog_RPE65:0.010112,(Cow_RPE65:0.021034,Chicken_R
PE65:0.080586):0.035205):0.007034):0.005327):0.081432):0.053838,Lamprey_RPE6
5:0.156962):0.279142,(BCOa_Oikopleura_dioica:0.385725,(BCOb_Oikopleura_dioic
a:0.038100,BCOc_Oikopleura_dioica:0.108348):0.475331):0.815430):0.110567,(Ci
ona_BCOa:0.697061,(BCOd_Oikopleura_dioica:0.738757,((Caenorhabditis_remanei_
BCOa:0.098374,Caenorhabditis_elegans_BCOa:0.137568):0.071588,(Caenorhabditis
_remanei_BCOb:0.093518,Caenorhabditis_elegans_BCOb:0.094543):0.070622):0.685
540):0.086381):0.046007):0.090757,((Lamprey_BCMO2a:0.010001,Lamprey_BCMO2b:0
.000000):0.368181,(((((Human_BCO2:0.078959,Dog_BCO2:0.063425):0.016634,Cow_B
CO2:0.107281):0.014975,(Mouse_BCO2:0.030337,Rat_BCO2:0.035459):0.076077):0.1
46776,Chicken_BCO2:0.170301):0.119362,(((((Pufferfish_BCO2c:0.123886,Fugu_BC
O2c:0.218005):0.123015,Medaka_BCO2c:0.160912):0.023871,Stickleback_BCO2c:0.0
55828):0.041821,Atlantic_cod_BCO2c:0.250613):0.166014,(((((Pufferfish_BCO2a:
0.109878,Fugu_BCO2b:0.026817):0.061475,Stickleback_BCO2a:0.070147):0.012303,
Medaka_BCO2b:0.093500):0.051329,(Zebrafish_BCO2a:0.138143,Atlantic_cod_BCO2a
:0.101265):0.017697):0.091177,(Zebrafish_BCO2b:0.151383,(Atlantic_cod_BCO2b:
0.118867,((Pufferfish_BCO2b:0.155487,Fugu_BCO2a:0.049695):0.059085,(Medaka_B
CO2a:0.094419,Stickleback_BCO2b:0.091750):0.014665):0.035792):0.095386):0.02
4917):0.083977):0.073192):0.099133):0.193859):0.064861,((((((((Anopheles_gam
biae_NinaB:0.053246,Culex_quinquefasciatus_NinaB:0.072737):0.125444,Drosophi
la_melanogaster_NinaB:0.346824):0.069614,(Nasonia_vitripennis_NinaB:0.213033
,(Acromyrmex_echinatior_NinaB:0.252461,Megachile_rotundata_NinaB:0.119542):0
.061391):0.121200):0.103129,Daphnia_pulex_BCOb:0.451745):0.002495,Bombyx_mor
i_NinaB:0.326058):0.384823,Saccoglossus_kowalevskii_BCOa:0.504167):0.037330,
Daphnia_pulex_BCOa:0.676980):0.097126,(Lamprey_BCMO1:0.766243,(Ciona_BCMOb:0
.690367,((((Pufferfish_BCMO1b:0.055860,Fugu_BCMO1a:0.044936):0.077133,(Stick
leback_BCMO1a:0.120260,Medaka_BCMO1b:0.115422):0.016085):0.083308,Zebrafish_
BCMO1:0.166726):0.174391,((((((Mouse_BCMO1:0.026224,Rat_BCMO1:0.022086):0.02
7119,Human_BCMO1:0.063069):0.010674,Dog_BCMO1:0.042785):0.033148,Cow_BCMO1:0
.091730):0.145080,Chicken_BCMO1:0.150599):0.089086,(Zebrafish_BCMO1a:0.21314
3,(Atlantic_cod_BCMO1:0.189449,(Stickleback_BCMO1b:0.109402,(Medaka_BCMO1a:0
.164722,(Pufferfish_BCMO1a:0.071483,Fugu_BCMO1b:0.076833):0.069118):0.023701
):0.043795):0.094504):0.137426):0.060970):0.142583):0.071356):0.135389):0.05
9423):0.071319,((Lancelet_BCOc_mito:0.359059,Lancelet_BCOd:0.381356):0.16903
9,((Sea_urchin_BCOb:0.421297,Saccoglossus_kowalevskii_BCOb:0.307805):0.28198
4,(Sea_urchin_BCOa:0.582328,(Sea_urchin_BCOc:0.624705,Lancelet_BCOa_lan:0.43
3963):0.101182):0.025529):0.036530):0.036661):0.014307,Lancelet_BCOb:0.50370
5):0.130960,(Nematostella_BCOc:0.556819,(Nematostella_BCOa:0.235924,Nematost
ella_BCOb:0.338452):0.357926):0.076491):0.172148,(((Caenorhabditis_elegans_B
COL:0.017283,Caenorhabditis_remanei_BCOL:0.017703):0.910407,Saccoglossus_kow
alevskii_BCOLa:0.707730):0.469603,(((((Lancelet_BCOLa_1:0.000000,Lancelet_BC
OLa_2:0.085379):0.026099,Lancelet_BCOLd_hybrid:0.042814):0.208354,(Lancelet_
BCOLc:0.195020,Lancelet_BCOLf:0.248298):0.069831):0.191544,(Lancelet_BCOLb:0
.255158,Lancelet_BCOLg:0.274512):0.162605):0.288094,((((Capitella_teleta_BCO
La:0.281278,Capitella_teleta_BCOLb:0.322075):0.294172,Capitella_teleta_BCOLc
:0.656880):0.205643,Lancelet_BCOLe:0.572981):0.022463,(Mimachlamys_nobilis_B
COL:0.607307,(((Crassostrea_gigas_BCOLb:0.176715,Crassostrea_gigas_BCOLc:0.2
61322):0.331106,Octopus_bimaculoides_BCOL:0.503634):0.051622,(Lottia_gigante
a_BCOL:0.282045,(Crassostrea_gigas_BCOLa:0.411239,(Biomphalaria_glabrata_BCO

L:0.246389,Aplysia_californica_BCOL:0.294151):0.200533):0.044692):0.133471):0.093595):0.147926):0.078673):0.386853):0.192683):0.087855,(((CCO_Long_Blastomyces_gilchristii_SLH14081:0.003350,CCO_Blastomyces_gilchristii_SLH14081:0.007252):0.355941,CCO_Pseudogymnoascus_sp_VKMF4246:0.339285):0.533062,(CCO_Aspergillus_oryzae_RIB40:0.447466,(CAO2_torulene_oxygenase_Neurospora_crassa_OR74A:0.345740,(torulene_oxygenase_Beauveria_bassiana_ARSEF2860:0.307199,CarT_torulene_oxygenase_Fusarium_fujikuroi:0.379351):0.160276):0.200831):0.248177):0.313545):0.102027,((CCO_Coccomyxa_subellipsoidea:0.506961,CCD8_Monoraphidium_neglectum:0.818701):0.161076,(CCD8_moss_Physcomitrella_patens:0.299400,(CCD8_Arabidopsis_thaliana:0.219495,((CCD8_Saccharum_ROC22:0.030025,CCD8_Zea_mays:0.052563):0.158550,(CCD8B_Prunus_mume:0.121165,(SiCCD8_Solanum_lycopersicum:0.051035,CCD8_DAD1_Petunia_hybrida:0.064298):0.051689):0.046144):0.093222):0.142349):0.488286):0.266169):0.014082,((BCO1_Halococcus_sediminicola:0.446075,LSD_Haloterrigena_salina:0.299290):0.166361,((BCO1_Natronomonas_moolapensis:0.210278,LSDL_Natronococcus_occultus:0.212016):0.380163,(LSDL_Haloquadratum_walsbyi_J07HQW2:0.296730,(RPE_Haloarcula_amylolytica:0.052269,RPE_Haloarcula_japonica:0.052060):0.173692):0.384941):0.160100):0.188708):0.206633,((PpCCO_Plesiocystis_pacifica:0.887074,NSC3_NosACO_Nostoc_sp._PCC_7120:0.661857):0.358232,(ACO_Auxenochlorella_protothecoides:0.840998,((((((((NSC2_NosACO_Nostoc_sp._PCC_7120:0.148284,ACO_Hapalosiphon_sp._MRB220:0.118564):0.062547,ACO_Scytonema_hofmannii_PCC_7110:0.158399):0.132262,ACO_Microcystis_aeruginosa:0.251511):0.021960,ACO_Synechococcus_sp._PCC73109:0.319991):0.091788,ACO_Cyanobacterium_aponinum:0.288193):0.044068,ACO_Leptolyngbya_valderiana:0.301027):0.075143,ACO_Synechocystis_sp._PCC6803:0.307177):0.113213,ACO_Synechococcus_sp._KORDI52:0.539785):0.357503,((((CCD7_Nicotiana_tomentosiformis:0.034985,CCD7_Petunia_hybrida:0.106436):0.050422,SiCCD7_Solanium_lycopersicum:0.058807):0.246828,CCD7_Zea_mays:0.308886):0.877188,((((((((((LSD_Aspergillus_oryzae:0.123736,LSD_Rco_Aspergillus_fumigatus:0.088856):0.084942,LSD_Aspergillus_kawachii_IFO4308:0.232950):0.095711,(CAO1_CCO_Neurospora_crassa_OR74A:0.008708,LSD_Rco_CAO1_Neurospora_crassa:0.002803):0.359338):0.025362,LSD_Colletotrichum_gloeosporioides_Naragc5:0.223524):0.264679,LSD_Rco_Ustilago_maydis:0.799605):0.102349,LSD_Rco_Chaetomium_globosum:0.519257):0.143656,(NOV1_LSD_Novosphingobium_aromaticivorans:0.340764,(SpaLSDI_Sphingomonas_paucimobilis:0.217654,SpaLSDIII_Sphingomonas_paucimobilis:0.188135):0.141963):0.217110):0.120984,(isoeugenol_monooxygenase_Pseudomonas_putida:0.167913,isoeugenol_monooxygenase_Pseudomonas_nitroreducens:0.129387):0.432792):0.045160,(LSD_Sphingobium_sp_SYK6:0.388999,NOV2_LSD_Novosphingobium_aromaticivorans:0.308304):0.529265):0.245829,(Blr5790_pLSD_Bradyrhizobium_diazoefficiens:0.147585,RpsCCO_Rhodopseudomonas_palustris:0.187105):0.477112):0.231338,((SaCCO_Sphingopyxis_alaskensis:0.527825,MtCCO_Mycobacterium_tuberculosis:0.618367):0.308636,(PPSIR1_17230_CCO_Plesiocystis_pacifica_SIR-
1:0.882277,(((CCD_Ostreococcus_lucimarinus_CCE9901:0.725621,CCD1like_Zea_mays:0.961814):0.252061,(MtLSD_Mycobacterium_tuberculosis:0.460148,Sala_1008CCO_Sphingopyxis_alaskensis:0.333858):0.497316):0.064578,(NSC1_NosCCD_Nostoc_sp._PCC_7120:0.415816,(CarX_CCO_Fusarium_fujikuroi:1.004756,((CCD1_Zea_mays:0.107407,CCD1_Arabidopsis_thaliana:0.121380):0.420187,((((ZCDlong_Zea_mays:0.054191,ZCD_Zea_mays:0.026960):0.201458,NCED4_Arabidopsis_thaliana:0.331303):0.116672,(CCD4a_CsZCD_Crocus_sativus:0.058217,CCD4b_CsZCD_Crocus_sativus:0.028859):0.305676):0.395002,(NCED6_Arabidopsis_thaliana:0.303802,((NCED1_Zea_mays:0.156037,VP14_Zea_mays:0.184512):0.090039,((NCED9_Arabidopsis_thaliana:0.195941,NCED3_Arabidopsis_thaliana:0.115309):0.044526,(NCED5_Arabidopsis_thaliana:0.147493,NCED2_Arabidopsis_thaliana:0.177308):0.065273):0.085851):0.108712):0.300292):0.252979):0.191806):0.077731):0.108284):0.073833):0.078074):0.083978):0.242261):0.178638):0.030721):0.068440):0.234904):0.000000
Cluster BCOL:
((Mimachlamys_nobilis_BCOL:0.607307,(((Crassostrea_gigas_BCOLb:0.176715,Crassostrea_gigas_BCOLc:0.261322):0.331106,Octopus_bimaculoides_BCOL:0.5036

34):0.051622,(Lottia_gigantea_BCOL:0.282045,(Crassostrea_gigas_BCOLa:0.411239,(Biomphalaria_glabrata_BCOL:0.246389,Aplysia_californica_BCOL:0.294151):0.200533):0.044692):0.133471):0.093595):0.147926,((((Lancelet_BCOLa_1:0.000000,Lancelet_BCOLa_2:0.085379):0.026099,Lancelet_BCOLd_hybrid:0.042814):0.208354,(Lancelet_BCOLc:0.195020,Lancelet_BCOLf:0.248298):0.069831):0.191544,(Lancelet_BCOLb:0.255158,Lancelet_BCOLg:0.274512):0.162605):0.288094,(((Capitella_teleta_BCOLa:0.281278,Capitella_teleta_BCOLb:0.322075):0.294172,Capitella_teleta_BCOLc:0.656880):0.205643,Lancelet_BCOLe:0.572981):0.022463):0.000000

Cluster CCD8_Plant:

((CCD8_Arabidopsis_thaliana:0.219495,((CCD8_Saccharum_ROC22:0.030025,CCD8_Zea_mays:0.052563):0.158550,(CCD8B_Prunus_mume:0.121165,(SiCCD8_Solanum_lycopersicum:0.051035,CCD8_DAD1_Petunia_hybrida:0.064298):0.051689):0.046144):0.093222):0.142349,(CCO_Coccomyxa_subellipsoidea:0.506961,CCD8_Monoraphidium_neglectum:0.818701):0.161076,CCD8_moss_Physcomitrella_patens:0.299400):0.000000

Da	6.199921
Db	2.523568
N	33.000000
C	16.000000
R	24.000000
Alpha ML	1.540650
Theta-II	-0.653998
Theta SE	0.646897
Ar	0.446104
PIr	0.312130
p	0.547945
d	0.547945
W	0.049693
Z	0.053838
Gr	0.600000
Gc	0.400000
h	0.050679
Q	0.960000
F00,N	0.082192
F00,R	0.000000
F00,C	0.000000

329	-1.203115
330	0.000000
331	-1.919249
332	0.000000
333	-1.121456
334	-2.056734
335	-0.728401
336	-0.935854
337	0.000000
363	-0.437844
364	-0.825735
520	-0.962769
521	0.000000
522	0.000000
523	0.000000
524	0.000000
525	0.000000
526	0.000000

594	0.000000
595	0.000000
596	0.000000
597	-0.546305
598	-1.198939
599	-2.221001
626	-0.881475
629	-0.567802
630	-0.606130
631	-1.125374
632	-0.660608
633	-0.821807
634	-0.869769
721	0.000000
722	-2.134827
723	-1.021423
724	0.000000
725	-0.879714
743	-1.142773
744	0.000000
745	0.000000
746	0.000000
747	-1.333097
882	-1.044466
883	-0.481407
884	-1.437287
885	-1.026469
886	-1.186824
887	0.000000
888	-1.814559
889	-0.804485
890	0.000000
891	0.000000
892	0.000000
1005	-0.985623
1006	-0.904869
1007	-1.017262
1008	0.000000
1009	0.000000
1010	0.000000
1011	0.000000
1670	-2.081414
1671	0.000000
1672	0.000000
1673	-2.430972
1674	-0.837455
1675	-1.002382
1726	0.000000
1727	0.000000
1728	0.000000
1729	-0.906460
1730	0.000000
1731	0.000000
1732	0.000000
1733	-1.176497
